# Expanding the Staphylococcus aureus SarA Regulon to Small RNAs

**DOI:** 10.1128/mSystems.00713-21

**Published:** 2021-10-12

**Authors:** Charlotte Oriol, Liviu Cengher, Adhar C. Manna, Tony Mauro, Marie-Laure Pinel-Marie, Brice Felden, Ambrose Cheung, Astrid Rouillon

**Affiliations:** a Rennes 1 University, INSERM, BRM [Bacterial Regulatory RNAs and Medicine], UMR_S 1230, Rennes, France; b Department of Microbiology and Immunology, Geisel School of Medicine at Dartmouth, Hanover, New Hampshire, USA; University of California, San Francisco

**Keywords:** *S. aureus*, ChIP-Seq, RNA-Seq, SarA regulon, sRNA

## Abstract

SarA, a transcriptional regulator of Staphylococcus aureus, is a major global regulatory system that coordinates the expression of target genes involved in its pathogenicity. Various studies have identified a large number of SarA target genes, but an in-depth characterization of the *sarA* regulon, including small regulatory RNAs (sRNAs), has not yet been done. In this study, we utilized transcriptome sequencing (RNA-Seq) and chromatin immunoprecipitation sequencing (ChIP-Seq) to determine a comprehensive list of SarA-regulated targets, including both mRNAs and sRNAs. RNA-Seq analysis indicated 390 mRNAs and 51 sRNAs differentially expressed in a Δ*sarA* mutant, while ChIP-Seq revealed 354 mRNAs and 55 sRNA targets in the S. aureus genome. We confirmed the authenticity of several novel SarA targets by Northern blotting and electrophoretic mobility shift assays. Among them, we characterized repression of *sprG2*, a gene that encodes the toxin of a type I toxin-antitoxin system, indicating a multilayer lockdown of toxin expression by both SarA and its cognate antitoxin, SprF2. Finally, a novel SarA consensus DNA binding sequence was generated using the upstream promoter sequences of 15 novel SarA-regulated sRNA targets. A genome-wide scan with a deduced SarA motif enabled the discovery of new potential SarA target genes which were not identified in our RNA-Seq and ChIP-Seq analyses. The strength of this new consensus was confirmed with one predicted sRNA target. The RNA-Seq and ChIP-Seq combinatory analysis gives a snapshot of the regulation, whereas bioinformatic analysis reveals a permanent view of targets based on sequence. Altogether these experimental and *in silico* methodologies are effective to characterize transcriptional factor (TF) regulons and functions.

**IMPORTANCE** Staphylococcus aureus, a commensal and opportunist pathogen, is responsible for a large number of human and animal infections, from benign to severe. Gene expression adaptation during infection requires a complex network of regulators, including transcriptional factors (TF) and sRNAs. TF SarA influences virulence, metabolism, biofilm formation, and resistance to some antibiotics. SarA directly regulates expression of around 20 mRNAs and a few sRNAs. Here, we combined high-throughput expression screening methods combined with binding assays and bioinformatics for an in-depth investigation of the SarA regulon. This combinatory approach allowed the identification of 85 unprecedented mRNAs and sRNAs targets, with at least 14 being primary. Among novel SarA direct targets, we characterized repression of *sprG2*, a gene that encodes the toxin of a toxin-antitoxin system, indicating a multilayer lockdown of toxin expression by both SarA and its cognate antitoxin, SprF2.

## INTRODUCTION

The Gram-positive bacterium Staphylococcus aureus resides as a commensal in about one-third of the human population (1) but can easily become a pathogen that causes a variety of diseases, ranging from mild to life-threatening infections such as endocarditis, pneumonia, bacteremia, and toxic shock syndrome (2). The emergence of multidrug-resistant strains and the lack of an effective vaccine are responsible for the burden of hard-to-treat acute and chronic staphylococcal infections. The pathogenicity of S. aureus is a complex process that depends upon coordinated and timely expression of virulence factors controlled by a network of regulators, including two-component regulatory systems, transcriptional factors (TFs), small regulatory RNAs (sRNAs), and small signaling molecules (3). Among transcriptional regulators, SarA is a global regulatory transcription element in S. aureus (4, 5). The *sarA* promoter region is extensive (∼800 bp), comprising three distinct promoters (P2, P3, and P1) that produce three distinct overlapping transcripts (P2 *sarA*, P3 *sarA*, and P1 *sarA*), each carrying the major 372-bp *sarA* open reading frame (ORF), which yields the 14.7-kDa SarA protein (6). Within this long promoter region, two sRNAs known as Teg49 and Teg48 have been characterized, with Teg49 being involved in the stability of the P3 *sarA* transcript and regulation of virulence genes independently of SarA (7, 8).

SarA, a homolog of MarR in Gram-negative bacteria, is a winged-helix DNA-binding protein that binds specific DNA sequences by interacting with specific amino acid residues within SarA (i.e., R84 and R90) for the regulation of its targets (9). The phosphorylation status of SarA, which involves *in vivo* and *in vitro* phosphorylation of threonine/serine residues, modifies its DNA binding abilities (10). Affinity of SarA for the *agr* B1/B2 binding site increases with the level of reducing agent and decreases with the level of oxidizing agent, and this involves the unique cysteine residue present in SarA protein (11). This redox-sensing residue, Cys9, is conserved between Sar family members. It may allow a thiol switch or phosphorylation and could represent the major site of phosphorylation in the SarA/MgrA family proteins (12–14). SarA binds to a conserved operator 26-bp sequence (ATTTGTATTTAATATTTATATAATTG) located in the upstream promoter region of several SarA-regulated target genes (13, 15–18). Interestingly, whereas Sterba et al. (19) proposed a 7-bp SarA consensus (ATTTTAT) motif, at least two 7-bp consensus motif sequences can, with some variation, be located within the conserved operator sequence described previously and could anchor the SarA dimer.

Considering the binding properties of SarA, which binds as a dimeric form of a flexible winged-helix structure with more than 40 Å distance between two wings (9), it is reasonable to predict that a functional SarA binding site would consist of at least two consensus binding motifs with any orientation (palindrome or inverted repeats) that could lead to regulation of a large number of targets in the S. aureus genome. In addition to SarA binding to DNA, it has been shown that it may bind to mRNA to influence its turnover (20). Nevertheless, published observations have suggested that SarA’s regulatory effects might be more complex than those of any other transcriptional regulators. SarA upregulates the synthesis of fibronectin- and fibrinogen-binding proteins, hemolysins (alpha-, beta-, and gamma-hemolysins), enterotoxins, toxic shock syndrome toxin 1, oxidative stresses (*sodM* and *trxB*), and genes involved in biofilm formation (e.g., *icaRA* and *bap*) and repressed expression of proteases (*ssp* and *aur*), protein A (*spa*), and collagen-binding proteins (*cna*) (4, 5, 13, 18, 21, 22). SarA regulates many genes indirectly by modulating the expression of other regulatory loci (e.g., *rot*, *agr*, *sarS*, *sarV*, and *sarT*) (9, 15, 16, 23–25). Deletion of *sarA* in S. aureus affects large numbers of target genes (∼120 genes) involved in virulence or metabolic processes, as determined by microarray analyses (26). SarA is the first and well-characterized member of a family of proteins called the SarA protein family based on protein sequence alignment. Nine other SarA paralogues (i.e., SarR, SarS, SarT, SarU, SarV, SarX, SarZ, MgrA, and Rot) have been characterized and are found to regulate a large number of target genes, including those involved in virulence, biofilm formation, autolysis, antibiotic resistance, and metabolic processes (5).

Various studies have also shown that SarA regulates transcription of at least three sRNAs: *RNAIII*, s*rn_3610_sprC*, and *srn_9340* (6, 15, 27, 28). The Staphylococcal Regulatory RNAs Database (SRD) lists around 500 potential sRNAs in S. aureus genomes (29), while very few of them have been characterized for their role in target gene regulation and only 50 of them have been classified as bona fide sRNAs (30). Typically, sRNA base-pairs with specific mRNA target to regulate their stability and translation efficiency (31). In the last decade, it was shown that a number of sRNAs are involved in the regulation of crucial cell processes such adaptation to environmental changes and virulence in various organisms, including S. aureus (32, 33). The best-described sRNA in S. aureus is RNAIII, a dual-function sRNA, which codes for delta toxin as well as functioning as an sRNA that binds target mRNAs to promote mRNA degradation (e.g., mRNAs for *spa*, *coa*, *sbi*, *ltaS*, *lytM*, *rot*, and others) or improve translation (e.g., *hla*, *map*, and *mgrA*) (34–36). These examples highlight the complexity and regulatory aspects of sRNA being an alternative regulator for target regulations under environmental changes. There are several well-characterized sRNAs, including RsaD, RsaA, SprC, and ArtR, for example. RsaD posttranscriptionally regulates *alsS* (acetolactate synthase) mRNA and enzyme levels (37), and its transcription is repressed by CodY, while the sRNA RsaA is known to repress the synthesis of the transcriptional regulator MgrA (38). Srn_3610_SprC, directly repressed by SarA, has been shown to reduce virulence and bacterial loads in a mouse infection model and to negatively regulate the major staphylococcal autolysin by sRNA-mRNA base-pairing (39). ArtR is involved in activating alpha toxin expression by targeting the 5′ untranslated region (UTR) of *sarT* mRNA (40). When sRNA is described , type I toxin-antitoxin (TA) systems must be cited. In this TA system, an antisense RNA plays the role of antitoxin to prevent the synthesis of its cognate toxin by directly base-pairing to the mRNA (41). Type I TA systems, like other TA systems, are not essential for normal cell proliferation but allow a growth slowdown or redirect bacterial metabolic resources until growth conditions improve and therefore represent a crucial element of S. aureus adaptation. SprA1_AS_ and SprF1 are examples of antitoxin sRNAs from functionally characterized SprA1/SprA1_AS_ and SprG1/SprF1 type I TA systems (42, 43).

Here, we present an extensive analysis of the SarA regulon by differential transcriptome sequencing (RNA-Seq) between the wild type and a *sarA* mutant strain and chromatin immunoprecipitation sequencing (ChIP-Seq) with anti-SarA-Myc. We obtained 441 (390 mRNAs and 51 sRNAs) and 409 (354 mRNAs and 55 sRNAs) targets regulated by SarA in RNA-Seq and ChIP-Seq, respectively. By combining both approaches, we identified 140 targets in common and 85 unprecedented characterized targets (72 mRNAs and 13 sRNAs). Among these 85 new targets, 16 targets (both mRNAs and sRNAs) were validated using genetic and biochemical approaches. We also identified three important sRNAs (i.e., *srn_2230_sprG2*, encoding type I toxin sRNA; *srn_4540_sprA2_AS_*, encoding type I antitoxin sRNA; and s*rn_1640_rsaD*), which were characterized for the authenticity of SarA-mediated regulation even if the last two sRNA targets did not appear as SarA common RNA-Seq/ChIP-Seq targets. In the SprG2/SprF2 TA system, SprG2 regulation involves SarA at the transcription level, whereas SprF2 sRNA antitoxin regulates SprG2 translation. Such a dual control probably allows S. aureus to finely tune the quantity of SprG2 peptide, which could lead to bacteriostasis or lethality. Finally, bioinformatics analysis led to the identification of a SarA consensus binding/regulatory DNA motif. Genome-wide scanning revealed several new SarA targets, which are not found in either RNA-Seq and ChIP-Seq analyses, among which one sRNA promoter was proven to be bound directly by SarA, using *in vitro* electrophoretic mobility shift assay (EMSA) studies. Overall, results from both approaches identified new SarA targets, including sRNAs for which identification of the TF responsible for variation in their expression may probably facilitate functional characterization.

## RESULTS

### Investigation of the SarA-dependent transcriptome in S. aureus strain HG003.

SarA is a pleiotropic transcriptional regulator involved in regulation of virulence, biofilm formation, oxidative stresses, and accessory antibiotic resistance factors, either directly by binding to the upstream promoter region or indirectly by regulating through other regulatory systems (44–48). The known targets of SarA are mostly mRNAs, with a few sRNAs (e.g., *RNAIII*, *srn_3610_SprC*, and *srn_9340*) (6, 15, 18, 22, 25–28, 46, 49–52). At present, genome-wide annotations of sRNAs in staphylococci are available (29, 30, 53), but the regulation of their expression is largely unknown. To determine the genome-wide SarA-regulated targets, we analyzed the transcriptomes of the wild-type HG003 and isogenic Δ*sarA* mutant strains (Table S1A) grown to exponential (E) or early stationary (S) phase (Fig. S1). We worked with HG003, an NCTC 8325 derivative. NCTC 8325 (RN1) is a S. aureus strain isolated from a sepsis patient in 1960 that is widely used for genetic and physiological studies (54). This strain is defective for two main regulators encoded by *rsbU* and *tcaR*: a positive activator of the general stress response regulator σ^B^ and a transcriptional activator of the protein A-encoding gene, respectively. HG003 has been repaired for both *rsbU* and *tcaR* genes (55) and is increasingly used by scientific community for studying regulation, biofilm formation, or antibiotic tolerance (30, 56–58).

10.1128/mSystems.00713-21.1 FIG S1 (A) Graph showing optical density as a function of time. Growth of HG003 (blue) and HG003 Δ*sarA* (black) strains is presented in a semilogarithmic scale. (B) Graph showing optical density as a function of time. Growth of HG003 + pCN36 (blue), HG003 + pCN36-*sarA* (orange), HG003 Δ*sarA* + pCN36 (grey), and HG003 Δ*sarA* + pCN36-*sarA* (yellow) strains is presented in a semilogarithmic scale. Download FIG S1, TIF file, 1.6 MB. Copyright © 2021 Oriol et al. 2021 Oriol et al. https://creativecommons.org/licenses/by/4.0/ This content is distributed under the terms of the Creative Commons Attribution 4.0 International license.

10.1128/mSystems.00713-21.5 TABLE S1 Strains and plasmids (A) and primers (B) used in this study. Download Table S1, DOCX file, 0.02 MB. Copyright © 2021 Oriol et al. 2021 Oriol et al. https://creativecommons.org/licenses/by/4.0/ This content is distributed under the terms of the Creative Commons Attribution 4.0 International license.

**FIG 1:**
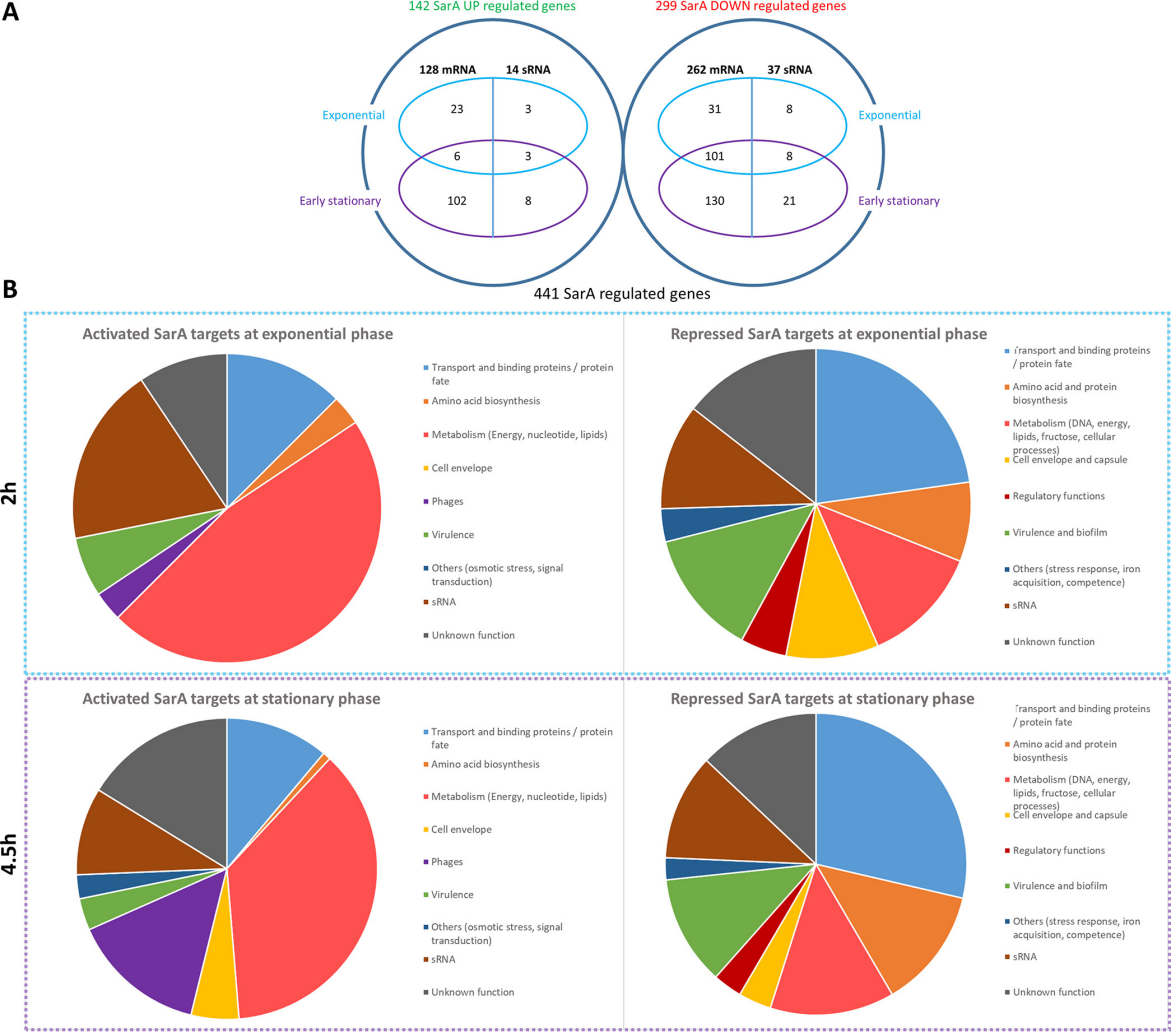
Transcriptomic studies of HG003 and HG003 Δ*sarA* strains. (A) Global visualization of SarA regulated genes detected through RNA-Seq experiments. (B) SarA target classification according to function. SarA-repressed targets are on the right, and SarA-activated targets are on the left.

An HTSeq/DESeq pipeline detected genes whose expression varies in the absence of the *sarA* gene. The thresholds used to obtain SarA targets were a fold change greater than or equal to 3 between wild-type HG003 and isogenic Δ*sarA* mutant strains combined with a minimum number of fragments per kilobase per millions of fragments mapped (FPKM) of 10 under at least one growth condition. RNA-Seq data indicated that the expression of 142 genes is activated (128 mRNA-encoding genes and 14 sRNA-encoding genes) and 299 (262 mRNA genes and 37 sRNA genes) repressed in the *sarA* mutant compared to the wild-type strain (Table S2). Among the activated transcripts, 25 were detected at the E phase and 110 at the S phase (Fig. 1A). The expression of 3 sRNA genes and 3 mRNA genes was induced at both phases of growth. Among the repressed transcripts, 132 are mRNAs and 16 are sRNAs repressed at E phase, 231 are mRNAs and 29 are sRNAs repressed at S phase, and 101 are mRNAs and 8 are sRNAs repressed at both growth phases (Fig. 1A). The number of regulated genes is twice as great at the early stationary growth phase as at the exponential growth phase in the HG003 Δ*sarA* strain despite the observation that overall SarA protein levels are known not to fluctuate significantly with growth phase (18, 59, 60). The implication is that SarA mainly regulates target genes during stationary growth phase (such as secreted proteins, toxins, and metabolic processes). As SarA is a protein that can be modified in ways that change its ability to bind promoters (e.g., oxidation and phosphorylation), it is likely that the higher number of targets at early stationary phase is linked either to posttranslational modification on SarA at the Cys9 residue or to multiple transcriptional regulators working in tandem in distinct cellular phases (9, 10, 14).

10.1128/mSystems.00713-21.6 TABLE S2 Complete RNA-Seq data. Selection shows genes with FPKM higher than 10 with three or more transcriptional variations between HG003 and HG003 Δ*sarA* mutant strains. Download Table S2, DOCX file, 0.1 MB. Copyright © 2021 Oriol et al. 2021 Oriol et al. https://creativecommons.org/licenses/by/4.0/ This content is distributed under the terms of the Creative Commons Attribution 4.0 International license.

Our RNA-Seq data show that the *sarA*-repressed targets are predominantly transport/binding proteins and proteins involved in protein fate, such as type VII secretion protein or proteases. The activated targets are mainly involved in central metabolism, like pyrimidine nucleotide biosynthesis (Fig. 1B; Table S2). The data confirm that among SarA-repressed targets, expression of several proteases, including those encoded by *sspA*, *sspB*, and *sspC* (SAOUHSC_00986-00988) and aureolysin (encoded by *aur*; SAOUHSC_02971) is controlled by SarA, in agreement with a known increase in protease production in *sarA* mutants (61). Moreover, *splA* to *-F* serine protease expression is strongly increased in the absence of *sarA*, as previously described for the S. aureus osteomyelitis model (62). Both serine and cysteine protease expression must be finely tuned due to their participation in biofilm formation, since it has been shown that more serine or/and cysteine proteases would lead to reduced biofilm formation (61). SarA also regulates genes involved in structure of biofilm like the *ica* operon (SAOUHSC_03002-3005) and *capA1* (SAOUHSC_03000), which are both repressed by SarA (Table 1; Table S2) (63, 64). The expression of the thermonuclease gene *nuc* (SAOUHSC_00818) was increased 130- and 269-fold in the *sarA* mutant at E and S phases, respectively (Table 1), which is in agreement with previously demonstrated repression in *sarA* mutants (65). RNA-Seq data also confirmed the repression of previously published two sRNA targets, *srn_3610_sprC* and *srn_9340* (Table 1; Table S2) (28).

**TABLE 1:** Extraction of RNA-Seq data with genes repressed by SarA^*a*^

Description and annotation	Gene name	Assignment	Differential expression in HG003 Δ*sarA* vs HG003 at growth phase			
			Exponential (2 h)		Early stationary (4.5 h)	
			Fold change	*P* value	Fold change	*P* value
Genes repressed by SarA						
Regulatory functions						
SAOUHSC_00674	*sarX*	Staphylococcal accessory regulator family	4.1	7.20E−44	3.7	3.29E−09
SAOUHSC_00694	*mgrA*	Staphylococcal accessory regulator family			3.8	6.70E−10
SAOUHSC_00818	*nuc*	Thermonuclease	130.1	4.46E−277	269.4	4.52E−115
SAOUHSC_00913	*lysR*	LysR family regulatory protein	65.1	5.18E−208	42.8	2.02E−71
SAOUHSC_00992	*atlR*	MarR family transcriptional regulator	17.3	8.53E−136	28.7	1.53E−54
SAOUHSC_01402	*msa*	Protein Msa (modulator of *sarA*)	4.8	3.48E−46	3.4	1.32E−09
** SAOUHSC_02569**	*sarY*	Staphylococcal accessory regulator family	8.8	8.35E−80	5.6	2.82E−16
** SAOUHSC_02570**		AraC family transcriptional regulator	11.4	1.21E−112	6.0	2.74E−20

Virulence						
SAOUHSC_00061		Myosin-cross-reactive antigen	11.3	1.58E−116	21.5	1.86E−46
SAOUHSC_00069	*spa*	Protein A			3.2	3.98E−08
SAOUHSC_00544	*sdrC*	Fibrinogen-binding protein SdrC	6.8	1.52E−73	5.4	8.24E−17
SAOUHSC_00545	*sdrD*	Fibrinogen-binding protein SdrD	8.0	4.39E−91	5.2	7.44E−15
SAOUHSC_00968		Bacteriocin-associated integral membrane protein			5.0	1.33E−15
SAOUHSC_01121	*hla*	Alpha-hemolysin	5.6	3.44E−39		
SAOUHSC_01448	*norB*	Quinolone resistance protein	13.3	3.53E−127	24.5	1.66E−55
** SAOUHSC_01954**	*lukD*	Leukotoxin LukD	6.4	5.27E−30	10.1	3.63E−27
** SAOUHSC_01955**	*lukE*	Leukotoxin LukE	9.5	1.76E−38	11.6	3.00E−29
SAOUHSC_02127	*scpA*	Staphopain thiol proteinase	126.7	0.00E + 00	57.2	2.25E−71
SAOUHSC_02129		Staphostatin A	65.3	5.34E−258	51.5	2.25E−71
SAOUHSC_02167	*scn*	Staphylococcal complement inhibitor SCIN	3.3	7.36E−33	5.6	6.14E−17
SAOUHSC_02171	*sak*	Staphylokinase	26.7	5.60E−186	30.1	7.57E−59
SAOUHSC_02169	*chp*	Chemotaxis-inhibiting protein CHIPS	16.6	1.45E−98	4.2	9.39E−10
** SAOUHSC_02241**	*lukG*	Leukocidin LukG	6.0	4.85E−67	5.7	4.99E−18
** SAOUHSC_02243**	*lukH*	Leukocidin LukH	6.9	5.09E−75	5.9	2.85E−19
SAOUHSC_02463	*hysA*	Hyaluronate lyase	8.0	2.48E−84	3.8	6.20E−12
SAOUHSC_02611	*lyrA*	Lysostaphin resistance protein A			3.3	1.96E−08
SAOUHSC_02696	*fmhA*	Methicillin resistance determinant protein (FemAB family)	38.9	1.01E−198	21.0	2.98E−51
SAOUHSC_02706	*sbi*	Immunoglobulin G-binding protein Sbi	9.3	1.75E−98	13.3	6.55E−39
** SAOUHSC_02709**	*hlgC*	Leukocidin s subunit			11.2	2.96E−32
** SAOUHSC_02710**	*hlgB*	Leukocidin f subunit			9.8	1.27E−28
SAOUHSC_02740		Drug resistance MFS transporter, drug:H+ antiporter-2			3.0	2.75E−07
SAOUHSC_02851	*cidA*	Holin-like protein CidA			4.1	7.94E−13
SAOUHSC_02883	*ssaA*	Secretory antigen SsaA (LysM domain-containing protein)	4.7	1.33E−50	4.2	4.44E−14
SAOUHSC_02963	*clfB*	Clumping factor B			5.6	7.01E−17
SAOUHSC_02971	*aur*	Zinc metalloproteinase aureolysin	467.8	0.00E + 00	162.0	4.69E−103

Biofilm						
** SAOUHSC_03002**	*icaA*	*N*-Glycosyltransferase			59.4	7.71E−60
** SAOUHSC_03003**	*icaD*	Intracellular adhesion protein D			21.7	3.66E−14
** SAOUHSC_03004**	*icaB*	Intercellular adhesion protein B			15.5	1.65E−25
** SAOUHSC_03005**	*icaC*	Intercellular adhesion protein C			4.6	2.10E−12

Stress response						
SAOUHSC_00093	*sodM*	Superoxide dismutase	14.0	1.15E−132	4.5	3.59E−13
SAOUHSC_02949	*gpxA2*	Putative glutathione peroxidase	6.3	1.77E−61	8.0	3.35E−26

sRNA transcripts^*b*^						
* srn_3610_sprC* (154 nt)			7.1	4.72E−64	8.9	6.84E−28
* srn_9340_sRNA287* (116 nt)			13.9	1.20E−50	24.8	1.66E−52

a Each gene pair or group in boldface is an operon structure. The AureoWiki database (https://aureowiki.med.uni-greifswald.de/Main_Page) was used to describe the gene annotation and assignment (103), and the SRD database was used for sRNA (http://srd.genouest.org/).

b Known SarA targets (28).

Among the SarA targets previously identified, the expression of gamma-hemolysin (HlgACB) leukotoxin genes *hlgC* and *hlgB* (*SAOUHSC_02709* and *SAOUHSC_02710*), which were previously shown by microarray to be decreased in the absence of *sarA* (26), was found in this study to be increased in the absence of *sarA* (Table S2). Leukotoxins participate in S. aureus virulence by targeted killing of host immune cells (66). We determined that the expression of other gene pairs producing leukotoxins are also increased in the absence of *sarA*, including *lukG*-*lukH* (SAOUHSC_02241-SAOUHSC_02243) and *lukD*-*lukE* (SAOUHSC_01954-SAOUHSC_01955). Altogether, all leukotoxin systems that are present in HG003 appear to be repressed by SarA. Although the role of SarA in the transcriptional regulation of pyrimidine operon (SAOUHSC_01165-01172) was described previously (67), *sarA*-mediated activation of the purine operon (SAOUHSC_01008-01012) and genes implicated in fatty acid metabolism (SAOUHSC_00195 to SAOUHSC_00198) are novel discoveries (Table S2).

Altogether, the RNA-Seq data revealed that the expression of 390 mRNAs and 51 sRNAs is influenced by *sarA* inactivation, of which 46 mRNAs and 3 sRNAs were previously characterized (13, 16, 18, 22, 25–27, 46, 49–52, 68). Therefore, the transcription of 344 novel mRNAs and 48 new sRNAs was altered in the absence of *sarA*, as determined by RNA-Seq using two different phases of growth.

### Identification of SarA-bound regions on a genome-wide scale.

**FIG 2:**
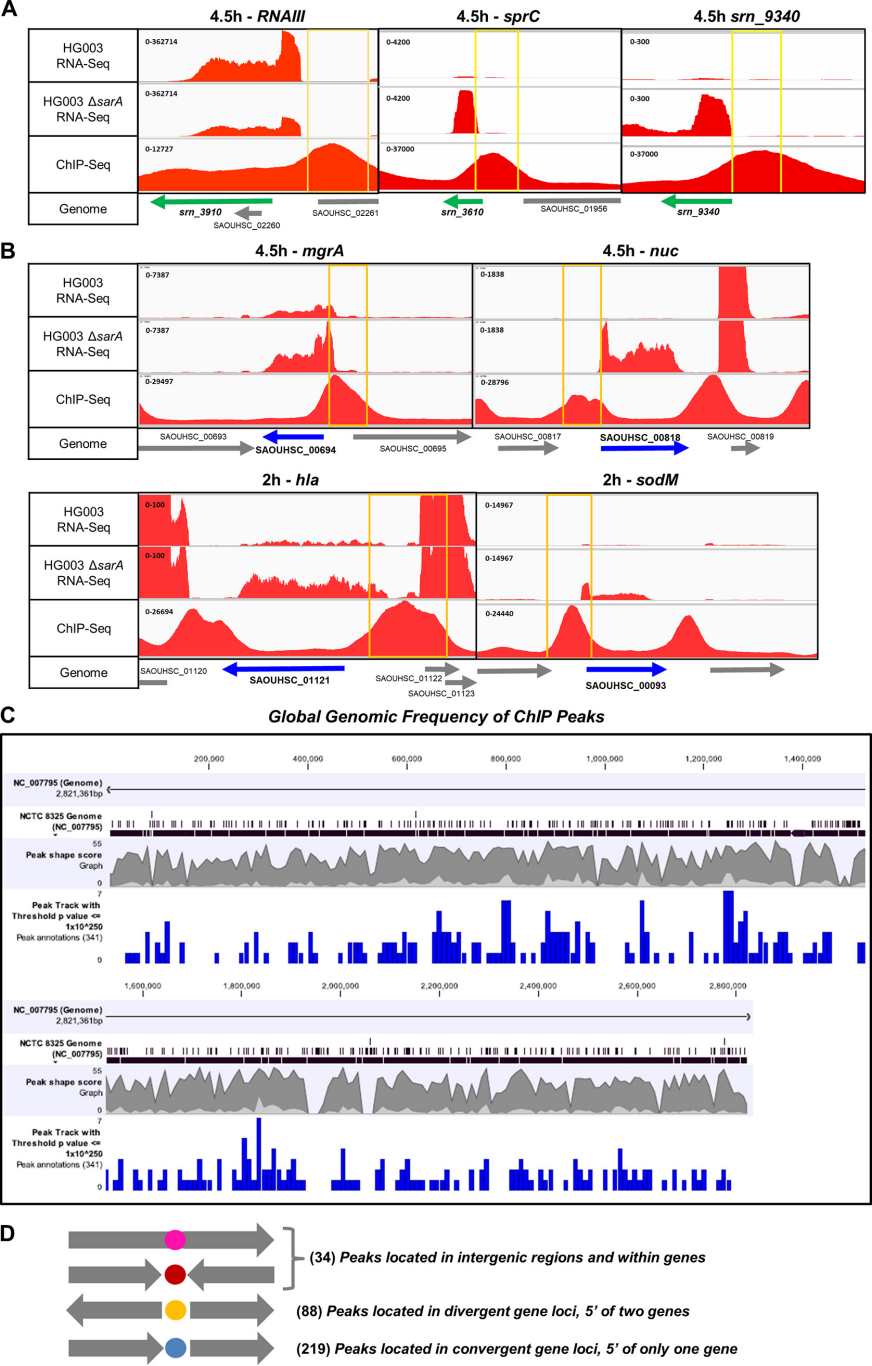
IGV visualization of RNA-Seq and ChIP-Seq results with known SarA targets: RNA-Seq reads and SarA DNA binding peak on chosen genomic locus. Comparison of RNA-Seq profiles of HG003 WT and HG003 Δ*sarA* strains with ChIP-Seq profiles with IGV visualization of (A) the known SarA sRNA targets RNAIII, Srn_3610_SprC, and Srn_9340 at 4.5 h of growth and (B) the known SarA mRNA targets SAOUHSC_00694 (*mgrA*) and SAOUHSC_00818 (*nuc*) at 4.5 h and SAOUHSC_00093 (*sodM*) and SAOUHSC_01121 (*hla*) at 2 h of growth. sRNA genes are indicated with green arrows and mRNA genes with blue arrows. The yellow box represents the ChIP-Seq peak region upstream of the SarA regulated gene. (C) Global genomic frequency of ChIP peaks across the S. aureus chromosome. (D) Targets sorting in different categories. The 341 putative SarA binding sites (ChIP peaks of E^−250^) are represented with respect to staphylococcal genes. The gray arrows represent the genes and their orientation. Pink and red circles represent 34 putative SarA binding sites inside a gene region and downstream of the gene region, respectively. The yellow circle shows 88 putative SarA binding sites upstream of 2 gene regions. The blue circle indicates 219 putative SarA binding sites upstream of a single gene.

To determine *in vivo* DNA binding regions of SarA on a genomic scale, we performed ChIP-Seq analysis (69) using an anti-SarA monoclonal antibody with wild-type cell extracts or an anti-Myc antibody with cell extracts of a *sarA* mutant harboring pSK236::P1*sarA-myc*. Growth of the *sarA* mutant harboring pSK236::P1*sarA-myc* was shown to be similar to that of the wild-type HG003 strain, and Western blotting revealed an equivalent level of SarA in both strains (Fig. S2A and B). In examining SarA protein retained in cross-linked DNA fragments, an anti-Myc antibody showed cleaner pulldown than the anti-SarA antibody, which had multiple bands (Fig. S2C and D). Cross-linked protein-DNA complexes containing Myc-tagged SarA were sheared and immunoprecipitated with anti-Myc monoclonal antibody and protein A-bound Sepharose beads. The DNA fragments were then blunted, ligated to adaptors, and identified by sequencing using the Illumina HiSeq 4000 system. The resulting reads were mapped to NC_007795, the common reference for HG003 and the SRD sRNA database (29, 56). All gene sequences with enrichment peaks compatible with transcriptional regulation were listed, and some examples of local enrichment are shown for both sRNAs and mRNAs (Fig. 2A and B).

10.1128/mSystems.00713-21.2 FIG S2 ChIP-Seq strain controls. (A) Graph showing optical density as a function of time. Growth of HG003 (blue), HG003 Δ*sarA* (red), HG003 + pSK236 (green), and HG003 Δ*sarA* + pSK236:*sarA*-myc (orange) is represented in a semilogarithmic scale. (B) Western blot showing SarA protein levels using anti-SarA antibodies (top). Deposit was controlled with Coomassie staining (bottom). Western blot analysis of the cross-lined sheared DNA fragments containing HG003 Δ*sarA* (pSK236-P1*sarA*-*myc*) cell lysates. (C) Ethidium bromide-stained 1.0% agarose gel containing cross-linked sheared lysates. (D) Western blots of anti-Myc (blot 1) and anti-SarA (blot 2) antibodies to cross-linked sheared lysates and detected by horseradish peroxidase (HRP)-conjugated respective secondary antibodies. Two samples were used, and equal amounts of cell lysate were loaded for both blots. Download FIG S2, TIF file, 2.3 MB. Copyright © 2021 Oriol et al. 2021 Oriol et al. https://creativecommons.org/licenses/by/4.0/ This content is distributed under the terms of the Creative Commons Attribution 4.0 International license.

The ChIP-Seq data obtained at the early stationary phase of growth (Fig. S2A) revealed 341 SarA binding sites/peaks with a threshold peak shape score (PSS) of 30.0 and *P* value of 1 × 10^−250^ or less, with a mix of coordinated, convergent, and divergent gene orientations around them (Table S3). In addition, we directly looked and confirmed these peaks to be in the 5′ regions of sRNAs or genes using CLC Workbench and Integrative Genomics Viewer (IGV). These stringent cutoffs were set to minimize the number of false-positive results and to increase the viability of motifs generated from selected regions. Of these 341 peaks, 307 appeared in the upstream promoter region of sRNAs or mRNAs, and 34 did not match any annotated promoters or are situated within the coding regions. Considering that SarA binding could be in the promoters of both convergent and divergent genes, we found 88 SarA binding peaks in the divergent genes. Binding of SarA occurred all along the genome with various peak intensities, though some regions seem to have higher binding than others (Fig. 2C). SarA had a lower strand coefficient (1.015) than expected for TFs, indicating some promiscuity in binding site and a wide distribution of *sarA* peaks. Overall, we identified 409 SarA binding targets in the S. aureus genome, which include both sRNA (55) and mRNA (354) targets (Table S3; Fig. 2D).

10.1128/mSystems.00713-21.7 TABLE S3 Selection of ChIP-Seq peak with a *P* value of 1 × 10^−250^ or less. mRNA genes appear with a yellow background, whereas sRNA genes have a blue background. Green highlighting is used where the ChIP peak could be associated with sRNA and mRNA genes. Download Table S3, DOCX file, 0.4 MB. Copyright © 2021 Oriol et al. 2021 Oriol et al. https://creativecommons.org/licenses/by/4.0/ This content is distributed under the terms of the Creative Commons Attribution 4.0 International license.

The SarA ChIP peaks on genes have been classified according to their putative functions (Table S4). The three sRNAs whose expression is known to be regulated by SarA (*RNAIII*, *srn_3610_sprC*, and *srn_9340*) have ChIP peaks at their promoter regions, with PSSs of 32.7, 49.9, and 54.8, respectively (Fig. 2A). This study also independently validated SarA binding to the promoters of known SarA mRNA targets like staphylococcal nuclease (SAOUHSC_00818; PSS 42.7), superoxide dismutase (encoded by *sodM*; SAOUHSC_00093; PSS 43.1), thioredoxin reductase (SAOUHSC_00785; PSS 26.4), and the pore-former alpha-hemolysin (SAOUHSC_01121: PSS 43.3) (Fig. 2B) (12, 26). Most of the targets can be divided into 3 classes of proteins: (i) transport/binding and protein fate, (ii) regulatory proteins, and (iii) proteins involved in virulence. Among novel potential SarA targets, there is the transcriptional regulator *mgrA* (SAOUHSC_00694), for which a ChIP peak (PSS, 46.6) is present in its promoter region (Table S3 and Fig. S2). Along with *sarA* (PSS, 37.8), *sarS* (PSS, 47.4), *sarT* (PSS, 34.7), *sarU* (PSS, 34.7), *sarX* (PSS, 35.9), *sarZ* (PSS, 38), *sarY* (PSS, 43.25), and *rot* (PSS, 52.3), our data show that 9 of 10 SarA family members possess a SarA ChIP binding peak in their promoter regions (Table S4). Although some of these *sarA* protein family genes (e.g., *sarA*, *sarS*, *sarT*, *sarU*, and *rot*) have been shown to be directly regulated by SarA, the presence of SarA ChIP peak in the upstream promoter region of other *sarA* paralogs may suggest a differential regulatory mechanism with either an environmental signal or involvement of other factors, which are yet to be elucidated. This result is in accordance with previous data showing cross regulations among SarA family proteins and also proposes some new potential regulations (5).

10.1128/mSystems.00713-21.8 TABLE S4 Classification of SarA-bound targets (ChIP-Seq peak with a *P* value of 1 × 10^−250^ or less) according to their function. Download Table S4, DOCX file, 0.03 MB. Copyright © 2021 Oriol et al. 2021 Oriol et al. https://creativecommons.org/licenses/by/4.0/ This content is distributed under the terms of the Creative Commons Attribution 4.0 International license.

### New sRNA and mRNA genes directly regulated by SarA: identification and validation of 12 SarA novel targets.

In order to differentiate between direct and indirect targets, the intersection between RNA-Seq (Table S2) and ChIP-Seq (Table S3) was used to identify a set of shared gene targets. These genes are likely to be direct targets, since both binding to the promoter region and transcriptional regulation co-occurred. Based on this criterion, 88 mRNA promoters and 15 sRNA promoters predicted to be commonly regulated by SarA were identified (Table S5). Nine mRNAs and three sRNAs genes were selected for further analysis and validation from this set of novel gene targets (Table S5, asterisks; Table 2). We deliberately chose SarA-repressed targets because transcriptional repression seemed to occur preferentially on the promoter region close to the −10 and −35 boxes, whereas transcriptional activation is a more variable process, with the binding site being more difficult to ascertain with EMSA.

**TABLE 2:** mRNA and sRNA targets examined for SarA regulation and binding

Gene group and annotation	Gene name	Assignment	Differential expression in HG003 Δ*sarA* vs HG003 at growth phase				ChIP-Seq data	
			Exponential (2 h)		Early stationary (4.5 h)		Distance to the ATG or +1 (nt)^*a*^	*P* value
			Fold change	*P* value	Fold change	*P* value		
mRNA genes repressed by SarA								
SAOUHSC_00088	*galE*	Hypothetical protein	75.51	3.27E−239	43.02	2.25E−71	85	7.09E−242
SAOUHSC_00555		Haloacid dehalogenase-like hydrolase	15.19	4.18E−136	7.04	2.12E−23	33	0
SAOUHSC_00913	*lysR*	Hypothetical protein	65.05	5.18E−208	42.81	2.02E−71	58	0
SAOUHSC_00961	*comK1*	Hypothetical protein	15.15	3.81E−60	9.37	2.53E−27	111	0
SAOUHSC_00975		Hypothetical protein	4.74	4.74E−53	26.62	1.94E−57	440	0
SAOUHSC_01452	*ald1*	Alanine dehydrogenase	16.71	5.29E−147	38.31	9.21E−64	197	0
SAOUHSC_02127	*scpA*	Staphopain thiol proteinase	126.67	0.00E + 00	57.24	2.25E−71	221	0
SAOUHSC_02696	*fmhA*	Methicillin resistance determinant	38.92	1.01E−198	20.99	2.98E−51	99	0
SAOUHSC_02820		Hypothetical protein	23.37	1.62E−165	44.41	1.11E−70	82	0

sRNA genes repressed by SarA								
* srn_3950_teg16*	Transcript	Unknown			21.83	4.41E−44	106	0
* srn_0860_rsaOB*	Transcript	Unknown			10.51	7.34E−29	88	1.09E−135
* srn_9335_tsr29*	Transcript	Unknown	3.09	9.40E−09	5.45	5.57E−15	113	0

a Distance to the ATG for mRNA genes; distance to the +1 position for sRNA genes.

10.1128/mSystems.00713-21.9 TABLE S5 Complete ChIP-Seq/RNA-Seq shared genes (SarA direct targets). Where operon organization is available, it appears with a grey background, with the first gene of the operon marked in bold. Asterisks highlight genes selected for experimental investigation of SarA direct regulation. SarA-activated genes are in green; SarA-repressed genes are in red. Download Table S5, DOCX file, 0.03 MB. Copyright © 2021 Oriol et al. 2021 Oriol et al. https://creativecommons.org/licenses/by/4.0/ This content is distributed under the terms of the Creative Commons Attribution 4.0 International license.

Our selection was based on (i) high repression level inferred from the RNA-Seq data, (ii) the presence of a ChIP-Seq peak with a *P* value of <10^−250^, and (iii) a SarA ChIP peak location compatible with transcriptional repression. Thus, these genes could serve as a stringent set from which the binding of SarA to gene promoters and downstream analysis could be examined. All 12 genes had not been identified previously as SarA direct targets, except for the staphopain thiol protease (SAOUHSC_02127) (70, 71). In addition to these, a LysR-like putative regulatory protein (SAOUHSC_00913) appeared to be repressed by SarA in a previous transcriptomic experiment (50), but direct regulation between *sarA* and *lysR* had not been demonstrated until now. For each candidate, the overall RNA-Seq and ChIP-Seq data were summarized using Integrative Genomic Viewer (Fig. S3) (72).

10.1128/mSystems.00713-21.3 FIG S3 IGV visualizations of potential SarA targets revealed through ChIP-Seq and RNA-Seq. Download FIG S3, PDF file, 0.4 MB. Copyright © 2021 Oriol et al. 2021 Oriol et al. https://creativecommons.org/licenses/by/4.0/ This content is distributed under the terms of the Creative Commons Attribution 4.0 International license.

**FIG 3:**
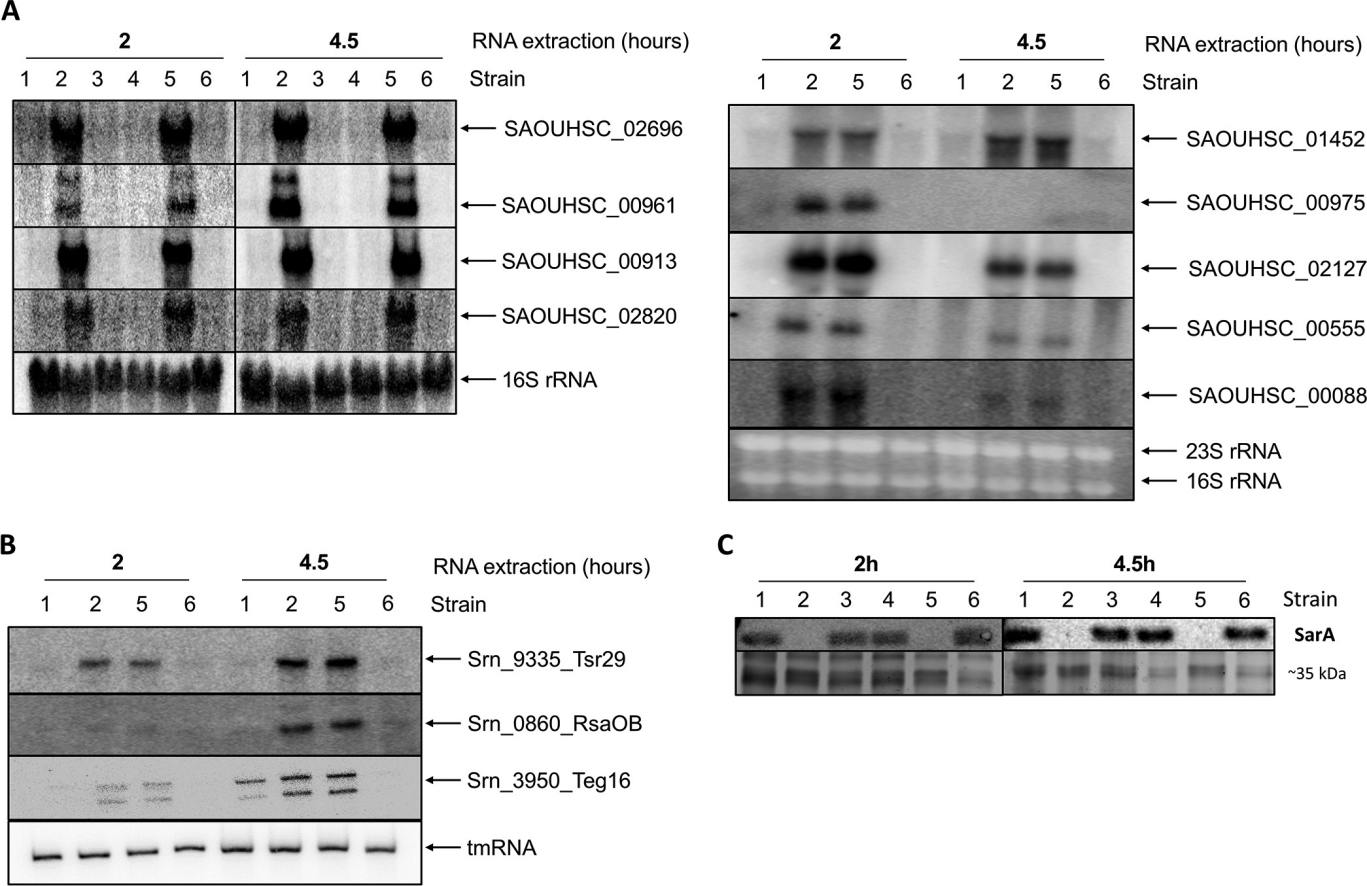
SarA represses new mRNA and sRNA targets. Expression levels of SarA mRNA targets (A) and sRNA targets (B) were monitored by Northern blotting. All samples were harvested after 2 h and 4.5 h of growth. A total of 10 or 15 μg of total RNA was extracted from different S. aureus strains and analyzed using radioactive specific PCR-product probes. tmRNA, 16S, or 16S + 23S RNA was used as a loading control (Table S1B). (C) Western blot showing the SarA protein levels using anti-SarA antibodies (top). Deposit was controlled with Coomassie staining (bottom image). Lanes (all panels): 1, HG003 WT; 2, HG003 Δ*sarA*; 3, HG003 + pCN36; 4, HG003 + pCN36-*sarA*; 5, HG003 Δ*sarA* + pCN36; 6, HG003 Δ*sarA* + pCN36-*sarA* for *sarA* complementation.

To test the regulation of these 12 targets by SarA, we isolated total cellular RNA from various HG003-derivative strains in two phases of growth. Northern blot assays were performed on either agarose or acrylamide gels, depending on the size of the analyzed mRNAs and sRNAs. All mRNAs and sRNAs selected based on stringent criteria showed increased expression based on band intensity in the absence of *sarA*, which confirms a repressive role of SarA on the expression of these targets (Fig. 3). We observed high level of transcripts in the absence of *sarA*, while these expression levels were restored to the wild-type level in the parent/complemented strains at both phases of growth (Fig. 3A and B). These Northern blot data confirmed our RNA-Seq results, whereas Western blotting performed in parallel (Fig. 3C) confirmed the presence or the absence of SarA.

**FIG 4:**
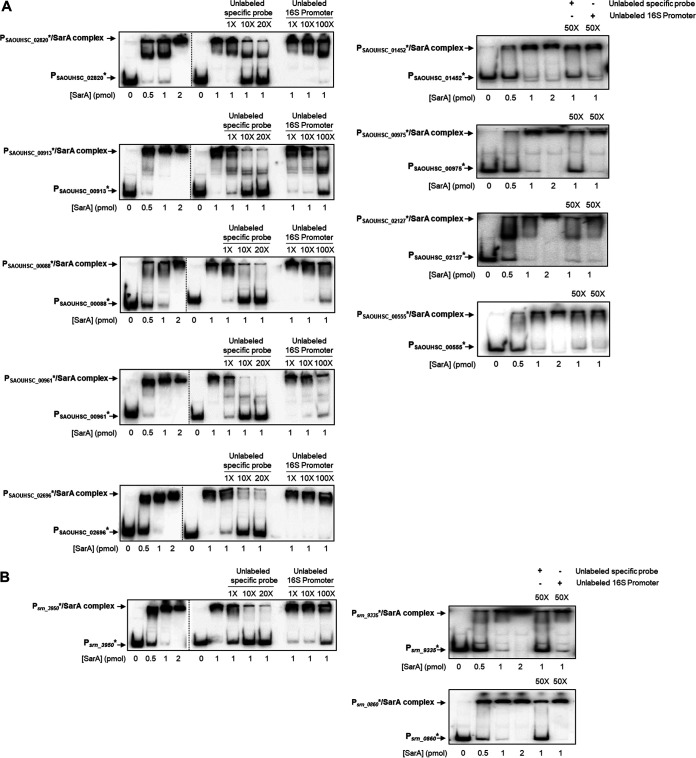
SarA specifically binds to the promoter region of all the selected targets *in vitro*. (A) SarA specifically binds to the 9 mRNAs promoter regions tested. (B) SarA specifically binds to the 3 sRNAs promoter regions tested. Electrophoretic mobility shift assays (EMSAs) were done using 10 fmol of each probe, a ^32^P-labeled promoter fragment of ∼250 bp, and increasing amounts of 0 to 2 pmol 6His-tagged SarA. Specificity of SarA binding was assessed with 1 pmol of SarA and increasing amounts of specific (unlabeled promoter tested) or nonspecific competitors (unlabeled 16S promoter). The promoter/SarA complex is inhibited only in the presence of excesses of the specific competitor.

The ChIP-Seq data suggested that SarA should bind the promoter regions of the 12 mRNAs and sRNAs to downregulate expression. To confirm the direct binding of SarA to the upstream promoter region of these targets, we performed EMSAs as shown in Fig. 4. For this purpose, a 250-bp region from −200 bp upstream to +50 bp downstream from the transcription start site (TSS) of all 12 targets were PCR amplified, radiolabeled, and used to perform EMSA with purified recombinant His_6_-SarA protein. The transcription start site (TSS) was determined by using TSS EMOTE (73). The addition of increasing amounts of SarA (from 0 to 2 pmol) led to progressive retardation of all selected DNA fragments (Fig. 4), suggesting that SarA binds each of the 12 promoter regions tested *in vitro*. To determine if SarA binding is specific, EMSA was also performed with 1 pmol of SarA and an excess of cold specific (promoter of interest) or nonspecific (16S) upstream promoter DNA fragments, demonstrating that the cold specific probe disrupts the labeled SarA-DNA complex, whereas the addition of excess nonspecific fragment cannot outcompete binding with SarA. Taken together, Northern blotting and EMSA results clearly indicated that the hypothesis that common RNA-Seq and ChIP-Seq targets are direct SarA targets is correct.

### SarA represses transcription of three novel sRNAs with imperfect selection criteria.

There were three sRNAs (*srn_4540_sprA2_AS_*, *srn_1640_rsaD*, and *srn_0455_tsr9*) that did not reach both the RNA-Seq and ChIP-Seq criteria for potential SarA direct targets. These were included in downstream studies for different reasons.

**FIG 5:**
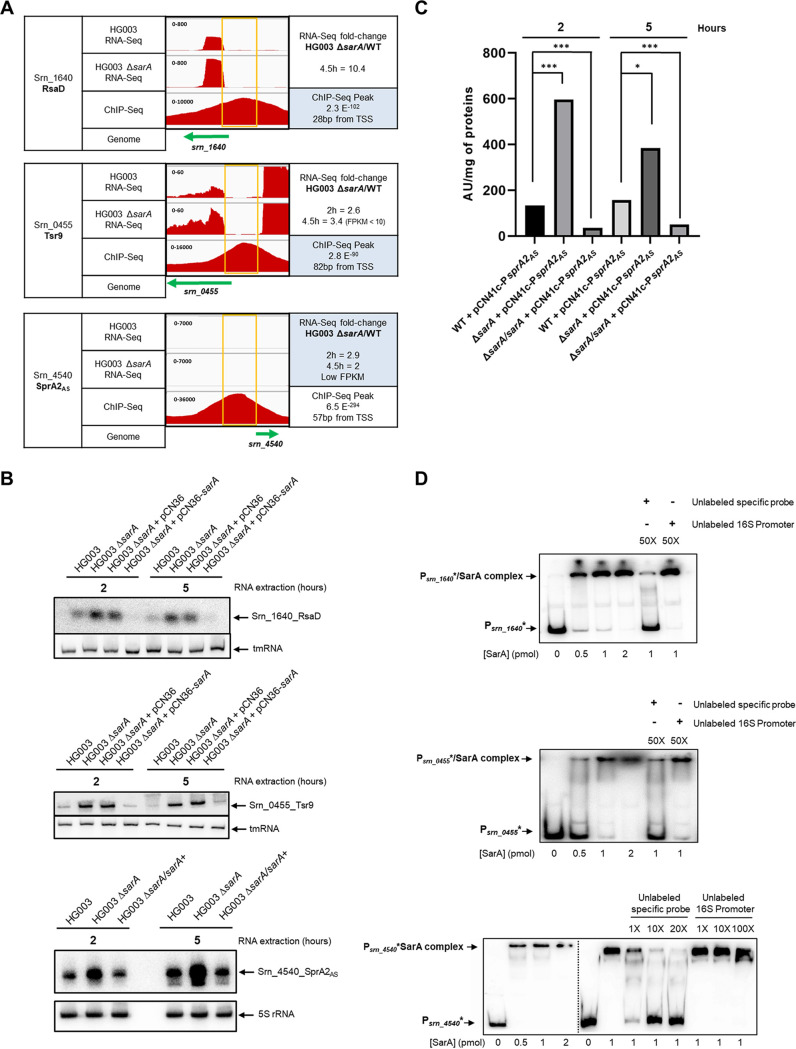
SarA represses 3 new sRNA. (A) IGV visualization of RNA-Seq profiles (HG003 and HG003 Δ*sarA*) and SarA ChIP-Seq profiles of RsaD, Tsr9, and SprA2_AS_. sRNA genes are indicated with green arrows, and the yellow box represents the ChIP-Seq peak region upstream of the sRNA gene repressed by SarA. The RNA-Seq fold change is indicated on the right, with the distance of the ChIP peak from the transcription start site (+1) (TSS). (B) sRNA targets expression in HG003 WT and in isogenic mutant for *sarA*. Northern blots used RNA extracted from WT S. aureus HG003, HG003 Δ*sarA*, HG003 Δ*sarA* + pCN36, and HG003 Δ*sarA* + pCN36-*sarA* or HG003 Δ*sarA/sarA*^+^ genomic expression. All samples were harvested after 2 h and 5 h of growth. Ten micrograms of total RNA was analyzed and revealed using radioactive probes, and tmRNA or 5S RNA was used as a loading control (same membrane revealed on a different day). (C) SarA effects on transcriptional activity of the *srn_4540_sprA2_AS_* promoter (P*sprA2_AS_*). HG003, HG003 Δ*sarA*, and a *sarA*-complemented strain (Δ*sarA/sarA*^+^) were transformed with pCN41c or pCN41c-P*sprA2_AS_*. P*sprA2_AS_* activity was estimated by measuring β-lactamase substrate hydrolysis. For each lane, the indicated β-lactamase activity was normalized by subtracting the background signal from the same strain where pCN41c-P*sprA2_AS_* was replaced by a pCN41c empty vector (not shown). Three independent experiments were done; error bars show standard deviations. *, *P* < 0.05; ***, *P* < 0.001 (Mann-Whitney test). (D) SarA specifically binds *in vitro* the promoter region of the 3 sRNA targets. SarA binds the promoter region of *srn_1640_rsaD*, *srn_0455_tsr9*, and *srn_4540_sprA2_AS_*. EMSAs were done using 10 fmol of each ^32^P-labeled promoter fragment and increasing amounts of 0 to 2 pmol 6His-tagged SarA. Specificity of the binding was assessed with the use of unlabeled *srn_1640_rsaD*, *srn_0455_tsr9*, and *srn_4540_sprA2_AS_*.promoters, and a 16S RNA promoter was used as the control for the competitive assay.

For Srn_4540_SprA2_AS_, an RNA antitoxin from a type I toxin-antitoxin (TA) system, there was a well-defined SarA ChIP peak 67 nucleotides (nt) upstream (PSS 36.6) from the +1 TSS with a *P* value of 6.9 × 10^−294^ (Fig. 5A and Table S3), but the 62-nt SprA2_AS_ sRNA was not detected in the RNA-Seq experiment (74). This is likely because of its small size, which would allow it to slip past the analysis.

For Srn_1640_RsaD, a stress‐responsive riboregulator of overflow metabolism (37), the threshold values were barely under our stringent cutoffs: RNA-Seq data show repression by SarA at the early stationary growth phase (Table S2), but the ChIP peak present at its promoter had a *P* value of only 2.1 × 10^−102^ (Fig. 5A).

For Srn_0455_Tsr9, an sRNA with a potential ORF but no identified peptide (53, 75), RNA-Seq data show repression by SarA, but the SarA ChIP-Seq peak on its promoter had a *P* value of only 2.8 × 10^−90^ (Fig. 5A; Table S3). We considered that the threshold for ChIP peak *P* values could have been adjusted to include sRNAs like these, because due to the promiscuous nature of SarA for AT rich sequences, we chose very stringent criteria for the analysis of the intersection of RNA-Seq with ChIP-Seq data.

Northern blot analyses with RNA extracted from HG003 and HG003 Δ*sarA* were performed at E and S growth phases. The expression levels of *srn_4540_sprA2_AS_*, *srn_1640_rsaD*, and *srn_0455_tsr9* were all higher in the *sarA* mutant and returned to wild-type levels upon complementation, confirming SarA as a repressor in these cases (Fig. 5B).

We hypothesized that this regulation occurs due to SarA binding at their respective promoters. To test this hypothesis, we used a β-lactamase reporter assay for the *srn_4540_sprA2_AS_* promoter (P*sprA2_AS_*) using strains expressing a pCN41-P*sprA2_AS_-blaZ* fusion. When wild-type HG003 and HG003 Δ*sarA* were compared for *srn_4540_sprA2_AS_*, there were 4-fold and 2.5-fold increases in β-lactamase activity at 2 and 5 h of growth, respectively, and β-lactamase activity levels were similar to that of the parental strain in a complemented HG003 Δ*sarA* strain with the *sarA* gene introduced onto the chromosome via the *geh* locus (Fig. 5C).

When the binding of SarA to the P*rsaD*, P*tsr9*, and P*sprA2_AS_*, promoters was examined by EMSA, SarA specifically bound the promoters of *srn_1640_rsaD*, *srn_0455_tsr9*, and *srn_4540_sprA2_AS_*, (Fig. 5D). Only a fragment of nonradioactive (cold) specific promoter was able to compete with its respective promoter, thereby demonstrating the specificity of the interaction.

All these results confirmed that SarA negatively regulates *srn_4540_sprA2_AS_*, *srn_1640_rsaD*, and *srn_0455_tsr9* expression at the transcriptional level, with SarA acting on respective promoters to reduce RNA expression. With these three examples, we show that the use of strict criteria for selecting direct targets could, at least for sRNA, be adapted case by case.

### SarA-driven repression of a type I toxin inhibits S. aureus toxicity.

Among the sRNAs identified as SarA potential targets is s*rn_2230_sprG2*, which encodes a toxin from the type I TA system SprG2/SprF2 (Table S5) (76). In this TA system, SprF2 is the antisense RNA antitoxin and regulates toxin production, under normal growth conditions, by binding to SprG2 mRNA and degrading it before it is translated.

**FIG 6:**
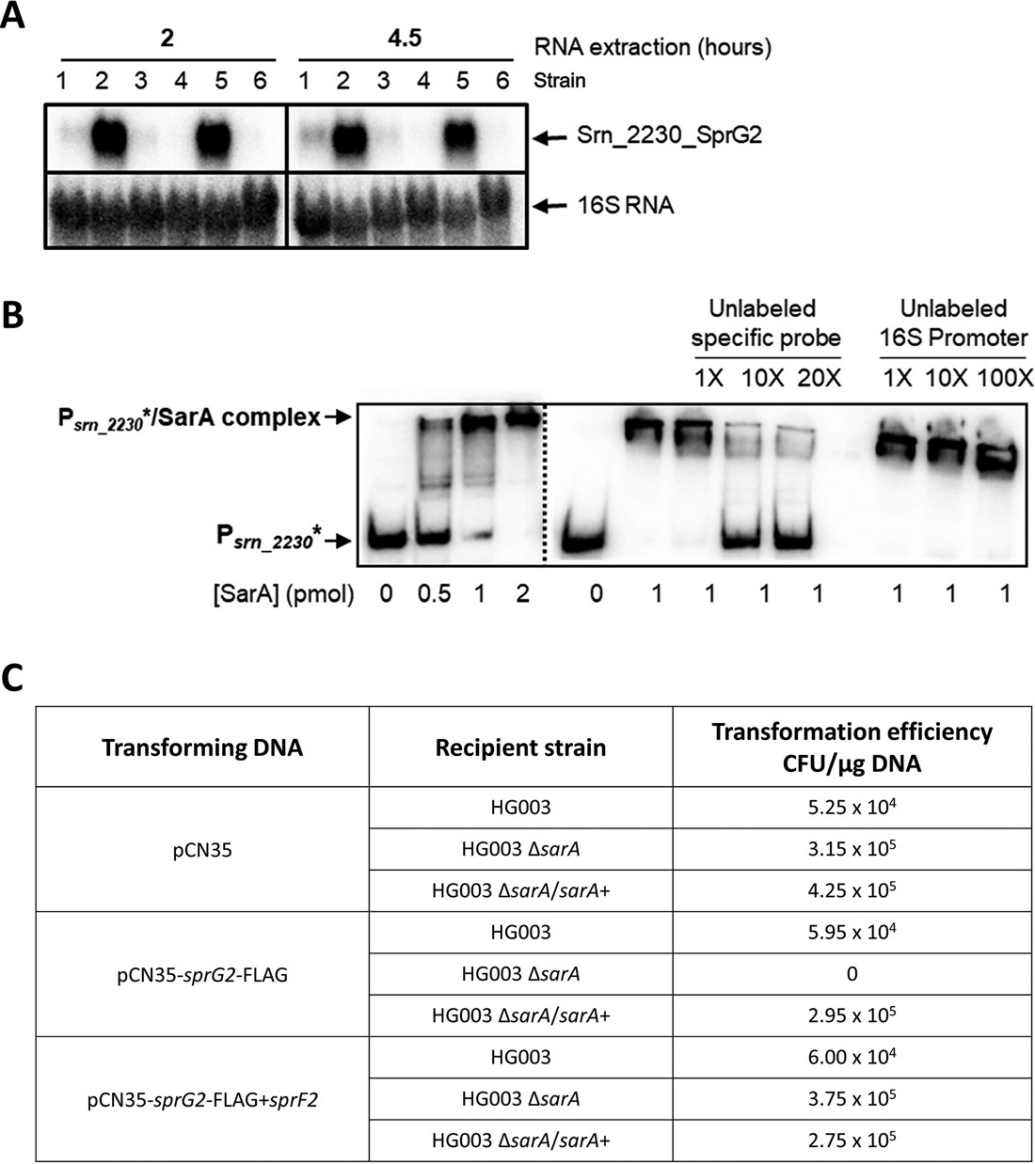
SarA involvement in the type I toxin-antitoxin system. (A) *srn_2230_sprG2* expression in HG003 WT and its isogenic mutant for *sarA*. Northern blots of RNA extracted from different S. aureus strains. Lanes: 1, HG003 WT; 2, HG003 Δ*sarA*; 3, HG003 with pCN36; 4, HG003 with pCN36-*sarA*; 5, HG003 Δ*sarA* with pCN36; 6, HG003 Δ*sarA* with pCN36-*sarA* for *sarA* complementation. All samples were harvested after 2 h and 4.5 h of growth. A total of 15 μg of total RNA was analyzed and revealed using a radioactive specific PCR-product probe and 16S RNA as a control. (B) SarA binds the *srn_2230_sprG2* promoter region. Specificity of the binding was assessed with the use of unlabeled *srn_2230_sprG2* promoter, and a 16S RNA promoter was used as control for the competitive assay. (C) Results of transformation efficiency with pCN35, pCN35-*sprG2*Flag, and pCN35-*sprG2*Flag+*sprF2* in HG003, HG003 Δ*sarA*, and HG003 Δ*sarA/sarA*^+^ for *sarA* complementation. It was impossible to obtain HG003 *sarA* clones with pCN35-*sprG2*Flag.

A Northern blot experiment (Fig. 6A) clearly showed that *srn_2230_sprG2* expression was increased in the absence of *sarA*. We performed EMSA studies to verify the direct regulation of SarA on *srn_2230_sprG2* expression, showing that SarA directly and specifically binds the *srn_2230_sprG2* promoter (Fig. 6B).

In the HG003 Δ*sarA* strain, Srn_2230_SprG2 mRNA toxin is expressed at higher levels than in isogenic HG003 (Fig. 6A). Thus, we wanted to see if this was followed by a high level of peptide production. To test our hypothesis, HG003, HG003 Δ*sarA*, and HG003 Δ*sarA/sarA^+^* were transformed by pCN35, pCN35_*sprG2*Flag, and pCN35_*sprG2*Flag *sprF2*. In a *sarA* deletion strain, we were unable to introduce the pCN35_*sprG2*Flag in the absence of *sprF2*, whereas it was possible to express *sprG2Flag+sprF2* in the same strain (Fig. 6C). The fact that we did not obtain any clones suggests that vector overexpression of SprG2-Flag RNA without any SarA and/or SprF2 regulation is toxic for S. aureus. The latter result led us to think that SarA could, in part, regulate the SprG2/SprF2 TA system via a repression of SprG2 toxin expression in order to prevent toxicity.

### Scanning the S. aureus genome for novel regulatory targets with a gapped “direct target” motif.

We used the GLAM2 tool from the MEME suite v5.3.0 to see if we could generate a predictive motif for SarA binding to the upstream promoter region of sRNAs. We hypothesized that a sequence-based bioinformatic search would be less biased by environmental or accessory regulatory interactions that would impact *in vivo* experiments like RNA-Seq and ChIP-Seq.

We initially used promoter sequence of different categories of SarA-repressed targets, including both sRNAs and mRNAs with promoter regions ranging from 100 to 200 bp. In preliminary tests, we saw that the use of a short promoter region (100 bp) starting before the TSS +1 in the data set for SarA direct regulation had the best chance at generating a good motif. We also elected to work with sRNA targets for which a +1 TSS was described and validated and which were identified as direct targets for SarA using the combined RNA-Seq and ChIP-Seq analysis. We did not imply a constraint that the motif be dyad symmetric on the search. Using the promoters of 15 selected sRNAs which are expected or proven to be SarA targets (Table S6), we used GLAM2 to generate a SarA position weight matrix (PWM) (Fig. S2A). The GLAM2-derived SarA PWM based on *in vivo* association is similar, but not identical, to the consensus binding operator sequence described by biochemical and genetic analysis (17, 77).

10.1128/mSystems.00713-21.10 TABLE S6 (A) GLAM2Scan analysis showing the 200 first S. aureus genome hits (GLAM2Scan results and their ties to possible regulatory targets for SarA [addition of RNA-Seq and ChIP-seq data when available]). The 15 sRNA promoter sequences used to generate the GLAM2 consensus are presented at the bottom. (B) Classification of SarA potential targets revealed by GLAM2Scan into 7 groups. Download Table S6, DOCX file, 0.2 MB. Copyright © 2021 Oriol et al. 2021 Oriol et al. https://creativecommons.org/licenses/by/4.0/ This content is distributed under the terms of the Creative Commons Attribution 4.0 International license.

In addition, numerous previously known SarA targets contain this PWM, indicating that the GLAM2-derived SarA PWM could be a true SarA binding motif. Interestingly, both DNase I footprinting mapped SarA binding operator (77) and GLAM2-derived SarA PWM consist of AT-rich sequences, and potential canonical SarA sequence recognization motif ATTTTAT (19) or ATTTAA (27, 78) is also detected in both analyses with some variations. The SarA binding operator (ATTTGT**ATTTAA**T**ATTTAT**ATAATTG) contains at least two potential recognition motifs (in bold), whereas the deduced 35-bp SarA PWM contains more than two similar motif sequences. Although the AT-rich motif generated by GLAM2 (Fig. S2A) does not exactly match any individual promoter or SarA binding site, it has some similarity to all of them and can be matched to well-established sites with tandem repeats and palindromic SarA binding sites like that found in SarA binding to the *agr* locus (27). Overall, the earlier-mapped SarA binding operator and GLAM2-derived SarA PWM are not exactly the same but have close similarity, as both contain AT rich sequences.

Using the best motif found with the standard number of iterations in GLAM2 (Fig. S4A), the position weight matrix was used to scan the whole S. aureus genome using GLAM2Scan (79). The first 200 matches were used to locate putative genomic SarA binding sites and their cognate genes (Table S6).

10.1128/mSystems.00713-21.4 FIG S4 (A) SarA PWM motif; (B) GLAM2Scan results compared with those obtained by footprinting experiments for *srn_3610_sprC* and *srn_9340* (28). (C) Analysis of 3 promoter sequences obtained after GLAM2Scan search (hits 16 to 18). Download FIG S4, TIF file, 2.5 MB. Copyright © 2021 Oriol et al. 2021 Oriol et al. https://creativecommons.org/licenses/by/4.0/ This content is distributed under the terms of the Creative Commons Attribution 4.0 International license.

Before categorizing the first 200 hits, we completed our bioinformatics analysis by comparing the sequence consensus obtained for *srn-3610_sprC* and *srn_9340* with the SarA protected sequence obtained by DNA footprints (28). In both of these sRNAs, the protected sequence is part of the predicted SarA consensus target site (Fig. S4B). This further validates that the consensus allows us to discern actual regulatory sites for SarA in the genome.

Moreover, putative SarA binding sequences obtained after GLAM2Scan analysis, hits 16 to 18, which correspond to unknown SarA targets (Fig. S4C), were screened and revealed tandem repeats and palindromic sequences (hits 17 and 18).

Then, we compared these 200 hits with already known SarA targets and newly identified SarA targets from this study (RNA-Seq and/or ChIP-Seq data), which allowed us to categorize targets into several groups (Table S6B).

The first group is composed of 14 targets described in the literature. This group includes SAOUHSC_02862 (*cplL*) and SAOUHSC_03001 (*icaR*) (26, 80). The second and third groups contain 43 and 38 targets found in the ChIP-Seq or RNA-Seq data, respectively. The ChIP-Seq-only group included *sarZ* and *opp4A* (SAOUHSC_00928), and the RNA-Seq group included *gltB* (SAOUHSC_00435), *gltT* (SAOUHSC_02667). The fourth group contains 24 targets that were found in both the RNA-Seq and ChIP-Seq results and therefore contains targets that are listed in Table S5. This group includes SAOUHSC_02696 (*fmhA*), SAOUHSC_00975, and SAOUHSC_01452 (*ald1*). All three targets were proved experimentally to be directly regulated by SarA. The fifth group consists of 11 targets found in literature and in at least one experimental result (RNA-Seq or ChIP-Seq). This group includes *hla* (SAOUHSC_01121), *sak* (SAOUHSC_02171), and *sarS* (SAOUHSC_00070) (51, 59, 60, 77). The sixth group correspond to 10 hits but only 5 targets that appeared in the RNA-Seq, ChIP-Seq, and literature data. This group includes *nuc* (SAOUHSC_00818), which is known to be regulated by SarA (50).

The seventh group is composed of 51 newly identified targets for which a SarA binding motif was discerned in their promoter regions. In this group, there are 45 mRNA genes and 6 sRNA genes potentially directly regulated by SarA. This group includes *srn_5010_ teg33*, *gltC* (SAOUHSC_00434), and *argF* (SAOUHSC_01128). In particular, *argF* as a SarA target resonates with a known SarA involvement in arginine biosynthesis/metabolism described in the literature, where RNA-Seq results showed that *argB*, -*C*, -*D*, -*G*, -*H*, -*J*, and -*R* and *rocA* and -*D* are apparently regulated by SarA (Table S2) (26, 67). One group is composed of the 15 sRNAs that were used to create the SarA binding motif and are underlined in yellow in Table S6.

Altogether, 60% of the SarA binding targets proposed by GLAM2Scan were already described as SarA direct or indirect targets.

**FIG 7:**
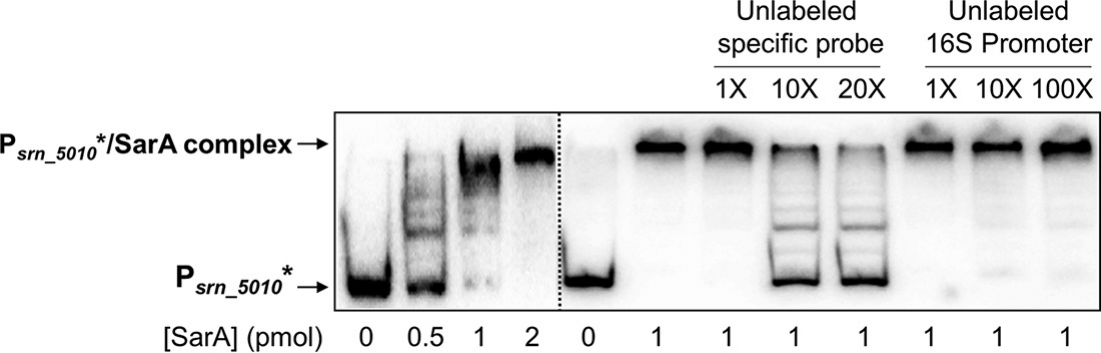
Validation of the *srn_5010_teg33* promoter as a SarA binding target. SarA specifically binds the *srn_5010_teg33* promoter. EMSAs were done using a 265-nt DNA probe of the *srn_5010_teg33* promoter and competed off with excess cold specific *srn_5010_teg33* promoter or a nonspecific 26-nt 16S promoter sequence.

From the set of novel putative regulatory targets of *sarA*, we experimentally challenged the sRNA *srn_5010*_*teg33* using EMSA as a proof of concept (hit 16). A 265-bp DNA probe, containing a 200-bp upstream region from the transcription start site of *srn_5010_teg33* and the first 65 bp of the *srn_5010_teg33* transcript, including the predicted SarA binding site, was PCR amplified. EMSA using purified SarA protein and the *srn_5010_teg33* radiolabeled probe showed that SarA is able to bind specifically to the *srn_5010_teg33* promoter region (Fig. 7). Two picomoles of purified SarA was able to completely retard 10 fmol of radiolabeled DNA fragment, which could be disassociated using cold *srn_5010_teg33* probe but not from a nonspecific 16S DNA fragment, thus demonstrating binding specificity of SarA to the target.

We thus surmise that this bioinformatics GLAM2/GLAM2Scan analysis strengthened the ChIP-Seq and RNA-Seq results and uncovered the potential to discover new SarA targets not revealed experimentally under the growth conditions of the RNA-Seq and ChIP-Seq experiments.

## DISCUSSION

Understanding how SarA participates in virulence, antibiotic resistance, biofilm formation, and environmental adaptation is central to understanding the biology and pathogenicity of S. aureus. To elucidate the comprehensive set of SarA regulation, its direct and indirect target genes should be identified. Here, we present an updated detailed analysis of the SarA regulon in S. aureus using both RNA-Seq and ChIP-Seq analyses focusing on sRNAs as the major SarA regulatory targets. The transcriptional profile by RNA-Seq of HG003 and its isogenic *sarA* deletion strain showed that the expression of 441 genes is impacted in the *sarA* mutant, confirming that SarA is a major regulator of gene expression in S. aureus. Previous microarray analysis indicated that the lack of SarA influences transcription of only ∼120 genes in strain RN27 (26). Our analysis increases the size of the SarA regulon up to four times and includes many sRNAs which could not be captured by microarray techniques.

Among novel targets, we identified the repression of two gene pairs producing leukotoxins by SarA. Therefore, with *hlgC*/*hlgB* (SAOUHSC_02709/SAOUHSC_02710) already described as a SarA activated target by Dunman et al. (26), there are at least three leukotoxins, *hlgC*-*hlgB* (SAOUHSC_02709/SAOUHSC_02710), *lukG*-*lukH* (SAOUHSC_02241_/SAOUHSC_02243), and *lukD*-*lukE* (SAOUHSC_01954/SAOUHSC_01955), that are regulated in the *sarA* mutant in our RNA-Seq experiment. Leukotoxins are, among other things, responsible for the targeted killing of host immune cells (66). Therefore, SarA can and does regulate leukotoxin production through its binding or release from leukotoxin promoter region. These results, in combination with ChIP-Seq analysis where a ChIP peak has been localized in the upstream regions of the 3 gene pairs producing leukotoxins (Table S3), emphasize the notion that SarA leukotoxin regulation is direct.

ChIP-Seq experiments have identified 341 DNA sequences bound to SarA, with *P* values under 10^−250^ (Table S3). The SarA-bound loci on the genome (Fig. 2D and Table S3) can be divided into two overarching subclasses. In the first subclass, the majority of the hits were peaks in the promoter regions of genes. This category includes 32 known targets that were independently validated in previous studies (6, 13, 15, 16, 18, 22, 25–28, 46, 49–52). It also included 325 mRNA and 52 sRNA promoters not previously reported as regulated by SarA; some of them have been proven by us to be SarA direct targets. The second category includes 34 hits localized in intergenic regions or situated in the middle of genes, perhaps lending credence to the hypothesis that SarA has an additional role of mediating architectural functions on the bacterial chromosome (11).

**FIG 8:**
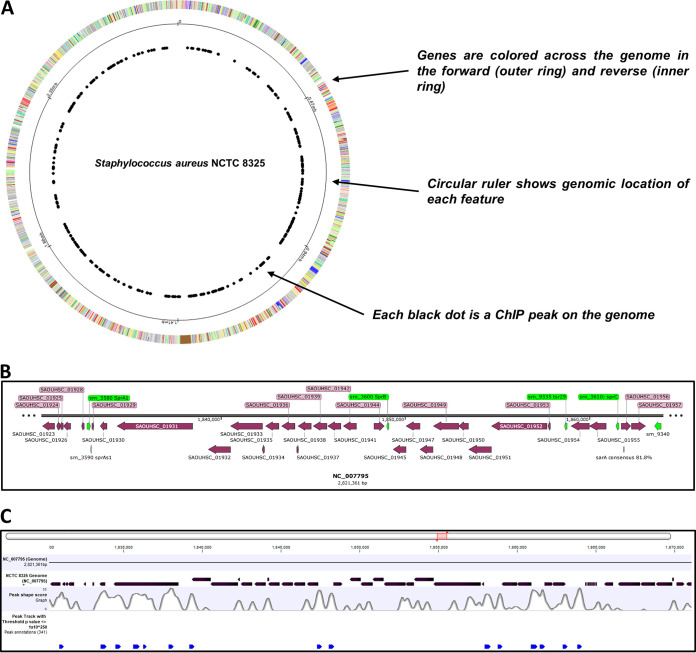
SarA binding locations on the vSAβ pathogenicity island in HG003. (A) Location of SarA binding sites by ChIP-Seq across the S. aureus chromosome. (B) Location of the vSAβ pathogenicity island in the NC_007795 chromosome (location, 1830265 to 1863962) with coding sequences (CDS) and sRNA annotations in SnapGene. (C) The vSAβ pathogenicity island rendered in CLC Genomic Workbench with tracks showing its location on the NC_007795 genome, genomic annotations, peak shape score from ChIP mapping, and annotated peaks with a threshold of 1E−250 (blue arrowheads).

Instead of being distributed evenly across the bacterial chromosome, ChIP-Seq peaks tended to cluster in certain regions (Fig. 8A). With a SarA binding event ∼3.5 times higher in pathogenicity island νSAβ (roughly 33.7 kb long, located in NC_007795 at positions 1830265 to 1863962 and 15 SarA ChIP peaks) than for the whole staphylococcal genome (2.82 Mb and 341 SarA ChIP peaks) (Fig. 8B and C), the νSAβ PI region appears as a privileged SarA binding zone. We observed that the promoter regions of 3 sRNA genes (*srn_3610_sprC*, *srn_9340*, *srn_9335*) and 5 mRNA genes (SAOUHSC_01923, SAOUHSC_01942, SAOUHSC_01944, SAOUHSC_01955, and SAOUHSC_01956) are directly bound by SarA, with up to 16 transcripts potentially under the control of the SarA regulation. Although the νSAβ PI AT content is higher than that of the whole genome (71% versus 67.3%, respectively) and SarA is known to bind AT-rich regions, such an AT content difference cannot explain the SarA binding increase. AT content variation likely impacts the DNA topology, and the higher SarA ChIP peaks events in that region could be in accordance with a possible architectural role of SarA in DNA binding (11).

Thirteen novel mRNA and sRNA gene targets of SarA were selected for validation and proven to be directly regulated by SarA, showing the merit of this multiomics approach to finding direct regulatory targets for Staphylococcus regulators for both mRNA and sRNA targets (Fig. 3, 4, and 7). Apart from this common target gene listing, we studied sRNAs *rsaD*, s*prA2_AS_*, and *tsr9* and proved a direct regulation by SarA. Altogether, the expression of 10 sRNAs is directly regulated by SarA (Fig. 3 to 7), which corresponds to a fifth of sRNAs revealed as SarA targets in our RNA-Seq or ChIP-Seq studies (Tables S3 and S5; Fig. S4).

Interestingly, among the novel targets of SarA is SprG2 RNA toxin from a type I toxin-antitoxin (TA) system SprG2/SprF2. *srn_2230_sprG2*, whose expression have been shown to be repressed by SarA (Fig. 6), encodes a membrane peptide whose overexpression triggers bacteriostasis (76). Overexpression of *srn_2230_sprG2* could therefore occur in the bacterial cell when SarA detaches from the *sprG2* promoter or when *sarA* expression is lowered. Expression of *sarA* mRNA is reduced in thymidine-auxotrophic small-colony variants (SCVs) and in SCVs of methicillin-resistant S. aureus (MRSA), with the possibility that *srn_2230_sprG2* is overexpressed in these cells (81–83). SCVs are associated with chronic and recurrent infections and *in vitro* with bacteria called persisters (84). These persister cells are a subpopulation of genetically identical and metabolically slow-growing bacteria that exhibits a multidrug-tolerant phenotype (85). Type I TA systems have been linked to persister cell formation (86–90). SarA-dependent *srn_2230_sprG2* overexpression may be another trigger that facilitates SCV and persister cells formation in S. aureus and should be examined in detail in further studies. A possible hypothesis on the role of SarA on SprG2/SprF2 TA systems is that SarA prevents toxin expression during E phase but allows their toxic activity during the S growth phase, when cell death or stasis is beneficial to the cell. Accordingly, Riffaud et al. showed that *srn_2230_sprG2* expression is higher in S phase than E phase (76).

Our study also reveals the implication of SarA in a second type I TA pair, the SprA2/SprA2_AS_ TA system. *srn_4550_sprA2* encodes PepA2, a toxic peptide which internally triggers bacterial death and is highly toxic to host cells (74). In this TA system regulated by SarA, the absence of *sarA* is responsible of an increase in antisense RNA antitoxin SprA2_AS_ (Fig. 5B and C). In this case, increased SarA binding on the *srn_4540_sprA2_AS_* promoter could be responsible for lower *sprA2_AS_* expression, whereas SarA release from the *srn_4540_sprA2_AS_* promoter could lead to an increase in the amount of Srn_4540_SprA2_AS_ RNA. Such a dual function for a type I RNA antitoxin has been described for SprF1, which is able to repress SprG1 toxin translation and to bind ribosomes to induce global translation inhibition, promoting persister cell formation (90).

By exploring novel SarA regulation targets, this work also identified Srn_1640_RsaD, which acts on carbon overflow metabolism through translation repression of the acetolactate synthase. Acetolactate synthase prevents intracellular acidification and participates in macrophage infection, antibiotic resistance, and biofilm formation (61, 91). These phenotypes fit well with the *sarA* mutant strain phenotype. Moreover, in a recent publication, *srn_1640_rsaD* expression was shown to be positively regulated by the SsrAB two-component system (TCS). Also, like *ssrAB*, *srn_1640_rsaD* expression is increased under NO stress (92). As *srn_1640_rsaD* is repressed by SarA and as SarA DNA binding abilities are sensitive to oxidative stress, the *srn_1640_rsaD* overexpression observed under NO stress could reflect SarA release from the *srn_1640_rsaD* promoter due to SarA posttranslational modifications.

Besides SarA regulation, Srn_1640_RsaD is also directly regulated by CodY, a second TF activated by branched-chain amino acids (37). A comparison between CodY and SarA sRNA regulons indicated that 11 of the 37 sRNAs repressed by SarA are also repressed by CodY, while only one of the 14 sRNAs activated by SarA is also activated by CodY (37). The number of sRNAs regulated by the two TFs is substantial, and the possible regulation of CodY by SarA should be investigated further in the future. On the other hand, genes under the control of several transcriptional factors are commonplace, and there have been links showing SarA and CodY coregulation in the quorum-sensing operon *agrBDCA* (93).

A SarA binding motif was generated using GLAM2 from the MEME suite using 15 sRNA SarA direct targets. Using this SarA binding motif, we arrived at 200 first hits on the genome which were classified as SarA targets based on literature and/or in our experimental results. By making this ranking, we realized that 60% of the hits corresponded to previously described SarA targets, validating our RNA-Seq and ChIP-Seq data. That ranking also revealed a set of 51 potential new SarA targets (Table S6B, group 7). Then, we wondered if the presence of a SarA binding motif in a promoter region leads to SarA binding. For this purpose, we selected the sRNA gene *srn_5010_teg33* and proved that SarA binds specifically on its promoter (Fig. 7).

Whereas RNA-Seq and ChIP-Seq give a picture of the regulation at one moment or under specific conditions, bioinformatic analysis gives a permanent view of targets based on sequence and therefore reveals new targets that could be influenced under other cellular conditions. Since sequence similarity, binding, and expression are intrinsically linked, the probability of a direct SarA target is higher than an indirect target to be found in all experiments. Thus, the use of combinatory RNA-Seq, ChIP-Seq, and bioinformatics approaches enriches the value of each individual experimental approach and allows the hierarchical grouping of results based on how constantly a target gene can be found. The SarA regulon is huge, and SarA appears as a multifaceted transcriptional regulator responsible for the positive or negative regulation of diverse sRNA or mRNA genes; this work highlights how the combinatory use of transcriptomics proves to be mutually beneficial when such a complex regulon is being investigated.

Despite decades of research on SarA, we have only scratched the surface of the *sarA* regulon. While previous studies have focused on understanding key interactions with strong transparent phenotypes, like quorum sensing and biofilm production, newly developed methods have allowed us to discover finer interactions for SarA and regulators like it.

Harnessing the power of a multiomics approach and integrating the strong predictive aspects of RNA-Seq as well as concrete biochemical determinations of ChIP-Seq has allowed the precise discovery of direct regulatory targets, without the need for multiple double mutants to establish regulatory chains. Furthermore, by using unbiased *in silico* discovery methods such as GLAM2Scan and using a probabilistic matrix rather than a rigid consensus sequence, we were able to explore regulatory targets that are difficult to detect using *in vivo* approaches (which can be heavily biased by the strain tested, medium composition, growth conditions, stresses, etc.). Nevertheless, *in silico*-discovered targets need to be validated by experimental studies.

Not only does this study echo and confirm known SarA regulatory targets, it also helps fill gaps in knowledge concerning sRNAs regulated by *sarA*, shows active phases of *sarA* expression, and elucidates subtle regulatory roles. As some sRNAs have been shown to be involved in virulence, antibiotic resistance, and biofilm formation, it could be of interest to screen SarA-regulated sRNAs to determine whether some are linked to SarA virulence and antibiotic resistance phenotype (38, 39, 94, 95). We also show that SarA regulates multiple leukocidins in late stationary phase. This study also brings up some interesting implications due to the preferential binding of SarA to the νSAβ PI. Hopefully, this sort of two-pronged integrative approach can be used for other regulators in S. aureus, both to establish clear direct regulatory networks and to find novel regulatory targets that can then be examined mechanistically in subsequent studies.

## MATERIALS AND METHODS

### Bacterial strains and culture conditions.

The strains and plasmids and the primers constructed and used in this study are summarized in Table S1A and B, respectively. Escherichia coli strains were grown at 37°C in LB (MoBio), and 50 μg/ml ampicillin or kanamycin was added when necessary. S. aureus strains were grown at 37°C in either brain heart infusion (BHI) medium or tryptic soy broth (TSB; both from Oxoid), with antibiotics added when needed (10 μg/ml erythromycin, chloramphenicol, or tetracycline or 250 μg/ml kanamycin). All experiments were done with S. aureus HG003 (55), using S. aureus RN4220 as an intermediate.

### Purification of SarA from E. coli.

The *sarA* coding sequence was inserted into pET42a in-frame with the 6×His N-terminal tag and transformed in BL21 Escherichia coli. SarA expression was induced by adding 1 mM isopropyl-β-d-1-thiogalactopyranoside (IPTG). Cells were harvested, washed, and resuspended in lysis buffer (10 mM HEPES [pH 7.5], 500 mM NaCl). Purification was done as previously described (28).

### RNA extraction and Northern blotting.

RNA extraction and Northern blot analyses were performed as previously described (16, 28). DNA probes for RNA detection are listed in Table S1B. For sRNA, 15 μg each of total RNAs were separated on 8% polyacrylamide–8 M urea gels and transferred onto Hybond-N+ membranes (Amersham, USA). For mRNA, 10 μg each of total RNAs were separated on 1% agarose-formaldehyde gels and transferred onto Hybond-H+ membranes (Amersham, USA). Specific ^32^P-labeled probes were hybridized in ExpressHyb solution (Clontech), then washed, exposed, and scanned with a PhosphorImager (Molecular Dynamics). Loading controls were performed with either transfer-messenger radiolabeled RNA (tmRNA), 5S rRNA, or 16S RNA or by using 23S and 16S ethidium bromide labeling.

### RNA-Seq.

RNA-Seq was performed as described by Bronsard et al. (96). Overnight cultures of S. aureus were diluted in fresh TSB broth to an optical density at 600 nm (OD_600_) of 0.1 and then cultured at 160 rpm for 2 h or 4.5 h at 37°C. Total RNA was extracted as described above and treated with amplification-grade DNase I (Invitrogen) to remove genomic contaminations. The absence of DNA was checked by qPCR in an Applied Biosystems 7500 instrument, and RNA integrity was verified on a Bioanalyzer (Agilent). rRNAs were depleted using a Ribo-Zero magnetic kit (Epicentre) according to the manufacturer’s recommendations. Stranded cDNA libraries were prepared using the NEBNext Ultra directional RNA library preparation kit for Illumina (New England Biolabs). The concentration, quality, and purity of the libraries were determined using a Bioanalyzer, a Qubit fluorometer (Invitrogen), and a Nanodrop spectrophotometer (Thermo Scientific). Libraries were sequenced on an Illumina HiSeq 1500 system (high output, 200 cycles, paired end), per the manufacturer’s instructions. Between 17.5 and 22 million reads were obtained per replicate. The NCTC8325 strain genome sequence and annotation file (in GFF format) were obtained from NCBI (http://srd.genouest.org/browse/NCTC8325).

All of the SRD *srn* genes described for NCTC 8325 were added to this GFF. Quality control of RNA-Seq reads and read mappings was performed as previously described (96). SAM files were filtered on bitwise flag values (97) to keep only properly paired reads and counted by HTSeq count (98) for stranded libraries. Then, differential expression analyses were calculated using DESeq (99). BAM files were visualized using the Artemis browser (100). Based on the results from HTSeq (see the supplemental material), an FPKM normalization (fragments per kilobase per millions of fragments mapped) was done for each transcript under each condition. From genes with FPKM higher than 10 under at least one condition, we selected those with three transcriptional variations between HG003 and HG003 Δ*sarA* mutant strains under at least one condition.

### ChIP assay.

ChIP was performed as described elsewhere (69). A *sarA* mutant of HG003 harboring the shuttle vector pSK236, containing P1 *sarA*-*myc*, was grown to post-exponential phase of growth (∼4 h) in 100 ml of TSB medium containing 10 μg per ml of chloramphenicol. The *sarA* P1 promoter, one of the three native *sarA* promoters, allows high expression of *sarA* while retaining autoregulation.

Formaldehyde was added to a final concentration of 1% to the growing cells, and after 20 min of incubation, glycine was added to a final concentration of 0.5 M. Fixed cross-linked cells were harvested by centrifugation and washed twice with Tris-buffered saline (pH 7.5). Cells were resuspended in 5 ml of lysis buffer (25 mM Tris-Cl [pH 7.5], 100 mM NaCl, 10 mM EDTA, 0.5 mM dithiothreitol [DTT], 0.1% Triton X-100) containing 1.0 mM phenylmethylsulfonyl fluoride (PMSF) and 50 μg per ml of lysostaphin and incubated for 30 min and 15 min at 37°C and 4°C, respectively. The lysed cells were diluted with IP buffer (50 mM HEPES-KOH [pH 7.5], 150 mM NaCl, 1 mM EDTA, 1% Triton X-100, and 0.1% SDS), subjected to brief sonication for complete lysis, and centrifuged for 45 min at 30,000 rpm and 4°C to remove cell debris. Supernatant containing the cytosolic fraction of proteins and nucleic acids was sheared by sonication to an average DNA fragment size between 200 and 500 bp. Sheared SarA-Myc tag cross-linked protein-DNA complex was immunoprecipitated by mixing with anti-Myc monoclonal antibody (5 μg per ml) and incubated for 2 h at 4°C with gentle mixing. A control experiment was performed without any added anti-Myc antibody.

Samples were incubated with washed protein A-Sepharose beads (20 μl per ml), and incubation was continued for another 90 min. Samples were washed twice with IP buffer, once with IP buffer containing 500 mM NaCl, once with wash buffer (20 mM Tris-Cl [pH 8.0], 250 mM LiCl, 1 mM EDTA, 0.5% Nonidet-P40, and 0.5% sodium deoxycholate), and once with TE (20 mM Tris-Cl [pH 7.5], 1 mM EDTA). Finally, bound DNA-protein complexes were eluted by incubating beads with elution buffer (50 mM Tris-Cl [pH 7.5], 10 mM EDTA, 1% SDS) at 65°C for 15 min. DNA fragments were released from DNA-protein complexes by de-cross-linking in half-strength elution buffer containing 100 μg per ml of proteinase K for 1 h at 55°C. Each experiment was performed in three duplicates, and released DNA fragments were pooled for further analysis. Eluted DNA was purified using a PCR purification kit (Thermo Fisher Scientific, USA) and quantified using Nanodrop (Thermo Scientific, USA). All ChIPs were performed at least three times.

To identify the sequence of the DNA fragments isolated from ChIP experiments, direct sequencing (ChIP-Seq) was performed using an Illumina HiSeq 4000 sequencer at our Institutional Molecular Biology Core Facilities (Dartmouth-Hitchcock). DNA was quantified by using Qubit (Life Technologies), and DNA integrity was assessed with a 2100 Bioanalyzer (Agilent Technologies). An amount of 50 ng of total DNA was used for the library preparation. The libraries were pooled at equimolarity and loaded at 2 nM for clustering. HiSeq 4000 sequencing was performed, resulting in approximately 81 million total paired-end 150-bp reads for six control samples and 95 million total reads for six treatment samples. Adaptor trimming was done before reads were mapped to S. aureus NCTC8325 (NC_007795.1) using CLC Genomics Workbench 11. Approximately 83 million reads aligned uniquely to the genome for the treatment samples (83%) and 51 million reads for the controls (63%).

The CLC Genomics Workbench ChIP pipeline was used for analysis and peak calling. Briefly, an initial maximum *P* value of 0.1 for peak calling of 0.1 was used for analysis. Quality control showed a high number of reads (82 million) and relative strand correlations of 0.884 but a somewhat low normalized strand coefficient of 1.012 for treatment data. Because of this, additional thresholding was done to increase the stringency, setting the *P* value cutoff to E−250 and the minimum peak value cutoff to 30.

### Consensus motif analysis.

To determine which sRNAs are regulated by SarA based on our ChIP output, we used R to scan the SRD sRNA database in S. aureus NCTC8325 sRNA, which had the center of a significant SarA ChIP peak up to 100 bp upstream of its TSS +1 (29) We compiled a list of direct sRNA targets for further analysis using the intersection of the sRNA sets found in ChIP-Seq and in RNA-seq.

For *de novo* motif discovery, we used the gapped-motif-finding tool GLAM2 v.1056 (from the MEME suite) for both our direct targets and previously confirmed sRNAs, adding up to 15 sequences of 100 bp from upstream of the TSS +1 of verified sRNAs (29, 79). Although AT rich, the best motif generated still had an alignment score of 243.077, followed by a similar AT-rich motif with alignment score of 236.693, indicating a good consensus for the SarA motif. Alignment with sRNA 5′ sequences had an average marginal score of 23.13 ± 4.158 with a range from 16.7 to 31.8. We then used GLAM2Scan v.1056 with this motif to search the genome of S. aureus NCTC 8325 uid237 in the GenBank Bacterial Genome and Proteins database, using both strands for 200 alignment hits. Each hit was manually curated to find for mRNAs and sRNAs which contain the generated SarA binding motif.

### EMSA.

The gel shift assay was adapted from reference 28. Promoters were amplified from the S. aureus HG003 genome by PCR using specific primers (listed in Table S1B). DNA probes (250 nt; 400 ng) were labeled with [γ-^32^P]ATP using T4 polynucleotide kinase (New England Biolabs). Binding reactions were carried out as previously described (28). Briefly, binding reaction medium containing 10 fmol DNA template, 20 mM HEPES-KOH (pH 7.6), 20% glycerol, 0.2 mM EDTA, 0.1 M KCl, 0.1 M MgCl_2_, 5 mM DTT, and 0.2 g poly(dI-dC) (0.15 μg) was incubated, when necessary, with purified SarA for 30 min at 30°C. To determine specificity fixation, competition assay was performed with increasing quantities of specific probe (unlabeled promoter) and nonspecific probe (unlabeled 16S RNA promoter). Samples were loaded on native 8% polyacrylamide gels. Detection was done with a Typhoon FLA 9500 (GE Healthcare).

### Beta-lactamase/reporter gene assay.

Overnight cultures were adjusted to an OD_600_ of 0.1. For each time point, cells were centrifuged and resuspended in 1× phosphate-buffered saline (PBS) to obtain an equal cell density throughout the assay. Cell lysis was performed using 0.7 mg/ml lysostaphin, 0.2 U/μl Benzonase, and 0.1 mM MgCl_2_ at 37°C for 20 min. After addition of 0.25 mg/ml nitrocefin (a β-lactamase substrate), the reaction mix was incubated 10 min at room temperature. β-Lactamase activity was quantified on a BioTek instrument every 10 min for 40 min at a wavelength of 492 nm. This activity was normalized against protein quantities as determined by a Bradford assay.

### Statistical analysis.

For statistical analysis, the two-tailed Mann-Whitney test was used for reporter gene experiments. Data were expressed as means ± standard deviations.

### Transformation efficiency.

Wild-type HG003 and the Δ*sarA* and Δ*sarA/sarA*^+^ variants were prepared with an electrocompetent protocol (101). Two hundred nanograms of plasmids pCN35, pCN35-*sprG2*Flag, and pCN35-*sprG2*Flag+*sprF2* was used to transform HG003 electrocompetent cells with dimethyl sulfoxide (DMSO) at 2,500 V. Cells were incubated 1 h at 37°C. One hundred microliters was spread on BHI with 10 μg/ml chloramphenicol and incubated 24 h at 37°C. The transformation efficiency was calculated per bacterium per μg of DNA.

### Data visualization using IGV, SnapGene, and CiVi.

To visualize RNA-Seq and ChIP-Seq data, we used IGV (72), SnapGene software (Insightful Science), and the CiVi circular genome visualizer (102).

### Data availability.

The data discussed in this publication have been deposited in NCBI's Gene Expression Omnibus and are accessible through GEO Series accession number GSE174164.
