# Supplementary figures and images for: Expanding the Staphylococcus aureus SarA Regulon to Small RNAs
Source: mSystems. 2021 Oct 12;6(5):e00713-21. doi: 10.1128/mSystems.00713-21 (PMC8510525; doi:10.1128/mSystems.00713-21)

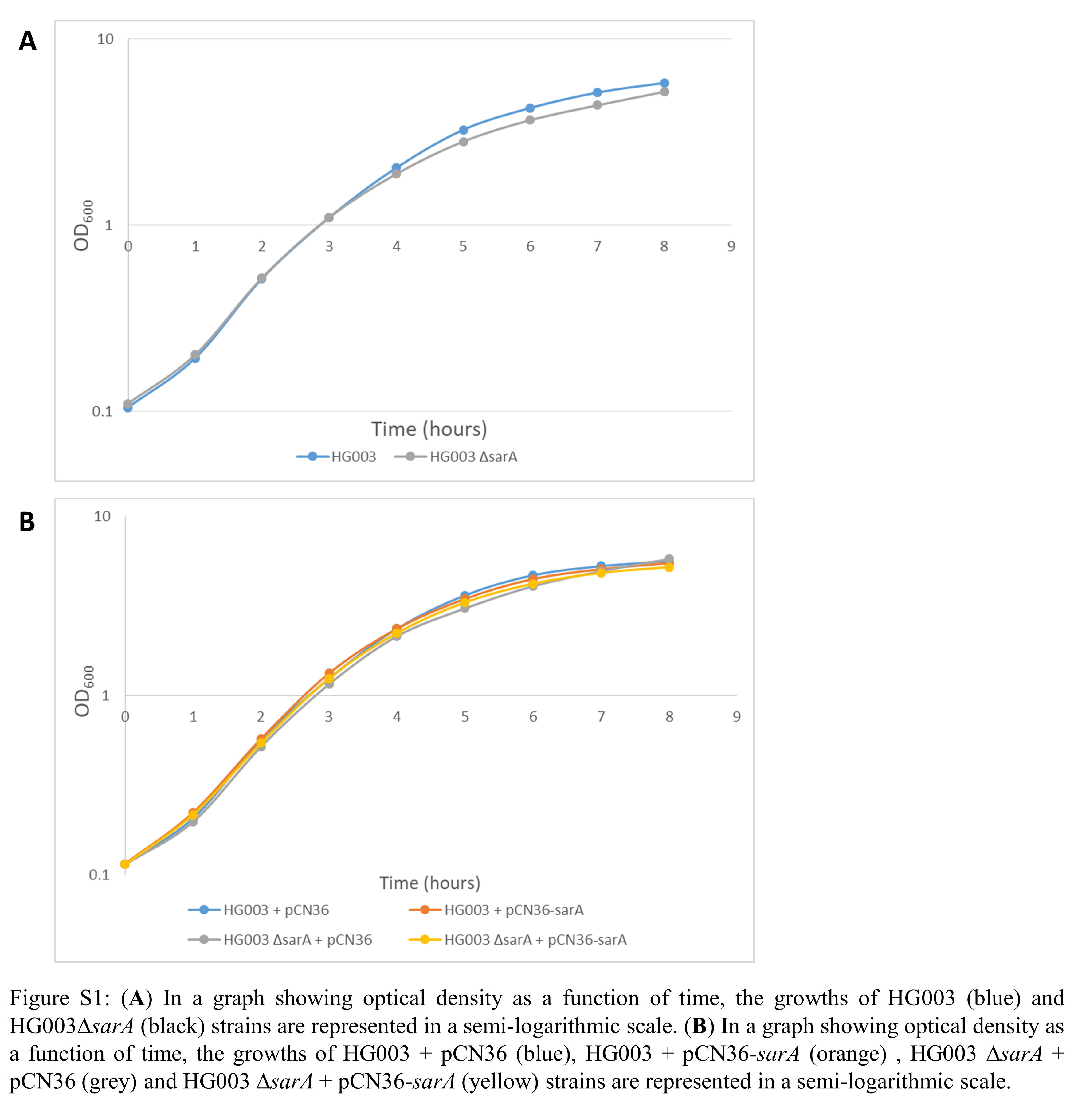

Supplement: FIG S1 [file msystems.00713-21-sf001.tif]

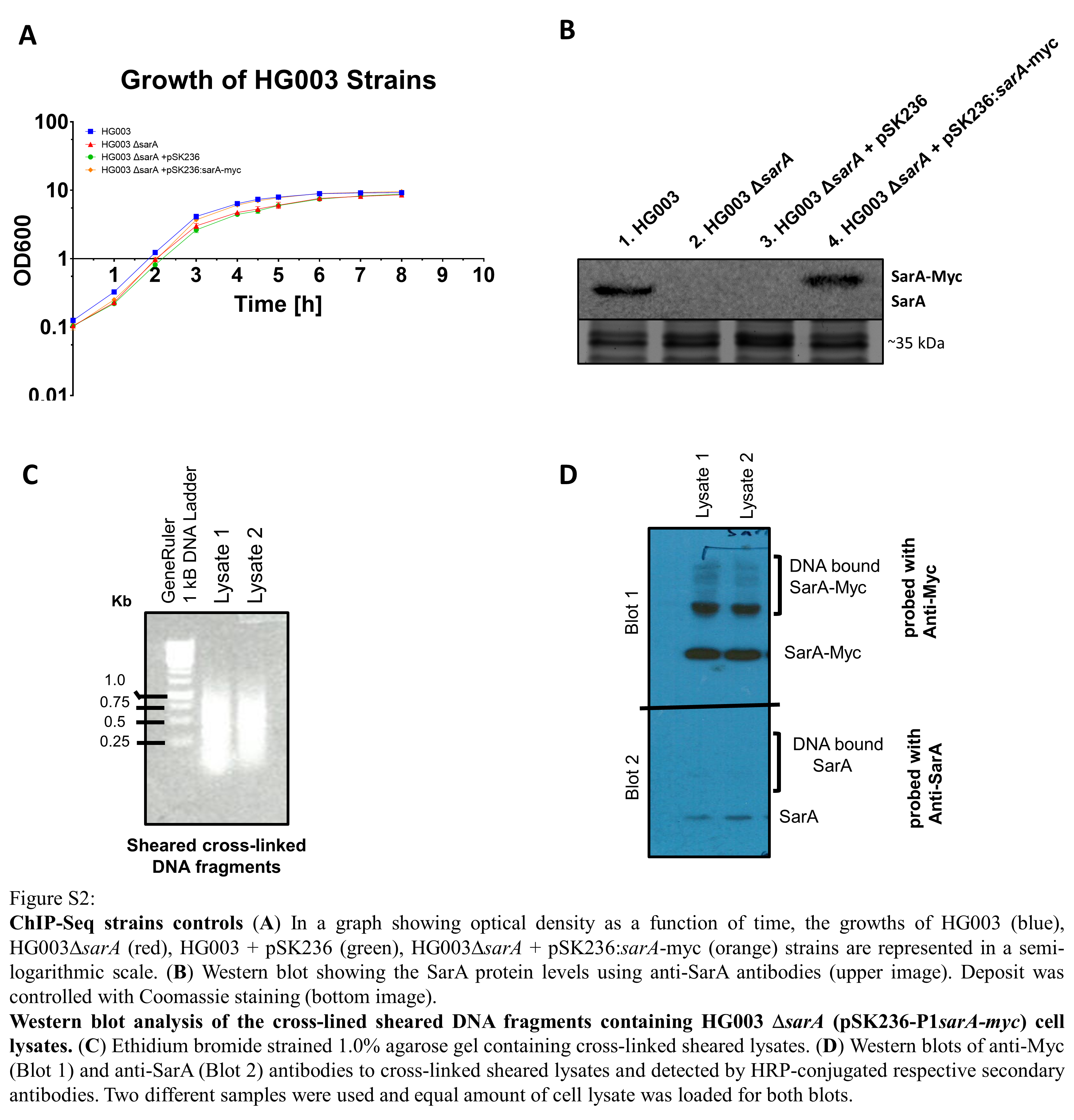

Supplement: FIG S2 [file msystems.00713-21-sf002.tif]

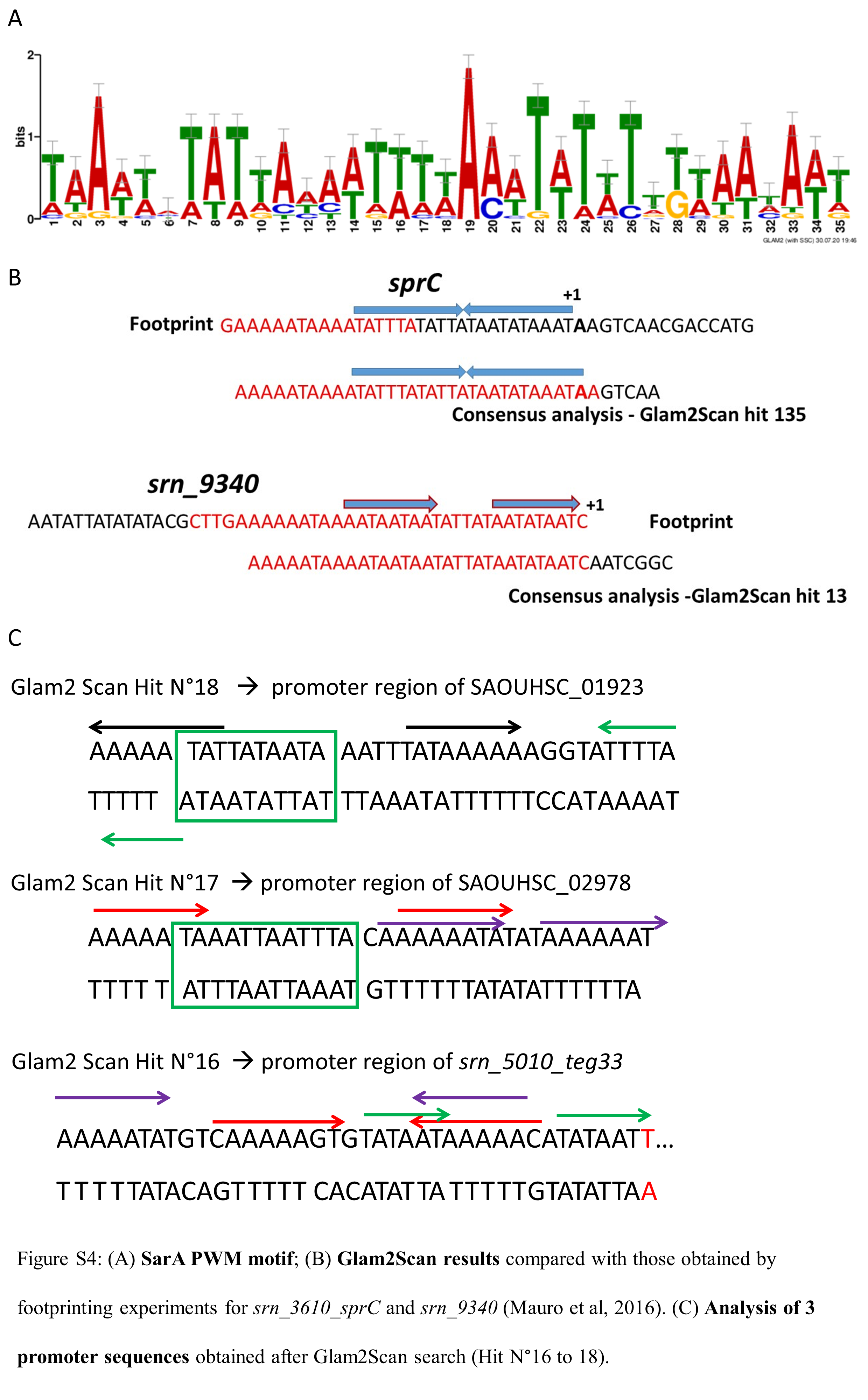

Supplement: FIG S4 [file msystems.00713-21-sf004.tif]
