# Supplementary material for: Expanding the Staphylococcus aureus SarA Regulon to Small RNAs
Source: mSystems. 2021 Oct 12;6(5):e00713-21. doi: 10.1128/mSystems.00713-21 (PMC8510525; doi:10.1128/mSystems.00713-21)
Supplement: TABLE S1 [file msystems.00713-21-st001.docx]

| **Strain** | **Strain** | **Strain Characteristics** | **References** |
| --- | --- | --- | --- |
| *Escherichia coli* | Xl1 blue | Used for cloning, transformation  Δ(ara-leu) 7697 araD139 fhuA ΔlacX74 galK16 galE15 e14-ϕ80dlacZΔM15 recA1 relA1 endA1 nupG rpsL (StrR) rph spoT1 Δ(mrr-hsdRMS-mcrBC)  Restriction-defective derivative of 8325-4 used for transformation (intermediary strain)  Considered wild type strains; rsbU and tcaR restored strain 8325  *sarA* ::*ermC* pCN36  pCN36-*sarA*  *sarA* ::*ermC* (pSK236 with P1*sarA* -myc) HG003 *sarA* ::*ermC* (pCN36)  HG003 *sarA* ::*ermC* (pCN36 with P1*sarA* )  *sarA* ::*ermC* complemented with single copy of the P2*sarA* DNA fragment on to the *geh* locus on chromosome |  |
| *Staphylococcus aureus* | RN4220 |  | Kreiswirth et *al* , 1983 |
| *Staphylococcus aureus* | HG003 |  | Herbert et *al* ., 2010 |
| *Staphylococcus aureus* | HG003 |  | Mauro et *al* , 2016 |
| *Staphylococcus aureus* | HG003 |  | This study |
| *Staphylococcus aureus* | HG003 |  | This study |
| ALC8693 | HG003 |  | This study |
| ALC8960 | HG003 |  | This study |
| ALC8961 | HG003 |  | This study |
| ALC8959 | HG003 |  | This study |
| **Plasmids** | ***S. aureus* resistance** | **Plasmid Characteristics** | **References** |
| pSK236 | Cm^R^ | pSK236  pSK236 containing P1 SarA-myc pCN36  pCN36 expressing *sarA* from endogenous promoter (P1) pCN35  pCN35 with *sprG2* -3XFLAG sequence before the termination codon of the predicted internal coding pCN35-*sprG2* -FLAG with *sprF2* under the control of SprF2 endogenous promoter  contains β−Lactamase gene without promoter  pCN41c with *sprA2 _AS_* promoter | Gaskill & Khan, 1988 |
| pSK236_*sarA* -myc | Cm^R^ |  | This study |
| pCN36 | Tet^R^ |  | Charpentier, 2004 |
| pCN36-*sarA* | Tet^R^ |  | Mauro et *al* , 2016 |
| pCN35 | Cm^R^ |  | Riffaud et *al* , 2018 |
| pCN35-*sprG2* -FLAG | Cm^R^ |  | Riffaud et *al* , 2018 |
| pCN35-*sprG2* -FLAG+*sprF2* | Cm^R^ |  | Riffaud et *al* , 2018 |
| pCN41c | Cm^R^ |  | Mauro et al, 2016 |
| pCN41c_P*sprA2 _AS_* | Cm^R^ |  | Amiot *et al* ., 2019 |

(A)

(B)

| **NB probes** | **Locus tag** | **Gene Name** | **Primer Name** | **Sequence** |
| --- | --- | --- | --- | --- |
| **sRNA** | *srn_2230* | *sprG2* | sprG2_probe | AACTTTGACATAGAATGATGGTTAA |
|  | *srn_3950* | *teg16* | teg16_probe | TTTGAAGTTGTCGGTTGATG |
|  | *srn_4540* | *sprA2 AS* | sprA2as_probe | TTTAGATGTTCAATTTATTATGTAA |
|  | *srn_4550* | *sprA2* | sprA2_probe | TTTATTGTTGCGCGTTCGTA |
|  | *srn_0860* | *rsaOB* | NB_rsaOB_5' | ACAGTTTAATGAAACGTAAACACAA |
|  |  |  | NB_rsaOB | CTT CCG TCC TTC GTA CCC GAA |
|  | *srn_9335* | *tsr29* | NB_tsr29_5' | AGGGGGTTTTATCTTTGGAAAAA |
|  |  |  | NB_tsr29_3’ | CGTCTTTTCACAACCAAAGCTA |
|  | *srn_1640* | *rsaD* | NB_rsaD_5' | GTC TGC TAT AAT GTT TTT AAC TTC TTC AA |
|  |  |  | NB_rsaD_3’ | AAC CAA GTG CAC ATG GTA ATA CAC TTG |
|  | *srn_0455* | *tsr9* | NB_tsr9_5' | CAA AGT TCA GAT TTG AGC TTT GCT |
|  |  |  | NB_tsr9_3’ | TTT GAA TAC AAA TAA TCA AAT CAT TGA TAA |
| **mRNA** | SAOUHSC_01452 | *ald1* | NB_ald1 | TTG TTT AAC CAT CTC ACG AGT ACC A |
|  |  |  | NB_ald1 | ATG TTA GTT GCA GTA GTC AAA GAA TTA |
|  | SAOUHSC_02696 | *fmhA* | NB_fmhA_5' | CAATGCAGACGGTGAAATTGTT |
|  |  |  | NB_fmhA_3’ | GTTGTTGCTTTAATTGTAACGTTT |
|  | SAOUHSC_00913 | *lysR* | NB_lysR_5' | GAACAGCATCTCAATGTGAAACTA |
|  |  |  | NB_lysR_3’ | GCCTCAGTTAGAAGATGATTATTC |
|  | SAOUHSC_00088 | *galE* | NB_galE | AGA TTT GGA AAG AGT TTT GAT AAC T |
|  |  |  | NB_galE | CGA CAT TTC CAC TTC TTT AGC TGT A |
|  | SAOUHSC_00961 | *comK1* | NB_comK1_5' | AGCAGAGACAAACCGCATTA |
|  |  |  | NB_comK1_3’ | CGGGATTTTTTGATATCATGCGAG |
|  | SAOUHSC_02820 | *HP (ABC transporter)* | NB_02820_5' | CTCTAGTTTCCAAAGCGGGA |
|  |  |  | NB_02820_3’ | GGATAGAGTTTGTGATTTTAACTAA |
|  | SAOUHSC_00555 | *(Hydrolase)* | NB_00555_5' | TTT GAT TTT GAT GGT ACG TGG GAC |
|  |  |  | NB_00555_3’ | CGG TAT TAA TTT CTG ATG GAT CAT TA |
|  | SAOUHSC_00975 | *HP (putative oxidoreductase)* | NB_00975_5' | ATG AAA TTA GGT ATG CTT TTA ATT AGA |
|  |  |  | NB_00975_3’ | ATTG TAG CCA TTT CTT CCA GCT GTA |
|  | SAOUHSC_02127 | *scpA* | NB_scpA_5' | ATG AAA AGA AAC TTT CCA AAA TTA |
|  |  |  | NB_scpA_3’ | AGA AGT TGT GGA CTT CTT CCC TGA |
| **EMSA** | **Locus tag** | **Gene Name** | **Primer Name** | **Sequence** |
| **sRNA** | *srn_2230* | *sprG2* | sprG2_Fwd | CCGTTGCACAAATTGTAGCT |
|  |  |  | sprG2_Rev | TTTACTCAGATAGCCACCAT |
|  | *srn_0860* | *rsaOB* | rsaOB_Fwd | AAGAGAGAATTCTAAATATATTTTATGTATTCTAAATGAGTC |
|  |  |  | rsaOB_Rev | AAGAGAGAATTCCTCTCTGTATAAGCAGGGTGTGT |
|  | *srn_9335* | *tsr29* | tsr29_Fwd | AAGAGAGAATTCGAAATTTAAGAGTTAAACAAAAAATAT |
|  |  |  | tsr29_Rev | AAGAGAGAATTCTTTAACTTGCACGTCTAAATCAAGA |
|  | *srn_3950* | *teg16* | teg16_Fwd | GTTGATTCTCCTATATTATG |
|  |  |  | teg16_Rev | TAAGCATTTTATTATACTCC |
|  | *srn_0455* | *tsr9* | tsr9_Fwd | AGAAAGGAATTCGCCATTCATATCCCCTTCCATA |
|  |  |  | tsr9_Rev | AAGAGAGAATTCAAAAAATTTATCAATGATTTGATTATTTGTA |
|  | *srn_4540* | *sprA2 AS* | sprA2as_Fwd | GATTTGTATCCCAATGTCCC |
|  |  |  | sprA2as_Rev | GATTTGGTGTATTTAGATGTTC |
|  | *srn_4550* | *sprA2* | sprA2_Fwd | CAGCAGAAATTGCACCATTTG |
|  |  |  | sprA2_Rev | GTTAAAGTAACGCCCGAGATG |
|  | *srn_1640* | *rsaD* | rsaD_Fwd | GTC TGC TAT AAT GTT TTT AAC TTC TTC AA |
|  |  |  | rsaD_Rev | AAC CAA GTG CAC ATG GTA ATA CAC TTG |
|  | *srn_5010* | *teg33* | teg33_Fwd | GAATTATTGCAAAATATATATC |
|  |  |  | teg33_Rev | AATAACTGCTAATTTTTTCATC |
| **mRNA** | SAOUHSC_01955 | *lukE* | lukE_Fwd | AAAGAAGAATTCAGCAGCATAAATGAATAAATTGTGT |
|  |  |  | lukE_Rev | AAGGAAGAATTCCTACTGACAAAGTTGCAGCTAACA |
|  | SAOUHSC_01452 | *ald1* | ald1_Fwd | AAGAGAGAATTCAACTATAAATCAAATGGAGTATAAGA |
|  |  |  | ald1_Rev | AAGAGAGAATTCTTTCATTTGTGAATAATTTCACAATTA |
|  | SAOUHSC_02696 | *fmhA* | fmhA_Fwd | CAATGCTCCCACTCCTAC |
|  |  |  | fmhA_Rev | CGTTACAAAGTTCATTTTATC |
|  | SAOUHSC_00913 | *lysR* | lysR_Fwd | GAATATTAAATTCATCCTCG |
|  |  |  | lysR_Rev | TCATGAACTTACCTCTTTC |
|  | SAOUHSC_00088 | *galE* | galE_Fwd | TCTAATGCATCAGTTGAAAGC |
|  |  |  | galE_Rev | CTAAATGCGACCCAATAAAA |
|  | SAOUHSC_00961 | *comK1* | comK1_Fwd | GTGACAAACGCTTCTAAAG |
|  |  |  | comK1_Rev | ACATAAGCAAAACCTCGC |
|  | SAOUHSC_02820 | *HP (ABC transporter)* | 02820_Fwd | CAGAATTTACCCTGTCTTCC |
|  |  |  | 02820_Rev | CATCCATATCACTATCTCCT |
|  | SAOUHSC_00555 | *(Hydrolase)* | 00555_Fwd | AAGAGAGAATTCTTGAACAAGCATCTCAATTATTTTTA |
|  |  |  | 00555_Rev | AGAGAAGAATTCAATATTAACACCCCTATTCGTTTA |
|  | SAOUHSC_00975 | *HP (putative oxidoreductase)* | 00975_Fwd | AAGAGAGAATTCATTTCCCTATAATCTTTGCACTAT |
|  |  |  | 00975_Rev | AGAAGAGAATTCCAACATTCATATTTTTAAAACATA |
|  | SAOUHSC_02127 | *scpA* | scpA_Fwd | AAGAGAGAATTCTTTCAAAATCGACTAATATATTTTCA |
|  |  |  | scpA_Rev | AGAAGAGAATTCCTTTATTTTATTATAAAATAATTA |
|  | Control | 16S RNA gene promoter | Promot_16S_Fwd | CATTGAAAACTGAATGACAA |
|  |  |  | Promot_16S_Rev | AAGTTATCCCAGTCTTATAG |

Table S1: Strains and plasmids (A) and primers (B) used in this study
