# Supplementary material for: Expanding the Staphylococcus aureus SarA Regulon to Small RNAs
Source: mSystems. 2021 Oct 12;6(5):e00713-21. doi: 10.1128/mSystems.00713-21 (PMC8510525; doi:10.1128/mSystems.00713-21)
Supplement: TABLE S2 [file msystems.00713-21-st002.docx]

| **Gene group and annotation** | **Gene name** | **Assignment** | **Differential expression HG003 Δ*sarA* vs HG003** | | | |
| --- | --- | --- | --- | --- | --- | --- |
|  |  |  | **Exponential (2h)** | | **Early stationnary (4.5h)** | |
|  |  |  | **Fold-change** | **p-value** | **Fold-change** | **p-value** |
| **Genes repressed by SarA** | | | | | | |
| **Transport and binding proteins & protein fate** | | | | | | |
| SAOUHSC_00010  SAOUHSC_00012 | *azlC*  *azlD* | Azaleucine resistance protein  Type II secretion system protein F |  |  | **6.3**  **6.4** | 1.28E-20  5.43E-19 |
| SAOUHSC_00102  SAOUHSC_00103 SAOUHSC_00104 SAOUHSC_00105 | *phnE1*  *phnE2 phnC phnD* | Phosphonates ABC transporter permease  Phosphonates ABC transporter permease Amino acid ABC transporter ATP-binding protein  Phosphonate ABC transporter substrate-binding protein | **13.4**  **18.8**  **39.1**  **41.8** | 2.75E-95  1.33E-86  1.92E-126  2.43E-193 | **19.1**  **21.3**  **23.0**  **19.0** | 2.27E-48  1.47E-47  5.15E-51  1.75E-49 |
| SAOUHSC_00106 |  | Putative ABC-type transporter | **11.4** | 1.01E-69 | **10.6** | 7.33E-32 |
| SAOUHSC_00168  SAOUHSC_00169 |  | Nickel ABC transporter  Nickel ABC transporter |  |  | **6.1**  **5.5** | 4.99E-18  3.42E-17 |
| SAOUHSC_00170 |  | Peptide ABC transporter substrate-binding protein |  |  | **6.3** | 2.21E-21 |
| SAOUHSC_00175  SAOUHSC_00176 | *malK*  *malE* | Multiple sugar-binding transport ATP-binding protein  Extracellular solute-binding protein |  |  | **5.8**  **3.7** | 6.24E-18  4.58E-10 |
| SAOUHSC_00249  SAOUHSC_00250 SAOUHSC_00251 SAOUHSC_00253 |  | ABC transporter  Hypothetical protein Hypothetical protein Hypothetical protein | **5.6**  **6.6**  **7.6**  **17.0** | 4.63E-49  1.18E-56  1.78E-63  7.79E-147 | **3.3**  **4.5**  **4.6**  **8.9** | 8.10E-09  1.67E-13  3.01E-13  1.16E-27 |
| SAOUHSC_00257 | *esxA* | WXG100 family type VII secretion effector | **18.0** | 6.41E-155 | **8.9** | 1.18E-24 |
| SAOUHSC_00258  SAOUHSC_00259 SAOUHSC_00260 SAOUHSC_00261 SAOUHSC_00262 | *esaA*  *essA esaB essB essC* | Type VII secretion protein  Type VII secretion protein Type VII secretion protein Type VII secretion protein Type VII secretion protein | **12.7**  **8.8**  **14.4**  **10.2**  **7.3** | 5.10E-121  2.16E-50  6.94E-21  6.95E-80  3.96E-80 | **11.2**  **9.3**  **7.9**  **7.3**  **5.8** | 7.60E-34  1.29E-25  1.15E-09  4.02E-23  6.78E-19 |
| SAOUHSC_00264 | *esxC* | Type VII secretion protein | **5.4** | 9.94E-27 | **6.7** | 7.47E-17 |
| SAOUHSC_00265 | *esxB* | Type VII secretion protein | **5.1** | 5.43E-25 | **6.9** | 1.84E-17 |
| SAOUHSC_00266 | *esaE* | Type VII secretion protein | **6.1** | 3.24E-43 | **6.2** | 2.99E-18 |
| SAOUHSC_00267 | *esxD* | Type VII secretion protein | **6.2** | 8.01E-27 | **5.1** | 7.29E-12 |
| SAOUHSC_00282 | *brnQ2* | Branched-chain amino acid transport system II carrier protein |  |  | **3.4** | 3.39E-09 |
| SAOUHSC_00290 |  | Phosphotransferase system, EIIC |  |  | **3.6** | 1.72E-09 |
| SAOUHSC_00423  SAOUHSC_00424 SAOUHSC_00426 | *metN2*  *metP2 metQ2* | D-methionine ABC transporter  Methionine ABC transporter permease ABC transporter substrate-binding protein |  |  | **15.6**  **17.2**  **12.0** | 5.85E-43  8.11E-47  5.25E-33 |
| SAOUHSC_00828 |  | L-lysine exporter |  |  | **3.1** | 1.23E-07 |
| SAOUHSC_00842  SAOUHSC_00843 SAOUHSC_00844 | *metN1*  *metP1 metQ1* | Methionine ABC transporter  Methionine ABC transporter Methionine ABC transporter |  |  | **3.5**  **4.4**  **5.1** | 6.47E-10  1.00E-13  3.39E-15 |
| SAOUHSC_00903 | *spsB* | Signal peptidase IB |  |  | **3.3** | 5.55E-09 |
| SAOUHSC_00923  SAOUHSC_00924 SAOUHSC_00925 SAOUHSC_00926 SAOUHSC_00927 | *opp-3B*  *opp-3C opp-3D opp-3F opp-3A* | Oligopeptide transport system permease  Oligopeptide transport system permease Oligopeptide transport system permease Oligopeptide transport system permease Oligopeptide ABC transporter |  |  | **3.2**  **4.1**  **3.8**  **3.8**  **3.5** | 2.42E-08  3.48E-12  8.95E-11  1.41E-10  7.54E-09 |

| SAOUHSC_00949  SAOUHSC_00986 | *sspC* | Sodium/alanine symporter family protein  Cysteine protease |  |  | **8.7**  **26.3** | 1.31E-28  8.92E-55 |
| --- | --- | --- | --- | --- | --- | --- |
| SAOUHSC_00987 | *sspB* | Cysteine protease |  |  | **26.1** | 9.28E-51 |
| SAOUHSC_00988 | *sspA* | Glutamyl endopeptidase |  |  | **34.2** | 1.20E-57 |
| SAOUHSC_01180 |  | Outer membrane assembly lipoprotein | **3.1** | 9.88E-25 | **3.1** | 7.17E-08 |
| SAOUHSC_01440 |  | Conserved hypothetical integral membrane protein |  |  | **3.5** | 1.01E-09 |
| SAOUHSC_01935 | *splF* | Serine protease | **4.2** | 1.31E-15 | **24.4** | 3.06E-55 |
| SAOUHSC_01936 | *splE* | Serine protease |  |  | **28.0** | 2.98E-59 |
| SAOUHSC_01938 | *splD* | Serine protease | **6.0** | 1.21E-18 | **29.2** | 1.36E-59 |
| SAOUHSC_01939 | *splC* | Serine protease | **7.1** | 2.25E-21 | **36.9** | 4.22E-66 |
| SAOUHSC_01941 | *splB* | Serine protease | **17.5** | 1.22E-34 | **52.4** | 5.50E-75 |
| SAOUHSC_01942 | *splA* | Serine protease | **24.3** | 3.00E-50 | **62.3** | 2.62E-77 |
| SAOUHSC_02247 | *ktrB* | Potassium uptake protein | **10.1** | 4.40E-105 | **7.2** | 9.33E-22 |
| SAOUHSC_02270 | *nrgA* | Ammonium transporter |  |  | **6.7** | 1.21E-15 |
| SAOUHSC_02557 |  | Urea transporter |  |  | **5.0** | 1.24E-14 |
| SAOUHSC_02595 |  | Bile acid transporter |  |  | **5.9** | 1.11E-19 |
| SAOUHSC_02622 | *gltS* | Sodium/glutamate symporter | **3.4** | 1.58E-33 | **3.6** | 5.72E-11 |
| SAOUHSC_02667 | *gltT* | Proton/glutamate symport protein |  |  | **4.7** | 1.50E-13 |
| SAOUHSC_02668 |  | Hypothetical protein |  |  | **3.1** | 1.50E-08 |
| SAOUHSC_02704 |  | Cation diffusion facilitator family transporter |  |  | **4.2** | 2.03E-13 |
| SAOUHSC_02763 | *cntF* | Peptide ABC transporter ATP-binding protein | **3.1** | 9.48E-13 |  |  |
| SAOUHSC_02766 | *cntB* | Peptide ABC transporter permease | **3.6** | 2.05E-22 |  |  |
| SAOUHSC_02767 | *cntA* | Peptide ABC transporter substrate-binding protein | **4.0** | 1.15E-41 |  |  |
| SAOUHSC_02773 |  | Aminobenzoyl-glutamate transport protein |  |  | **3.1** | 2.88E-07 |
| SAOUHSC_02806 | *gntP* | Gluconate permease |  |  | **4.0** | 4.21E-10 |
| SAOUHSC_02808 | *gntK* | Gluconate kinase |  |  | **10.0** | 1.65E-28 |
| SAOUHSC_02809 | *gntR* | Gluconate operon transcriptional repressor |  |  | **13.6** | 8.71E-37 |
| SAOUHSC_02820 |  | ABC transporter ATP-binding protein | **23.4** | 1.62E-165 | **44.4** | 1.11E-70 |
| SAOUHSC_02821 |  | Lantibiotic protection ABC transporter permease subunit, MutG family | **18.6** | 3.92E-150 | **36.6** | 4.17E-64 |
| SAOUHSC_02923 |  | Amino acid transporter |  |  | **12.3** | 9.01E-33 |
| SAOUHSC_02943 | *citM* | Citrate transporter | **12.9** | 3.23E-95 | **10.3** | 3.19E-32 |
| SAOUHSC_03018 |  | Cobalt ECF transporter T component |  |  | **9.3** | 3.84E-30 |
| SAOUHSC_03019 |  | ABC transporter ATP-binding protein |  |  | **11.8** | 2.47E-35 |
| SAOUHSC_03020 |  | ECF transporter S component, folate family |  |  | **21.3** | 5.15E-51 |
| SAOUHSC_03021 |  | Adenosyl-fluoride synthase |  |  | **17.9** | 1.50E-46 |
|  | | | | | | |
| **Amino acids biosynthesis** | | | | | | |
| SAOUHSC_00013 | *metX* | Homoserine O-acetyltransferase |  |  | **4.4** | 4.50E-14 |
| SAOUHSC_00147 | *argB* | Acetylglutamate kinase | **7.4** | 2.64E-75 |  |  |
| SAOUHSC_00148 | *argJ* | Bifunctional ornithine acetyltransferase/N-acetylglutamate synthase | **9.9** | 1.34E-99 |  |  |
| SAOUHSC_00149 | *argC* | N-acetyl-gamma-glutamyl-phosphate reductase | **13.4** | 1.52E-115 |  |  |
| SAOUHSC_00150 | *argD* | Ornithine aminotransferase | **29.0** | 7.87E-169 | **3.5** | 4.71E-08 |
| SAOUHSC_00338 | *metE* | 5-methyltetrahydropteroyltriglutamate-- homocysteine S-methyltransferase |  |  | **3.7** | 2.89E-10 |
| SAOUHSC_00339 | *metF* | Bifunctional homocysteine S-methyltransferase/5,10-methylenetetrahydrofolate reductase |  |  | **3.9** | 1.18E-11 |
| SAOUHSC_00340 | *metC* | Trans-sulfuration enzyme family protein |  |  | **4.2** | 1.83E-11 |
| SAOUHSC_00341 | *metI* | O-succinylhomoserine (thiol)-lyase |  |  | **3.2** | 2.99E-07 |

| SAOUHSC_00733 | *hisC* | Histidinol-phosphate aminotransferase |  |  | **4.7** | 5.67E-15 |
| --- | --- | --- | --- | --- | --- | --- |
| SAOUHSC_00898 | *argH* | Argininosuccinate lyase | **3.7** | 3.89E-39 |  |  |
| SAOUHSC_00899 | *argG* | Argininosuccinate synthase | **3.3** | 1.09E-32 |  |  |
| SAOUHSC_01319 | *thrD* | Aspartate kinase | **6.8** | 4.47E-51 | **57.3** | 2.33E-75 |
| SAOUHSC_01320 | *hom* | Homoserine dehydrogenase |  |  | **20.0** | 3.23E-44 |
| SAOUHSC_01321 | *thrC* | Threonine synthase |  |  | **16.7** | 8.50E-40 |
| SAOUHSC_01322 | *thrB* | Homoserine kinase |  |  | **19.0** | 4.66E-43 |
| SAOUHSC_01371 | *trpB* | Tryptophan synthase subunit beta |  |  | **3.5** | 1.08E-07 |
| SAOUHSC_01372 | *trpA* | Tryptophan synthase subunit alpha |  |  | **3.6** | 1.34E-08 |
| SAOUHSC_01395 | *asd* | Aspartate semialdehyde dehydrogenase |  |  | **3.9** | 8.26E-11 |
| SAOUHSC_01396 | *dapA* | 4-hydroxy-tetrahydrodipicolinate synthase |  |  | **3.8** | 8.98E-11 |
| SAOUHSC_01397 | *dapB* | 4-hydroxy-tetrahydrodipicolinate reductase |  |  | **3.5** | 1.44E-09 |
| SAOUHSC_01398 | *dapD* | 2,3,4,5-tetrahydropyridine-2,6-dicarboxylate N-acetyltransferase |  |  | **3.5** | 5.68E-09 |
| SAOUHSC_01832 |  | Phosphoserine transaminase |  |  | **9.0** | 4.92E-28 |
| SAOUHSC_01833 | *serA* | D-3-phosphoglycerate dehydrogenase |  |  | **8.7** | 4.26E-26 |
| SAOUHSC_02281 | *ilvD* | Dihydroxy-acid dehydratase |  |  | **19.2** | 1.75E-49 |
| SAOUHSC_02282 | *ilvB* | Acetolactate synthase large subunit |  |  | **17.8** | 3.23E-46 |
| SAOUHSC_02283 | *ilvH* | Acetolactate synthase 1 regulatory subunit |  |  | **15.1** | 6.07E-33 |
| SAOUHSC_02284 | *ilvC* | Ketol-acid reductoisomerase |  |  | **13.4** | 6.98E-39 |
| SAOUHSC_02285 | *leuA* | 2-isopropylmalate synthase |  |  | **15.2** | 3.72E-41 |
| SAOUHSC_02286 | *leuB* | 3-isopropylmalate dehydrogenase |  |  | **15.5** | 3.16E-43 |
| SAOUHSC_02287 | *leuC* | Isopropylmalate isomerase large subunit |  |  | **17.9** | 8.99E-47 |
| SAOUHSC_02288 | *leuD* | 3-isopropylmalate dehydratase small subunit |  |  | **15.6** | 1.36E-42 |
| SAOUHSC_02289 | *ilvA2* | Threonine dehydratase |  |  | **15.4** | 2.76E-42 |
| SAOUHSC_02970 | *argR* | Arginine repressor | **7.9** | 2.12E-43 | **4.8** | 1.26E-12 |
| SAOUHSC_03012 | *hisD hisG hisZ* | Histidinol-phosphate aminotransferase Histidinol dehydrogenase  ATP phosphoribosyltransferase catalytic subunit  ATP phosphoribosyltransferase regulatory subunit | **4.2**  **5.1**  **4.9**  **4.5** | 1.14E-18  2.66E-34  7.69E-19  2.05E-26 | **8.3**  **17.0** | 3.05E-20  1.65E-27 |
| SAOUHSC_03013 |  |  |  |  |  |  |
| SAOUHSC_03014 |  |  |  |  |  |  |
| SAOUHSC_03015 |  |  |  |  |  |  |
|  | | | | | | |
| **Protein biosynthesis** | | | | | | |
| SAOUHSC_00520 | *rplJ* | 50S ribosomal protein L10 |  |  | **3.8** | 2.08E-11 |
| SAOUHSC_00521 | *rplL* | 50S ribosomal protein L7/L12 |  |  | **3.3** | 3.84E-09 |
|  | | | | | | |
| **Biosynthesis of cofactors** | | | | | | |
| SAOUHSC_00171 | *ggt* | Gamma-glutamyltranspeptidase |  |  | **5.1** | 2.32E-17 |
| SAOUHSC_00284 |  | 5'-nucleotidase | **3.4** | 3.25E-33 |  |  |
| SAOUHSC_00827 |  | Alpha-ribazole phosphatase | **5.8** | 8.96E-53 |  |  |
| **DNA metabolism** | | | | | | |
| SAOUHSC_00734 |  | Putative 5'(3')-deoxyribonucleotidase |  |  | **3.7** | 8.95E-11 |
| SAOUHSC_01744 | *recJ* | Single-stranded-DNA-specific exonuclease |  |  | **3.1** | 1.73E-08 |

| SAOUHSC_02911 *queH* Adenine nucleotide alpha hydrolases superfamily protein **3.3** 9.77E-32 | | | | | | |
| --- | --- | --- | --- | --- | --- | --- |
| **Energy metabolism** | | | | | | |
| SAOUHSC_00088 | *galE* | UDP-glucose 4-epimerase | **75.5** | 3.27E-239 | **43.0** | 2.25E-71 |
| SAOUHSC_00089 |  | Undecaprenyl-phosphate glucose phosphotransferase | **76.6** | 6.94E-223 | **26.9** | 8.71E-56 |
| SAOUHSC_00555 |  | Haloacid dehalogenase-like hydrolase | **15.2** | 4.18E-136 | **7.0** | 2.12E-23 |
| SAOUHSC_01450 |  | Amino acid transporter | **15.3** | 4.58E-140 | **28.1** | 1.31E-55 |
| SAOUHSC_01451 | *ilvA1* | Threonine dehydratase | **16.3** | 2.58E-144 | **32.0** | 1.07E-59 |
| SAOUHSC_01452 | *ald1* | Alanine dehydrogenase | **16.7** | 5.29E-147 | **38.3** | 9.21E-64 |
| SAOUHSC_01835 |  | Hypothetical protein |  |  | **4.8** | 3.37E-14 |
| SAOUHSC_02922 | *ldh2* | L-lactate dehydrogenase | **3.9** | 2.62E-41 | **4.6** | 4.89E-13 |
| **Carbohydrates** | | | | | | |
| SAOUHSC_00113 | *adhE* | Bifunctional acetaldehyde-CoA/alcohol deshydrogenase | **5.5** | 4.86E-62 |  |  |
| SAOUHSC_00190 |  | Glycerophosphoryl diester phosphodiesterase, periplasmic |  |  | **7.4** | 1.72E-24 |
|  | | | | | | |
| **Amino acids and derivates** | | | | | | |
| SAOUHSC_00435 | *gltB* | Glutamate synthase large subunit |  |  | **37.8** | 1.38E-61 |
| SAOUHSC_00436 | *gltD* | Glutamate synthase subunit beta |  |  | **48.1** | 7.01E-69 |
| SAOUHSC_00989 | *patA* | LL-diaminopimelate aminotransferase |  |  | **4.8** | 4.97E-14 |
|  | | | | | | |
| **Fatty acid and phospholipid metabolism** | | | | | | |
| SAOUHSC_00300 | *geh* | Lipase | **5.2** | 4.78E-59 |  |  |
| SAOUHSC_00661 |  | Esterase |  |  | **3.2** | 4.26E-09 |
|  | | | | | | |
| **Fructose metabolism** | | | | | | |
| SAOUHSC_00706 | *fruR* | Transcriptional repressor of the fructose operon |  |  | **41.9** | 4.49E-64 |
| SAOUHSC_00707 | *fruB* | 1-phosphofructokinase |  |  | **42.1** | 4.30E-65 |
| SAOUHSC_00708 | *fruA* | Fructose specific permease |  |  | **38.0** | 9.34E-62 |
|  | | | | | | |
| **Central intermediary metabolism** | | | | | | |
| SAOUHSC_01972 | *prsA* | Protein export protein | **4.0** | 7.52E-44 | **3.8** | 3.54E-10 |
| SAOUHSC_02558 | *ureA* | Urease subunit gamma |  |  | **4.4** | 2.76E-12 |
| SAOUHSC_02559 | *ureB* | Urease subunit beta |  |  | **3.4** | 6.12E-09 |
| SAOUHSC_02561 | *ureC* | Urease subunit alpha |  |  | **3.7** | 1.93E-11 |
| SAOUHSC_02924 |  | 4-aminobutyrate aminotransferase |  |  | **15.5** | 8.43E-43 |

| **Cell envelope & capsule** | | | | | | |
| --- | --- | --- | --- | --- | --- | --- |
| SAOUHSC_00090 |  | Exopolysaccharide biosynthesis glycosyltransferase | **43.2** | 1.61E-204 | **10.6** | 1.00E-32 |
| SAOUHSC_00092 |  | Membrane protein involved in the export of O-antigen, teichoic acid lipoteichoic acids | **7.0** | 3.80E-78 |  |  |
| SAOUHSC_00114 | *capA* | Capsular polysaccharide biosynthesis protein | **5.2** | 5.81E-19 |  |  |
| SAOUHSC_00115 | *capB* | Capsular polysaccharide biosynthesis protein | **5.3** | 1.43E-18 |  |  |
| SAOUHSC_00116 | *capC* | Capsular polysaccharide biosynthesis protein | **4.3** | 2.02E-15 |  |  |
| SAOUHSC_00117 | *capD* | Capsular polysaccharide biosynthesis protein | **6.8** | 2.26E-42 |  |  |
| SAOUHSC_00118 | *capE* | Capsular polysaccharide biosynthesis protein | **3.7** | 1.69E-16 | **3.2** | 3.34E-08 |
| SAOUHSC_00119 | *capF* | Capsular polysaccharide biosynthesis protein | **3.5** | 2.73E-16 |  |  |
| SAOUHSC_00255 |  | LPXTG cell wall anchor domain | **7.5** | 5.18E-65 | **8.9** | 4.90E-26 |
| SAOUHSC_02404 | *fmtB* | SasC/Mrp/FmtB intercellular aggregation domain | **3.1** | 4.96E-30 | **6.9** | 3.67E-21 |
| SAOUHSC_02576 | *ssaA* | Secretory antigen | **5.5** | 1.10E-60 | **6.7** | 8.97E-22 |
| SAOUHSC_02855 |  | LysM domain-containing protein |  |  | **4.1** | 1.33E-12 |
| SAOUHSC_02998 | *cap1C* | Capsular polysaccharide biosynthesis protein Cap5C | **90.6** | 6.08E-267 | **26.5** | 6.55E-57 |
| SAOUHSC_02999 | *capB1* | Capsular polysaccharide biosynthesis protein Cap5B | **152.0** | 6.14E-279 | **42.4** | 3.30E-67 |
| SAOUHSC_03000 | *capA1* | Capsular polysaccharide biosynthesis protein CapA | **213.9** | 2.24E-275 | **56.6** | 6.64E-73 |
|  | | | | | | |
| **Cellular processes** | | | | | | |
| SAOUHSC_00051 | *plc* | 1-phosphatidylinositol phosphodiesterase | **12.2** | 2.63E-114 | **23.6** | 5.13E-49 |
| SAOUHSC_00427 | *sle1* | Autolysin |  |  | **5.5** | 2.18E-16 |
| SAOUHSC_00721 | *queC* | 7-cyano-7-deazaguanine synthase QueC |  |  | **5.6** | 1.46E-16 |
| SAOUHSC_01219 | *lytN* | Cell wall hydrolase | **5.7** | 2.44E-47 | **7.2** | 1.03E-23 |
| SAOUHSC_01220 | *fmhC* | FemAB-related protein | **3.4** | 1.12E-27 | **6.4** | 3.63E-21 |
| SAOUHSC_02941 | *nrdG* | Anaerobic ribonucleoside-triphosphate reductase activating protein |  |  | **12.2** | 1.70E-33 |
| SAOUHSC_02942 | *nrdR* | Anaerobic ribonucleoside triphosphate reductase |  |  | **8.6** | 1.87E-24 |
| SAOUHSC_02973 |  | Cell division protein ZipA | **5.7** | 3.71E-45 |  |  |
| SAOUHSC_02979 |  | N-acetylmuramoyl-L-alanine amidase |  |  | **3.2** | 1.01E-07 |
| SAOUHSC_03016 |  | Polysaccharide deacetylase | **10.1** | 4.23E-100 | **7.1** | 1.39E-23 |
| SAOUHSC_03017 |  | N-acetyltransferase |  |  | **8.2** | 3.09E-25 |
| **Lipoproteins** | | | | | | |
| SAOUHSC_00052 | *csaA1* | Staphylococcus tandem lipoproteins | **16.4** | 3.91E-53 | **8.7** | 1.32E-22 |
| SAOUHSC_00053 |  | Staphylococcus tandem lipoproteins | **7.2** | 1.09E-34 | **6.5** | 7.91E-18 |
| SAOUHSC_00054 |  | Staphylococcus tandem lipoproteins | **4.8** | 4.07E-29 | **6.0** | 1.38E-17 |
| SAOUHSC_00055 |  | Staphylococcus tandem lipoproteins | **3.9** | 5.38E-26 | **4.1** | 4.07E-12 |
|  | | | | | | |
| **Regulatory functions** | | | | | | |
| SAOUHSC_00674 | *sarX* | Staphylococcal accessory regulator family | **4.1** | 7.20E-44 | **3.7** | 3.29E-09 |
| SAOUHSC_00694 | *mgrA* | Staphylococcal accessory regulator family |  |  | **3.8** | 6.70E-10 |
| SAOUHSC_00818 | *nuc* | Thermonuclease | **130.1** | 4.46E-277 | **269.4** | 4.52E-115 |
| SAOUHSC_00913 | *lysR* | LysR family regulatory protein | **65.1** | 5.18E-208 | **42.8** | 2.02E-71 |
| SAOUHSC_00992 | *atlR* | MarR family transcriptional regulator | **17.3** | 8.53E-136 | **28.7** | 1.53E-54 |

| SAOUHSC_01402 *msa* | | Protein msa (Modulator of *sarA* ) | **4.8** | 3.48E-46 | **3.4** | 1.32E-09 |
| --- | --- | --- | --- | --- | --- | --- |
| SAOUHSC_02569  SAOUHSC_02570 | *sarY* | Staphylococcal accessory regulator family  AraC family transcriptional regulator | **8.8**  **11.4** | 8.35E-80  1.21E-112 | **5.6**  **6.0** | 2.82E-16  2.74E-20 |
|  | | | | | | |
| **Virulence** | | | | | | |
| SAOUHSC_00061 |  | Myosin-cross-reactive antigen | **11.3** | 1.58E-116 | **21.5** | 1.86E-46 |
| SAOUHSC_00069 | *spa* | Protein A |  |  | **3.2** | 3.98E-08 |
| SAOUHSC_00544 | *sdrC* | Fibrinogen-binding protein SdrC | **6.8** | 1.52E-73 | **5.4** | 8.24E-17 |
| SAOUHSC_00545 | *sdrD* | Fibrinogen-binding protein SdrD | **8.0** | 4.39E-91 | **5.2** | 7.44E-15 |
| SAOUHSC_00968 |  | Bacteriocin-associated integral membrane protein |  |  | **5.0** | 1.33E-15 |
| SAOUHSC_01121 | *hla* | Alpha-hemolysin | **5.6** | 3.44E-39 |  |  |
| SAOUHSC_01448 | *norB* | Quinolone resistance protein | **13.3** | 3.53E-127 | **24.5** | 1.66E-55 |
| SAOUHSC_01954 | *lukD* | Leukotoxin LukD | **6.4** | 5.27E-30 | **10.1** | 3.63E-27 |
| SAOUHSC_01955 | *lukE* | Leukotoxin LukE | **9.5** | 1.76E-38 | **11.6** | 3.00E-29 |
| SAOUHSC_02127 | *scpA* | Staphopain thiol proteinase | **126.7** | 0.00E+00 | **57.2** | 2.25E-71 |
| SAOUHSC_02129 |  | Staphostatin A | **65.3** | 5.34E-258 | **51.5** | 2.25E-71 |
| SAOUHSC_02167 | *scn* | Staphylococcal complement inhibitor SCIN | **3.3** | 7.36E-33 | **5.6** | 6.14E-17 |
| SAOUHSC_02171 | *sak* | Staphylokinase | **26.7** | 5.60E-186 | **30.1** | 7.57E-59 |
| SAOUHSC_02169 | *chp* | Chemotaxis-inhibiting protein CHIPS | **16.6** | 1.45E-98 | **4.2** | 9.39E-10 |
| SAOUHSC_02241 | *lukG* | Leukocidin LukG | **6.0** | 4.85E-67 | **5.7** | 4.99E-18 |
| SAOUHSC_02243 | *lukH* | Leukocidin LukH | **6.9** | 5.09E-75 | **5.9** | 2.85E-19 |
| SAOUHSC_02463 | *hysA* | Hyaluronate lyase | **8.0** | 2.48E-84 | **3.8** | 6.20E-12 |
| SAOUHSC_02611 | *lyrA* | Lysostaphin resistance protein A |  |  | **3.3** | 1.96E-08 |
| SAOUHSC_02696 | *fmhA* | Methicillin resistance determinant protein (FemAB family) | **38.9** | 1.01E-198 | **21.0** | 2.98E-51 |
| SAOUHSC_02706 | *sbi* | Immunoglobulin G-binding protein Sbi | **9.3** | 1.75E-98 | **13.3** | 6.55E-39 |
| SAOUHSC_02709 | *hlgC* | Leukocidin s subunit |  |  | **11.2** | 2.96E-32 |
| SAOUHSC_02710 | *hlgB* | Leukocidin f subunit |  |  | **9.8** | 1.27E-28 |
| SAOUHSC_02740 |  | Drug resistance MFS transporter, drug:H+ antiporter-2 |  |  | **3.0** | 2.75E-07 |
| SAOUHSC_02851 | *cidA* | Holin-like protein CidA |  |  | **4.1** | 7.94E-13 |
| SAOUHSC_02883 | *ssaA* | Secretory antigen SsaA (LysM domain-containing protein) | **4.7** | 1.33E-50 | **4.2** | 4.44E-14 |
| SAOUHSC_02963 | *clfB* | Clumping factor B |  |  | **5.6** | 7.01E-17 |
| SAOUHSC_02971 | *aur* | Zinc metalloproteinase aureolysin | **467.8** | 0.00E+00 | **162.0** | 4.69E-103 |
|  | | | | | | |
| **Biofilm** | | | | | | |
| SAOUHSC_03002 | *icaA* | N-glycosyltransferase |  |  | **59.4** | 7.71E-60 |
| SAOUHSC_03003 | *icaD* | Intracellular adhesion protein D |  |  | **21.7** | 3.66E-14 |
| SAOUHSC_03004 | *icaB* | Intercellular adhesion protein B |  |  | **15.5** | 1.65E-25 |
| SAOUHSC_03005 | *icaC* | Intercellular adhesion protein C |  |  | **4.6** | 2.10E-12 |
|  | | | | | | |
| **Stress response** | | | | | | |
| SAOUHSC_00093 | *sodM* | Superoxide dismutase | **14.0** | 1.15E-132 | **4.5** | 3.59E-13 |
| SAOUHSC_02949 | *gpxA2* | Putative glutathione peroxidase | **6.3** | 1.77E-61 | **8.0** | 3.35E-26 |
|  | | | | | | |

|  | | | | | | |
| --- | --- | --- | --- | --- | --- | --- |
| **Iron acquisition and metabolism** | | | | | | |
| SAOUHSC_00074 | *sirA* | Heme ABC transporter | **3.2** | 1.02E-26 |  |  |
| SAOUHSC_00976 |  | Heme ABC transporter |  |  | **3.0** | 5.42E-08 |
| SAOUHSC_01079 | *isdB* | Cell surface receptor IsdB for hemoglobin and hemoglobin-haptoglobin complexes | **5.8** | 1.54E-37 | **3.5** | 8.79E-09 |
| SAOUHSC_02640 | *hrtA* | Heme efflux system ATPase |  |  | **3.8** | 1.53E-11 |
|  | | | | | | |
| **Competence** | | | | | | |
| SAOUHSC_00961 | *comK1* | Competence transcription factor | **15.1** | 3.81E-60 | **9.4** | 2.53E-27 |
| **sRNA** | SRD data  Description | Length (nucleotides) |  |  |  |  |
| *srn_0050_teg1* | Transcript | 229 |  |  | **4.2** | 7.59E-12 |
| *srn_0280_sRNA16* | ND | 372 |  |  | **4.0** | 2.81E-12 |
| *srn_0730_sRNA48* | ND | 256 | **5.3** | 2.98E-06 |  |  |
| *srn_0765_tsr11* | 5'UTR | 94 |  |  | **3.3** | 2.03E-08 |
| *srn_0790_sau6657* | ND | 91 | **4.7** | 6.55E-06 |  |  |
| *srn_0795* | Transcript | 279 | **4.6** | 4.20E-32 |  |  |
| *srn_0860_rsaOB* | Transcript | 421 |  |  | **10.5** | 7.34E-29 |
| *srn_0930_teg76* | Transcript | 223 |  |  | **3.8** | 7.87E-11 |
| *srn_1100_sau6054* | 3'UTR | 143 |  |  | **4.2** | 2.77E-08 |
| *srn_1440_sRNA127* | ND | 32 |  |  | **3.0** | 2.59E-03 |
| *srn_1480_sRNA130* | ND | 603 | **4.0** | 1.72E-03 |  |  |
| *srn_1530_sRNA133* | 5'UTR | 272 | **3.1** | 8.20E-29 |  |  |
| *srn_1640_rsaD* | Transcript | 177 |  |  | **10.4** | 3.05E-27 |
| *srn_1760_sRNA149* | Transcript | 127 |  |  | **11.6** | 2.65E-13 |
| *srn_1870_sau6428* | 5'UTR | 55 |  |  | **3.8** | 2.19E-02 |
| *srn_2230_sprG2* | Transcript | 241 | **15.5** | 1.16E-143 | **9.0** | 1.94E-25 |
| *srn_2250_sprG4* | Transcript | 369 | **9.8** | 2.19E-08 |  |  |
| *srn_2730_teg106* | 3'UTR | 46 | **3.6** | 1.35E-08 | **3.1** | 2.71E-05 |
| *srn_2740_teg108* | Transcript | 103 | **5.4** | 1.94E-05 |  |  |
| *srn_2780_sau6282* | 5'UTR | 70 |  |  | **10.3** | 2.26E-04 |
| *srn_2800_sau14* | ND | 99 |  |  | **33.5** | 1.10E-28 |
| *srn_2890_sau72* | ND | 125 |  |  | **5.4** | 2.17E-05 |
| *srn_3130_teg116* | ND | 55 |  |  | **3.2** | 8.39E-03 |
| *srn_3340_teg72* | 5'UTR | 148 | **5.6** | 2.62E-24 |  |  |
| *srn_3500_sRNA277* | 5'UTR | 244 |  |  | **3.2** | 1.30E-08 |
| *srn_3610_sprC* | Transcript | 154 | **7.1** | 4.72E-64 | **8.9** | 6.84E-28 |
| *srn_3630_sau69* | 5'UTR | 98 | **4.3** | 3.68E-06 | **3.5** | 3.03E-03 |
| *srn_3950_teg16* | Transcript | 225 |  |  | **21.8** | 4.41E-44 |
| *srn_4500_teg127* | ND | 37 |  |  | **5.8** | 1.87E-02 |
| *srn_4666_tsr36* | CDS | 526 |  |  | **3.5** | 6.87E-10 |
| *srn_4680_sau19* | Transcript | 75 |  |  | **4.6** | 3.85E-05 |
| *srn_4970_sRNA398* | ND | 144 | **7.4** | 6.67E-36 | **8.8** | 3.07E-23 |
| *srn_4980_teg32* | ND | 224 | **7.9** | 1.62E-21 | **12.0** | 1.10E-34 |

| *srn_5040_sRNA403* | ND |  | 138 |  |  | **5.1** | 3.11E-05 |
| --- | --- | --- | --- | --- | --- | --- | --- |
| *srn_9320_sRNA258* | Transcript |  | 36 |  |  | **3.5** | 4.58E-05 |
| *srn_9335_tsr29* | Transcript |  | 131 | **3.1** | 9.40E-09 | **5.5** | 5.57E-15 |
| *srn_9340_sRNA287* | Transcript |  | 369 | **13.9** | 1.20E-50 | **24.8** | 1.66E-52 |
|  | | | | | | | |
| **Unknown function** | | | | | | | |
| SAOUHSC_00034 | *cstR* | Hypothetical protein |  |  |  | **3.3** | 7.67E-05 |
| SAOUHSC_00047 |  | Hypothetical protein |  |  |  | **3.5** | 3.74E-09 |
| SAOUHSC_00084 |  | Hypothetical protein |  | **3.4** | 5.09E-18 |  |  |
| SAOUHSC_00091 |  | Hypothetical protein |  | **28.5** | 3.37E-176 | **5.7** | 1.26E-16 |
| SAOUHSC_00174 |  | Hypothetical protein |  |  |  | **4.4** | 1.98E-09 |
| SAOUHSC_00182 |  | Hypothetical protein |  | **3.2** | 7.15E-23 |  |  |
| SAOUHSC_00254 |  | Hypothetical protein |  | **11.0** | 2.12E-76 | **10.4** | 1.71E-28 |
| SAOUHSC_00256 |  | Hypothetical protein |  | **7.2** | 6.77E-65 | **3.8** | 1.32E-11 |
| SAOUHSC_00330 |  | Hypothetical protein |  |  |  | **6.1** | 4.85E-20 |
| SAOUHSC_00381a |  | Hypothetical protein |  | **3.3** | 8.51E-06 | **5.0** | 1.72E-12 |
| SAOUHSC_00716 | *saeQ* | Hypothetical protein |  |  |  | **3.3** | 9.13E-09 |
| SAOUHSC_00737 |  | Hypothetical protein |  |  |  | **3.8** | 3.61E-03 |
| SAOUHSC_00774 |  | Hypothetical protein |  |  |  | **3.1** | 1.76E-08 |
| SAOUHSC_00807 |  | Hypothetical protein |  | **3.3** | 1.62E-21 |  |  |
| SAOUHSC_00808 |  | Hypothetical protein |  | **3.7** | 3.17E-37 |  |  |
| SAOUHSC_00863 |  | Hypothetical protein |  |  |  | **3.4** | 6.09E-09 |
| SAOUHSC_00911 |  | Hypothetical protein |  | **4.8** | 3.40E-45 | **3.3** | 7.21E-09 |
| SAOUHSC_00967 |  | Hypothetical protein |  | **3.3** | 1.79E-03 | **21.5** | 1.35E-21 |
| SAOUHSC_00975 |  | Hypothetical protein |  | **4.7** | 4.74E-53 | **26.6** | 1.94E-57 |
| SAOUHSC_01005 |  | Hypothetical protein |  |  |  | **4.8** | 1.41E-13 |
| SAOUHSC_01023 |  | Hypothetical protein |  |  |  | **3.1** | 1.01E-02 |
| SAOUHSC_01113 |  | Hypothetical protein |  | **4.2** | 9.08E-40 |  |  |
| SAOUHSC_01289 |  | Hypothetical protein |  | **23.4** | 6.16E-62 | **13.1** | 4.07E-33 |
| SAOUHSC_01290 |  | Hypothetical protein |  | **5.5** | 8.64E-36 | **4.2** | 8.29E-13 |
| SAOUHSC_01291 |  | Hypothetical protein |  | **4.8** | 4.08E-26 | **3.5** | 3.97E-09 |
| SAOUHSC_01292 |  | Hypothetical protein |  | **4.4** | 1.59E-24 |  |  |
| SAOUHSC_01295 |  | Hypothetical protein |  | **4.3** | 1.26E-07 | **3.6** | 1.88E-04 |
| SAOUHSC_01584 |  | Hypothetical protein |  | **3.1** | 4.59E-11 |  |  |
| SAOUHSC_01798 |  | Hypothetical protein |  | **4.9** | 2.48E-56 |  |  |
| SAOUHSC_01851 |  | Hypothetical protein |  |  |  | **4.4** | 8.34E-08 |
| SAOUHSC_01899 |  | Hypothetical protein |  |  |  | **3.7** | 1.25E-10 |
| SAOUHSC_01917 |  | Hypothetical protein |  | **6.3** | 4.48E-28 | **5.4** | 6.32E-11 |
| SAOUHSC_01923 |  | Hypothetical protein |  | **6.2** | 4.86E-57 | **4.0** | 5.84E-12 |
| SAOUHSC_01944 |  | Hypothetical protein |  |  |  | **9.4** | 1.32E-22 |
| SAOUHSC_02104 |  | Hypothetical protein |  |  |  | **3.3** | 1.45E-05 |
| SAOUHSC_02782 |  | Hypothetical protein |  |  |  | **5.9** | 1.20E-06 |
| SAOUHSC_02856 |  | Hypothetical protein |  |  |  | **9.2** | 2.66E-26 |
| SAOUHSC_02857 |  | Hypothetical protein |  |  |  | **8.5** | 2.74E-20 |
| SAOUHSC_02858 |  | Hypothetical protein |  |  |  | **9.3** | 2.47E-27 |
| SAOUHSC_02888 |  | Hypothetical protein |  |  |  | **32.2** | 1.95E-64 |

| SAOUHSC_02950 |  | Hypothetical protein |  |  | **11.3** | 8.62E-31 |
| --- | --- | --- | --- | --- | --- | --- |
| **Gene group and annotation** | **Gene name** | **Assignment** | **Differential expression HG003 *ΔsarA* vs HG003** | | | |
|  |  |  | **Exponential (2h)** | | **Early stationnary (4.5h)** | |
|  |  |  | **Fold-change** | **p-value** | **Fold-change** | **p-value** |
| **Genes actived by SarA** | | | | | | |
| **Transport and binding proteins & protein fate** | | | | | | |
| SAOUHSC_00201 | *opp-5A* | Nickel ABC transporter | **3.5** | 7.26E-35 |  |  |
| SAOUHSC_00281 |  | Formate/nitrite transporter | **3.6** | 7.04E-37 |  |  |
| SAOUHSC_00310 | *ulaA* | PTS system ascorbate-specific transporter |  |  | **30.7** | 2.83E-34 |
| SAOUHSC_00311 |  | PTS EIIB type-2 domain-containing protein |  |  | **32.7** | 1.43E-38 |
| SAOUHSC_00312 |  | PTS system, fructose subfamily, IIA component |  |  | **21.1** | 3.65E-32 |
| SAOUHSC_00313 |  | PTS system, fructose subfamily, IIA component |  |  | **10.9** | 1.04E-20 |
| SAOUHSC_01861 |  | Glutamyl aminopeptidase |  |  | **3.2** | 1.56E-06 |
| SAOUHSC_01945 | *epiG* | Lantibiotic protection ABC transporter permease subunit, MutG family |  |  | **3.5** | 1.62E-07 |
| SAOUHSC_01947 | *epiE* | Lantibiotic protection ABC transporter permease subunit, MutE/EpiE family |  |  | **4.0** | 7.71E-09 |
| SAOUHSC_01948 | *epiF* | Lantibiotic protection ABC transporter, ATP-binding subunit |  |  | **3.9** | 1.67E-08 |
| SAOUHSC_02153 | *pmtB* | ABC-2 transporter family protein |  |  | **7.5** | 1.82E-16 |
| SAOUHSC_02154 | *pmtA* | ABC transporter ATP-binding protein |  |  | **8.3** | 3.56E-17 |
| SAOUHSC_02155 | *pmtR* | Histidine utilization repressor |  |  | **7.7** | 3.48E-17 |
| SAOUHSC_02597 | *glvC* | PTS system transporter |  |  | **4.3** | 1.66E-09 |
| SAOUHSC_02671 | *narK* | Nitrite transporter | **8.5** | 2.03E-94 |  |  |
| SAOUHSC_02864 | *feoB* | Ferrous iron transport protein B | **3.0** | 1.00E-14 |  |  |
| SAOUHSC_02905 |  | Type II secretion protein |  |  | **5.1** | 1.30E-08 |
| **Energy metabolism** | | | | | | |
| SAOUHSC_00203 |  | Hypothetical protein | **3.7** | 7.61E-39 |  |  |
| SAOUHSC_00204 | *hmp* | Globin domain-containing protein | **3.6** | 5.82E-38 |  |  |
| SAOUHSC_00535 |  | UDP-glucose 4-epimerase |  |  | **4.8** | 1.59E-10 |
| SAOUHSC_00894 | *rocD* | Ornithine-oxo-acid transminase |  |  | **3.3** | 9.21E-07 |
| SAOUHSC_01007 | *folD* | 5,10-methylene-tetrahydrofolate cyclohydrolase |  |  | **3.9** | 5.09E-08 |
| SAOUHSC_01216 | *sucC* | Succinyl CoA synthetase |  |  | **3.0** | 9.52E-06 |
| SAOUHSC_01794 | *gapB* | Glyceraldehyde 3-phosphate deshydrogenase |  |  | **3.7** | 9.36E-08 |
| SAOUHSC_01801 | *citC* | Isocitrate deshydrogenase |  |  | **3.4** | 9.21E-07 |
| SAOUHSC_01802 | *citZ* | Citrate synthase |  |  | **3.2** | 1.05E-06 |
| SAOUHSC_01818 | *ald2* | Alanine deshydrogenase |  |  | **7.3** | 1.20E-14 |
| SAOUHSC_01910 | *pckA* | Phosphoenolpyruvate carboxykinase |  |  | **4.1** | 1.08E-08 |
| SAOUHSC_02151 | *pmtD* | Hypothetical protein |  |  | **5.0** | 8.74E-11 |
| SAOUHSC_02152 | *pmtC* | ABC transporter ATP-binding protein |  |  | **6.2** | 3.48E-13 |
| SAOUHSC_02379 | *deoC2* | Aldolase |  |  | **4.4** | 3.49E-09 |
| SAOUHSC_02467 | *budA1* | Alpha-acetolactate decarboxylase | **22.3** | 4.29E-166 |  |  |
| SAOUHSC_02468 | *budB* | Acetolactate synthase | **20.8** | 3.74E-165 |  |  |
| SAOUHSC_02606 | *hutl* | Imidazolonepropionase |  |  | **13.7** | 4.02E-23 |
| SAOUHSC_02607 | *hutU* | Urocanate hydratase |  |  | **17.9** | 1.61E-26 |
| SAOUHSC_02647 | *mqo1* | Malate quinone oxidoreductase |  |  | **3.2** | 2.58E-06 |
| SAOUHSC_02675 | *nreC* | Oxygen regulatory protein | **6.5** | 7.59E-73 |  |  |

| SAOUHSC_02676 | *nreB* | Oxygen sensor histidine kinase | **6.7** | 6.66E-76 |  |  |
| --- | --- | --- | --- | --- | --- | --- |
| SAOUHSC_02677 | *nreA* | Nif-specific regulatory protein | **7.1** | 3.43E-79 |  |  |
| SAOUHSC_02678 | *narI* | Respiratory nitrate reductase subunit gamma | **9.6** | 9.80E-102 |  |  |
| SAOUHSC_02679 | *narJ* | Respiratory nitrate reductase subunit delta | **10.0** | 2.56E-103 |  |  |
| SAOUHSC_02680 | *narH* | Nitrate reductase subunit beta | **8.1** | 1.73E-90 |  |  |
| SAOUHSC_02681 | *narG* | Nitrate reductase subunit alpha | **4.8** | 1.62E-55 |  |  |
| SAOUHSC_02839 | *sdaAA* | L-serine dehydratase |  |  | **3.1** | 1.97E-06 |
| SAOUHSC_02840 | *sdaAB* | L-serine dehydratase iron-sulfur-dependent subunit beta |  |  | **6.8** | 4.40E-13 |
| SAOUHSC_02841 |  | Hypothetical protein |  |  | **9.2** | 6.03E-19 |
| SAOUHSC_02869 | *rocA* | Pyrroline-5-carboxylate dehydrogenase |  |  | **4.9** | 4.94E-11 |
| SAOUHSC_02975 | *manP* | PTS system fructose-specific transporter |  |  | **3.6** | 1.17E-07 |
| SAOUHSC_02976 | *pmi* | Mannose-6-phosphate isomerase |  |  | **4.1** | 6.59E-09 |
|  | | | | | | |
| **Amino acids** | | | | | | |
| SAOUHSC_00144 | *ausA* | Non-ribosomal peptide synthetase | **3.3** | 6.00E-32 |  |  |
| SAOUHSC_01884 | *putA* | Proline dehydrogenase |  |  | **3.3** | 4.12E-07 |
|  | | | | | | |
| **Nucleotide metabolism** | | | | | | |
| SAOUHSC_00019 | *purA* | Adenylosuccinate synthetase |  |  | **4.4** | 1.98E-09 |
| SAOUHSC_01008 | *purE* | 5-(carboxyamino)imidazole ribonucleotide mutase |  |  | **4.0** | 2.29E-08 |
| SAOUHSC_01009 | *purK* | 5-(carboxyamino)imidazole ribonucleotide mutase |  |  | **4.0** | 1.44E-08 |
| SAOUHSC_01010 | *purC* | Phosphoribosylaminoimidazole-succinocarboxamide synthase |  |  | **3.6** | 2.12E-07 |
| SAOUHSC_01011 | *purS* | Phosphoribosylformylglycinamidine synthase |  |  | **3.9** | 1.34E-06 |
| SAOUHSC_01012 | *purQ* | Phosphoribosylformylglycinamidine synthase |  |  | **4.2** | 4.42E-09 |
| SAOUHSC_01165 | *pyrP* | Uracil permease |  |  | **7.4** | 6.37E-14 |
| SAOUHSC_01166 | *pyrB* | Aspartate carbamoyltransferase catalytic subunit |  |  | **7.7** | 1.89E-13 |
| SAOUHSC_01168 | *pyrC* | Dihydroorotase |  |  | **5.2** | 2.65E-11 |
| SAOUHSC_01169 | *carA* | Carbamoyl phosphate synthase small subunit |  |  | **3.3** | 1.07E-06 |
| SAOUHSC_02377 | *pdp* | Pyrimidine-nucleoside phosphorylase |  |  | **4.2** | 1.21E-08 |
|  | | | | | | |
| **Carbohydrates** | | | | | | |
| SAOUHSC_00712 |  | Aldo/keto reductase |  |  | **3.2** | 2.97E-06 |
| SAOUHSC_01845 | *fhs* | Formate-tetrahydrofolate ligase |  |  | **4.8** | 2.21E-10 |
|  | | | | | | |
| **Fatty acid and phospholipids metabolism** | | | | | | |
| SAOUHSC_00195 | *fadA* | Acetyl-CoA acetyltransferase |  |  | **17.7** | 6.65E-28 |
| SAOUHSC_00196 | *fadB* | Fatty acid oxidation complex, alpha subunit |  |  | **11.2** | 2.53E-21 |
| SAOUHSC_00197 | *fadD* | Acyl-CoA dehydrogenase |  |  | **7.3** | 3.01E-16 |
| SAOUHSC_00198 | *fadE* | O-succinylbenzoate-CoA ligase |  |  | **4.4** | 5.16E-10 |
| SAOUHSC_02877 | *crtN* | Squalene synthase |  |  | **3.1** | 5.12E-06 |
| SAOUHSC_02879 | *crtM* | Squalene desaturase |  |  | **3.5** | 2.15E-07 |
| SAOUHSC_02880 | *crtQ* | 4,4'-diaponeurosporenoate glycosyltransferase |  |  | **3.1** | 5.92E-06 |
| SAOUHSC_03006 | *gehA* | Lipase |  |  | **3.1** | 9.33E-07 |

|  | | | | | | |
| --- | --- | --- | --- | --- | --- | --- |
| **Central intermediary metabolism** | | | | | | |
| SAOUHSC_00577 | *mvaK1* | Mevalonate kinase |  |  | **3.0** | 1.04E-05 |
| SAOUHSC_02682 | *nasF* | Uroporphyrin-III C-methyltransferase | **6.0** | 1.52E-68 |  |  |
| SAOUHSC_02683 | *nasE* | Assimilatory nitrite reductase [NAD(P)H] small subunit | **5.5** | 8.24E-62 |  |  |
| SAOUHSC_02684 | *nasD* | Assimilatory nitrite reductase [NAD(P)H] large subunit | **4.5** | 3.76E-50 |  |  |
| SAOUHSC_02685 | *nirR* | Hypothetical protein | **3.7** | 9.05E-39 |  |  |
|  | | | | | | |
| **Cell envelope** | | | | | | |
| SAOUHSC_00156 |  | Hypothetical protein |  |  | **3.8** | 1.09E-08 |
| SAOUHSC_00157 | *murQ* | Estherase |  |  | **5.0** | 1.01E-11 |
| SAOUHSC_00158 |  | PTS system transporter |  |  | **6.0** | 5.63E-13 |
| SAOUHSC_00160 |  | Hypothetical protein |  |  | **7.7** | 2.90E-16 |
| SAOUHSC_00294 | *nanT* | Hypothetical protein |  |  | **3.9** | 1.72E-09 |
| SAOUHSC_00295 | *nanA* | N-acetylneuraminate lyase |  |  | **3.7** | 6.79E-09 |
|  | | | | | | |
| **Osmotic stress** | | | | | | |
| SAOUHSC_02932 | *betA* | Choline dehydrogenase |  |  | **3.7** | 2.71E-08 |
| SAOUHSC_02933 | *betB* | Betaine aldehyde dehydrogenase |  |  | **5.6** | 7.60E-13 |
|  | | | | | | |
| **Signal transduction** | | | | | | |
| SAOUHSC_01799 | *phoR* | Histidine kinase |  |  | **3.4** | 9.31E-07 |
| **Virulence** | | | | | | |
| SAOUHSC_00232 | *lrgA* | Murein hydrolase regulator LrgA | 8.7 | 3.52E-87 |  |  |
| SAOUHSC_00233 | *lrgB* | Antiholin-like protein | **7.3** | 2.38E-81 | **3.8** | 1.75E-08 |
| SAOUHSC_00812 | *clfA* | Clumping factor |  |  | **3.6** | 1.55E-07 |
| SAOUHSC_01135 | *psmβ1* | Staphylococcus haemolytic protein |  |  | **19.0** | 7.67E-28 |
| SAOUHSC_01136 | *psmβ2* | Staphylococcus haemolytic protein |  |  | **18.1** | 1.50E-26 |
|  | | | | | | |
| **sRNA** | SRD data  Description | Length (nucleotides) |  |  |  |  |
| *srn_0510_rsaG* | Transcript | 194 |  |  | **8.8** | 4.06E-19 |
| *srn_0910_sRNA73* | CDS | 160 | **4.0** | 9.54E-04 |  |  |
| *srn_1070_sau41* | Transcript | 469 |  |  | **25.5** | 8.11E-33 |
| *srn_1550_teg49* | Transcript | 517 | **3.7** | 4.65E-36 | **30.5** | 1.26E-33 |
| *srn_1900_sau15* | Antisense | 92 |  |  | **4.9** | 1.78E-07 |
| *srn_2590_sau46* | 3'UTR | 65 | **3.1** | 5.49E-03 | **9.4** | 1.88E-07 |
| *srn_3760_sau7009* | ND | 50 |  |  | **5.1** | 1.17E-06 |
| *srn_4015_tsr31* | 3'UTR | 72 |  |  | **3.8** | 9.29E-04 |

| *srn_4390_rsaOG* | Transcript |  | 111 |  |  | **8.8** | 1.58E-19 |
| --- | --- | --- | --- | --- | --- | --- | --- |
| *srn_4450_sau6307* | ND |  | 75 |  |  | **3.2** | 7.80E-03 |
| *srn_4470_rsaX28* | Transcript |  | 1177 | **3.0** | 7.62E-28 | **6.2** | 1.97E-13 |
| *srn_4480_teg126* | 5'UTR |  | 91 | **8.5** | 3.55E-48 |  |  |
| *srn_4490_sRNA365* | ND |  | 229 | **4.2** | 3.55E-10 |  |  |
| *srn_4790_sRNA387* | CDS |  | 305 |  |  | **5.7** | 2.72E-13 |
|  | | | | | | | |
| **Phage** | | | | | | | |
| SAOUHSC_02016 |  | Phage protein |  |  |  | **6.3** | 2.32E-11 |
| SAOUHSC_02017 |  | Hypothetical protein |  | **3.8** | 4.08E-06 | **5.4** | 1.52E-06 |
| SAOUHSC_02020 |  | Holin |  |  |  | **3.8** | 9.80E-06 |
| SAOUHSC_02034 |  | Hypothetical protein |  |  |  | **3.4** | 1.28E-05 |
| SAOUHSC_02035 |  | Hypothetical protein |  |  |  | **3.4** | 3.23E-06 |
| SAOUHSC_02036 |  | Phage structural protein |  |  |  | **4.1** | 1.92E-09 |
| SAOUHSC_02037 |  | Hypothetical protein |  |  |  | **3.2** | 7.79E-06 |
| SAOUHSC_02040 |  | Hypothetical protein |  |  |  | **3.6** | 3.90E-06 |
| SAOUHSC_02041 |  | Phi Mu50B-like protein |  |  |  | **3.6** | 1.45E-06 |
| SAOUHSC_02042 |  | Phi Mu50B-like protein |  |  |  | **3.9** | 1.46E-06 |
| SAOUHSC_02043 |  | Phage head protein |  |  |  | **4.0** | 1.40E-09 |
| SAOUHSC_02044 |  | Phage capsid protein |  |  |  | **3.7** | 1.24E-08 |
| SAOUHSC_02061 |  | Phi PVL orf 50-like protein |  |  |  | **3.2** | 2.86E-05 |
| SAOUHSC_02064 |  | Phi ETA orf 25-like protein |  |  |  | **3.2** | 2.69E-05 |
| SAOUHSC_02065 |  | Hypothetical protein |  |  |  | **3.2** | 5.94E-05 |
| SAOUHSC_02066 |  | Hypothetical protein |  |  |  | **3.5** | 7.27E-04 |
| SAOUHSC_02068 |  | Hypothetical protein |  |  |  | **3.1** | 6.68E-06 |
|  | | | | | | | |
| **Unknown function** | | | | | | | |
| SAOUHSC_00134 |  | Hypothetical protein |  | **3.2** | 2.55E-19 | **4.4** | 7.60E-10 |
| SAOUHSC_00181 |  | Hypothetical protein |  |  |  | **3.1** | 4.06E-07 |
| SAOUHSC_00193 |  | Hypothetical protein |  |  |  | **8.6** | 9.55E-12 |
| SAOUHSC_00205 |  | Hypothetical protein |  | **3.1** | 7.31E-05 |  |  |
| SAOUHSC_00413 | *mpsB* | Hypothetical protein |  |  |  | **3.9** | 2.75E-08 |
| SAOUHSC_00414 | *mpsC* | Hypothetical protein |  |  |  | **3.4** | 1.81E-07 |
| SAOUHSC_00617 |  | Hypothetical protein |  |  |  | **3.5** | 4.73E-07 |
| SAOUHSC_00820  SAOUHSC_00821 |  | Hypothetical protein  Hypothetical protein |  |  |  | **4.0**  **3.6** | 6.94E-09  5.84E-08 |
| SAOUHSC_00838 |  | Hypothetical protein |  |  |  | **7.0** | 9.37E-16 |
| SAOUHSC_00839 |  | Hypothetical protein |  |  |  | **5.8** | 7.01E-13 |
| SAOUHSC_00971 |  | Hypothetical protein |  | **5.1** | 9.56E-57 | **5.2** | 6.83E-12 |
| SAOUHSC_01138 |  | Hypothetical protein |  |  |  | **3.4** | 7.24E-07 |
| SAOUHSC_01817 |  | Hypothetical protein |  |  |  | **3.1** | 6.30E-06 |
| SAOUHSC_01918 |  | Calcium-binding protein |  |  |  | **3.3** | 1.47E-06 |
| SAOUHSC_01919 |  | Hypothetical protein |  |  |  | **3.4** | 4.89E-07 |
| SAOUHSC_01956 |  | Pseudogene |  |  |  | **3.5** | 1.61E-06 |
| SAOUHSC_02054 |  | Hypothetical protein |  |  |  | **3.3** | 5.67E-04 |

| SAOUHSC_02701 | Hypothetical protein | **19.3** | 2.77E-20 |
| --- | --- | --- | --- |
| SAOUHSC_02906 | Hypothetical protein | **4.5** | 4.46E-07 |

Table S2 : Complete RNA-Seq data.

Selection shows genes with FPKM higher than 10 with transcriptional variations between HG003 and HG003∆sarA mutant strains higher or equal to three.
