# Supplementary material for: Expanding the Staphylococcus aureus SarA Regulon to Small RNAs
Source: mSystems. 2021 Oct 12;6(5):e00713-21. doi: 10.1128/mSystems.00713-21 (PMC8510525; doi:10.1128/mSystems.00713-21)
Supplement: TABLE S3 [file msystems.00713-21-st003.docx]

| Borderline inclusion | | | | mRNA | sRNA |  | |
| --- | --- | --- | --- | --- | --- | --- | --- |
| **Region with nearby genes** | **Peak Center Length** | **Peak Shape Score** | **P-value** | **Gene Start** | **Gene Stop** | **Gene Direction** | **Gene ID** |
| **34511..34875** | 34737 365 | 40.7 | 0 |  |  |  |  |
| **49224..49505** | 49366 282 | 39.4 | 0 |  |  |  |  |
|  | | | | 49490 | 50092 | + | SAOUHSC_00047 |
| **54054..54353** | 54214 300 | 38.3 | 1.59E-321 |  |  |  |  |
|  | | | | 54255 | 55241 | + | SAOUHSC_00051 |
| **71179..71494** | 71319 316 | 35.5 | 1.31E-276 |  |  |  |  |
|  | | | | 71508 | 73100 | + | SAOUHSC_00067 |
|  |  |  |  | 73429 | 74979 | - | SAOUHSC_00069 |
| **74883..75269** | 75130 387 | 39.4 | 0 |  |  |  |  |
|  | | | | 75400 | 76152 | - | SAOUHSC_00070 |
| **76071..76365** | 76211 295 | 47.4 | 0 |  |  |  |  |
| **90944..91240** | 91102 297 | 34.3 | 4.49E-258 |  |  |  |  |
|  | | | | 91163 | 91570 | + | SAOUHSC_00084 |
| **99281..99564** | 99425 284 | 43.1 | 0 |  |  |  |  |
| 93345..93657 | 93485 313 | 33.2 | 7.09E-242 |  |  |  |  |
|  | | | | 93570 | 94550 | + | SAOUHSC_00088 |
| **100126..100459** | 100320 334 | 34.8 | 3.74E-266 |  |  |  |  |
|  | | | | 100455 | 101054 | + | SAOUHSC_00094 |
|  |  |  |  | 109108 | 110064 | - | SAOUHSC_00105 |
| **110038..110332** | 110178 295 | 43.5 | 0 |  |  |  |  |
|  | | | | 110293 | 111837 | + | SAOUHSC_00106 |
| **114893..115199** | 115033 307 | 40.9 | 0 |  |  |  |  |
| **116271..116563** | 116424 293 | 40.2 | 0 |  |  |  |  |
|  | | | | 116538 | 119147 | + | SAOUHSC_00113 |
| **119287..119570** | 119427 284 | 41.2 | 0 |  |  |  |  |
|  | | | | 119492 | 120160 | + | SAOUHSC_00114 |
| **147066..147350** | 147206 285 | 40.4 | 0 |  |  |  |  |
|  | | | | 147348 | 148598 | + | SAOUHSC_00143 |
| **148505..148804** | 148645 300 | 34.8 | 1.91E-265 |  |  |  |  |
|  | | | | 148741 | 148817 | + | *srn_0380_teg140* |
|  |  |  |  | 149045 | 156220 | +  - | SAOUHSC_00144 |
|  |  |  |  | 211198 | 211542 |  | SAOUHSC_00191 |
| **211530..211948** | 211670 419 | 33.9 | 6.14E-252 |  |  |  |  |
|  | | | | 211732 | 213642 | + | SAOUHSC_00192 |
|  |  |  |  | 262382 | 263380 | - | SAOUHSC_00242 |
| **263568..263855** | 263717 288 | 36.1 | 3.65E-285 |  |  |  |  |
|  | | | | 263760 | 264152 | + | SAOUHSC_00244 |
|  |  |  |  | 271579 | 273102 | - | SAOUHSC_00253 |
| **273010..273353** | 273150 344 | 41.5 | 0 |  |  |  |  |
|  | | | | 273373 | 274026 | + | SAOUHSC_00254 |
|  |  |  |  | 274790 | 275683 | - | SAOUHSC_00256 |
| **275594..275887** | 275734 294 | 37.1 | 2.51E-301 |  |  |  |  |
|  | | | | 275931 | 276224 | + | SAOUHSC_00257 |
| **291428..291728** | 291589 301 | 37.8 | 3.43E-313 |  |  |  |  |
|  | | | | 291701 | 291919 | + | SAOUHSC_00272 |
|  |  |  |  | 294524 | 294648 | - | *srn_9020_sRNA52* |

| **294744..295039** | 294884 | 296 | 42.4 | 0 |
| --- | --- | --- | --- | --- |
|  |  |  |  |  |
| **299073..299374** | 299235 | 302 | 37.0 | 6.50E-300 |
| **313990..314294** | 314155 | 305 | 49.5 | 0 |
| **363674..363966** | 363827 | 293 | 39.9 | 0 |
| **366347..366642** | 366487 | 296 | 36.0 | 1.34E-284 |
| **375221..375535** | 375361 | 315 | 45.8 | 0 |
| **377766..378051** | 377912 | 286 | 38.8 | 0 |
| **385973..386285** | 386113 | 313 | 42.9 | 0 |
| **401878..402291** | 402156 | 414 | 34.6 | 1.49E-262 |
| **405171..405487** | 405311 | 317 | 39.5 | 0 |
| **407002..407304** | 407142 | 303 | 39.0 | 0 |
| **418620..418931** | 418792 | 312 | 34.8 | 1.06E-265 |
| **466486..466775** | 466637 | 290 | 35.5 | 7.67E-277 |
| **482997..483302** | 483137 | 306 | 45.0 | 0 |
| **489603..489889** | 489750 | 287 | 46.9 | 0 |
| **498378..498686** | 498547 | 309 | 40.0 | 0 |
| **499817..500108** | 499957 | 292 | 34.3 | 6.38E-258 |
| **507862..508141** | 508002 | 280 | 35.3 | 5.79E-274 |
| **510679..510971** | 510832 | 293 | 42.7 | 0 |

|  | | | |
| --- | --- | --- | --- |
| 295206 | 295580 | + | SAOUHSC_00279 |
| 297408 | 298685 | - | SAOUHSC_00282 |
| 298600 | 298693 | - | *srn_0765_tsr11* |
|  | | | |
| 299299 | 300189 | + | SAOUHSC_00284 |
| 312595 | 313908 | - | SAOUHSC_00299 |
|  | | | |
| 314326 | 316398 | + | SAOUHSC_00300 |
| 363376 | 363534 | - | SAOUHSC_00353 |
|  | | | |
| 363894 | 364505 | + | SAOUHSC_00354 |
| 366133 | 366396 | - | SAOUHSC_00357 |
|  | | | |
| 366696 | 366947 | + | SAOUHSC_00358 |
|  | | | |
| 377409 | 377816 | - | SAOUHSC_00371 |
|  | | | |
| 378025 | 378247 | + | *srn_0930_teg76* |
| 385923 | 386075 | - | SAOUHSC_00381a |
| 386298 | 386353 | + | *srn_0960_teg147* |
| 402508 | 403185 | + | SAOUHSC_00399 |
| 404819 | 405127 | - | SAOUHSC_00401 |
|  | | | |
| 405465 | 406283 | + | SAOUHSC_00402 |
|  | | | |
| 407345 | 408082 | + | SAOUHSC_00405 |
| 416282 | 418757 | - | *srn_1090_teg19as* |
|  | | | |
| 418921 | 419595 | + | SAOUHSC_00416 |
| 466317 | 466521 | - | *srn_1210_sRNA106* |
|  | | | |
| 466774 | 467622 | + | SAOUHSC_00466 |
| 483226 | 483451 | + | *srn_1270_teg44* |
| 489935 | 490027 | + | SAOUHSC_00492 |
| 498266 | 498442 | - | *srn_1330_sRNA118* |
|  | | | |
| 500138 | 501025 | + | SAOUHSC_00499 |
|  | | | |
| 508167 | 509531 | + | SAOUHSC_00507 |
|  | | | |

| 511186 | 512640 | + | SAOUHSC_00509 |
| --- | --- | --- | --- |
| 547751 | 550738 | + | SAOUHSC_00544 |
| 549609 | 550460 | - | *srn_1470_sRNA129* |
| 551105 | 555154 | + | SAOUHSC_00545 |
| 556869 | 558359 | - | SAOUHSC_00548 |
| 562979 | 563626 | + | SAOUHSC_00555 |
| 564128 | 565528 | + | SAOUHSC_00556 |
| 574840 | 575490 | - | SAOUHSC_00572 |
| 575845 | 575987 | + | *srn_1510_rsaA* |
| 579485 | 580405 | + | SAOUHSC_00577 |
| 585426 | 586835 | + | SAOUHSC_00584 |
| 593183 | 593752 | + | SAOUHSC_00598 |
| 593789 | 594424 | + | SAOUHSC_00599 |
| 599997 | 600268 | + | *srn_1530_sRNA133* |
| 600268 | 601278 | + | SAOUHSC_00608 |
|  | | | |
| 610262 | 611050 | + | SAOUHSC_00619 |
| 611301 | 611675 | - | SAOUHSC_00620 |
| 611672 | 611798 | - | *srn_1540_teg48* |
| 611843 | 612359 | - | *srn_1550_teg49* |
| 657304 | 657921 | + | SAOUHSC_00669 |
| 659279 | 660287 | + | *srn_1670_teg20as* |
| 659532 | 660329 | - | SAOUHSC_00671 |
|  | | | |
| 660570 | 660695 | + | *srn_1680_sRNA142* |
| 662031 | 664181 | + | SAOUHSC_00673 |
| 664261 | 664686 | + | SAOUHSC_00674 |
| 664876 | 665592 | + | SAOUHSC_00675 |

| **547421..547815** | 547677 | 395 | 49.3 | 0 |
| --- | --- | --- | --- | --- |
| **550804..551102** | 550963 | 299 | 41.5 | 0 |
| **558290..558649** | 558426 | 360 | 41.7 | 0 |
| **562767..563085** | 562946 | 319 | 45.3 | 0 |
| **563718..564132** | 563993 | 415 | 40.3 | 0 |
| **575615..575925** | 575786 | 311 | 45.5 | 0 |
| **578890..579306** | 579030 | 417 | 42.7 | 0 |
| **584873..585280** | 585141 | 408 | 37.6 | 0.00E+00 |
| **592700..593065** | 592840 | 366 | 38.1 | 1.102E-317 |
| **593424..593723** | 593584 | 300 | 43.0 | 0 |
| **599731..600046** | 599871 | 316 | 51.0 | 0 |
| **609967..610253** | 610114 | 287 | 44.6 | 0 |

| **612252..612647** | 612508 | 396 | 37.8 | 2.952E-313 |
| --- | --- | --- | --- | --- |
| **650222..650545** | 650362 | 324 | 37.1 | 2.92E-302 |
| **656836..657149** | 656976 | 314 | 47.6 | 0 |
| **659120..659415** | 659276 | 296 | 45.1 | 0 |
| **660333..660652** | 660473 | 320 | 44.6 | 0 |
| **661669..661966** | 661828 | 298 | 46.7 | 0 |
| **663898..664315** | 664176 | 418 | 35.9 | 4.89E-282 |
| **664535..664874** | 664675 | 340 | 38.0 | 8.009E-317 |

| **666071..666360** | 666211 | 290 | 44.0 | 0 |
| --- | --- | --- | --- | --- |
|  |  |  |  |  |
| **675674..676061** | 675814 | 388 | 36.6 | 5.69E-293 |
| **678088..678385** | 678228 | 298 | 35.4 | 6.86E-275 |
| **679787..680070** | 679931 | 284 | 46.6 | 0 |
| **682306..682652** | 682513 | 347 | 33.8 | 1.47E-250 |
| **686387..686803** | 686665 | 417 | 34.7 | 1.11E-264 |
| **696886..697167** | 697028 | 282 | 38.4 | 2.17E-322 |
| **699841..700126** | 699981 | 286 | 38.8 | 0 |
| **702161..702446** | 702301 | 286 | 45.5 | 0 |
| **709742..710159** | 710020 | 418 | 36.5 | 1.85E-291 |
| **730833..731156** | 731017 | 324 | 41.1 | 0 |
| **738682..738997** | 738858 | 316 | 34.6 | 5.78E-262 |
| **744107..744446** | 744307 | 340 | 41.0 | 0 |
| **749649..749931** | 749792 | 283 | 37.3 | 1.37E-304 |
| **764867..765198** | 765059 | 332 | 39.1 | 0 |
| **774129..774425** | 774269 | 297 | 45.3 | 0 |
| **783242..783660** | 783382 | 419 | 46.7 | 0 |
| **790455..790787** | 790648 | 333 | 39.5 | 0 |
| **790944..791295** | 791084 | 352 | 44.9 | 0 |
| **791919..792221** | 792059 | 303 | 35.7 | 2.23E-279 |
| **792709..792997** | 792849 | 289 | 34.9 | 1.48E-267 |
| **798919..799198** | 799059 | 280 | 34.3 | 8.61E-258 |

|  | | | |
| --- | --- | --- | --- |
| 666324 | 666529 | + | *srn_1690_sRNA143* |
| 666418  674809 | 667062  675684 | +  - | SAOUHSC_00678 SAOUHSC_00691 |
| 675903 | 677534 | + | SAOUHSC_00692 |
| 679331 | 679774 | - | SAOUHSC_00694 |
| 680001 | 680927 | + | SAOUHSC_00695 |
| 682543 | 684096 | + | SAOUHSC_00698 |
| 685991 | 686608 | - | SAOUHSC_00701 |
| 697168 | 698007 | + | SAOUHSC_00712 |
| 701725 | 702165 | - | SAOUHSC_00717 |
| 710153 | 712093 | + | SAOUHSC_00728 |
| 731044 | 732015 | + | SAOUHSC_00746 |
| 738977 | 740101 | + | SAOUHSC_00756 |
| 744387 | 745442 | + | SAOUHSC_00762 |
| 749940 | 752471 | + | SAOUHSC_00769 |
| 765448 | 766887 | + | SAOUHSC_00784 |
| 773243 | 774145 | - | SAOUHSC_00792 |
| 774294 | 774421 | + | *srn_1910_rsaH* |
| 783701 | 784159 | + | SAOUHSC_00800 |
| 790206 | 790934 | - | SAOUHSC_00808 |
| 791370 | 791525 | + | SAOUHSC_00809 |
| 792193 | 792723 | + | SAOUHSC_00811 |
| 792986 | 795769 | + | SAOUHSC_00812 |
| 799223 | 799693 | + | SAOUHSC_00817 |

| 800034 | 800720 | + | SAOUHSC_00818 |
| --- | --- | --- | --- |
| 801077 | 801277 | + | SAOUHSC_00819 |
| 801491 | 801586 | - | *srn_1960_rsaOL* |
|  | | | |
| 803399 | 803689 | - | *srn_1980_sRNA169* |
| 804000 | 804203 | + | SAOUHSC_00825 |
| 805315 | 805932 | - | SAOUHSC_00828 |
| 806087 | 806581 | - | SAOUHSC_00830 |
| 807550 | 808266 | + | SAOUHSC_00832 |
| 814851 | 815672 | + | SAOUHSC_00844 |
| 816453 | 817304 | - | SAOUHSC_00846 |
| 817632 | 818393 | + | SAOUHSC_00847 |
| 834107 | 834259 | + | SAOUHSC_00868 |
| 855526 | 855656 | + | *srn_2080_sau6851* |
| 855577 | 855921 | + | SAOUHSC_00892 |
| 875667 | 876569 | + | SAOUHSC_00906 |
| 877458 | 878774 | - | SAOUHSC_00908 |
| 880619 | 882433 | + | SAOUHSC_00911 |
| 885304 | 886173 | - | SAOUHSC_00913 |
| 886283 | 887428 | + | SAOUHSC_00914 |
| 889148 | 889663 | + | SAOUHSC_00917 |
| 890665 | 890850 | - | SAOUHSC_00919 |
| 891145 | 892086 | + | SAOUHSC_00920 |
| 893422 | 893833 | - | *srn_2125_tsr17* |
| 894011  899923  905548 | 894937  901638  906537 | +  +  - | SAOUHSC_00923  SAOUHSC_00928 SAOUHSC_00933 |

| **799718..800057** | 799858 | 340 | 42.7 | 0 |
| --- | --- | --- | --- | --- |
| **800752..801053** | 800914 | 302 | 45.9 | 0 |
| **801539..801819** | 801679 | 281 | 44.2 | 0 |
| **803501..803917** | 803779 | 417 | 42.8 | 0 |
| **805904..806195** | 806043 | 292 | 35.9 | 3.02E-282 |
| **806544..806962** | 806684 | 419 | 37.8 | 1.948E-312 |
| **814657..814984** | 814845 | 328 | 35.0 | 2.64E-269 |
| **817344..817634** | 817484 | 291 | 45.1 | 0 |
| **833799..834106** | 833967 | 308 | 42.6 | 0 |
| **855136..855443** | 855304 | 308 | 42.8 | 0 |
| **875315..875606** | 875467 | 292 | 39.5 | 0 |
| **878678..878965** | 878826 | 288 | 38.1 | 3.843E-317 |
| **880272..880572** | 880412 | 301 | 53.6 | 0 |
| **886092..886396** | 886231 | 305 | 50.4 | 0 |
| **887923..888279** | 888055 | 357 | 39.7 | 0 |
| **888604..888904** | 888744 | 301 | 48.0 | 0 |
| **889632..889935** | 889772 | 304 | 47.5 | 0 |
| **890827..891155** | 891016 | 329 | 40.6 | 0 |
| **893807..894109** | 893970 | 303 | 43.7 | 0 |
| **899714..899995** | 899854 | 282 | 38.2 | 5.476E-320 |

| **906558..906858** | 906698 | 301 | 39.8 | 0 |
| --- | --- | --- | --- | --- |
| **907328..907617** | 907478 | 290 | 34.0 | 4.31E-253 |
| **909186..909476** | 909337 | 291 | 46.6 | 0 |
| **911425..911710** | 911571 | 286 | 47.3 | 0 |
| **912867..913152** | 913006 | 286 | 34.4 | 7.27E-260 |
| **913541..913834** | 913681 | 294 | 46.3 | 0 |
| **922139..922427** | 922279 | 289 | 34.9 | 1.03E-266 |
| **924789..925075** | 924929 | 287 | 42.7 | 0 |
| **928133..928426** | 928287 | 294 | 45.2 | 0 |
| **939432..939735** | 939571 | 304 | 46.7 | 0 |
| **941972..942261** | 942122 | 290 | 33.7 | 9.01E-249 |
| **943724..944024** | 943864 | 301 | 44.9 | 0 |
| **944429..944776** | 944639 | 348 | 34.7 | 2.20E-264 |
| **946652..946974** | 946792 | 323 | 46.9 | 0 |
| **949305..949711** | 949572 | 407 | 47.8 | 0 |
| **960420..960750** | 960560 | 331 | 46.2 | 0 |
| **963170..963529** | 963390 | 360 | 38.3 | 2.88E-321 |
| **967764..968056** | 967904 | 293 | 46.7 | 0 |
| **970546..970839** | 970700 | 294 | 48.9 | 0 |

| 906832 | 907227 | + | SAOUHSC_00934 |
| --- | --- | --- | --- |
| 907598 | 908317 | + | SAOUHSC_00935 |
| 909472 | 911280 | + | SAOUHSC_00937 |
| 913038 | 913631 | - | SAOUHSC_00940 |
| 913817 | 914164 | + | SAOUHSC_00941 |
| 920996 | 922081 | - | SAOUHSC_00948 |
| 922423 | 923991 | + | SAOUHSC_00949 |
| 925085 | 925594 | + | SAOUHSC_00951 |
| 926875 | 928050 | - | SAOUHSC_00953 |
| 928483 | 929964 | + | SAOUHSC_00954 |
| 938930 | 939460 | - | SAOUHSC_00961 |
| 939709 | 939927 | + | SAOUHSC_00962 |
| 942176 | 942416 | + | *srn_2230_sprG2* |
| 943109 | 943329 | - | *srn_2270_sRNA192* |
| 945297 | 945617 | + | SAOUHSC_00969 |
| 946343 | 946633 | - | SAOUHSC_00971 |
| 946912 | 947199 | + | SAOUHSC_00972 |
| 948776 | 949132 | - | SAOUHSC_00975 |
| 949716 | 950579 | + | SAOUHSC_00976 |
| 959416 | 960426 | - | SAOUHSC_00988 |
| 962304 | 963314 | - | SAOUHSC_00991 |
| 963467 | 963886 | + | SAOUHSC_00992 |
| 964093 | 967863 | - | SAOUHSC_00994 |
| 969158 | 969331 | + | *srn_2310_sRNA194* |
| 969198 | 970415 | - | SAOUHSC_00997 |
| 970866  977138 | 972059  977455 | +  - | SAOUHSC_00998 SAOUHSC_01005 |

| 1042227 | 1044164 | - | SAOUHSC_01079 |
| --- | --- | --- | --- |
| 1045019 | 1045289 | + | *srn_2420_sRNA201* |
| 1045629 | 1046312 | + | SAOUHSC_01082 |
| 1050762 | 1051502 | + | SAOUHSC_01091 |
| 1070537 | 1070866 | + | SAOUHSC_01110 |
| 1070943 | 1071105 | + | *srn_2460_teg58* |
| 1071235 | 1071636 | - | SAOUHSC_01112 |
| 1072308 | 1072814 | + | SAOUHSC_01113 |
| 1073073 | 1073570 | + | SAOUHSC_01114 |
| 1075703 | 1075936 | - | SAOUHSC_01120 |
| 1076413 | 1077372 | - | SAOUHSC_01121 |
| 1078045 | 1078191 | + | SAOUHSC_01122 |
| 1080460 | 1081185 | - | SAOUHSC_01127 |
| 1081623 | 1082624 | + | SAOUHSC_01128 |
| 1085279 | 1085369 | - | *srn_2510_sau6297* |
| 1085615 | 1085842 | + | SAOUHSC_01131 |
| 1085894 | 1086109 | + | *srn_2520_teg60* |
| 1103160 | 1103825 | + | SAOUHSC_01152 |
| 1132021 | 1132974 | + | SAOUHSC_01180 |
| 1171904 | 1172644 | + | SAOUHSC_01221 |
| 1176773 | 1177669 | + | SAOUHSC_01224 |
| 1202091 | 1204187 | + | SAOUHSC_01251 |
| 1226777 | 1229299 | + | SAOUHSC_01272 |
| 1245527 | 1245751 | + | SAOUHSC_01289 |

| **977438..977729** | 977590 | 292 | 37.5 | 2.22E-307 |
| --- | --- | --- | --- | --- |
| **1044143..1044427** | 1044283 | 285 | 48.4 | 0 |
| **1045425..1045720** | 1045581 | 296 | 39.1 | 0 |
| **1050473..1050774** | 1050613 | 302 | 44.3 | 0 |
| **1070230..1070549** | 1070410 | 320 | 49.1 | 0 |
| **1070710..1071003** | 1070864 | 294 | 46.1 | 0 |
| **1071840..1072200** | 1072061 | 361 | 52.3 | 0 |
| **1072908..1073316** | 1073047 | 409 | 37.5 | 3.47E-308 |
| **1075974..1076392** | 1076114 | 419 | 43.4 | 0 |
| **1077624..1078042** | 1077764 | 419 | 50.8 | 0 |
| **1081287..1081593** | 1081454 | 307 | 38.6 | 0 |
| **1085364..1085656** | 1085503 | 293 | 33.9 | 1.02E-252 |
| **1085656..1085989** | 1085850 | 334 | 34.7 | 4.97E-264 |
| **1102781..1103087** | 1102948 | 307 | 38.2 | 6.331E-319 |
| **1131637..1131927** | 1131776 | 291 | 48.3 | 0 |
| **1170458..1170766** | 1170627 | 309 | 39.6 | 0 |
| **1171327..1171584** | 1171445 | 258 | 41.8 | 0 |
| **1176369..1176688** | 1176549 | 320 | 37.2 | 2.50E-303 |
| **1201754..1202091** | 1201894 | 338 | 42.8 | 0 |
| **1226356..1226642** | 1226496 | 287 | 46.5 | 0 |
| **1244843..1245132** | 1244983 | 290 | 48.4 | 0 |
| **1245383..1245741** | 1245523 | 359 | 46.2 | 0 |

| **1245741..1246087** | 1245949 | 347 | 34.9 | 4.79E-267 |
| --- | --- | --- | --- | --- |
| **1246427..1246784** | 1246567 | 358 | 46.3 | 0 |
| **1247271..1247541** | 1247407 | 271 | 41.7 | 0 |
| **1248249..1248530** | 1248389 | 282 | 38.4 | 1E-323 |
| **1249051..1249468** | 1249191 | 418 | 42.6 | 0 |
| **1250052..1250332** | 1250193 | 281 | 42.3 | 0 |
| **1251396..1251785** | 1251646 | 390 | 47.2 | 0 |
| **1253477..1253828** | 1253617 | 352 | 35.7 | 1.31E-279 |
| **1255400..1255732** | 1255540 | 333 | 38.9 | 0 |
| **1256327..1256712** | 1256467 | 386 | 37.9 | 2.638E-315 |
| **1257213..1257504** | 1257353 | 292 | 44.6 | 0 |
| **1259778..1260062** | 1259918 | 285 | 47.0 | 0 |
|  |  |  |  |  |
| **1260795..1261102** | 1260963 | 308 | 36.9 | 4.52E-299 |
| **1271870..1272259** | 1272010 | 390 | 43.4 | 0 |
| **1272479..1272774** | 1272619 | 296 | 38.0 | 6.291E-316 |
| **1274742..1275063** | 1274924 | 322 | 36.1 | 6.58E-285 |
| **1276328..1276610** | 1276471 | 283 | 36.8 | 7.34E-297 |
| **1280785..1281091** | 1280952 | 307 | 42.1 | 0 |
| **1281724..1282072** | 1281862 | 349 | 36.5 | 3.08E-291 |
| **1282181..1282500** | 1282321 | 320 | 38.8 | 0 |
| **1282500..1282799** | 1282660 | 300 | 41.5 | 0 |
| **1284400..1284712** | 1284540 | 313 | 35.5 | 3.72E-276 |
| **1298532..1298826** | 1298672 | 295 | 48.2 | 0 |

| 1246045 | 1246251 | + | SAOUHSC_01290 |
| --- | --- | --- | --- |
| 1246952 | 1247062 | + | SAOUHSC_01291 |
| 1247490 | 1247675 | + | SAOUHSC_01292 |
| 1248420 | 1248671 | + | SAOUHSC_01295 |
| 1249422 | 1249670 | + | SAOUHSC_01297 |
| 1250210 | 1250347 | + | SAOUHSC_01301 |
| 1251668 | 1251859 | + | SAOUHSC_01306 |
| 1252408 | 1253433 | - | SAOUHSC_01307 |
| 1253703 | 1253900 | + | SAOUHSC_01307a |
| 1255614 | 1256522 | + | SAOUHSC_01311 |
| 1256058 | 1256100 | - | *srn_2770_sau6904* |
| 1256491  1258314 | 1257222  1258916 | +  + | SAOUHSC_01312 SAOUHSC_01314 |
| 1260032 | 1260886 | - | SAOUHSC_01317 |
| 1260933 | 1261002 | - | *srn_2780_sau6282* |
|  | | | |
| 1260987 | 1261203 | + | *srn_2790_sRNA224* |
| 1272237 | 1272506 | + | SAOUHSC_01329 |
| 1272663 | 1273640 | + | SAOUHSC_01330 |
| 1276664 | 1278541 | + | SAOUHSC_01337 |
| 1280956 | 1283986 | + | SAOUHSC_01343 |
| 1284057 | 1284419 | - | SAOUHSC_1342a |
| 1284623 | 1286269 | + | SAOUHSC_01346 |
| 1298809 | 1299660 | + | SAOUHSC_01356 |

| **1301175..1301514** | 1301375 | 340 | 52.6 | 0 |
| --- | --- | --- | --- | --- |
| **1316719..1317034** | 1316858 | 316 | 35.7 | 1.63E-279 |
| **1318126..1318405** | 1318266 | 280 | 37.4 | 3.00E-306 |
| **1319606..1319905** | 1319766 | 300 | 45.7 | 0 |
| **1325444..1325725** | 1325584 | 282 | 34.6 | 1.14E-262 |
| **1327530..1327814** | 1327670 | 285 | 37.1 | 5.55E-301 |
| **1345309..1345598** | 1345449 | 290 | 54.4 | 0 |
| **1346181..1346474** | 1346335 | 294 | 44.5 | 0 |
| **1351199..1351485** | 1351346 | 287 | 40.4 | 0 |
| **1354074..1354382** | 1354243 | 309 | 45.7 | 0 |
| **1355303..1355644** | 1355443 | 342 | 43.0 | 0 |
| **1362250..1362622** | 1362390 | 373 | 44.2 | 0 |
| **1362679..1362961** | 1362822 | 283 | 39.5 | 0 |
| **1365375..1365719** | 1365515 | 345 | 41.0 | 0 |
| **1370431..1370724** | 1370571 | 294 | 37.6 | 0.00E+00 |
| **1404755..1405057** | 1404895 | 303 | 42.1 | 0 |
| **1410269..1410582** | 1410443 | 314 | 52.1 | 0 |
| **1420413..1420727** | 1420553 | 315 | 40.5 | 0 |
| **1425998..1426297** | 1426138 | 300 | 36.7 | 2.84E-295 |
| **1430141..1430445** | 1430306 | 305 | 34.4 | 2.55E-260 |
| **1441762..1442046** | 1441901 | 285 | 51.2 | 0 |
| **1445095..1445430** | 1445292 | 336 | 39.8 | 0 |

| 1301484 | 1304006 | + | SAOUHSC_01359 |
| --- | --- | --- | --- |
| 1316912 | 1317056 | + | *srn_2910_teg63* |
| 1318357 | 1319616 | + | SAOUHSC_01374 |
| 1320299 | 1321066 | + | SAOUHSC_01375 |
| 1325827 | 1327641 | + | SAOUHSC_01383 |
| 1345917 | 1346117 | - | SAOUHSC_01403 |
| 1346759 | 1347028 | + | SAOUHSC_01406 |
| 1349856 | 1351199 | - | SAOUHSC_01411 |
| 1353324 | 1354115 | - | SAOUHSC_01413 |
| 1354528 | 1355337 | - | SAOUHSC_01415 |
| 1361633 | 1362292 | - | SAOUHSC_01420 |
| 1362554 | 1362685 | - | SAOUHSC_01421 |
| 1362885 | 1363065 | + | *srn_2975_tsr25* |
| 1364792 | 1365301 | - | SAOUHSC_01425 |
| 1370010 | 1370489 | - | SAOUHSC_01434 |
| 1376091 | 1404698 | - | SAOUHSC_01447 |
| 1409132 | 1410250 | - | SAOUHSC_01452 |
| 1420039 | 1420389 | - | SAOUHSC_01464 |
| 1420878 | 1421555 | + | SAOUHSC_01466 |
| 1425296 | 1425982 | - | SAOUHSC_01470 |
| 1440410 | 1441576 | - | SAOUHSC_01483 |
| 1444601 | 1445173 | - | SAOUHSC_01488 |
| 1445639 | 1445786 | + | *srn_3040_sau50* |
| 1448594 | 1449769 | - | SAOUHSC_01493 |

| 1450171 | 1450308 | + | SAOUHSC_01494 |
| --- | --- | --- | --- |
| 1453447 | 1453563 | - | SAOUHSC_01500 |
| 1453688 | 1455148 | - | SAOUHSC_01501 |
| 1510560 | 1511429 | - | SAOUHSC_01584 |
| 1513187 | 1513346 | + | *srn_3090_sRNA245* |
| 1520498 | 1521253 | - | SAOUHSC_01596 |
| 1521425 | 1522210 | + | SAOUHSC_01597 |
| 1522315 | 1523235 | - | SAOUHSC_01598 |
| 1523548 | 1525032 | + | SAOUHSC_01599 |
| 1541182 | 1541634 | - | SAOUHSC_01617 |
| 1556581 | 1556727 | - | SAOUHSC_01636 |
| 1557239 | 1557559 | - | SAOUHSC_01638 |
| 1583920 | 1584867 | - | SAOUHSC_01673 |
| 1586893 | 1587576 | - | SAOUHSC_01677 |
| 1588289 | 1589635 | - | SAOUHSC_01679 |
| 1611370 | 1612074 | - | SAOUHSC_01705 |
| 1632959 | 1634101 | - | SAOUHSC_01727 |
| 1634413 | 1635426 | + | SAOUHSC_01728 |
| 1644149 | 1646338 | - | SAOUHSC_01742 |
| 1647306 | 1649579 | - | SAOUHSC_01744 |
| 1673762 | 1674688 | - | SAOUHSC_01774 |
| 1675567 | 1676913 | - | SAOUHSC_01776 |
| 1679287 | 1680588 | - | SAOUHSC_01779 |
| 1696753 | 1698240 | - | SAOUHSC_01798 |

| **1449900..1450304** | 1450040 | 405 | 34.2 | 4.27E-257 |
| --- | --- | --- | --- | --- |
| **1453360..1453777** | 1453638 | 418 | 44.1 | 0 |
| **1455123..1455421** | 1455262 | 299 | 38.8 | 0 |
| **1510064..1510480** | 1510204 | 417 | 35.4 | 4.11E-275 |
| **1511402..1511693** | 1511554 | 292 | 41.0 | 0 |
| **1521180..1521473** | 1521334 | 294 | 44.8 | 0 |
| **1523170..1523460** | 1523321 | 291 | 38.1 | 4.578E-318 |
| **1541592..1541981** | 1541842 | 390 | 37.1 | 4.06E-301 |
| **1551526..1551898** | 1551665 | 373 | 33.8 | 1.26E-250 |
| **1556635..1556980** | 1556774 | 346 | 43.6 | 0 |
| **1557508..1557795** | 1557648 | 288 | 45.7 | 0 |
| **1584844..1585141** | 1584984 | 298 | 40.1 | 0 |
| **1587546..1587850** | 1587686 | 305 | 37.4 | 8.42E-307 |
| **1590097..1590406** | 1590267 | 310 | 36.0 | 4.89E-284 |
| **1611076..1611408** | 1611269 | 333 | 41.3 | 0 |
| **1612364..1612669** | 1612530 | 306 | 43.3 | 0 |
| **1634107..1634409** | 1634247 | 303 | 34.3 | 2.38E-258 |
| **1646553..1646851** | 1646712 | 299 | 51.3 | 0 |
| **1649491..1649809** | 1649631 | 319 | 38.6 | 0 |
| **1674650..1674951** | 1674790 | 302 | 41.5 | 0 |
| **1676917..1677216** | 1677057 | 300 | 41.8 | 0 |
| **1680593..1680898** | 1680733 | 306 | 39.5 | 0 |

| **1698266..1698553** | 1698406 | 288 | 46.8 | 0 |
| --- | --- | --- | --- | --- |
| **1706069..1706393** | 1706209 | 325 | 40.7 | 0 |
| **1710713..1711010** | 1710871 | 298 | 34.6 | 3.47E-263 |
| **1712779..1713085** | 1712918 | 307 | 40.5 | 0 |
|  |  |  |  |  |
| **1714339..1714668** | 1714479 | 330 | 55.0 | 0 |
| **1728331..1728646** | 1728507 | 316 | 37.5 | 7.60E-308 |
| **1733594..1733883** | 1733734 | 290 | 49.3 | 0 |
| **1752127..1752418** | 1752279 | 292 | 34.1 | 1.09E-254 |
| **1757352..1757712** | 1757492 | 361 | 47.2 | 0 |
| **1758945..1759258** | 1759085 | 314 | 41.1 | 0 |
| **1788130..1788467** | 1788270 | 338 | 43.7 | 0 |
| **1788467..1788804** | 1788665 | 338 | 36.0 | 4.45E-284 |
|  |  |  |  |  |
| **1794960..1795296** | 1795100 | 337 | 52.3 | 0 |
| **1802141..1802434** | 1802281 | 294 | 36.2 | 7.32E-288 |
| **1803909..1804221** | 1804049 | 313 | 35.3 | 4.07E-273 |
|  |  |  |  |  |
| **1807780..1808066** | 1807920 | 287 | 34.3 | 7.08E-259 |
| **1808086..1808377** | 1808238 | 292 | 33.9 | 4.00E-252 |
| **1809724..1810100** | 1809864 | 377 | 36.0 | 4.56E-284 |
| **1811866..1812125** | 1812006 | 260 | 35.4 | 7.31E-275 |
| **1812131..1812547** | 1812408 | 417 | 36.6 | 5.53E-294 |

| 1709745 | 1710668 | - | SAOUHSC_01807 |
| --- | --- | --- | --- |
| 1711926 | 1712783 | - | SAOUHSC_01809 |
| 1712978 | 1714207 | - | SAOUHSC_01810 |
| 1714211 | 1714493 | - | *srn_3360_sRNA267* |
| 1727898 | 1728392 | - | SAOUHSC_01822 |
| 1732038 | 1733732 | - | SAOUHSC_01827 |
| 1733869 | 1734333 | + | SAOUHSC_01828 |
| 1750440 | 1752107 | - | SAOUHSC_01845 |
| 1756346 | 1757335 | - | SAOUHSC_01850 |
| 1757875 | 1758966 | - | SAOUHSC_01852 |
| 1781582 | 1788142 | - | SAOUHSC_01873 |
| 1788485 | 1788796 | - | SAOUHSC_01874 |
| 1794447 | 1794848 | - | SAOUHSC_01879 |
| 1794850 | 1795093 | - | *srn_3500_sRNA277* |
|  | | | |
| 1800919 | 1801923 | - | SAOUHSC_01889 |
| 1801961 | 1802261 | - | *srn_3510_teg74* |
| 1802443 | 1803945 | - | SAOUHSC_01890 |
| 1804451 | 1804783 | + | *srn_3520_sRNA279* |
| 1804468 | 1804782 | + | SAOUHSC_01891 |
| 1806782 | 1807636 | - | SAOUHSC_01895 |
| 1808335 | 1808805 | + | SAOUHSC_01897 |
| 1809348 | 1809791 | - | SAOUHSC_01899 |
| 1810087 | 1810722 | + | SAOUHSC_01900 |
| 1811162 | 1811875 | - | SAOUHSC_01901 |
| 1812135 | 1812437 | + | SAOUHSC_01902 |
| 1812693 | 1813058 | + | SAOUHSC_01903 |

| **1826279..1826575** | 1826419 | 297 | 45.8 | 0 |
| --- | --- | --- | --- | --- |
| **1830915..1831201** | 1831064 | 287 | 44.5 | 0 |
| **1833542..1833916** | 1833777 | 375 | 47.8 | 0 |
| **1834479..1834824** | 1834614 | 346 | 37.1 | 3.69E-302 |
| **1835605..1836018** | 1835880 | 414 | 40.9 | 0 |
| **1836242..1836462** | 1836323 | 221 | 39.7 | 0 |
| **1837859..1838173** | 1837999 | 315 | 53.5 | 0 |
| **1839175..1839500** | 1839315 | 326 | 41.0 | 0 |
| **1847284..1847593** | 1847454 | 310 | 49.3 | 0 |
| **1848028..1848373** | 1848234 | 346 | 41.9 | 0 |
|  |  |  |  |  |
| **1857924..1858306** | 1858064 | 383 | 40.2 | 0 |
| **1858755..1859055** | 1858895 | 301 | 40.5 | 0 |
| **1860861..1861278** | 1861001 | 418 | 49.7 | 0 |
| **1861427..1861754** | 1861615 | 328 | 49.9 | 0 |
| **1862883..1863190** | 1863051 | 308 | 39.7 | 0 |
| **1863808..1864104** | 1863948 | 297 | 54.8 | 0 |
| **1874845..1875138** | 1874999 | 294 | 51.0 | 0 |
|  |  |  |  |  |
| **1876304..1876600** | 1876443 | 297 | 34.1 | 3.07E-255 |
| **1889762..1890047** | 1889902 | 286 | 41.1 | 0 |
| **1896900..1897180** | 1897040 | 281 | 42.7 | 0 |
| **1906554..1906947** | 1906694 | 394 | 51.8 | 0 |
| **1906947..1907302** | 1907163 | 356 | 46.0 | 0 |
| **1909089..1909375** | 1909229 | 287 | 41.6 | 0 |

| 1825909 | 1826364 | - | SAOUHSC_01917 |
| --- | --- | --- | --- |
| 1826667 | 1827317 | + | SAOUHSC_01918 |
| 1830347 | 1830988 | - | SAOUHSC_01923 |
| 1846654 | 1847361 | - | SAOUHSC_01942 |
| 1848325 | 1848891 | + | SAOUHSC_01944 |
| 1857745 | 1857888 | - | SAOUHSC_01953 |
| 1857773 | 1857902 | - | *srn_9335.1_tsr29* |
|  | | | |
| 1858652 | 1858782 | - | *srn_9335_tsr29* |
| 1859995 | 1860900 | - | SAOUHSC_01955 |
| 1861445 | 1861598 | - | *srn_3610_sprC* |
| 1861729 | 1862268 | + | SAOUHSC_01956 |
| 1863742 | 1863897 | - | *srn_9340_sRNA287* |
|  | | | |
| 1875110 | 1875207 | + | *srn_3630_sau69* |
| 1875249 | 1876211 | + | SAOUHSC_01972 |
| 1876332 | 1877273 | - | SAOUHSC_01973 |
| 1889485 | 1889880 | - | SAOUHSC_01984 |
| 1889999 | 1890361 | + | SAOUHSC_01985 |
| 1895775 | 1896836 | - | SAOUHSC_01992 |
| 1897329 | 1898648 | + | SAOUHSC_01993 |
| 1904931 | 1906485 | - | SAOUHSC_R0003 |
| 1908723 | 1909178 | - | SAOUHSC_01999 |
| 1909259 | 1910548 | + | SAOUHSC_02000 |
| 1914153 | 1914695 | - | SAOUHSC_02004 |

| **1914668..1914986** | 1914808 | 319 | 42.2 | 0 |
| --- | --- | --- | --- | --- |
| **1922056..1922378** | 1922239 | 323 | 41.1 | 0 |
| **1987952..1988336** | 1988092 | 385 | 42.7 | 0 |
| **1991578..1991874** | 1991735 | 297 | 42.5 | 0 |
| **2001938..2002257** | 2002118 | 320 | 54.6 | 0 |
| **2003489..2003880** | 2003741 | 392 | 42.5 | 0 |
| **2004903..2005210** | 2005043 | 308 | 34.8 | 4.29E-265 |
| **2007330..2007663** | 2007524 | 334 | 39.3 | 0 |
| **2019043..2019378** | 2019239 | 336 | 35.3 | 1.64E-272 |
| **2024550..2024967** | 2024828 | 418 | 37.8 | 1.334E-313 |
| **2031388..2031777** | 2031639 | 390 | 48.8 | 0 |
| **2077813..2078130** | 2077953 | 318 | 41.4 | 0 |
| **2079753..2080054** | 2079893 | 302 | 36.8 | 2.42E-296 |
| **2081560..2081849** | 2081710 | 290 | 43.2 | 0 |
| **2082696..2082977** | 2082838 | 282 | 40.3 | 0 |
| **2111253..2111537** | 2111393 | 285 | 44.7 | 0 |
|  |  |  |  |  |
| **2128264..2128575** | 2128404 | 312 | 51.2 | 0 |
| **2128672..2128988** | 2128849 | 317 | 36.8 | 3.23E-297 |
| **2131564..2131981** | 2131842 | 418 | 44.1 | 0 |
| **2133479..2133777** | 2133619 | 299 | 41.0 | 0 |
| **2134673..2135006** | 2134867 | 334 | 38.6 | 0 |
| **2149729..2150113** | 2149869 | 385 | 36.7 | 7.14E-296 |

| 1921172 | 1921981 | - | SAOUHSC_02012 |
| --- | --- | --- | --- |
| 1986589 | 1987536 | - | SAOUHSC_02114 |
| 1991204 | 1991506 | - | SAOUHSC_02118 |
| 1991873 | 1993411 | + | SAOUHSC_02119 |
| 2000183 | 2001478 | - | SAOUHSC_02126 |
| 2002339 | 2003505 | + | SAOUHSC_02127 |
| 2004308 | 2004910 | - | SAOUHSC_02131 |
| 2005995 | 2007464 | - | SAOUHSC_02133 |
| 2007648 | 2008724 | + | SAOUHSC_02134 |
| 2018697 | 2018870 | - | SAOUHSC_02145 |
| 2019321 | 2020361 | + | SAOUHSC_02146 |
| 2023888 | 2024760 | - | SAOUHSC_02152 |
| 2029598 | 2031352 | - | SAOUHSC_02161 |
| 2076731 | 2077786 | - | SAOUHSC_02243 |
| 2078221 | 2079444 | + | SAOUHSC_02244 |
| 2080723 | 2081634 | - | SAOUHSC_02246 |
| 2081795 | 2083102 | + | SAOUHSC_02247 |
| 2110834 | 2111268 | - | SAOUHSC_02280 |
| 2111499 | 2111723 | + | *srn_3950_teg16* |
| 2111806 | 2113494 | + | SAOUHSC_02281 |
| 2128216 | 2128323 | - | SAOUHSC_02294 |
| 2133155 | 2133481 | - | SAOUHSC_02300 |
| 2148167 | 2149687 | - | SAOUHSC_02316 |
| 2152214 | 2152379 | + | *srn_3980_sRNA324* |

| **2166838..2167128** | 2166989 | 291 | 34.0 | 8.08E-254 |
| --- | --- | --- | --- | --- |
| **2187913..2188330** | 2188191 | 418 | 37.8 | 1.806E-313 |
| **2192492..2192777** | 2192638 | 286 | 35.7 | 3.12E-279 |
| **2204060..2204378** | 2204200 | 319 | 35.8 | 3.54E-281 |
| **2210330..2210641** | 2210470 | 312 | 35.2 | 2.43E-272 |
| **2214540..2214945** | 2214679 | 406 | 47.2 | 0 |
| **2235942..2236257** | 2236082 | 316 | 35.9 | 1.82E-282 |
| **2267396..2267690** | 2267536 | 295 | 35.2 | 5.15E-271 |
| **2269143..2269435** | 2269283 | 293 | 34.9 | 9.62E-268 |
| **2281890..2282216** | 2282077 | 327 | 42.5 | 0 |
| **2284472..2284763** | 2284612 | 292 | 41.5 | 0 |
|  |  |  |  |  |
| **2293868..2294222** | 2294008 | 355 | 34.8 | 4.66E-266 |
| **2294791..2295195** | 2295059 | 405 | 53.3 | 0 |
| **2295269..2295686** | 2295547 | 418 | 53.7 | 0 |
| **2344470..2344754** | 2344610 | 285 | 36.8 | 3.71E-297 |
| **2348272..2348631** | 2348492 | 360 | 42.9 | 0 |
| **2351964..2352266** | 2352127 | 303 | 48.5 | 0 |
| **2357890..2358234** | 2358030 | 345 | 43.3 | 0 |
| **2361409..2361734** | 2361549 | 326 | 52.8 | 0 |
| **2365814..2366129** | 2365954 | 316 | 34.0 | 1.37E-253 |
| **2366358..2366661** | 2366523 | 304 | 35.7 | 6.62E-280 |

| 2166644 | 2166877 | - | SAOUHSC_02338 |
| --- | --- | --- | --- |
| 2186626 | 2187885 | - | SAOUHSC_02365 |
| 2192005 | 2192535 | - | SAOUHSC_02369 |
| 2203639 | 2204052 | - | SAOUHSC_02382 |
| 2210575 | 2211666 | + | SAOUHSC_02390 |
| 2214716 | 2214878 | + | *srn_9480_sRNA334* |
| 2235152 | 2235961 | - | SAOUHSC_02407 |
| 2266921 | 2267457 | - | SAOUHSC_02443 |
| 2267632 | 2269194 | - | SAOUHSC_02444 |
| 2281666 | 2281827 | + | SAOUHSC_A02331 |
| 2284053 | 2284469 | - | SAOUHSC_02461 |
| 2284707 | 2284857 | + | *srn_4205_tsr32* |
| 2284713 | 2284880 | + | SAOUHSC_02462 |
| 2293660 | 2293756 | - | *srn_4220_sRNA345* |
| 2295453 | 2296589 | - | SAOUHSC_02472 |
| 2343695 | 2344477 | - | SAOUHSC_02549 |
| 2344767 | 2345564 | + | SAOUHSC_02550 |
| 2347253 | 2348194 | - | SAOUHSC_02553 |
| 2351097 | 2351987 | - | SAOUHSC_02557 |
| 2352262 | 2352564 | + | SAOUHSC_02558 |
| 2357499 | 2357846 | - | SAOUHSC_02566 |
| 2359433 | 2361388 | - | SAOUHSC_02570 |
| 2361998 | 2362801 | + | SAOUHSC_02571 |
| 2364729 | 2365811 | - | SAOUHSC_02574 |
| 2366059 | 2366481 | + | SAOUHSC_02575 |

| 2366718 | 2367218 | + | SAOUHSC_02576 |
| --- | --- | --- | --- |
| 2371792 | 2374746 | - | SAOUHSC_02582 |
| 2378949 | 2379686 | - | SAOUHSC_02587 |
| 2380509 | 2381381 | + | SAOUHSC_02589 |
| 2402756 | 2403442 | - | SAOUHSC_02612 |
| 2403616 | 2404269 | + | SAOUHSC_02613 |
| 2408453 | 2409100 | - | SAOUHSC_02620 |
| 2409499 | 2410107 | + | SAOUHSC_02621 |
| 2410444 | 2411652 | + | SAOUHSC_02622 |
| 2423442 | 2423897 | - | SAOUHSC_02636 |
| 2424092 | 2425060 | + | SAOUHSC_02638 |
| 2453508 | 2453873 | - | SAOUHSC_02668 |
| 2454271 | 2454717 | + | SAOUHSC_02669 |
| 2474417 | 2474599 | - | SAOUHSC_02688 |
| 2475336 | 2476886 | - | SAOUHSC_02690 |
| 2478589 | 2478939 | - | SAOUHSC_02695 |
| 2479206 | 2480456 | + | SAOUHSC_02696 |
| 2484752 | 2485438 | - | SAOUHSC_02703 |
| 2487184 | 2487288 | - | SAOUHSC_02705 |
| 2487383 | 2488693 | + | SAOUHSC_02706 |
| 2490726 | 2491673 | + | SAOUHSC_02709 |
| 2500219 | 2501982 | - | SAOUHSC_02719 |
| 2502532 | 2502593 | + | *srn_4540_sprAs2* |
| 2529341 | 2530468 | - | SAOUHSC_02752 |
| 2531693  2555933 | 2532355  2556124 | -  - | SAOUHSC_02754 SAOUHSC_02781 |
| 2556335 | 2556409 | + | *srn_4680_sau19* |

| **2374792..2375072** | 2374932 | 281 | 45.4 | 0 |
| --- | --- | --- | --- | --- |
| **2379872..2380174** | 2380037 | 303 | 35.8 | 1.18E-280 |
| **2403370..2403655** | 2403516 | 286 | 35.6 | 1.66E-277 |
| **2409306..2409588** | 2409446 | 283 | 42.7 | 0 |
| **2410155..2410444** | 2410306 | 290 | 35.2 | 7.06E-272 |
| **2420058..2420452** | 2420313 | 395 | 34.3 | 1.82E-258 |
| **2423929..2424222** | 2424069 | 294 | 36.8 | 3.31E-297 |
| **2453919..2454228** | 2454090 | 310 | 38.0 | 1.138E-316 |
| **2474399..2474760** | 2474621 | 362 | 36.5 | 1.17E-291 |
| **2476956..2477247** | 2477109 | 292 | 34.7 | 1.14E-263 |
| **2478951..2479246** | 2479107 | 296 | 41.7 | 0 |
| **2485376..2485688** | 2485549 | 313 | 34.2 | 4.83E-257 |
| **2487192..2487479** | 2487340 | 288 | 41.9 | 0 |
| **2490228..2490536** | 2490368 | 309 | 40.9 | 0 |
| **2501937..2502293** | 2502077 | 357 | 41.9 | 0 |
| **2502293..2502614** | 2502475 | 322 | 36.6 | 6.90E-294 |
| **2530490..2530800** | 2530630 | 311 | 36.6 | 4.34E-293 |
| **2532321..2532628** | 2532461 | 308 | 37.5 | 0.00E+00 |
| **2556128..2556441** | 2556303 | 314 | 39.9 | 0 |

| **2557708..2558060** | 2557922 | 353 | 43.6 | 0 |
| --- | --- | --- | --- | --- |
| **2562314..2562636** | 2562454 | 323 | 37.8 | 7.369E-313 |
| **2565028..2565377** | 2565238 | 350 | 34.1 | 7.63E-256 |
| **2567161..2567446** | 2567307 | 286 | 37.1 | 2.29E-302 |
|  |  |  |  |  |
| **2568145..2568482** | 2568343 | 338 | 34.6 | 7.43E-263 |
| **2575349..2575766** | 2575627 | 418 | 34.7 | 8.44E-264 |
| **2576212..2576471** | 2576352 | 260 | 38.8 | 0 |
| **2580745..2581031** | 2580892 | 287 | 35.1 | 8.67E-270 |
| **2594827..2595100** | 2594967 | 274 | 34.0 | 5.45E-253 |
| **2597845..2598139** | 2598000 | 295 | 43.3 | 0 |
| **2607805..2608086** | 2607947 | 282 | 42.4 | 0 |
| **2610215..2610506** | 2610355 | 292 | 36.7 | 1.32E-295 |
| **2611176..2611466** | 2611315 | 291 | 37.4 | 5.60E-307 |
| **2628478..2628764** | 2628625 | 287 | 37.5 | 4.55E-308 |
| **2629366..2629659** | 2629506 | 294 | 35.3 | 1.00E-273 |
| **2659493..2659799** | 2659660 | 307 | 39.9 | 0 |
| **2675028..2675356** | 2675168 | 329 | 44.0 | 0 |
| **2678803..2679162** | 2678943 | 360 | 45.0 | 0 |
| **2691353..2691667** | 2691528 | 315 | 47.7 | 0 |
| **2714085..2714389** | 2714253 | 305 | 38.5 | 0 |

| 2556559 | 2556681 | + | SAOUHSC_02782 |
| --- | --- | --- | --- |
| 2556685 | 2557479 | - | SAOUHSC_02783 |
| 2564791 | 2565183 | - | SAOUHSC_02791 |
| 2565300 | 2567108 | + | SAOUHSC_02793 |
| 2567390 | 2567726 | + | *srn_4705_tsr37* |
| 2567392 | 2567658 | + | SAOUHSC_02794 |
| 2575197 | 2575517 | - | SAOUHSC_02799 |
| 2575879 | 2576622 | + | SAOUHSC_02800 |
| 2577879 | 2580632 | - | SAOUHSC_02802 |
| 2593502 | 2594779 | - | SAOUHSC_02815 |
| 2595326 | 2595937 | + | SAOUHSC_02816 |
| 2597190 | 2597648 | - | SAOUHSC_02819 |
| 2598082 | 2598777 | + | SAOUHSC_02820 |
| 2607117 | 2607788 | - | SAOUHSC_02829 |
| 2608103 | 2609050 | + | SAOUHSC_02830 |
| 2609299 | 2610099 | - | SAOUHSC_02831 |
| 2610420 | 2611055 | + | SAOUHSC_02832 |
| 2629201 | 2629458 | - | SAOUHSC_02858 |
| 2629651 | 2629724 | + | *srn_9510_sRNA390* |
| 2657597 | 2659408 | - | SAOUHSC_02885 |
| 2674777 | 2675055 | - | SAOUHSC_02907 |
| 2678161 | 2678883 | - | SAOUHSC_02911 |
| 2679237 | 2679686 | + | SAOUHSC_02912 |
| 2691655 | 2692545 | + | SAOUHSC_02926 |
| 2714065 | 2714178 | - | SAOUHSC_02950 |
| 2714402 | 2714625 | - | *srn_4980_teg32* |

| **2714602..2714891** | 2714742 | 290 | 46.4 | 0 |  | | | |
| --- | --- | --- | --- | --- | --- | --- | --- | --- |
|  |  |  |  |  | 2722968 | 2725601 | - | SAOUHSC_02963 |
| **2725536..2725917** | 2725676 | 382 | 37.6 | 4.244E-310 |  |  |  |  |
|  |  |  |  |  | 2732674 | 2734170 | - | SAOUHSC_02971 |
| **2734168..2734510** | 2734308 | 343 | 46.0 | 0 |  |  |  |  |
|  |  |  |  |  | 2734627 | 2735154 | - | SAOUHSC_02972 |
| **2734972..2735389** | 2735250 | 418 | 41.2 | 0 |  |  |  |  |
|  |  |  |  |  | 2735412 | 2735870 | + | SAOUHSC_02973 |
|  |  |  |  |  | 2759929 | 2766744 | - | SAOUHSC_02990 |
| **2766843..2767174** | 2767037 | 332 | 45.5 | 0 |  |  |  |  |
|  |  |  |  |  | 2772939 | 2773601 | - | SAOUHSC_03000 |
| **2773607..2773886** | 2773746 | 280 | 44.5 | 0 |  |  |  |  |
| **2774161..2774445** | 2774306 | 285 | 52.5 | 0 |  |  |  |  |
|  |  |  |  |  | 2779036 | 2781078 | - | SAOUHSC_03006 |
| **2781174..2781480** | 2781314 | 307 | 38.1 | 6.699E-318 |  |  |  |  |

Table S3: Selection of ChIP-Seq peak with a p value of 1.10^-250^ or less.

mRNA genes appeared in the table with a yellow background whereas sRNA genes appeared in the table with a blue background. Green highlighting was used when ChIP peak could be associated to sRNA and mRNA genes.
