# Supplementary material for: Expanding the Staphylococcus aureus SarA Regulon to Small RNAs
Source: mSystems. 2021 Oct 12;6(5):e00713-21. doi: 10.1128/mSystems.00713-21 (PMC8510525; doi:10.1128/mSystems.00713-21)
Supplement: TABLE S4 [file msystems.00713-21-st004.docx]

| Gene group and annotation | Gene name | Assignment |
| --- | --- | --- |
| **Transport and binding proteins & protein fate** | | |
| SAOUHSC_00067 *lctP1* L-lactate permease  SAOUHSC_00105 *phnD* Phosphonate ABC transporter substrate-binding protein  SAOUHSC_00106 HP Putative ABC-type transporter  SAOUHSC_00253 HP Hypothetical protein  SAOUHSC_00257 *esxA* WXG100 family type VII secretion effector SAOUHSC_00282 *brnQ2* Branched-chain amino acid transport system II carrier protein SAOUHSC_00358 HP Hypothetical protein  SAOUHSC_00416 HP Hypothetical protein  SAOUHSC_00548 HP Hypothetical protein  SAOUHSC_00556 *proP* Proline/betaine transporter  SAOUHSC_00695 HP Hypothetical protein  SAOUHSC_00698 HP Hypothetical protein  SAOUHSC_00769 *secA* Preprotein translocase subunit SecA  SAOUHSC_00828 HP L-lysine exporter  SAOUHSC_00844 *metQ1* Signal peptidase IB  SAOUHSC_00923 *opp-3B/oppB* Oligopeptide transport system permease SAOUHSC_00928 *opp-4A* Oligopeptide ABC transporter substrate-binding protein SAOUHSC_00937 *pepF* Oligoendopeptidase F  SAOUHSC_00949 HP Sodium/alanine symporter family protein  SAOUHSC_00988 *sspA* Glutamyl endopeptidase  SAOUHSC_00994 *atl* Bifunctional autolysin  SAOUHSC_01180 HP Hypothetical protein SAOUHSC_01311 ABC transporter ATP-binding protein SAOUHSC_01312 HP Hypothetical protein  SAOUHSC_01346 *opuD1* Glycine betaine transporter  SAOUHSC_01383 HP Hypothetical protein  SAOUHSC_01406 *acyP* Acylphosphatase  SAOUHSC_01411 *brnQ3* Branched-chain amino acid transport system II carrier protein SAOUHSC_01447 *ebh* Hypothetical protein  SAOUHSC_01779 *tig* Trigger factor  SAOUHSC_01942 *splA* Serine protease  SAOUHSC_01972 *prsA* Protein export protein PrsA  SAOUHSC_01992 PTS system transporter subunit IIC domain-containing protein SAOUHSC_02119 *putP* High affinity proline permease  SAOUHSC_02145 HP Hypothetical protein  SAOUHSC_02244 Succinyl-diaminopimelate desuccinylase SAOUHSC_02247 *ktrB* Potassium uptake protein  SAOUHSC_02444 *opuD2* BCCT family osmoprotectant transporter  SAOUHSC_02472 HP Hypothetical protein  SAOUHSC_02549 *modA* Molybdenum ABC transporter substrate-binding protein SAOUHSC_02557 Urea transporter  SAOUHSC_02622 *gltS* Sodium/glutamate symporter  SAOUHSC_02668 HP Hypothetical protein  SAOUHSC_02690 HP Hypothetical protein  SAOUHSC_02719 HP Hypothetical protein  SAOUHSC_02752 HP Hypothetical protein SAOUHSC_02754 ABC transporter ATP-binding protein SAOUHSC_02815 HP Hypothetical protein  SAOUHSC_02820 HP ABC transporter ATP-binding protein  SAOUHSC_02832 HP Hypothetical protein | | |
| **Amino acids biosynthesis** | | |
| SAOUHSC_00144 *ausA* Non-ribosomal peptide synthetase  SAOUHSC_00466 *ipk* 4-diphosphocytidyl-2C-methyl-D-erythritol kinase  SAOUHSC_00832 *aroD* 3-dehydroquinase SAOUHSC_00914 2-isopropylmalate synthase SAOUHSC_01128 *argF* Ornithine carbamoyltransferase | | |

| SAOUHSC_01307 SAOUHSC_01483 SAOUHSC_01597 SAOUHSC_01617 SAOUHSC_01776 SAOUHSC_01852 SAOUHSC_02281 SAOUHSC_02830 | HP  *aroC proC argR hemA aroA2 ilvD ddh* | Hypothetical protein Chorismate synthase  Pyrroline-5-carboxylate reductase Arginine repressor  Glutamyl-tRNA reductase  Bifunctional 3-deoxy-7-phosphoheptulonate synthase/chorismate Dihydroxy-acid dehydratase  D-lactate dehydrogenase | mutase |
| --- | --- | --- | --- |
| **Biosynthesis of cofactors** | | | |
| SAOUHSC_00284 SAOUHSC_00499 | HP  *pdxS* | Hypothetical protein  Pyridoxal biosynthesis lyase PdxS | |
| **DNA metabolism** | | | |
| SAOUHSC_00507 | *radA* | DNA repair protein RadA | |
| SAOUHSC_00819 | *cspC* | Hypothetical protein | |
| SAOUHSC_00935 | *trfA* | Adaptor protein MecA | |
| SAOUHSC_01224 | *xerC* | Site-specific recombinase | |
| SAOUHSC_01272 | *mutS* | DNA mismatch repair protein MutS | |
| SAOUHSC_01343 | *sbcC* | Exonuclease | |
| SAOUHSC_01403 | *cspA* | Cold shock protein | |
| SAOUHSC_01466 | *recU* | Holliday junction-specific endonuclease | |
| SAOUHSC_01470 | *dnaD* | Hypothetical protein | |
| SAOUHSC_01638 | *comGE* | Hypothetical protein | |
| SAOUHSC_01673 | *phoH* | Hypothetical protein | |
| SAOUHSC_01744 | *recJ* | Single-stranded-DNA-specific exonuclease | |
| SAOUHSC_02316 | *cshA* | DEAD-box ATP dependent DNA helicase | |
| SAOUHSC_02621 | *aag* | DNA-3-methyladenine glycosylase | |
| SAOUHSC_02791 |  | Pyrophosphohydrolase | |
| SAOUHSC_02911 | *queH* | Adenine nucleotide alpha hydrolases superfamily protein | |
| **Energy metabolism** | | | |
| SAOUHSC_00088 | *galE* | UDP-glucose 4-epimerase | |
| SAOUHSC_00555 |  | Haloacid dehalogenase-like hydrolase | |
| SAOUHSC_00608 | *adh1* | Alcohol dehydrogenase | |
| SAOUHSC_00619 | HP | Hypothetical protein | |
| SAOUHSC_00756 |  | Glycerate kinase | |
| SAOUHSC_00906 | HP | Hypothetical protein | |
| SAOUHSC_01337 | *tkt* | Transketolase | |
| SAOUHSC_01415 | HP | Hypothetical protein | |
| SAOUHSC_01452 | *ald1* | Alanine dehydrogenase | |
| SAOUHSC_01901 | *tal* | Putative translaldolase | |
| SAOUHSC_02126 | *purB* | Adenylosuccinate lyase | |
| SAOUHSC_02152 | *pmtC* | ABC transporter ATP-binding protein | |
| SAOUHSC_02550 | *fdhD* | Formate dehydrogenase accessory protein | |
| SAOUHSC_02829 | *frp* | NAD(P)H-flavin oxidoreductase | |
| **Carbohydrates** | | | |
| SAOUHSC_00113 | *adhE* | Bifunctional acetaldehyde-CoA/alcohol deshydrogenase | |
| SAOUHSC_00712 | HP | Aldo/keto reductase | |
| SAOUHSC_01599 | *zwf* | Glucose-6-phosphate 1-dehydrogenase | |
| SAOUHSC_01807 | *pfkA* | 6-phosphofructokinase | |
| SAOUHSC_01810 |  | NADP-dependent malic enzyme | |
| SAOUHSC_01845 | *fhs* | Formate-tetrahydrofolate ligase | |
| SAOUHSC_02612 | *rpiA* | Ribose-5-phosphate isomerase A | |
| SAOUHSC_02703 | *gpmA* | 2,3-bisphosphoglycerate-dependent phosphoglycerate mutase | |
| SAOUHSC_02793 | *pgcA* | Hypothetical protein | |
| SAOUHSC_02926 | *fdaB* | Fructose-1,6-bisphosphate aldolase | |

| **Fatty acid and phospholipid metabolism** | | |
| --- | --- | --- |
| SAOUHSC_00300 | *geh* | Lipase |
| SAOUHSC_00920 | *fabH* | 3-oxoacyl-ACP synthase III |
| SAOUHSC_01596 | HP | Hypothetical protein |
| SAOUHSC_03006 | *gehA* | Lipase |
| **Central intermediary metabolism** | | |
| SAOUHSC_00577 SAOUHSC_02558 | *mvaK1 ureA* | Mevalonate kinase Urease subunit gamma |
| **Cell envelope & capsule** | | |
| SAOUHSC_00114 | *capA/cap5A* | Capsular polysaccharide biosynthesis protein |
| SAOUHSC_00143 | HP | hypothetical protein |
| SAOUHSC_00691 | *uppP* | Undecaprenyl pyrophosphate phosphatase |
| SAOUHSC_00728 | *ltaS* | Hypothetical protein |
| SAOUHSC_00762 | *tagO* | Hypothetical protein |
| SAOUHSC_00847 | *sufC* | ABC transporter ATP-binding protein |
| SAOUHSC_00953 | *ugtP* | Diacylglycerol glucosyltransferase |
| SAOUHSC_00954 | *murE* | UDP-N-acetylmuramoylalanyl-D-glutamate--L-lysine ligase |
| SAOUHSC_01374 | *femB* | Methicillin resistance factor |
| SAOUHSC_01828 |  | GAF domain-containing protein |
| SAOUHSC_01900 | *spdA* | hypothetical protein |
| SAOUHSC_02012 | *sgtB* | glycosyltransferase |
| SAOUHSC_02365 | *murA2* | UDP-N-acetylglucosamine 1-carboxyvinyltransferase |
| SAOUHSC_02576 | *ssaA* | Secretory antigen SsaA (LysM domain-containing protein) |
| SAOUHSC_02589 | HP | Hypothetical protein |
| SAOUHSC_02638 | HP | Hypothetical protein |
| SAOUHSC_03000 | *cap1A* | Capsular polysaccharide biosynthesis protein CapA |
| **Cellular processes** | | |
| SAOUHSC_00051 | *plc* | 1-phosphatidylinositol phosphodiesterase |
| SAOUHSC_00299 | HP | Hypothetical protein |
| SAOUHSC_00584 | HP | Hypothetical protein |
| SAOUHSC_00671 |  | Secretory antigen SsaA-like protein |
| SAOUHSC_00908 | *cdr* | Coenzyme A disulfide reductase |
| SAOUHSC_00948 | *cozEa* | Hypothetical protein |
| SAOUHSC_01314 | HP | Hypothetical protein |
| SAOUHSC_01742 | *relA* | GTP pyrophosphokinase |
| SAOUHSC_01822 | *tpx* | 2-Cys peroxiredoxin |
| SAOUHSC_01827 | *ezrA* | Septation ring formation regulator EzrA |
| SAOUHSC_01902 | HP | Hypothetical protein |
| SAOUHSC_01953 | *epiA* | Gallidermin superfamily EpiA protein |
| SAOUHSC_01973 | *cbf1* | 3'-5' exoribonuclease YhaM |
| SAOUHSC_02280 | *tsaE* | Hypothetical protein |
| SAOUHSC_02553 | HP | Hypothetical protein |
| SAOUHSC_02885 | *oatA* | Hypothetical protein |
| SAOUHSC_02973 | HP | Cell division protein ZipA |
| SAOUHSC_1342a |  | Large-conductance mechanosensitive channel |
| **Regulatory functions** | | |
| SAOUHSC_00070 | *sarS* | Accessory regulator-like protein |
| SAOUHSC_00620 | *sarA* | Accessory regulator A |
| SAOUHSC_00674 | *sarX* | Staphylococcal accessory regulator family |
| SAOUHSC_00675 | HP | Hypothetical protein |
| SAOUHSC_00694 | *mgrA* | Staphylococcal accessory regulator family |
| SAOUHSC_00818 | *nuc* | Thermonuclease |
| SAOUHSC_00913 | *lysR* | LysR family regulatory protein |
| SAOUHSC_00934 | *spxA* | Transcriptional regulator Spx |
| SAOUHSC_00992 | *atlR* | MarR family transcriptional regulator |
| SAOUHSC_00997 | HP | Hypothetical protein |

| SAOUHSC_01420 | *arlR* | DNA-binding response regulator |
| --- | --- | --- |
| SAOUHSC_01464 | HP | Hypothetical protein |
| SAOUHSC_01879 | *rot* | Virulence factor regulator protein |
| SAOUHSC_01850 | *ccpA* | Catabolite control protein A |
| SAOUHSC_01891 | *arsR* | Arsenate operon regulator |
| SAOUHSC_02300 | *rsbV* | STAS domain-containing protein |
| SAOUHSC_02390 | HP | Hypothetical protein |
| SAOUHSC_02461 |  | MerR family transcriptional regulator |
| SAOUHSC_02566 | *sarR* | Staphylococcal accessory regulator family |
| SAOUHSC_02570 |  | AraC family transcriptional regulator |
| SAOUHSC_02669 | *sarZ* | Staphylococcal accessory regulator family |
| SAOUHSC_02799 | *sarT* | Accessory regulator T |
| SAOUHSC_02800 | *sarU* | Staphylococcal accessory regulator family |
| SAOUHSC_02819 | HP | Hypothetical protein |
| **Virulence** | | |
| SAOUHSC_00069 | *spa* | Protein A |
| SAOUHSC_00094 | *sasD* | Hypothetical protein |
| SAOUHSC_00192 | *coa* | Staphylocoagulase |
| SAOUHSC_00399 | *ssl11* | Superantigen-like protein |
| SAOUHSC_00544 | *sdrC* | Fibrinogen-binding protein SdrC |
| SAOUHSC_00545 | *sdrD* | Fibrinogen-binding protein SdrD |
| SAOUHSC_00812 | *clfA* | Clumping factor |
| SAOUHSC_00817 | HP | Hypothetical protein |
| SAOUHSC_01110 | *ecb* | Fibrinogen-binding protein-like protein |
| SAOUHSC_01114 | *efb* | Fibrinogen-binding protein |
| SAOUHSC_01121 | *hla* | Alpha-hemolysin |
| SAOUHSC_01127 | *ssl14* | Superantigen-like protein |
| SAOUHSC_01501 | *ebpS* | Elastin binding protein |
| SAOUHSC_01705 |  | Enterotoxin family protein |
| SAOUHSC_01809 | *accD* | Acetyl-CoA carboxylase carboxyltransferase subunit beta |
| SAOUHSC_01873 | *sasC* | Hypothetical protein |
| SAOUHSC_01955 | *lukE* | Leukotoxin lukE |
| SAOUHSC_02127 | *scpA* | Staphopain thiol proteinase |
| SAOUHSC_02161 | *eap/map* | MHC class II analog protein |
| SAOUHSC_02243 | *lukH* | Leukocidin LukH |
| SAOUHSC_02571 | *ssaA* | Secretory antigen |
| SAOUHSC_02696 | *fmhA* | Methicillin resistance determinant protein (FemAB family) |
| SAOUHSC_02706 | *sbi* | Immunoglobulin G-binding protein |
| SAOUHSC_02709 | *hglC* | Leukocidin s subunit |
| SAOUHSC_02802 | *fnbB* | Fibronectin binding protein B |
| SAOUHSC_02963 | *clfB* | Clumping factor B |
| SAOUHSC_02971 | *aur* | Zinc metalloproteinase aureolysin |
| SAOUHSC_02972 | *isaB* | Immunodominant antigen B |
| SAOUHSC_02990 | *sasA* | Hypothetical protein |
| **Stress response** | | |
| SAOUHSC_01413 | HP | Hypothetical protein |
| **Iron acquisition and metabolism** | | |
| SAOUHSC_00746 | *sstA* | Hypothetical protein |
| SAOUHSC_00976 | HP | Heme ABC transporter |
| SAOUHSC_01079 | *isdB* | Cell surface receptor IsdB for hemoglobin and hemoglobin-haptoglobin complexes |
| SAOUHSC_01082 | *isdC* | Hypothetical protein |
| **Competence** | | |
| SAOUHSC_00961 | *comK1* | Competence transcription factor |
| **Transcription** | | |
| SAOUHSC_00811 | HP | Hypothetical protein |

| SAOUHSC_00892 SAOUHSC_00951 SAOUHSC_01251 SAOUHSC_01598 SAOUHSC_01897 SAOUHSC_02369 | HP HP  *pnpA rnz sigS rpoE* | Hypothetical protein Hypothetical protein  Polynucleotide phosphorylase/polyadenylase AtsA/ElaC family protein  Hypothetical protein  DNA-directed RNA polymerase subunit delta |
| --- | --- | --- |
| **Traduction** | | |
| SAOUHSC_01329 SAOUHSC_01493 SAOUHSC_R0003 | *rpsN2 rpsA rrsD* | 30S ribosomal protein S14 30S ribosomal protein S1 16S Ribosomal RNA |
| **Cofactors, Vitamins, Prosthetic Groups, Pigments** | | |
| SAOUHSC_01434 | *dfrA* | Dihydrofolate reductase |
| SAOUHSC_01774 | *hemC* | Porphobilinogen deaminase |
| SAOUHSC_01889 | *ribD* | Riboflavin biosynthesis protein RibD |
| SAOUHSC_02000 | *gsaB* | Glutamate-1-semialdehyde aminotransferase |
| SAOUHSC_02133 | *pncB* | Nicotinate phosphoribosyltransferase |
| **Energy metabolism** | | |
| SAOUHSC_01330 SAOUHSC_01356 SAOUHSC_01727 | *guaC glcT iscS* | Guanosine 5'-monophosphate oxidoreductase Transcription antiterminator  Hypothetical protein |
| **Regulation and Cell signaling** | | |
| SAOUHSC_00998 SAOUHSC_01359 SAOUHSC_01488 | *fmtA mprF* HP | Methicillin resistance protein FmtA Hypothetical protein  Hypothetical protein |
| **Protein synthesis** | | |
| SAOUHSC_00357 | HP | Hypothetical protein |
| SAOUHSC_00509 | *gltX* | Glutamyl-tRNA synthetase |
| SAOUHSC_00933 | *trpS* | Tryptophanyl-tRNA synthetase |
| SAOUHSC_01091 | HP | Hypothetical protein |
| SAOUHSC_01425 | HP | Hypothetical protein |
| SAOUHSC_01679 | *mtaB* | Hypothetical protein |
| SAOUHSC_01874 | HP | Hypothetical protein |
| SAOUHSC_01890 | HP | Hypothetical protein |
| SAOUHSC_01895 | HP | Hypothetical protein |
| SAOUHSC_02118 | *gatC* | Aspartyl/glutamyl-tRNA amidotransferase subunit C |
| **Quorum sensing and biofilm formation** | | |
| SAOUHSC_00673 SAOUHSC_02636 | *rbf tcaR* | Hypothetical protein |
| **Mobile element protein** | | |
| SAOUHSC_01993 | *tnp3* |  |
| **Nitrogen Metabolism** | | |
| SAOUHSC_02134 | *nos* | Nitric oxide synthase oxygenase subunit |
| **Phosphorus Metabolism** | | |
| SAOUHSC_02816 | HP | Hypothetical protein |
| **Respiration** | | |
| SAOUHSC_02582 | *fdhA* | Formate dehydrogenase subunit alpha |
| **RNA Metabolism** | | |

| SAOUHSC_01221 | *dprA* | Hypothetical protein |
| --- | --- | --- |
| **Signal transduction** | | |
| SAOUHSC_00242 | *rbsR* | Hypothetical protein |
| **Sulfur Metabolism** | | |
| SAOUHSC_01999 | HP | Hypothetical protein |
| **sRNA** | | |
| *srn_0380_teg140* | | |
| *srn_0765_tsr11* | | |
| *srn_0930_teg76* | | |
| *srn_0960_teg147* | | |
| *srn_1090_teg19as* | | |
| *srn_1210_sRNA106* | | |
| *srn_1270_teg44* | | |
| *srn_1330_sRNA118* | | |
| *srn_1470_sRNA129* | | |
| *srn_1510_rsaA* | | |
| *srn_1530_sRNA133* | | |
| *srn_1540_teg48* | | |
| *srn_1550_teg49* | | |
| *srn_1670_teg20as* | | |
| *srn_1680_sRNA142* | | |
| *srn_1690_sRNA143* | | |
| *srn_1910_rsaH* | | |
| *srn_1960_rsaOL* | | |
| *srn_1980_sRNA169* | | |
| *srn_2080_sau6851* | | |
| *srn_2125_tsr17* | | |
| *srn_2230_sprG2* | | |
| *srn_2270_sRNA192* | | |
| *srn_2310_sRNA194* | | |
| *srn_2420_sRNA201* | | |
| *srn_2460_teg58* | | |
| *srn_2510_sau6297* | | |
| *srn_2520_teg60* | | |
| *srn_2770_sau6904* | | |
| *srn_2780_sau6282* | | |
| *srn_2790_sRNA224* | | |
| *srn_2910_teg63* | | |
| *srn_2975_tsr25* | | |
| *srn_3040_sau50* | | |
| *srn_3090_sRNA245* | | |
| *srn_3360_sRNA267* | | |
| *srn_3500_sRNA277* | | |
| *srn_3510_teg74* | | |
| *srn_3520_sRNA279* | | |
| *srn_3610_sprC* | | |
| *srn_3630_sau69* | | |
| *srn_3950_teg16* | | |
| *srn_3980_sRNA324* | | |
| *srn_4205_tsr32* | | |
| *srn_4220_sRNA345* | | |
| *srn_4540_sprAs2* | | |
| *srn_4680_sau19* | | |
| *srn_4705_tsr37* | | |
| *srn_4980_teg32* | | |
| *srn_9020_sRNA52* | | |

| *srn_9335.1_tsr29 srn_9335_tsr29 srn_9340_sRNA287 srn_9480_sRNA334 srn_9510_sRNA390* | | |
| --- | --- | --- |
| **Unknown function** | | |
| SAOUHSC_00279 | HP | Hypothetical protein |
| SAOUHSC_00047 | HP | Hypothetical protein |
| SAOUHSC_00084 | HP | Hypothetical protein |
| SAOUHSC_00191 | HP | Hypothetical protein |
| SAOUHSC_00244 | HP | Hypothetical protein |
| SAOUHSC_00254 | HP | Hypothetical protein |
| SAOUHSC_00256 | HP | Hypothetical protein |
| SAOUHSC_00272 | HP | Hypothetical protein |
| SAOUHSC_00353 | HP | Hypothetical protein |
| SAOUHSC_00354 | *selX* | Hypothetical protein |
| SAOUHSC_00371 | HP | Hypothetical protein |
| SAOUHSC_00381a | HP | Hypothetical protein |
| SAOUHSC_00401 | HP | Hypothetical protein |
| SAOUHSC_00402 | *lpl3* | Hypothetical protein |
| SAOUHSC_00405 | *lpl9* | Hypothetical protein |
| SAOUHSC_00492 | HP | Hypothetical protein |
| SAOUHSC_00572 | HP | Hypothetical protein |
| SAOUHSC_00598 | HP | Hypothetical protein |
| SAOUHSC_00599 | HP | Hypothetical protein |
| SAOUHSC_00669 | *pitR* | Hypothetical protein |
| SAOUHSC_00678 | HP | Hypothetical protein |
| SAOUHSC_00692 | HP | Hypothetical protein |
| SAOUHSC_00701 | HP | Hypothetical protein |
| SAOUHSC_00717 | *saeP* | Hypothetical protein |
| SAOUHSC_00784 | HP | Hypothetical protein |
| SAOUHSC_00792 | HP | Hypothetical protein |
| SAOUHSC_00800 | HP | Hypothetical protein |
| SAOUHSC_00808 | HP | Hypothetical protein |
| SAOUHSC_00809 | HP | Hypothetical protein |
| SAOUHSC_00825 | HP | Hypothetical protein |
| SAOUHSC_00830 | HP | Hypothetical protein |
| SAOUHSC_00846 | HP | Hypothetical protein |
| SAOUHSC_00868 | *dltX* | Hypothetical protein |
| SAOUHSC_00911 | HP | Hypothetical protein |
| SAOUHSC_00917 | HP | Hypothetical protein |
| SAOUHSC_00919 | HP | Hypothetical protein |
| SAOUHSC_00940 | HP | Hypothetical protein |
| SAOUHSC_00941 | HP | Hypothetical protein |
| SAOUHSC_00962 | HP | Hypothetical protein |
| SAOUHSC_00969 | HP | Hypothetical protein |
| SAOUHSC_00971 | HP | Hypothetical protein |
| SAOUHSC_00972 | HP | Hypothetical protein |
| SAOUHSC_00975 | HP | Hypothetical protein |
| SAOUHSC_00991 | HP | Hypothetical protein |
| SAOUHSC_01005 | HP | Hypothetical protein |
| SAOUHSC_01112 | *flr* | Formyl peptide receptor-like 1 inhibitory protein |
| SAOUHSC_01113 | HP | Hypothetical protein |
| SAOUHSC_01120 | HP | Hypothetical protein |
| SAOUHSC_01122 | HP | Hypothetical protein |
| SAOUHSC_01131 | HP | Hypothetical protein |
| SAOUHSC_01152 | HP | Hypothetical protein |
| SAOUHSC_01289 | HP | Outer membrane assembly lipoprotein |
| SAOUHSC_01290 | HP | Hypothetical protein |
| SAOUHSC_01291 | HP | Hypothetical protein |

| SAOUHSC_01292 HP Hypothetical protein  SAOUHSC_01295 HP Hypothetical protein  SAOUHSC_01297 HP Hypothetical protein  SAOUHSC_01301 HP Hypothetical protein  SAOUHSC_01306 HP Hypothetical protein  SAOUHSC_01307a HP Hypothetical protein  SAOUHSC_01317 HP Hypothetical protein  SAOUHSC_01375 HP Hypothetical protein  SAOUHSC_01421 HP Hypothetical protein  SAOUHSC_01494 HP Hypothetical protein  SAOUHSC_01500 HP Hypothetical protein  SAOUHSC_01584 HP Hypothetical protein  SAOUHSC_01636 HP Hypothetical protein  SAOUHSC_01677 HP Hypothetical protein  SAOUHSC_01728 HP Hypothetical protein  SAOUHSC_01798 HP Hypothetical protein  SAOUHSC_01899 HP Hypothetical protein  SAOUHSC_01903 *crcB1* Camphor resistance protein CrcB  SAOUHSC_01917 HP Hypothetical protein  SAOUHSC_01918 HP Calcium-binding protein  SAOUHSC_01923 HP Hypothetical protein  SAOUHSC_01944 HP Hypothetical protein  SAOUHSC_01956 HP Pseudogene  SAOUHSC_01984 HP Hypothetical protein  SAOUHSC_01985 HP Hypothetical protein  SAOUHSC_02004 HP Hypothetical protein  SAOUHSC_02114 *dgkB* Lipid kinase  SAOUHSC_02131 HP Hypothetical protein  SAOUHSC_02146 HP Hypothetical protein  SAOUHSC_02246 *fhuD1* Hypothetical protein  SAOUHSC_02294 HP Hypothetical protein  SAOUHSC_02338 HP Hypothetical protein  SAOUHSC_02382 HP Hypothetical protein  SAOUHSC_02407 *dacA* Hypothetical protein  SAOUHSC_02443 *amaP* Hypothetical protein  SAOUHSC_02462 HP Hypothetical protein  SAOUHSC_02574 HP Hypothetical protein  SAOUHSC_02575 HP Hypothetical protein  SAOUHSC_02587 *spdB* hypothetical protein  SAOUHSC_02613 HP Hypothetical protein  SAOUHSC_02620 HP Hypothetical protein  SAOUHSC_02688 HP Hypothetical protein  SAOUHSC_02695 HP Hypothetical protein  SAOUHSC_02705 HP Hypothetical protein  SAOUHSC_02781 HP Hypothetical protein  SAOUHSC_02782 HP Hypothetical protein  SAOUHSC_02783 HP Hypothetical protein  SAOUHSC_02794 HP Hypothetical protein  SAOUHSC_02831 HP Hypothetical protein  SAOUHSC_02858 HP Hypothetical protein  SAOUHSC_02907 HP Hypothetical protein  SAOUHSC_02912 HP Hypothetical protein  SAOUHSC_02950 HP Hypothetical protein  SAOUHSC_A02331 HP Hypothetical protein |
| --- |

Table S4: Classification of SarA bound targets (ChIP-Seq peak with a p value of 1.10^-250^ or less) regarding to their function.
