# Supplementary material for: Expanding the Staphylococcus aureus SarA Regulon to Small RNAs
Source: mSystems. 2021 Oct 12;6(5):e00713-21. doi: 10.1128/mSystems.00713-21 (PMC8510525; doi:10.1128/mSystems.00713-21)
Supplement: TABLE S5 [file msystems.00713-21-st005.docx]

|  | | | **RNA-seq Data** | | **ChIP-seq Data** | | |
| --- | --- | --- | --- | --- | --- | --- | --- |
|  |  |  | **Differential expression HG003 Δ*sarA* vs HG003** | | Distance to the ATG (nt) | Score | p-value |
| **Gene group and an-tation** | **Gene name** | **Assignment** | **Exponential (2h)** | **Early stationnary**  **(4.5h)** |  |  |  |
|  |  |  | Fold-change | Fold-change |  |  |  |
| SAOUHSC_00047 | HP | Hypothetical protein | **- 3.5** | | 124 | 39.4 | 0 |
| SAOUHSC_00051 | *plc* | 1-phosphatidyli-sitol phosphodiesterase | **12.2 23.6** | | 41 | 38.3 | 1.59E-321 |
| SAOUHSC_00069 | *spa* | Protein A | **- 3.2** | | 151 | 39.4 | 0.00E+00 |
| SAOUHSC_00084 | HP | Hypothetical protein | **3.4 -** | | 61 |  |  |
| **SAOUHSC_00088*** | ***galE*** | **UDP-glucose 4-epimerase** | **75.5** | **43.02** | 85 | 33.2 | 7.09E-242 |
| SAOUHSC_00089 |  | Undecaprenyl-phosphate glucose phosphotransferase | **76.6** | **27.0** |  |  |  |
| SAOUHSC_00093 | *sodM* | Superoxide dismutase | **14.0** | **4.5** | 63 | 43.1 | 0 |
| SAOUHCS_00102 | *phnE1* | Phosphonates ABC transporter permease | **13.4** | **19.9** |  |  |  |
| SAOUHCS_00103 | *phnE2* | Phosphonates ABC transporter permease | **18.8** | **21.3** |  |  |  |
| SAOUHCS_00104 | *phnC* | Amino-acid ABC transporter ATP-binding protein | **39.1** | **23.0** |  |  |  |
| **SAOUHCS_00105** | ***phnD*** | **Phosphonate ABC transporter substrate binding protein** | **41.8** | **19.0** | 114 | 43.5 | 0.00E+00 |
| SAOUHSC_00106 | HP | Putative ABC-type transporter | **11.4 10.6** | | 115 | 43.5 | 0.00E+00 |
| SAOUHSC_00113 | *adhE* | Bifunctional acetaldehyde-CoA/alcohol deshydrogenase | **5.5 -** | | 114 | 40.2 | 0 |
| **SAOUHSC_00114** | ***capA/cap5A*** | **Capsular polysaccharide biosynthesis protein** | **5.2 -** | | 65 | 41.2 | 0 |
| SAOUHSC_00115 | *capB* | Capsular polysaccharide biosynthesis protein | **5.3 -** | |  |  |  |
| SAOUHSC_00116 | *capC* | Capsular polysaccharide biosynthesis protein | **4.3 -** | |  |  |  |
| SAOUHSC_00117 | *capD* | Capsular polysaccharide biosynthesis protein | **6.8 -** | |  |  |  |
| SAOUHSC_00118 | *capE* | Capsular polysaccharide biosynthesis protein | **3.7 3.2** | |  |  |  |
| SAOUHSC_00119 | *capF* | Capsular polysaccharide biosynthesis protein | **3.5 -** | |  |  |  |
| SAOUHSC_00144 | *ausA* | Non-ribosomal peptide synthetase | **3.3 -** | | 400 | 34.8 | 1.91E-265 |
| SAOUHSC_00249 |  | ABC transporter | **5.6** | **3.3** |  |  |  |
| SAOUHSC_00250 |  | Hypothetical protein | **6.6** | **4.5** |  |  |  |
| SAOUHSC_00251 |  | Hypothetical protein | **7.6** | **4.6** |  |  |  |
| **SAOUHSC_00253** | **HP** | **Hypothetical protein** | **17.1** | **8.9** | 48 | 41.5 | 0.00E+00 |
| SAOUHSC_00254 | HP | Hypothetical protein | **11.0 10.4** | | 223 | 41.5 | 0 |
| SAOUHSC_00256 | HP | Hypothetical protein | **7.2 3.8** | | 51 | 37.1 | 2.51E-301 |
| SAOUHSC_00257 | *esxA* | WXG100 family type VII secretion effector | **18.0 8.9** | | 197 | 37.1 | 2.51E-301 |
| SAOUHSC_00282 | *brnQ* | Branched-chain amino-acid transport system II carrier protein | **- 3.4** | | 550 | 37.0 | 6.50E-300 |
| SAOUHSC_00284 | HP | Hypothetical protein | **- 3.4** | | 64 | 37.0 | 6.50E-300 |
| SAOUHSC_00300 | *geh* | Lipase | **5.2 -** | | 171 | 49.5 | 0 |
| SAOUHSC_00381a | HP |  | **3.3 5.0** | | 38 | 42.9 | 0 |
| SAOUHSC_00544 | *sdrC* | Fibrinogen-binding protein SdrC | **6.8 5.4** | | 74 | 49.3 | 0 |
| SAOUHSC_00545 | *sdrD* | Fibrinogen-binding protein SdrD | **8.0 5.2** | | 142 | 41.5 | 0 |
| SAOUHSC_00555* |  | Haloacid dehalogenase-like hydrolase | **15.2 7.0** | | 33 | 45.3 | 0 |
| SAOUHSC_00577 | *mvaK1* | Mevalonate kinase | **- 3.0** | | 455 | 42.7 | 0 |
| SAOUHSC_00674 | *sarX* | Staphylococcal accessory regulator family | **4.1 3.7** | | 85 | 35.9 | 4.89E-282 |
| SAOUHSC_00694 | *mgrA* | Staphylococcal accessory regulator family | **- 3.8** | | 157 | 46.6 | 0 |
| SAOUHSC_00712 | HP | Aldo/keto reductase | **- 3.2** | | 140 | 38.4 | 2.17E-322 |
| SAOUHSC_00808 | HP |  | **3.7 -** | | 150 | 39.5 | 0 |
| SAOUHSC_00812 | *clfA* | Clumping factor | **- 3.6** | | 97 | 34.9 | 1.48E-267 |
| SAOUHSC_00818 | *nuc* | Thermonuclease | **130.1 269.4** | | 176 | 42.7 | 0 |
| SAOUHSC_00828 | HP | L-lysine exporter | **- 3.1** | | 111 | 35.9 | 3.02E-282 |
| SAOUHSC_00842 | *metN1* | Methionine ABC transporter | **-** | **3.5** |  |  |  |

| SAOUHSC_00843  **SAOUHSC_00844** | *metP1*  ***metQ1*** | Methionine ABC transporter  **Methionine ABC transporter** | **-**  **-** | **4.4**  **5.1** | 6 | 35.0 | 2.64E-269 |
| --- | --- | --- | --- | --- | --- | --- | --- |
| SAOUHSC_00911  SAOUHSC_00913* | HP  *lysR* | Hypothetical protein  LysR family regulatory protein | **4.8**  **65.1** | **3.3**  **42.8** | 207  58 | 53.6  50.4 | 0  0 |
| **SAOUHSC_00923** | ***opp-3B*** | **Oligopeptide transport system permease** | **-** | **3.2** | 41 | 43.7 | 0 |
| SAOUHSC_00924 | *opp-3C* | Oligopeptide transport system permease | **-** | **4.1** |  |  |  |
| SAOUHSC_00925 | *opp-3D* | Oligopeptide transport system permease | **-** | **3.8** |  |  |  |
| SAOUHSC_00926 | *opp-3F* | Oligopeptide transport system permease | **-** | **3.8** |  |  |  |
| SAOUHSC_00927 | *opp-3A* | Oligopeptide ABC transporter | **-** | **3.5** |  |  |  |
| SAOUHSC_00949 | HP | Sodium/alanine symporter family protein | **- 8.7** | | 144 | 34.9 | 1.03E-266 |
| SAOUHSC_00961* | *comK1* | Competence transcription factor | **15.15 9.4** | | 111 | 46.7 | 0 |
| SAOUHSC_00971 | HP | Hypothetical protein | **5.1 5.2** | | 159 | 46.9 | 0 |
| SAOUHSC_00975* | HP | Hypothetical protein | **4.74 26.62** | | 440 | 47.8 | 0 |
| SAOUHSC_00976 | HP | Heme ABC transporter | **4.7 26.6** | | 144 | 47.8 | 0 |
| SAOUHSC_00987 | *sspB* | Cysteine protease | **-** | **26.1** |  |  |  |
| **SAOUHSC_00988** | ***sspA*** | **Glutamyl endopeptidase** | **-** | **34.2** | 134 | 46.2 | 0 |
| SAOUHSC_00992 | *atlR* | MarR family transcriptional regulator | **17.3 28.7** | | 77 | 38.3 | 2.88E-321 |
| SAOUHSC_01005 | HP | Hypothetical protein | **- 4.8** | | 135 | 37.5 | 2.22E-307 |
| SAOUHSC_01079 | *isdB* | Cell surface receptor IsdB for hemoglobin and hemoglobin-haptoglobin complexes | **5.8 3.5** | | 119 | 48.4 | 0.00E+00 |
| SAOUHSC_01113 | HP | Hypothetical protein | **4.2 -** | | 247 | 52.3 | 0 |
| SAOUHSC_01121 | *hla* | Alpha-hemolysin | **5.6 -** | | 392 | 50.8 | 0 |
| SAOUHSC_01180 | HP | Hypothetical protein | **3.1 3.1** | | 245 | 48.3 | 0 |
| SAOUHSC_01289 | HP | Outer membrane assembly lipoprotein | **23.4 13.1** | | 4 | 46.2 | 0 |
| SAOUHSC_01290 | HP | Hypothetical protein | **5.5 4.2** | | 96 | 34.9 | 4.79E-267 |
| SAOUHSC_01291 | HP | Hypothetical protein | **4.8 3.5** | | 385 | 46.3 | 0 |
| SAOUHSC_01292 | HP | Hypothetical protein | **4.4 -** | | 83 | 41.7 | 0 |
| SAOUHSC_01295 | HP | Hypothetical protein | **4.3 3.6** | | 31 | 38.4 | 1E-323 |
| SAOUHSC_01450 |  | Amino-acid transporter | **15.3** | **28.1** |  |  |  |
| SAOUHSC_01451 | *ilvA1* | Threonine dehydratase | **16.3** | **32.0** |  |  |  |
| **SAOUHSC_01452*** | ***ald1*** | **Alanine dehydrogenase** | **16.7** | **38.3** | 197 | 52.1 | 0 |
| SAOUHSC_01584 | HP | Hypothetical protein | **3.1 -** | | 125 | 41.0 | 0 |
| SAOUHSC_01744 | *recJ* | Single-stranded-DNA-specific exonuclease | **- 3.1** | | 52 | 38.6 | 0 |
| SAOUHSC_01798 |  | Hypothetical protein | **4.9 -** | | 166 | 46.8 | 0 |
| SAOUHSC_01845 | *fhs* | Formate-tetrahydrofolate ligase | **- 4.8** | | 172 | 34.1 | 1.09E-254 |
| SAOUHSC_01899 | HP | Hypothetical protein | **- 3.7** | | 73 | 36.0 | 4.56E-284 |
| SAOUHSC_01917 | HP | Hypothetical protein | **6.3 5.4** | | 55 | 45.8 | 0 |
| SAOUHSC_01918 | HP | Calcium-binding protein | **- 3.3** | | 248 | 45.8 | 0 |
| SAOUHSC_01923 | HP | Hypothetical protein | **6.2 4.0** | | 73 | 44.5 | 0 |
| SAOUHSC_01938 | *splD* | Serine protease | **6.0** | **29.2** |  |  |  |
| SAOUHSC_01939 | *splC* | Serine protease | **7.1** | **36.9** |  |  |  |
| SAOUHSC_01941 | *splB* | Serine protease | **17.5** | **52.4** |  |  |  |
| **SAOUHSC_01942** | ***splA*** | **Serine protease** | **24.3** | **62.3** | 93 | 49.3 | 0 |
| SAOUHSC_01944 | HP | Hypothetical protein | **-** | **9.4** | 91 | 41.9 | 0 |
| SAOUHSC_01954 | *lukD* | Leukotoxin LukD | **6.4** | **10.1** |  |  |  |
| **SAOUHSC_01955** | ***lukE*** | **Leukotoxin LukE** | **9.5** | **11.6** | 101 | 49.7 | 0 |
| SAOUHSC_01956 | HP | Pseudogene | **-** | **3.5** | 114 | 49.9 | 0 |
| SAOUHSC_01972 | *prsA* | Protein export protein | **4.0** | **3.8** | 250 | 51.0 | 0 |
| SAOUHSC_02127* | *scpA* | Staphopain thiol proteinase | **126.7** | **57.2** | 221 | 51.0 | 0 |
| SAOUHSC_02151 |  | Hypothetical protein | **-** | **5.0** |  |  |  |

| **SAOUHSC_02152** | ***pmtC*** | **ABC transporter ATP-binding protein** | **-** | **6.2** | 68 | 37.8 | 1.334E-313 |
| --- | --- | --- | --- | --- | --- | --- | --- |
| SAOUHSC_02241 | *lukG* | Leukocidin LukG | **6.0** | **5.7** |  |  |  |
| **SAOUHSC_02243** | ***lukH*** | **Leukocidin LukH** | **6.9** | **5.9** | 197 | 41.4 | 0 |
| SAOUHSC_02247 | *ktrB* | Potassium uptake protein | **10.1** | **7.2** | 85 | 43.2 | 0 |
| **SAOUHSC_02281** | ***ilvD*** | **Dihydroxy-acid dehydratase** | **-** | **19.2** | 415 | 44.7 | 0 |
| SAOUHSC_02282 | *ilvB* | Acetolactate synthase large subunit | **-** | **17.8** |  |  |  |
| SAOUHSC_02283 | *ilvH* | Acetolactate synthase 1 regulatory subunit | **-** | **15.1** |  |  |  |
| SAOUHSC_02557 |  | Urea transporter | **-** | **5.0** | 140 | 48.5 | 0 |
| **SAOUHSC_02558** | ***ureA*** | **Urease subunit gamma** | **-** | **4.4** | 135 | 48.5 | 0 |
| SAOUHSC_02559 | *ureB* | Urease subunit beta | **-** | **3.4** |  |  |  |
| SAOUHSC_02561 | *ureC* | Urease subunit alpha | **-** | **3.7** |  |  |  |
| SAOUHSC_02569 | *sarY* | Staphylococcal accessory regulator family | **8.8** | **5.6** |  |  |  |
| **SAOUHSC_02570** |  | **AraC family transcriptional regulator** | **11.4** | **6.0** | 161 | 52.8 | 0 |
| SAOUHSC_02576 | *ssaA* | Secretory antigen | **5.5** | **6.7** | 195 | 35.7 | 6.62E-280 |
| SAOUHSC_02622 | *gltS* | Sodium/glutamate symporter | **3.4** | **3.6** | 138 | 35.2 | 7.06E-272 |
| SAOUHSC_02667 | *gltT* | Proton/glutamate symport protein | **-** | **4.7** |  |  |  |
| **SAOUHSC_02668** | **HP** | **Hypothetical protein** | **-** | **3.1** | 217 | 38.0 | 1.138E-316 |
| SAOUHSC_02696* | *fmhA* | Methicillin resistance determinant protein (FemAB family) | **38.9** | **21.0** | 99 | 41.7 | 0 |
| SAOUHSC_02706 | *sbi* | Immu-globulin G-binding protein | **9.3** | **13.3** | 43 | 41.9 | 0 |
| **SAOUHSC_02709** | ***hglC*** | **Leukocidin s subunit** | **-** | **11.2** | 358 | 40.9 | 0 |
| SAOUHSC_02710 | *hlgB* | Leukocidin f subunit | **-** | **9.8** |  |  |  |
| SAOUHSC_02782 | HP | Hypothetical protein | **- 5.9** | | 256 | 39.9 | 0 |
| SAOUHSC_02820* | HP | ABC transporter ATP-binding protein | **23.4 44.4** | | 82 | 43.3 | 0 |
| SAOUHSC_02858 | HP | Hypothetical protein | **- 9.3** | | 48 | 35.3 | 1.00E-273 |
| SAOUHSC_02911 | *queH* | Adenine nucleotide alpha hydrolases superfamily protein | **3.3 -** | | 60 | 45.0 | 0 |
| SAOUHSC_02950 | HP | Hypothetical protein | **- 11.3** | | 75 | 38.5 | 0 |
| SAOUHSC_02963 | *clfB* | Clumping factor B | **- 5.6** | | 75 | 37.6 | 4.244E-310 |
| SAOUHSC_02971 | *aur* | Zinc metalloproteinase aureolysin | **467.8 162.0** | | 138 | 46.0 | 0 |
| SAOUHSC_02973 | HP | Cell division protein ZipA | **5.7 -** | | 162 | 41.2 | 0 |
| SAOUHSC_02998 | *cap1C* | Capsular polysaccharide biosynthesis protein Cap5C | **90.6** | **26.5** |  |  |  |
| SAOUHSC_02999 | *capB1* | Capsular polysaccharide biosynthesis protein Cap5B | **152.0** | **42.4** |  |  |  |
| **SAOUHSC_03000** | ***cap1A*** | **Capsular polysaccharide biosynthesis protein CapA** | **213.9** | **56.6** | 145 | 44.5 | 0 |
| SAOUHSC_03006 | *gehA* | Lipase | **-** | **3.1** | 236 | 38.1 | 6.699E-318 |
| **sRNA** |  |  |  | | Distance to the 5' (nt) | Score | p-value |
| *srn_0765_tsr11* |  |  | **- 3.3** | | 542 | 37.0 | 6.5E-300 |
| *srn_0860_rsaOB** |  |  | **- 10.5** | | 88 | 24.8 | 1.09E-135 |
| *srn_0930_teg76* |  |  | **- 3.8** | | 113 | 38.8 | 0 |
| *srn_1530_sRNA133* |  |  | **3.1 -** | | 126 | 51.0 | 0 |
| *srn_1550_teg49* |  |  | **3.7 30.5** | | 149 | 37.8 | 2.952E-313 |
| *srn_2230_sprG2* |  |  | **15.5 9.0** | | 54 | 33.7 | 9.01E-249 |
| *srn_2780_sau6282* |  |  | **- 10.3** | | (+) 39 | 36.9 | 4.52E-299 |
| *srn_3500_sRNA277* |  |  | **- 3.2** | | 7 | 52.3 | 0 |
| *srn_9335_tsr29** |  |  | **3.1 5.5** | | 113 | 40.5 | 0 |
| *srn_3610_sprC* |  |  | **7.1 8.9** | | 17 | 49.9 | 0 |
| *srn_9340_sRNA287* |  |  | **13.9 24.8** | | 51 | 54.8 | 0 |
| *srn_3630_sau69* |  |  | **4.3 3.5** | | 111 | 51.0 | 0 |
| *srn_3950_teg16** |  |  | **- 21.8** | | 106 | 44.7 | 0 |
| *srn_4680_sau19* |  |  | **- 4.6** | | 32 | 39.9 | 0 |

| *srn_4980_teg32* |  |  | **7.9 12.0** | 117 | 46.4 | 0 |
| --- | --- | --- | --- | --- | --- | --- |

Table S5: **Complete ChIP-Seq/ RNA-Seq shared genes (SarA direct targets).**

When operon organization were available it appeared in the table with a grey background, the first gene of the operon marked in bold.

Asterix mark was used to highlight genes selected for experimental investigation of SarA direct regulation. Written in green are SarA activated genes and written in red are SarA repressed genes.
