# Supplementary material for: Expanding the Staphylococcus aureus SarA Regulon to Small RNAs
Source: mSystems. 2021 Oct 12;6(5):e00713-21. doi: 10.1128/mSystems.00713-21 (PMC8510525; doi:10.1128/mSystems.00713-21)
Supplement: FIG S3 [file msystems.00713-21-sf003.pdf]

SAOUHSC_00088 (*galE*)

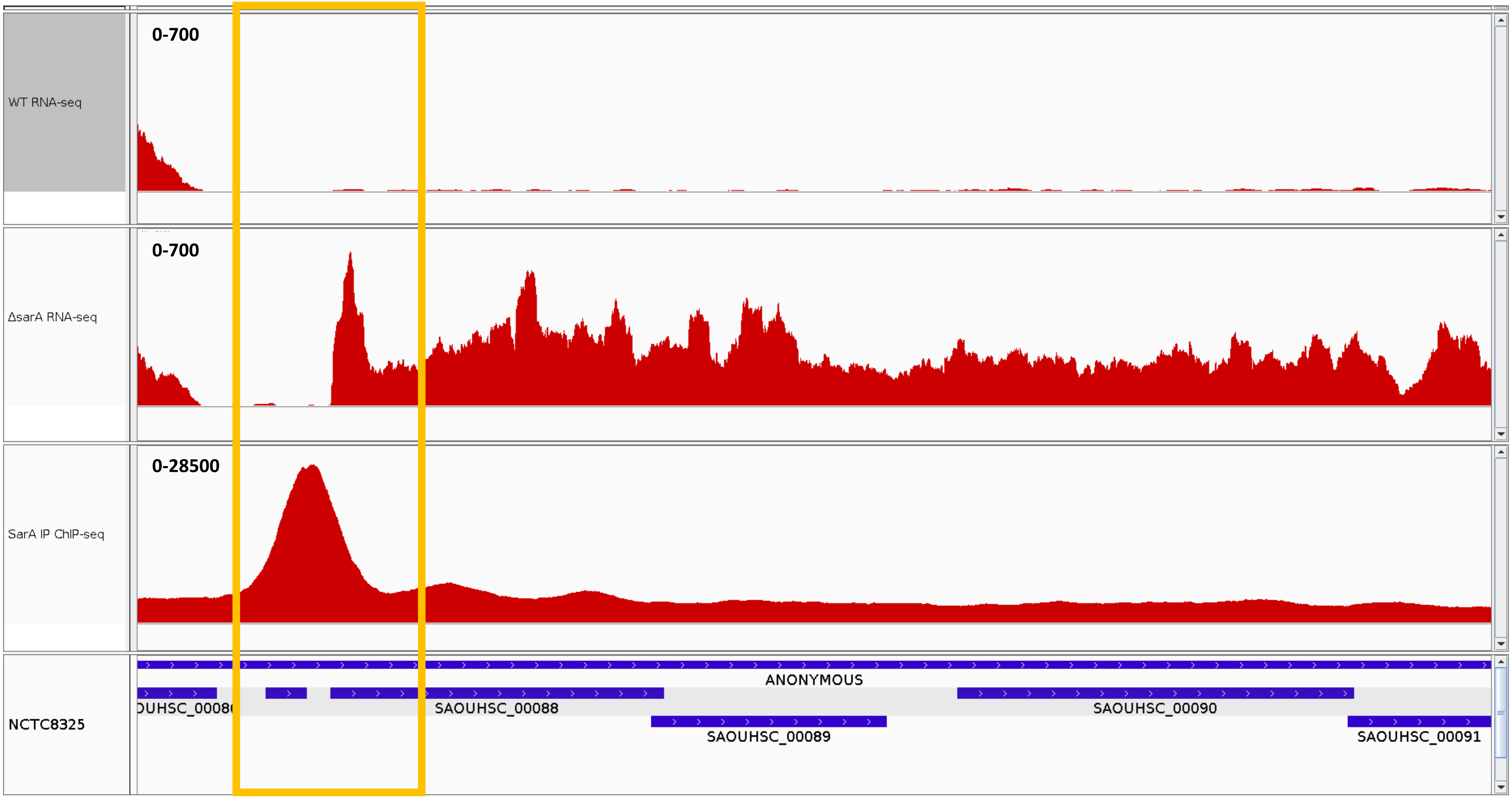

SAOUHSC_00555

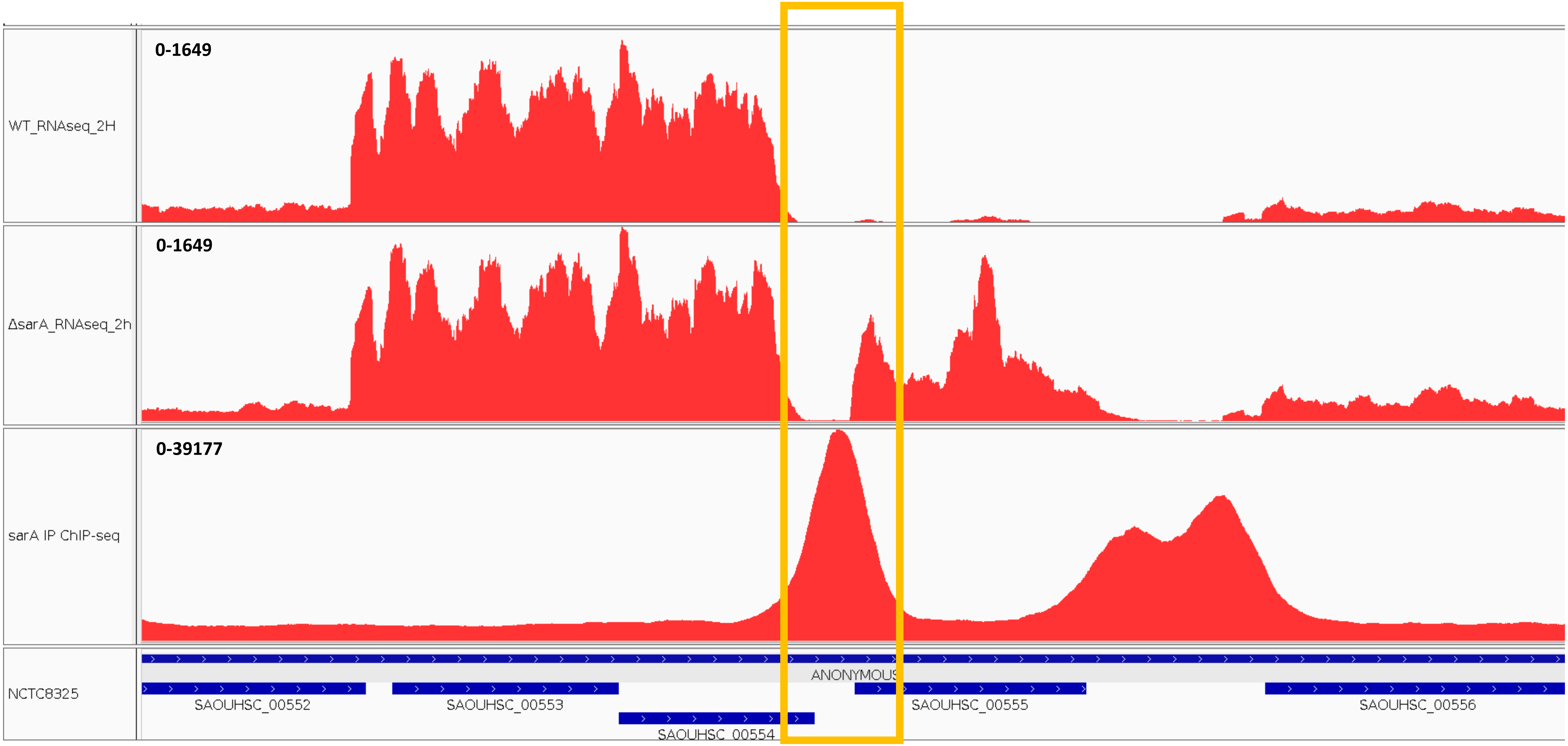

SAUOHSC-00913 (*lysR)*

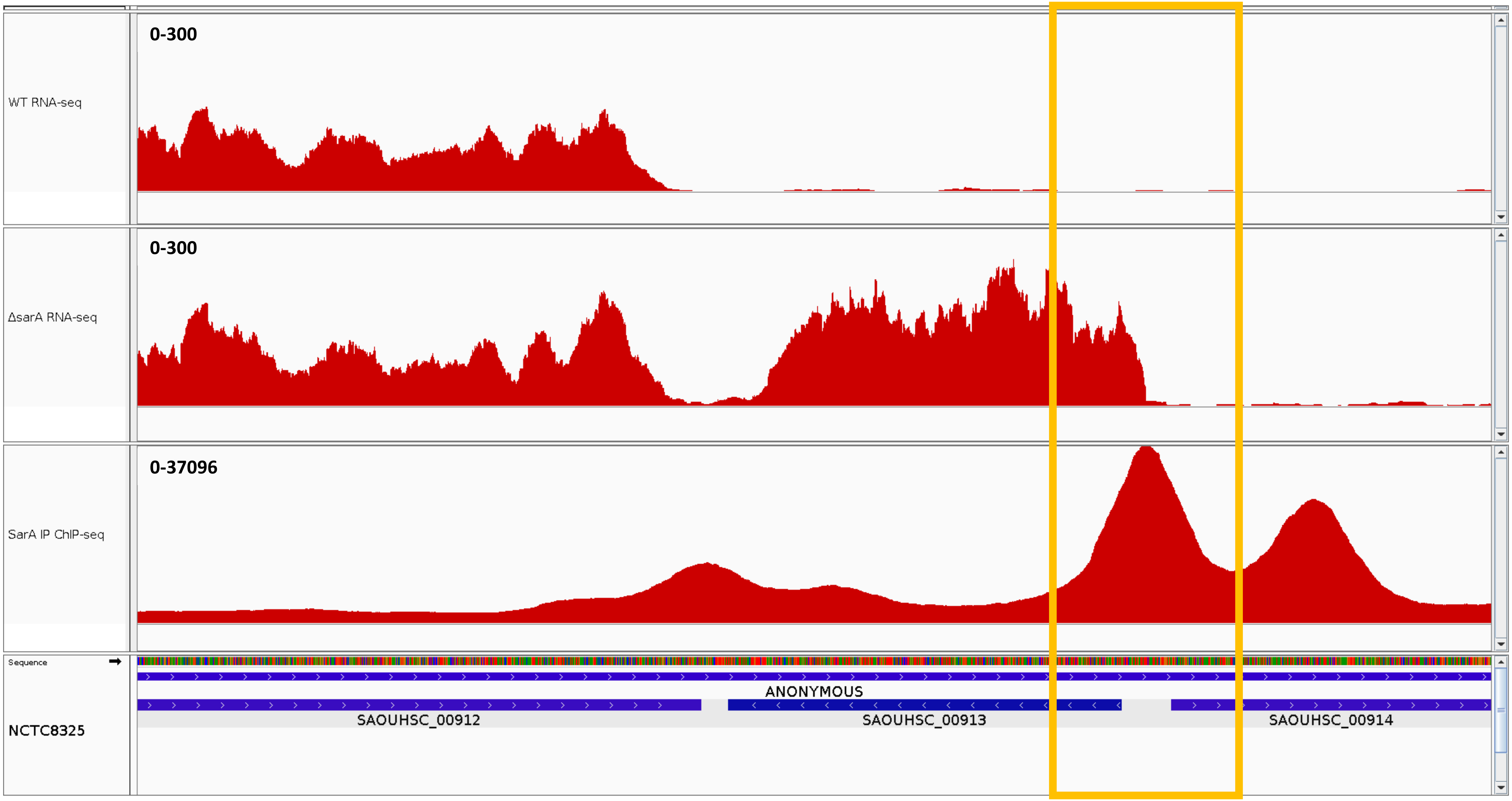

SAOUHSC_00961 (*comK1*)

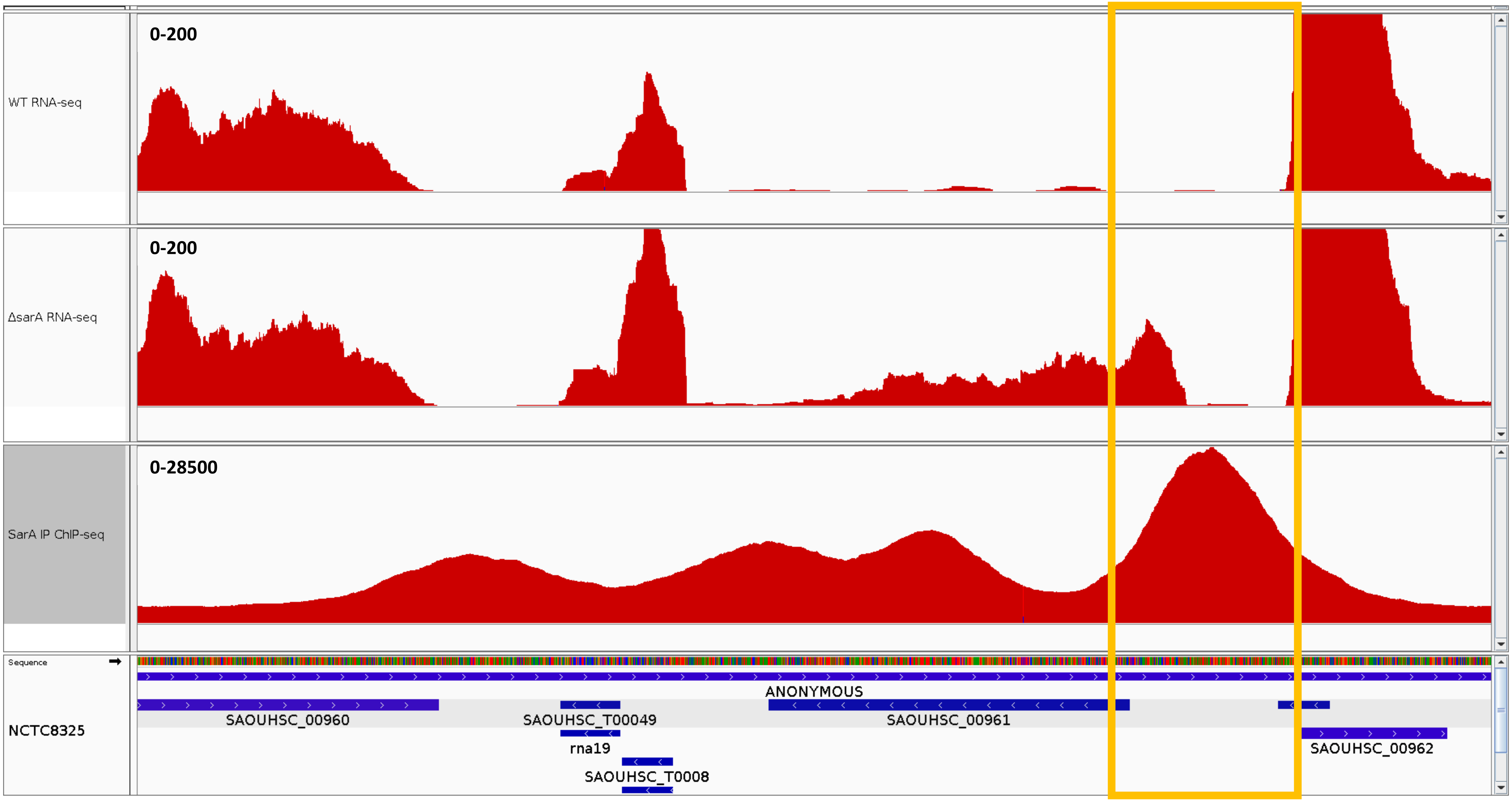

SAOUHSC_00975

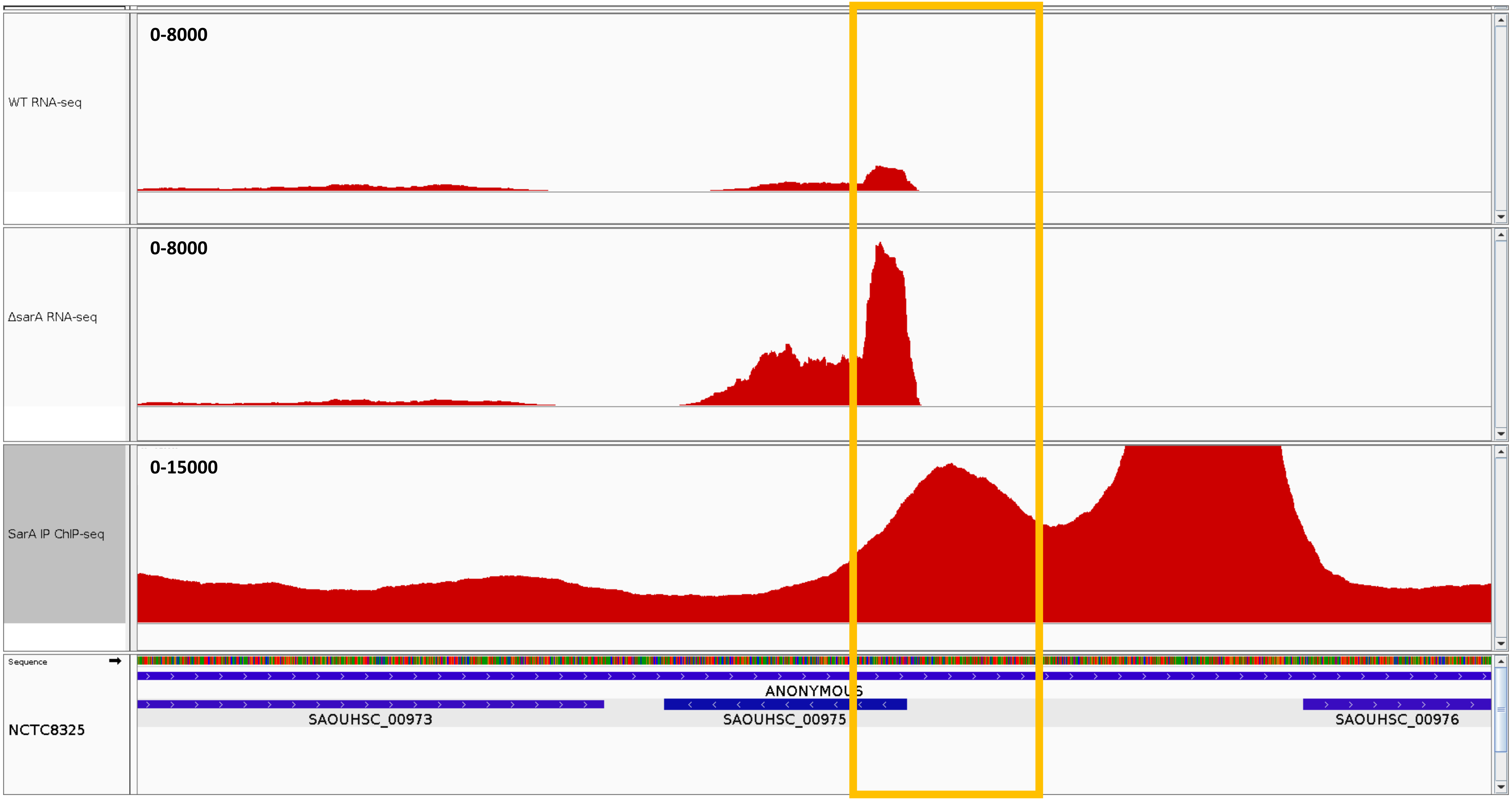

SAOUHSC_01452 (*ald1*)

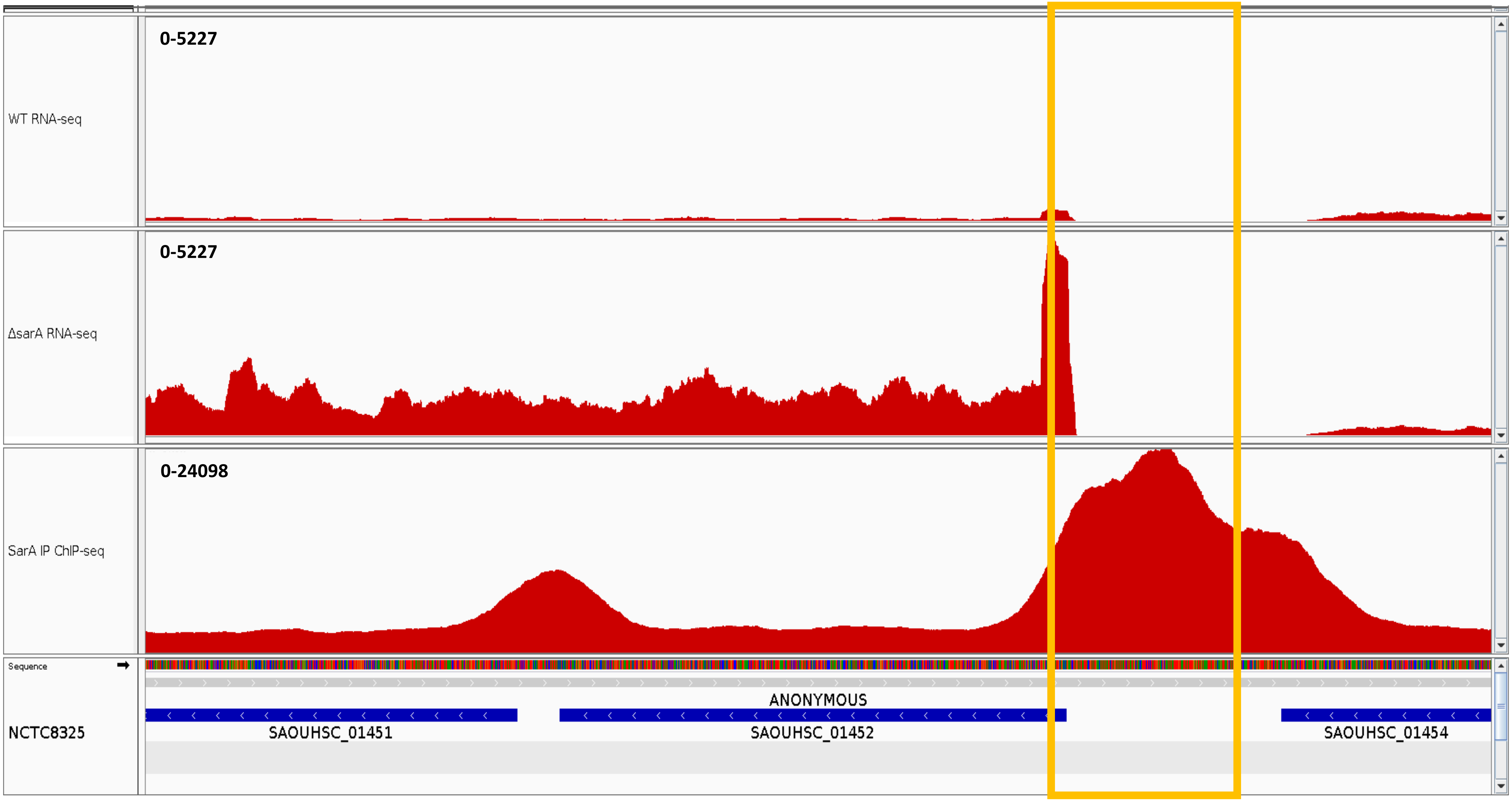

SAOUHSC_02127 (*scpA)*

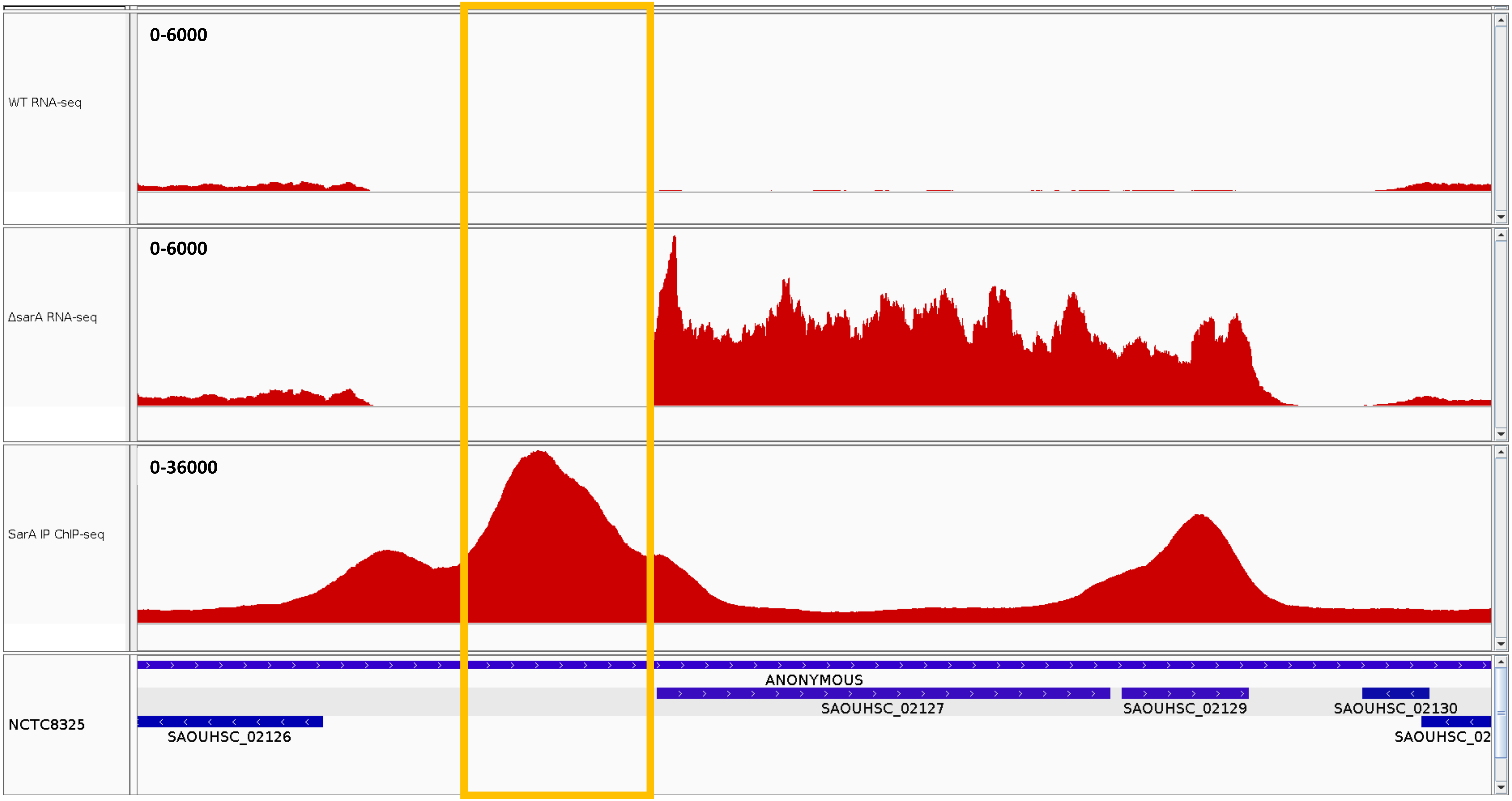

SAOUHSC_02696 (*fmhA*)

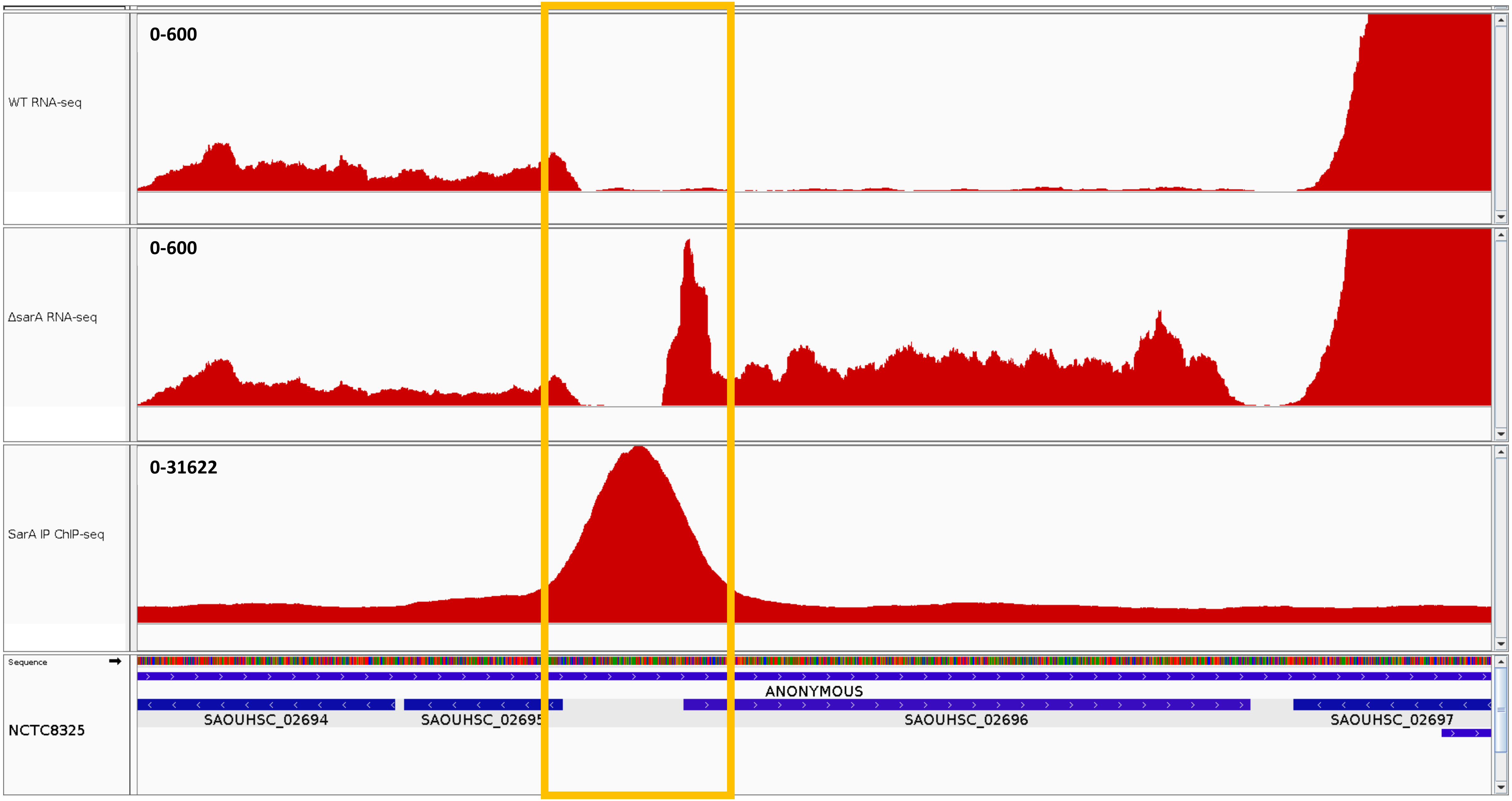

SAOUHSC_02820

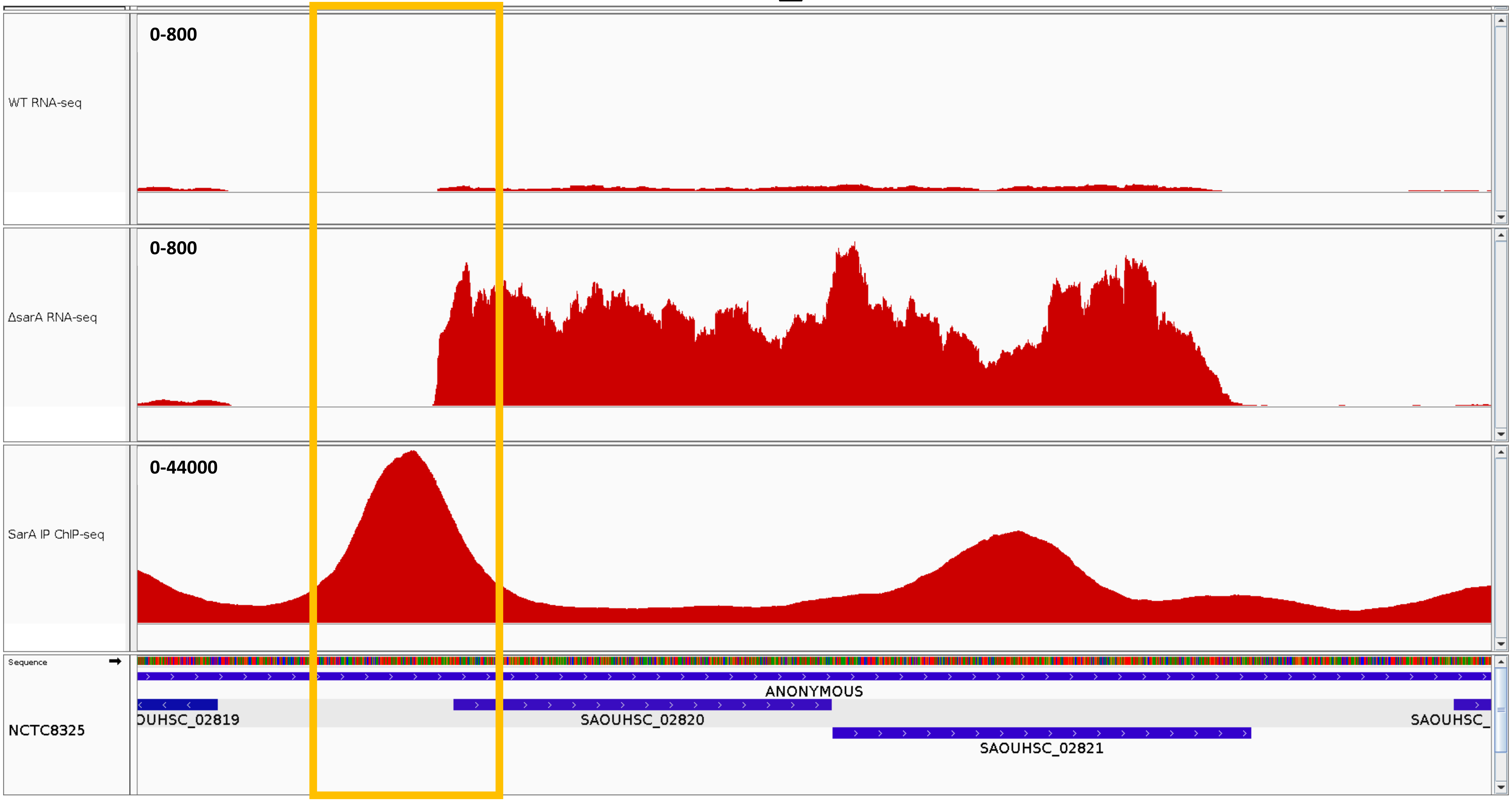

srn_0860 (rsaOB)

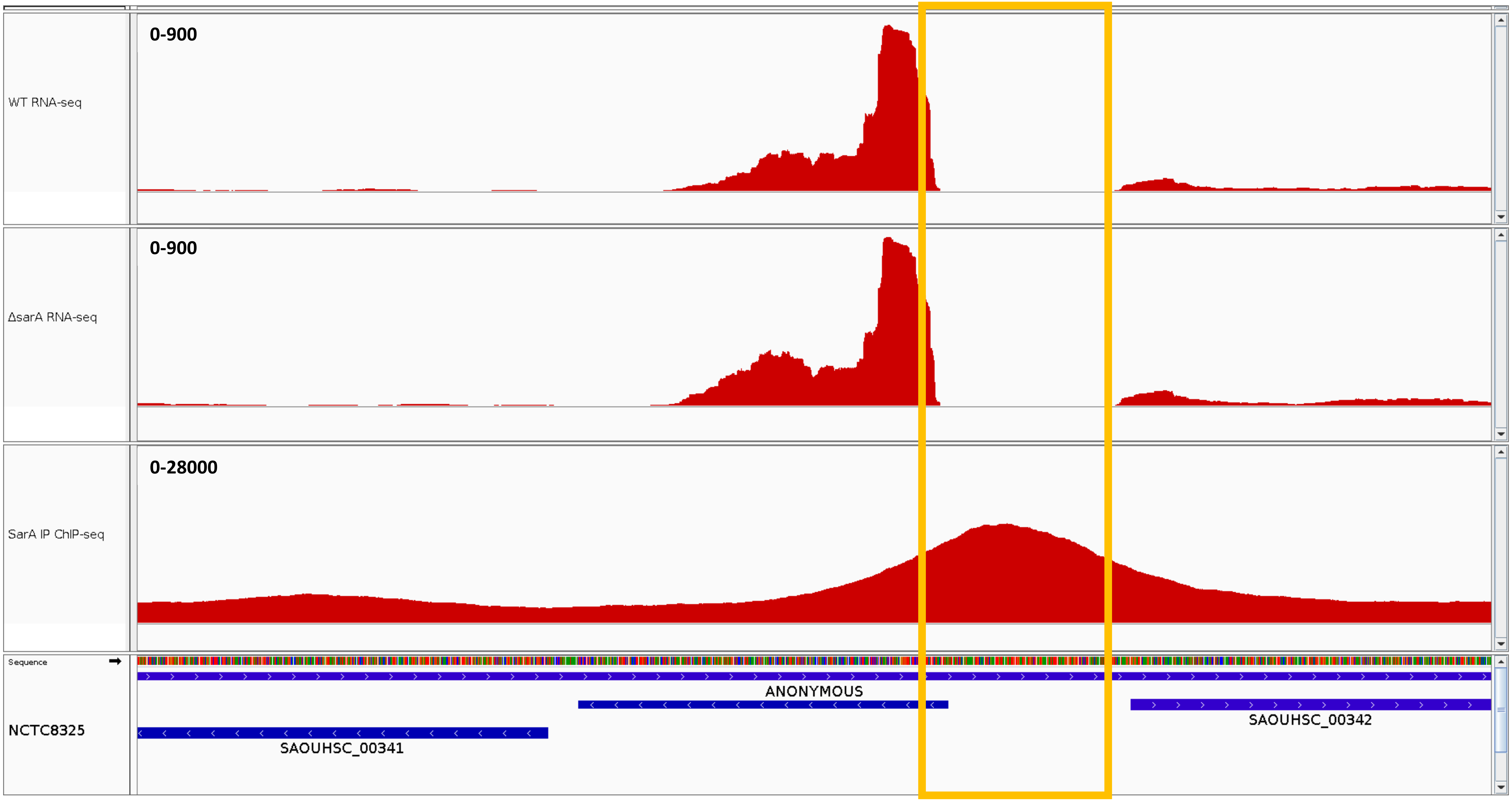

srn_2230 (sprG2)

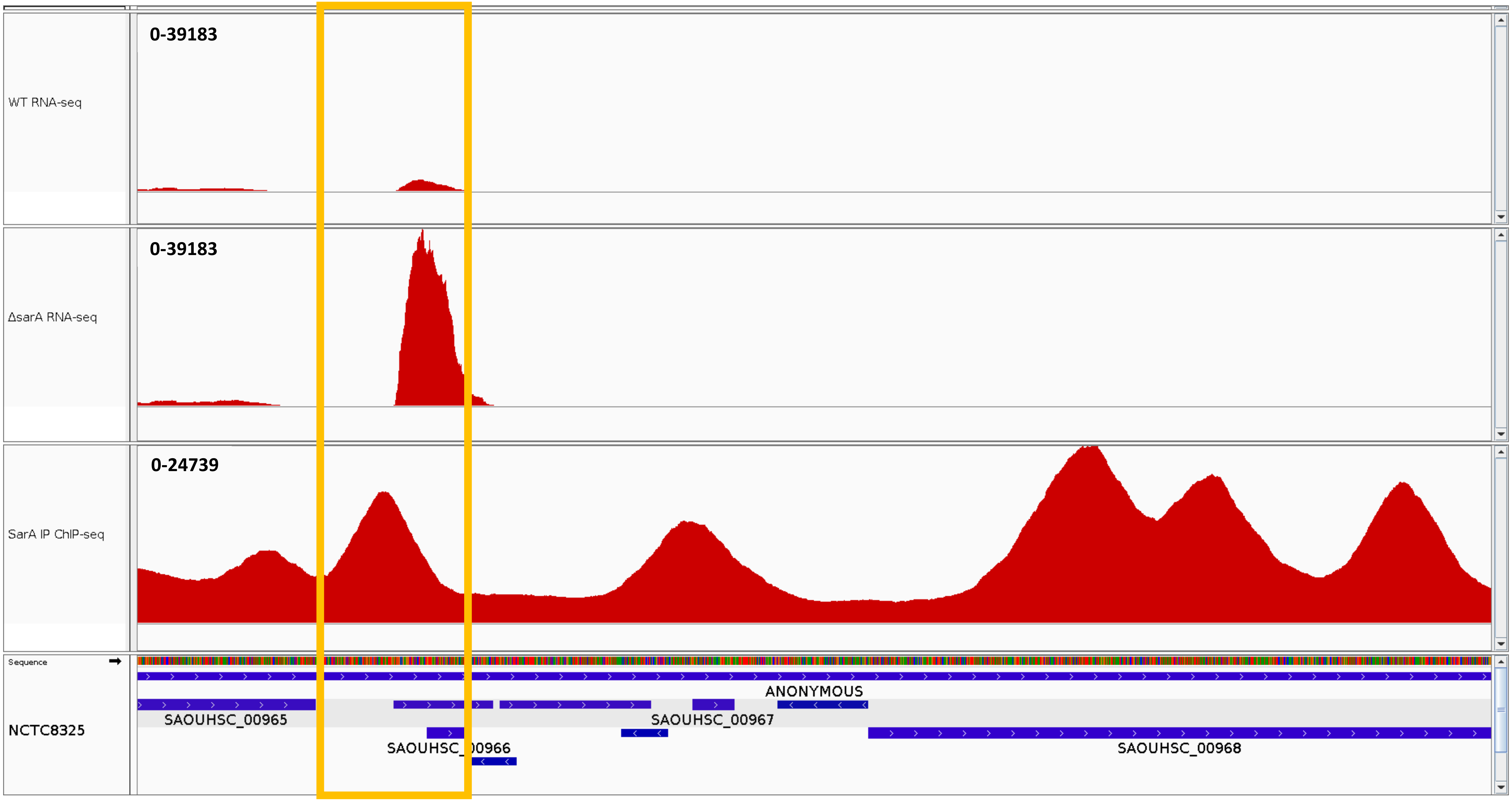

srn_3950 (teg16)

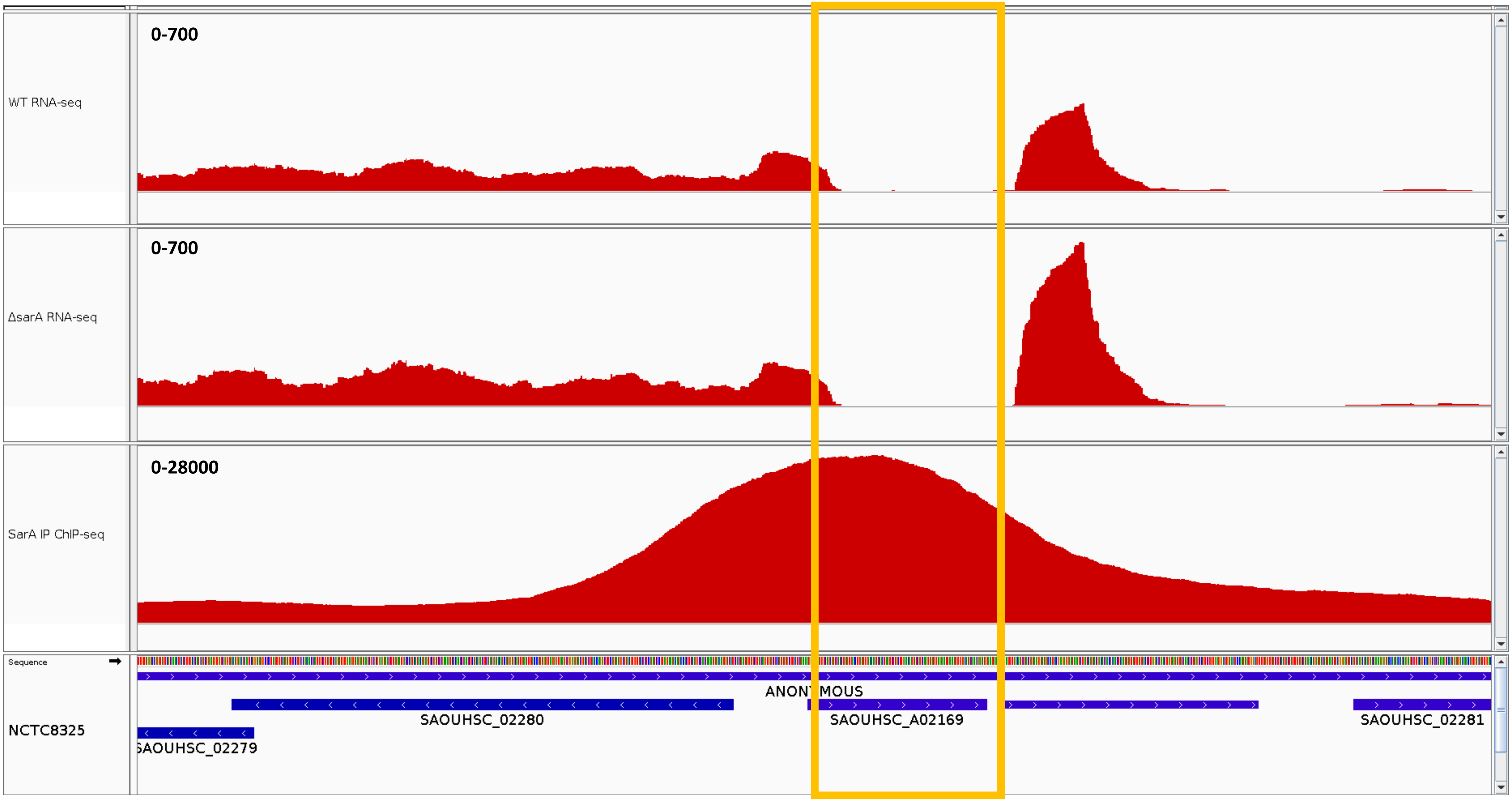

srn_9335 (*tsr29*)

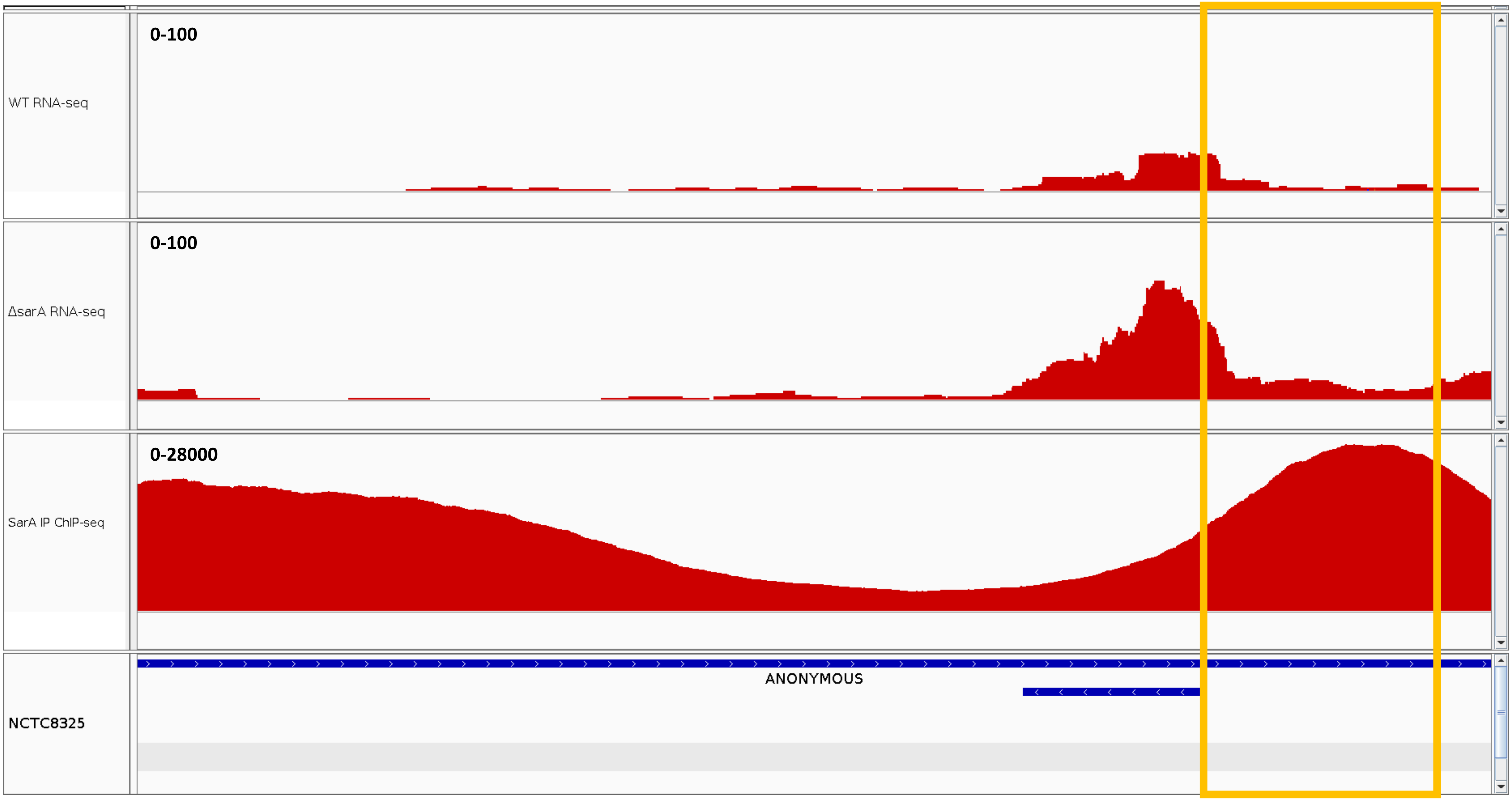

Figure S3: IGV visualizations of potential SarA targets revealed through ChIP-Seq and RNA-Seq.
