# Supplementary material for: Expanding the Staphylococcus aureus SarA Regulon to Small RNAs
Source: mSystems. 2021 Oct 12;6(5):e00713-21. doi: 10.1128/mSystems.00713-21 (PMC8510525; doi:10.1128/mSystems.00713-21)
Supplement: TABLE S6 [file msystems.00713-21-st006.docx]

GLAM2SCAN Version 1056

**Searched against Genebank BacStaphylococcus Version 210 Conditional Formating Conditonal Formating**

/home/meme/meme-5.1.1/bin/glam2scan -O . -n 25 -2 n alignment_1 db/genbank_Staphylococcus_aureus_NCTC_8325_uid237_210.fna < 1.4 < 1.4

> 1.5 > 1.5

If you use this program in your research, please cite: MC Frith, NFW Saunders, B Kobe, TL Bailey, "Discovering sequence motifs with arbitrary insertions and deletions", PLoS Computational Biology, 4(5):e1000071, 2008. [full text]

Gene Info

Gene Info

Gene Info

ChIP

peak

ChIP

peak

ChIP

peak

ChIP

peak

RNA

Seq

RNA

Seq

RNA

Seq p-value

RNA

Seq

RNA

Seq

RNA

Seq

START SITE END STRAND SCORE

Image of

Locus

Known

or novel

Name of Feature Genes Gene Peptide ATG

or sRNA +1

Orientation Center of peak

Relative

to ATG

Score p-value FC 2 h Reg

**adj**

**2h**

FC 4.5 h Reg p-value adj

**4h**

| 942169 | aaaaatattatacc | 942132 | - | 38 | GLAM2Scan_Hit_1 | Used for Consensus | srn_2230 sprG2_Teg19b | 6 bp 5' | srn_2230 | 942176 | **+** | 942122 | 54 | 33.67 | 9.01E-249 | 15.47657 | - | 1.16E-143 | 8.98636 | - | 1.94E-25 |
| --- | --- | --- | --- | --- | --- | --- | --- | --- | --- | --- | --- | --- | --- | --- | --- | --- | --- | --- | --- | --- | --- |
| 639896 | gaaagtaaattaaa | 639932 | + | 35.8 | GLAM2Scan_Hit_2 | Used for Consensus | srn_1640 rsaD | 8 bp 5' | srn_1640 | 639887 | **-** | 639915 | 28 | 21.45 | 2.30E-102 | 1.02201 | - | 9.85E-01 | 10.42897 | - | 3.05E-27 |
| 2556250 | acaaatttatcacta | 2556286 | + | 33.6 | GLAM2Scan_Hit_3 |  | SAOUHSC_02781 | 125 bp 5' | SAOUHSC_02781 | 2556124 | - | 2556303 | 179 | 39.91 | 0 | 0.81218 | + | 7.28E-02 | 1.06737 | - | 8.55E-01 |
|  |  |  |  |  |  | Used for Consensus | srn_4680 Sau19 | 48 bp 5' | srn_4680 | 2556335 | + | 2556303 | 32 | 39.91 | 0 | 1.79285 | - | 2.03E-02 | 4.57573 | - | 3.85E-05 |

2111466 acaaatattttaaat 2111430 - 31.8 GLAM2Scan_Hit_4 SAOUHSC_A02169 Overlap SAOUHSC_A02169 2111333 + 2111393 -60 44.69 0 N/A N/A

| 2.25465 | - | 2.45E-14 | 21.83322 | - | 4.41E-44 |
| --- | --- | --- | --- | --- | --- |
| 2.60383 | - | 1.61E-05 | 3.36531 | - | 1.44E-04 |
| 7.9365 | - | 1.62E-21 | 12.04632 | - | 1.10E-34 |
| 1.4047 | - | 2.11E-03 | 10.50866 | - | 7.34E-29 |
| 1.07836 | - | 8.09E-01 | 1.70307 | - | 1.97E-02 |

| 0.88975 | + | 7.72E-01 | 1.20761 | - | 6.66E-01 |
| --- | --- | --- | --- | --- | --- |
| 1.72251 | - | 1.31E-06 | 3.21929 | - | 1.30E-08 |
| 0.98012 | + | 9.59E-01 | 1.16186 | - | 6.06E-01 |
|  |  | |  |  | |
| 0.92778 | + | 9.75E-01 | 0.91713 | + | 9.93E-01 |
| 2.90054 | - | 1.84E-02 | 1.98166 | - | 2.05E-01 |
| 13.90664 | - | 1.20E-50 | 24.83422 | - | 1.66E-52 |
| 2.44115 | - | 4.40E-19 | 2.09854 | - | 6.74E-04 |
| 1.27089 | - | 1.75E-01 | 2.71588 | - | 5.58E-05 |
| No No | | |  |  | |
| 0.7078 | + | 5.05E-04 | 0.92413 | + | 6.68E-01 |
| 1.10653 | - | 4.45E-01 | 1.6279 | - | 3.30E-02 |
| 1.31656 | - | 1.42E-02 | 3.15192 | - | 1.01E-07 |
| 6.19355 | - | 4.86E-57 | 3.96395 | - | 5.84E-12 |
| 0.79923 | + | 1.00E+00 | 2.83861 | - | 5.46E-01 |
| 0.32747 | + | 6.06E-01 | 1.34821 | - | 9.99E-01 |
| 6.19355 | - | 4.86E-57 | 3.96395 | - | 5.84E-12 |
| 0.9089 | + | 6.95E-01 | 0.70867 | + | 2.30E-01 |
| 0.72832 | + | 4.41E-03 | 0.52434 | + | 8.64E-03 |
| 1.46399 | - | 3.34E-01 | 9.42549 | - | 1.32E-22 |
| 3.40503 | - | 1.12E-27 | 6.36069 | - | 3.63E-21 |
| 26.73416 | - | 5.60E-186 | 30.13278 | - | 7.57E-59 |
| 130.10088 | - | 4.46E-277 | 269.37109 | - | 4.52E-115 |
| 2.07206 | - | 2.11E-03 | 5.45302 | - | 5.57E-15 |
| 0.7941 | + | 5.15E-01 | 3.54694 | - | 1.79E-07 |
| 1.10623 | - | 8.48E-01 | 2.59527 | - | 4.65E-05 |
| 0.4592 | + | 4.05E-12 | 37.75961 | - | 1.38E-61 |
| 0.8858 | + | 4.67E-01 | 0.96428 | + | 9.67E-01 |
| 4.09833 | - | 7.20E-44 | 3.67075 | - | 3.29E-09 |
| 0.96048 | + | 8.52E-01 | 0.88959 | + | 7.01E-01 |
| 1.64515 | - | 3.69E-06 | 0.65349 | + | 7.92E-02 |
| 1.61897 | - | 4.82E-06 | 1.76011 | - | 7.28E-03 |
| 0.76118 | + | 1.73E-02 | 0.71527 | + | 2.13E-01 |
|  |  |  |  |  |  |
| 130.10088 | - | 4.46E-277 | 269.37109 | - | 4.52E-115 |
| 3.7387 | - | 3.17E-37 | 1.17192 | - | 5.82E-01 |
| 1.62296 | - | 7.62E-02 | 2.22359 | - | 1.07E-02 |
| 0.79722 | + | 5.79E-02 | 0.51252 | + | 8.70E-03 |
| 0.76106 | + | 3.02E-02 | 1.24184 | - | 4.29E-01 |
| 2.3299 | - | 5.23E-18 | 0.52375 | + | 7.54E-03 |
| 0.92465 | + | 7.21E-01 | 0.44762 | + | 4.95E-04 |
| 0.98055 | + | 9.62E-01 | 0.97202 | + | 9.81E-01 |
| 0.95479 | + | 9.39E-01 | 0.89998 | + | 8.25E-01 |
| 1.10446 | - | 7.24E-01 | 0.96502 | + | 1.00E+00 |
| 1.28162 | - | 2.61E-02 | 1.94643 | - | 2.39E-03 |
| 0.87532 | + | 3.10E-01 | 0.85562 | + | 6.11E-01 |
| 0.87819 | + | 4.67E-01 | 0.79229 | + | 4.11E-01 |
| 0.92257 | + | 5.91E-01 | 0.89695 | + | 7.11E-01 |
| 6.821 | - | 1.52E-73 | 5.39123 | - | 8.24E-17 |
| 7.09485 | - | 4.72E-64 | 8.90355 | - | 6.84E-28 |
| 0.88443 | + | 3.92E-01 | 1.07467 | - | 8.43E-01 |
| 1.76035 | - | 1.88E-08 | 4.67781 | - | 1.50E-13 |
| 0.89417 | + | 4.59E-01 | 0.56821 | + | 1.87E-02 |
| 0.92257 | + | 5.91E-01 | 0.89695 | + | 7.11E-01 |
|  |  |  |  |  |  |
| 3.32792 | - | 1.79E-03 | 21.50901 | - | 1.35E-21 |
| 3.25168 | - | 1.09E-32 | 1.43121 | - | 3.00E-01 |
| 0.70001 | + | 1.11E-03 | 0.52117 | + | 1.05E-02 |
| 4.73974 | - | 4.74E-53 | 26.62055 | - | 1.94E-57 |
| 2.70575 | - | 6.72E-07 | 1.20429 | - | 7.11E-01 |
| 0.7801 | + | 3.60E-02 | 0.68019 | + | 1.92E-01 |
| 3.11672 | - | 8.20E-29 | 1.84375 | - | 5.48E-03 |
| 1.10193 | - | 6.11E-01 | 0.55357 | + | 1.99E-02 |
| 2.45627 | - | 5.07E-19 | 2.32279 | - | 6.09E-05 |
| 17.29416 | - | 8.53E-136 | 28.67278 | - | 1.53E-54 |
| 0.15046 | + | 3.06E-01 | None | | |

| 1.01972 | - | 1.00E+00 | 0.53748 | + | 9.21E-03 |
| --- | --- | --- | --- | --- | --- |
| 1.24481 | - | 6.59E-02 | 1.09092 | - | 8.13E-01 |
| 1.32053 | - | 1.94E-02 | 3.03244 | - | 2.75E-07 |
| 1.11872 | - | 4.25E-01 | 3.12445 | - | 1.23E-07 |
| 5.60247 | - | 3.44E-39 | 1.1356 | - | 7.08E-01 |
| 4.4948 | - | 3.33E-06 | 1.45916 | - | 1.32E-01 |
| 1.39979 | - | 5.91E-01 | 1.60988 | - | 3.01E-01 |
| 1.46399 | - | 3.34E-01 | 9.42549 | - | 1.32E-22 |
| 2.53772 | - | 1.63E-15 | 4.13676 | - | 1.33E-12 |
| 0.78372 | + | 2.71E-01 | 9.24658 | - | 2.66E-26 |
| 6.3261 | - | 4.48E-28 | 5.3767 | - | 6.32E-11 |
| 0.81029 | + | 8.47E-02 | 0.35705 | + | 4.01E-05 |
| 1.15299 | - | 3.17E-01 | 1.81599 | - | 7.64E-03 |

| 174152 acaaatattaaaag 174190 + 31.5 GLAM2Scan_Hit_5  2714676 aaaaatatatagat 2714641 - 31 GLAM2Scan_Hit_6 | | | | | | Used for Consensus srn_3950 teg16 Used for Consensus srn_0455 tsr9 Used for Consensus srn_4980 teg32 | 32 bp from 5' srn_3950 2111499 + 2111393 106 44.69 0  25 bp 5' srn_0455 174126 - 174208 82 20.11 2.78E-90  15 bp 5' srn_4980 2714625 - 2714742 117 46.37 0 | | | | | | | |  |  |  |  |  | |
| --- | --- | --- | --- | --- | --- | --- | --- | --- | --- | --- | --- | --- | --- | --- | --- | --- | --- | --- | --- | --- |
| 356702 | aaagatttgtcaac | 356741 | + | 30.4 | GLAM2Scan_Hit_7 | Used for Consensus srn_0860 RsaOB | 26 bp 5' | srn_0860 | 356675 | - | 356763 | 88 | 24.76 | 1.09E-135 |  |  |  |  |  |  |
|  |  |  |  |  |  |  |  | SAOUHSC_00342 | 356884 | + | 356763 | 121 | 24.76 | 1.09E-135 |  |  |  |  |  |  |
| 2614055 | aaaaataattaatg | 2614016 | - | 29.8 | GLAM2Scan_Hit_8 | SAOUHSC_02838 | On 5' | SAOUHSC_02838 | None |  |  |  | NA | NA | N/A |  |  | N/A |  |  |
| 909388 | acaaatatttaaata | 909349 | - | 29.4 | GLAM2Scan_Hit_9 | SAOUHSC_00936 | Overlap | SAOUHSC_00936 | 908438 | + | 909377 | -939 | NA | NA |  |  |  |  |  |  |
| 1795114 | gaaaatatatcaat | 1795150 | + | 29.3 | GLAM2Scan_Hit_10 | Used for Consensus srn_3500 sRNA277 | 20 bp 5' | srn_3500 | 1795093 | - | 1795100 | 7 | 52.29 | 0 |  |  |  |  |  |  |
| 746762 | aaaaataagattaa | 746799 | + | 29.3 | GLAM2Scan_Hit_11 | SAOUHSC_00764 | On 3' | SAOUHSC_00764 | 746397 | + | 746769 | -372 | NA | NA |  |  |  |  |  |  |
|  |  |  |  |  |  |  |  |  |  |  |  |  |  |  |  |  |  |  |  |  |
| 2502462 | ataattaaattcattt | 2502497 | + | 29 | GLAM2Scan_Hit_12 | Used for Consensus SAOUHSC_02720 / | On 3' | SAOUHSC_02720 | N/A |  |  |  | NA | NA |  |  |  |  |  |  |
|  |  |  |  |  |  | srn_4540 sprAs2 | 34 bp 3' | srn_4540 | 2502532 | + | 2502457 | 75 | NA | NA |  |  |  |  |  |  |
| 1863936 | aaaaataaaataat | 1863899 | - | 29 | GLAM2Scan_Hit_13 | Used for Consensus srn_9340 sRNA287 | 1 bp 5' | srn_9340 | 1863897 | - | 1863948 | 51 | 54.78 | 0 |  |  |  |  |  |  |
| 660508 | aaaaatatgaacc | 660473 | - | 29 | GLAM2Scan_Hit_14 | SAOUHSC_00671 (secretory antigen SsaA-like protein) / | 143 bp 5' / | SAOUHSC_00671 | 660329 | - | 660473 | 144 | 44.56 | 0 |  |  |  |  |  |  |
|  |  |  |  |  |  | Used for Consensus srn_1680 sRNA142 | 63 bp 5' | srn_1680 | 660569 | + | 660473 | 96 | 44.56 | 0 |  |  |  |  |  |  |
| 661458 | aaaaatatattacct | 661495 | + | 28.3 | GLAM2Scan_Hit_15 | None Close |  |  |  |  |  | -125pb 5' | 12.64 | 6.04E-37 |  |  |  |  |  |  |
| 2721352 | aaaaatatgtcaaa | 2721315 | - | 28.2 | GLAM2Scan_Hit_16 | New sRNA srn_5010 Teg33 | 9 bp 5' | srn_5010 | 2721313 | - | 2721325 | 12 | 12.64 | 6.04E-37 |  |  |  |  |  |  |
| 2744123 | aaaaataaattaat | 2744088 | - | 28.2 | GLAM2Scan_Hit_17 | Same location as 168 SAOUHSC_02978 (phage infection protein) | 29 bp from 5' / | SAOUHSC_02978 | 2744058 | - | 2744123 | 65 | 33.43 | 2.63E-245 |  |  |  |  |  |  |
|  |  |  |  |  |  | / SAOUHSC_02979 (N-acetylmuramoyl-L-alanine amidase) | 145 bp 5' | SAOUHSC_02979 | 2744269 | + | 2744123 | 146 | 33.43 | 2.63E-245 |  |  |  |  |  |  |
| 1831080 | aaaaatattataata | 1831044 | - | 28.2 | GLAM2Scan_Hit_18 | Same location as 20 SAOUHSC_01923 | 55 bp from 5' | SAOUHSC_01923 | **1830988** | **-** | 1831664 | 676 | NA | NA |  |  |  |  |  |  |
| 1488099 | aaaaataaattattt | 1488136 | + | 27.5 | GLAM2Scan_Hit_19 | SAOUHSC_01540 (bacteriophage L54a HNH endonuclease fa | On 3' | SAOUHSC_01540 | N/A |  |  |  | NA | NA |  |  |  |  |  |  |
|  |  |  |  |  |  | SAOUHSC_01539 (terminase small subunit | / 100 bp 5' | SAOUHSC_01539 | 1487998 | - | 1488110 | 112 | 12.59 | 1.20E-36 |  |  |  |  |  |  |
| 1831039 | caaaataaaatac | 1831075 | + | 27.4 | GLAM2Scan_Hit_20 | Same location as 18 SAOUHSC_01923 | 50 bp 5' | SAOUHSC_01923 | 1830988 | **-** | 1831664 | 676 | NA | NA |  |  |  |  |  |  |
| 1459693 | aaaaatatgataaa | 1459658 | - | 27.4 | GLAM2Scan_Hit_21 | SAOUHSC_01508 | Overlap | SAOUHSC_01508 | 1460141 | - | 1459652 | -489 | 23.26 | 5.31E-120 |  |  |  |  |  |  |
| 1533308 | acaaatataactat | 1533344 | + | 27.4 | GLAM2Scan_Hit_22 | SAOUHSC_01609 | Overlap | SAOUHSC_01609 | None? |  |  |  | NA | NA |  |  |  |  |  |  |
| 1848285 | gaaaatataataat | 1848249 | - | 27.4 | GLAM2Scan_Hit_23 | Same location as 65 SAOUHSC_01944 | 39 bp from 5 | SAOUHSC_01944 | 1848325 | + | 1848234 | 91 | 41.93 | 0 |  |  |  |  |  |  |
| 1171183 | aaaaataaatcaa | 1171223 | + | 27.2 | GLAM2Scan_Hit_24 | SAOUHSC_01220 | Overlap | SAOUHSC_01220 | 1170355 | + | 1171445 | -1090 | 41.84 | 0 |  |  |  |  |  |  |
| 2036267 | gaaaaaatattaaa | 2036303 | + | 27.1 | GLAM2Scan_Hit_25 | SAOUHSC_02171 staphylokinase | 100 bp 5' | SAOUHSC_02171 | 2036166 | - | 2036270 | 104 | 23.86 | 3.73E-126 |  |  |  |  |  |  |
| 799922 | atagttattttattata | 799959 | + | 27.1 | GLAM2Scan_Hit_26 | Same location as 35, SAOUHSC_00818 thermonuclease | 74 bp | SAOUHSC_00818 | 800034 | + | 799858 | 176 | 42.72 | 0 |  |  |  |  |  |  |
| 1858881 | acaaattaataaca | 1858842 | - | 27 | GLAM2Scan_Hit_27 | Used for Consensus srn_9335 tsr29 | 59 bp 5' | srn_9335 | 1858782 | - | 1858895 | 113 | 40.47 | 0 |  |  |  |  |  |  |
| 899869 | aaaattattacaag | 899831 | - | 27 | GLAM2Scan_Hit_28 | SAOUHSC_00928 (oligopeptide ABC transporter substrate-bin | 53 bp 5' | SAOUHSC_00928 | 899923 | + | 899854 | 69 | 38.22 | 5.47622361850438e-320 |  |  |  |  |  |  |
| 433225 | aaaaatagaacatt | 433190 | - | 26.8 | GLAM2Scan_Hit_29 | SAOUHSC_00434 (LysR family transcriptional regulator) / | 38 bp 5' / 106 bp 5' | SAOUHSC_00434 | 433151 | - | 433254 | 103 | 27.85 | 5.38E-171 |  |  |  |  |  |  |
|  |  |  |  |  |  | SAOUHSC_00435 (glutamate synthase large subunit) |  | SAOUHSC_00435 | 433332 | + | 433254 | 78 | 27.85 | 5.38E-171 |  |  |  |  |  |  |
| 1257462 | gaaaacaaaataa | 1257426 | - | 26.7 | GLAM2Scan_Hit_30 | SAOUHSC_01313 (histidine kinase) | Overlap | SAOUHSC_01313 | 1257226 | + | 1257353 | -127 | 44.64 | 0 |  |  |  |  |  |  |
| 664367 | aacaatataaagc | 664403 | + | 26.7 | GLAM2Scan_Hit_31 | SAOUHSC_00674_sarX | Overlap | SAOUHSC_00674 | 664261 | + | 664176 | 85 | 35.87 | 4.89E-282 |  |  |  |  |  |  |
| 76259 | aaattttaataaaaa | 76223 | - | 26.6 | GLAM2Scan_Hit_32 | sarS (Same location a SAOUHSC_00070_sarS | 70 bp 5' | SAOUHSC_00070 | 76152 | - | 76211 | - | 47.42 | 0 |  |  |  |  |  |  |
| 1219781 | aaaaataattcaaa | 1219819 | + | 26.6 | GLAM2Scan_Hit_33 | SAOUHSC_01264 / | 89 bp 5' / | SAOUHSC_01264 | 1219691 | - | 1219757 | 66 | 27.20 | 3.29E-163 |  |  |  |  |  |  |
|  |  |  |  |  |  | SAOUHSC_01265 | 45 bp 5' | SAOUHSC_01265 | 1219865 | + | 1219757 | 108 | 27.20 | 3.29E-163 |  |  |  |  |  |  |
| 2633678 | aaaaataacacac | 2633714 | + | 26.6 | GLAM2Scan_Hit_34 | SAOUHSC_02861 (methylated-DNA--protein-cysteinemethyltra | 252 bp 5' / | SAOUHSC_02861 | 2633425 | - | 2633711 | 286 | 25.81 | 3.03E-147 |  |  |  |  |  |  |
|  |  |  |  |  |  | SAOUHSC_02862 (ATP-dependent Clp protease ATP-binding | 119 bp 5' | SAOUHSC_02862 | 2633834 | + | 2633711 | 123 | 25.81 | 3.03E-147 |  |  |  |  |  |  |
| 799975 | atacatttttacaatt | 799938 | - | 26.5 | GLAM2Scan_Hit_35 | Same location as 26, SAOUHSC_00818 thermonuclease | 74 bp | SAOUHSC_00818 | 800034 | + | 799858 | 176 | 42.72 | 0 |  |  |  |  |  |  |
| 791130 | agaaatatattactt | 791166 | + | 26.3 | GLAM2Scan_Hit_36 | SAOUHSC_00808 | 195 bp 5' | SAOUHSC_00808 | 790934 | - | 791684 | 750 | NA | NA |  |  |  |  |  |  |
| 1079806 | aaaaattatataata | 1079770 | - | 26.2 | GLAM2Scan_Hit_37 | SAOUHSC_01125 (superantigen-like protein) /01124 dans ope | Overlap | SAOUHSC_01125 | 1086365 | - | 1079794 | -6571 | 28.87 | 1.31E-183 |  |  |  |  |  |  |
| 2344699 | gcaaatatgttaaa | 2344735 | + | 26.1 | GLAM2Scan_Hit_38 | SAOUHSC_02549 (molybdenum ABC transporter substrate-bi | 221 bp 5' / | SAOUHSC_02549 | 2344476 | - | 2344610 | 134 | 36.82 | 3.71E-297 |  |  |  |  |  |  |
|  |  |  |  |  |  | SAOUHSC_02550 (formate dehydrogenase accessory protein | 31 bp 5' | SAOUHSC_02550 | 2344767 | + | 2344610 | 157 | 36.82 | 3.71E-297 |  |  |  |  |  |  |
| 2093735 | aagaataaaaaac | 2093697 | - | 25.8 | GLAM2Scan_Hit_39 | Used for Consensus srn_3910 RNAIII | 23 bp 5' | srn_3910 | 2093673 | - | 2093784 | 111 | NA | NA |  |  |  |  |  |  |
| 1802849 | aaaattaaagactt | 1802884 | + | 25.8 | GLAM2Scan_Hit_40 | SAOUHSC_01890 | Overlap | SAOUHSC_01890 | 1803945 | - | 1802880 | -1065 | 3.59 | 0.000166 |  |  |  |  |  |  |
| 2557995 | ataagtatataatcc | 2557959 | - | 25.8 | GLAM2Scan_Hit_41 | SAOUHSC_02784 | 19 bp 3' | SAOUHSC_02784 | N/A |  |  |  | NA | NA |  |  |  |  |  |  |
| 1329349 | acaaatttagtaaa | 1329313 | - | 25.7 | GLAM2Scan_Hit_42 | SAOUHSC_01385 (phosphate transporter ATP-binding protein | 30 bp 5' / | SAOUHSC_01385 | None |  |  |  | NA | NA |  |  |  |  |  |  |
|  |  |  |  |  |  | SAOUHSC_01386 (phosphate ABC transporter permease) | On 3' | SAOUHSC_01386 | N/A |  |  |  | NA | NA |  |  |  |  |  |  |
| 838474 | aataataaaataat | 838509 | + | 25.7 | GLAM2Scan_Hit_43 | SAOUHSC_00872 | 104 bp 3' | SAOUHSC_00872 | N/A |  |  |  | NA | NA |  |  |  |  |  |  |
|  |  |  |  |  |  | / SAOUHSC_00873 | / 102 bp 3' | SAOUHSC_00873 | N/A |  |  |  | NA | NA |  |  |  |  |  |  |
| 2041567 | aagaatataacata | 2041532 | - | 25.6 | GLAM2Scan_Hit_44 | SAOUHSC_02180 (phage minor structural protein) | Overlap | SAOUHSC_02180 | None |  |  |  | NA | NA |  |  |  |  |  |  |
| 547532 | ataaataatttaaag | 547571 | + | 25.6 | GLAM2Scan_Hit_45 | SAOUHSC_00543 / | 211 bp 3' / | SAOUHSC_00543 | N/A |  |  |  | NA | NA |  |  |  |  |  |  |
|  |  |  |  |  |  | SAOUHSC_00544 (fibrinogen-binding protein SdrC) | 220pb 5' | SAOUHSC_00544 | 547751 | + | 547677 | 74 | 49.28 | 0 |  |  |  |  |  |  |
| 1861599 | ataaatattttattttt | 1861635 | + | 25.4 | GLAM2Scan_Hit_46 | Used for Consensus ( srn_3610 sprC | 0 bp 5' | srn_3610 | 1861598 | - | 1861615 | 17 | 49.88 | 0 |  |  |  |  |  |  |
| 646288 | aaaaatatgatagt | 646324 | + | 25.4 | GLAM2Scan_Hit_47 | SAOUHSC_00659 | 25 bp 5' | SAOUHSC_00659 | 346350 | + | 346300 | 50 | NA | NA |  |  |  |  |  |  |
| 2453406 | ataaatattttaatttt | 2453371 | - | 25.4 | GLAM2Scan_Hit_48 | SAOUHSC_02667 | 44 bp 5' | SAOUHSC_02667 | 2453326 | - | 2453398 | 72 | 19.20 | 1.93E-82 |  |  |  |  |  |  |
| 1164201 | ataaatataataaa | 1164237 | + | 25.4 | GLAM2Scan_Hit_49 | SAOUHSC_01213 | Overlap | SAOUHSC_01213 | 1164741 | - | 1163222 | -1519 | 15.40 | 8.68E-54 |  |  |  |  |  |  |
| 547314 | aaaataatattaaa | 547350 | + | 25.3 | GLAM2Scan_Hit_50 | Same location as 88, SAOUHSC_00543 / | On 3' | SAOUHSC_00543 | N/A |  |  |  | NA | NA |  |  |  |  |  |  |
|  |  |  |  |  |  | SAOUHSC_00544 (fibrinogen-binding protein SdrC) |  |  |  |  |  |  |  |  |  |  |  |  |  |  |
| 942915 | gcaaataaattaac | 942959 | + | 25.2 | GLAM2Scan_Hit_51 | SAOUHSC_00967 | Overlap | SAOUHSC_00967 | 942903 | + | 942886 | 17 | 31.40 | 1.06E-216 |  |  |  |  |  |  |
| 864602 | ataaatatgtaaaa | 864566 | - | 25.1 | GLAM2Scan_Hit_52 | Same location as 188 SAOUHSC_00899 (argininosuccinate synthase) / SAOUHSC_0 | 56 bp 5' | SAOUHSC_00899 | 864509 | - | 864609 | 100 | 13.57 | 3.10E-42 |  |  |  |  |  |  |
|  |  |  |  |  |  |  |  | SAOUHSC_00900 | 864860 | + | 864609 | 251 | 13.57 | 3.10E-42 |  |  |  |  |  |  |
| 949200 | aaagattttttataag | 949240 | + | 25.1 | GLAM2Scan_Hit_53 | SAOUHSC_00975 | 67 bp from 5' | SAOUHSC_00975 | 949132 | - | None |  | NA | NA |  |  |  |  |  |  |
| 2081590 | caaaataaataaat | 2081626 | + | 25.1 | GLAM2Scan_Hit_54 | SAOUHSC_02246 | Overlap | SAOUHSC_02246 | 2081634 | - | 2081710 | 76 | 43.18 | 0.00E+00 |  |  |  |  |  |  |
| 2.66E+06 | aaaaatattgtgata | 2659427 | - | 25.1 | GLAM2Scan_Hit_55 | Same location as 171 SAOUHSC_02885 | 18 bp 5' | SAOUHSC_02885 | 2659408 | - | 2659660 | 252 | 39.94 | 0.00E+00 |  |  |  |  |  |  |
| 6.00E+05 | gaaattaaattaatc | 599930 | + | 25 | GLAM2Scan_Hit_56 | New sRNA srn_1530 sRNA133 | 66 bp 5' | srn_1530 | 599997 | + | 599871 | 126 | 50.97 | 0.00E+00 |  |  |  |  |  |  |
| 8.82E+05 | aaaaatatttacaa | 882531 | + | 25 | GLAM2Scan_Hit_57 | SAOUHSC_00912 (ATP-dependent Clp protease ATP-binding | 104 bp 5' | SAOUHSC_00912 | 882636 | + | 882505 | 131 | 5.12 | 1.55E-07 |  |  |  |  |  |  |
| 963411 | aaaattataaaaaa | 963447 | + | 25 | GLAM2Scan_Hit_58 | Same location as 114 SAOUHSC_00991 / SAOUHSC_00992 (MarR family transcripti | 96 bp 5' / 19 bp 5' | SAOUHSC_00991 | 963314 | - | 963390 | 76 | 38.30 | 2.88E-321 |  |  |  |  |  |  |
|  |  |  |  |  |  |  |  | SAOUHSC_00992 | 963467 | + | 963390 | 77 | 38.30 | 2.88E-321 |  |  |  |  |  |  |
| 1250159 | aaaattattgaaaa | 1250199 | + | 24.9 | GLAM2Scan_Hit_59 | SAOUHSC_01300 / SAOUHSC_01301 | Overlap / 10 bp 5' | SAOUHSC_01300 | 1249947 | + | 1250193 | -246 | 42.33 | 0.00E+00 |  |  |  |  |  |  |
|  |  |  |  |  |  |  |  | SAOUHSC_01301 | 1250210 | + | 1250193 | 17 | 42.33 | 0.00E+00 | None |  |  | None |  |  |
| 815972 | aaaattatctaaac | 815935 | - | 24.8 | GLAM2Scan_Hit_60 | SAOUHSC_00845 | 53 bp 5' | SAOUHSC_00845 | 815930 | + | 816026 | -96 | NA | NA |  |  |  |  |  |  |
| 1838407 | ataaatattttaaata | 1838371 | - | 24.8 | GLAM2Scan_Hit_61 | SAOUHSC_01931 | Overlap | SAOUHSC_01931 | 1838485 | - | 1837999 | -486 | 53.54 | 0.00E+00 |  |  |  |  |  |  |
| 2520487 | aacaatatcataaa | 2520523 | + | 24.8 | GLAM2Scan_Hit_62 | Same location as 101 SAOUHSC_02740 (MFS drug transporter) | 58 bp to 5' | SAOUHSC_02740 | 2520428 | - | 2520496 | 68 | 16.54 | 9.79E-62 |  |  |  |  |  |  |
| 805408 | aaaattattaacaat | 805372 | - | 24.7 | GLAM2Scan_Hit_63 | SAOUHSC_00828 | Overlap | SAOUHSC_00828 | 805932 | - | 805285 | -647 | 30.19 | 1.84E-200 |  |  |  |  |  |  |
| 1077555 | aaaagttaaaaac | 1077519 | - | 24.7 | GLAM2Scan_Hit_64 | SAOUHSC_01121 / srn_2480 sRNA205/ srn_2490 sRNA206 | 146 bp to 5' / Overlap | SAOUHSC_01121 | 1077372 | - | 1077764 | 392 | 50.77 | 0.00E+00 |  |  |  |  |  |  |
|  |  |  |  |  |  |  |  | srn_2480 | 1077692 | - | 1077764 | 72 | 50.77 | 0.00E+00 |  |  |  |  |  |  |
|  |  |  |  |  |  |  |  | srn_2490 | 1077719 | + | 1077764 | -45 | 50.77 | 0.00E+00 |  |  |  |  |  |  |
| 1848240 | aaaaatgtattaac | 1848199 | - | 24.7 | GLAM2Scan_Hit_65 | Same location as 23 SAOUHSC_01944 | 84 bp 5' | SAOUHSC_01944 | 1848325 | + | 1848234 | 91 | 41.93 | 0.00E+00 |  |  |  |  |  |  |
| 2628633 | gcgaattatttaaat | 2628596 | - | 24.7 | GLAM2Scan_Hit_66 | SAOUHSC_02855 (LysM domain-containing protein) / SAOUH | 154 bp 3' / 72 bp 3' | SAOUHSC_02855 | N/A |  |  |  | NA | NA |  |  |  |  |  |  |
|  |  |  |  |  |  |  |  | SAOUHSC_02856 | N/A |  |  |  | NA | NA |  |  |  |  |  |  |
| 1826122 | gcaattcaatacatt | 1826158 | + | 24.6 | GLAM2Scan_Hit_67 | SAOUHSC_01917 | Overlap | SAOUHSC_01917 | 1826364 | - | 1826415 | 51 | NA | NA |  |  |  |  |  |  |
| 1540379 | acaaatatgacaa | 1540343 | - | 24.6 | GLAM2Scan_Hit_68 | SAOUHSC_01615 (DNA repair protein RecN) | Overlap | SAOUHSC_01615 | 1541166 | - | 1540156 | -1010 | 13.35 | 5.89E-41 |  |  |  |  |  |  |
| 483194 | aaaaatatgaaaa | 483232 | + | 24.6 | GLAM2Scan_Hit_69 | New sRNA srn_1270 Teg44 | On 5' | srn_1270 | 483226 | + | 483137 | 89 | 45.02 | 0.00E+00 |  |  |  |  |  |  |

| 908696 | aaagaaataaaac | 908732 | + | 24.6 | GLAM2Scan_Hit_70 |  | SAOUHSC_00936 | Overlap | SAOUHSC_00936 | 908430 | + | 908622 | -192 | 8.08 | 3.15E-16 |
| --- | --- | --- | --- | --- | --- | --- | --- | --- | --- | --- | --- | --- | --- | --- | --- |
| 2707997 | acaattatttcattaa | 2708033 | + | 24.6 | GLAM2Scan_Hit_71 | Same location as 178 | SAOUHSC_02942 (anaerobic ribonucleoside triphosphate redu | 133 bp 5' | SAOUHSC_02942 | 2707866 | - | 2707965 | 99 | 19.32 | 1.77E-83 |
| 49395 | ataaatataataaa | 49433 | + | 24.6 | GLAM2Scan_Hit_72 | Same location as 131 | SAOUHSC_00047 | 56 bp to 5' | SAOUHSC_00047 | 49490 | + | 49366 | 124 | 39.44 | 0.00E+00 |
| 2010044 | aaaaataagacaa | 2010082 | + | 24.5 | GLAM2Scan_Hit_73 |  | SAOUHSC_02137 (sodium-dependent transporter) | Overlap | SAOUHSC_02137 | 2011290 | - | 2010275 | -1015 | 4.67 | 1.48E-06 |
| 101167 | acaaataaataaa | 101204 | + | 24.5 | GLAM2Scan_Hit_74 |  | SAOUHSC_00094 / SAOUHSC_00096 (GntR family transcripti | 112 bp 3' / 165 bp 3' | SAOUHSC_00094 | N/A |  | None |  | NA | NA |
|  |  |  |  |  |  |  |  |  | SAOUHSC_00096 | N/A |  |  |  | NA | NA |
| 136686 | aaaaatttataaag | 136648 | - | 24.5 | GLAM2Scan_Hit_75 |  | SAOUHSC_00131 | Overlap | SAOUHSC_00131 | 136841 | - | None |  | NA | NA |
| 962121 | aaatataaatcaaa | 962157 | + | 24.5 | GLAM2Scan_Hit_76 |  | SAOUHSC_00989 | 12 bp 5' | SAOUHSC_00989 | 962108 | - | 961530 | -578 | 9.73 | 1.09E-22 |
| 1006369 | aaaaattagtgaaa | 1006404 | + | 24.4 | GLAM2Scan_Hit_77 |  | SAOUHSC_01037 | 4 bp 5' | SAOUHSC_01037 | 1006364 | - | 1006457 | 93 | 32.37 | 3.73E-230 |
| 2084568 | aaaaacataacaa | 2084606 | + | 24.4 | GLAM2Scan_Hit_78 |  | SAOUHSC_02249 | Overlap | SAOUHSC_02249 | 2084941 | - | 2084592 | -349 | 33.08 | 2.47E-240 |
| 1974470 | aaaaataaaaaaa | 1974509 | + | 24.4 | GLAM2Scan_Hit_79 | vraS | SAOUHSC_02099 | Overlap | SAOUHSC_02099 | 1974624 | - | 1974252 | -372 | 3.87 | 5.38E-05 |
| 375292 | aaaaatttattcaaa | 375329 | + | 24.3 | GLAM2Scan_Hit_80 |  | None Close |  |  |  |  |  |  | NA | NA |
| 799052 | ataaatatattattaa | 799087 | + | 24.3 | GLAM2Scan_Hit_81 |  | SAOUHSC_00817 | 135 bp 5' | SAOUHSC_00817 | 799223 | + | 799059 | 164 | 34.28 | 8.61E-258 |
| 1300192 | aaaaattaattaatt | 1300229 | + | 24.3 | GLAM2Scan_Hit_82 |  | SAOUHSC_01358 | Overlap | SAOUHSC_01358 | 1299795 | + | 1300212 | -417 | 16.04 | 3.62E-58 |
| 2486742 | atgaataaattagtt | 2486704 | - | 24.3 | GLAM2Scan_Hit_83 | Same location as 107 | SAOUHSC_02704 | 72 bp 5' | SAOUHSC_02704 | 2486631 | - | 2486704 | 73 | 28.12 | 3.09E-174 |
| 558211 | aaaattatattacta | 558175 | - | 24.2 | GLAM2Scan_Hit_84 |  | SAOUHSC_00548 | Overlap | SAOUHSC_00548 | 558359 | - | 558426 | 67 | 41.66 | 0.00E+00 |
| 2610297 | ataaatatatcgtta | 2610260 | - | 24.2 | GLAM2Scan_Hit_85 |  | SAOUHSC_02831 | 160 bp 5' | SAOUHSC_02831 | 2610099 | - | 2610355 | 256 | 36.73 | 1.32E-295 |
| 889104 | aaaaattaataacg | 889067 | - | 24.2 | GLAM2Scan_Hit_86 |  | SAOUHSC_00916 | Overlap | SAOUHSC_00916 | 888046 | + | 888744 | -698 | 48.02 | 0.00E+00 |
| 808866 | gaaacttaattacat | 808902 | + | 24.2 | GLAM2Scan_Hit_87 |  | SAOUHSC_00833 | On 3' | SAOUHSC_00833 | N/A |  |  |  | NA | NA |
| 547326 | aaaaatatgtaaat | 547361 | + | 24.1 | GLAM2Scan_Hit_88 | Same location as 50, | SAOUHSC_00543/SAOUHSC_00544 | 5 bp 3' | SAOUHSC_00543 | N/A |  |  |  | NA | NA |
| 799965 | acaattaattaataa | 799929 | - | 24 | GLAM2Scan_Hit_89 | Same location as 26, | SAOUHSC_00818 thermonuclease | 68 bp to 5' | SAOUHSC_00818 | 800034 | + | 799858 | 176 | 42.72 | 0.00E+00 |
| 2288241 | aaaattaatataata | 2288205 | - | 23.9 | GLAM2Scan_Hit_90 |  | SAOUHSC_02464 | On 5' | SAOUHSC_02464 | 2288214 | - | 2288278 | 64 | 25.69 | 7.52E-146 |
| 1441990 | aaaaatataatcaa | 1442025 | + | 23.9 | GLAM2Scan_Hit_91 |  | SAOUHSC_01484 | Overlap | SAOUHSC_01484 | 1442162 | - | 1441901 | -261 | 51.18 | 0.00E+00 |
| 806624 | aaaaatttcttaatta | 806661 | + | 23.9 | GLAM2Scan_Hit_92 |  | SAOUHSC_00830 | 42 bp 5' | SAOUHSC_00830 | 806581 | - | 806684 | 103 | 37.77 | 1.95E-312 |
| 1081657 | aaagttaaaaaact | 1081618 | - | 23.9 | GLAM2Scan_Hit_93 |  | SAOUHSC_01128 (ornithine carbamoyltransferase) | On 5' | SAOUHSC_01128 | 1081623 | + | 1081454 | 169 | 38.65 | 0.00E+00 |
| 50247 | ataaatatggtaatg | 50287 | + | 23.9 | GLAM2Scan_Hit_94 | Same location as 167 | SAOUHSC_00049 | 71 bp 5' | SAOUHSC_00049 | 50359 | + | 50268 | 91 | 29.44 | 7.88E-191 |
| 893898 | aaaaaaatattgat | 893934 | + | 23.8 | GLAM2Scan_Hit_95 |  | SAOUHSC_00922 / SAOUHSC_00923 | 129 bp 5' / 76 bp 5' | SAOUHSC_00922 | 893768 | - | 893970 | 202 | 43.71 | 0.00E+00 |
|  |  |  |  |  |  |  |  |  | SAOUHSC_00923 | 894011 | + | 893970 | 41 | 43.71 | 0.00E+00 |
| 275790 | ataaatatattgaat | 275753 | - | 23.8 | GLAM2Scan_Hit_96 |  | SAOUHSC_00256 / SAOUHSC_00257 | 69 bp 5' / 140 bp 5' | SAOUHSC_00256 | 275683 | - | 275734 | 51 | 37.08 | 2.51E-301 |
|  |  |  |  |  |  |  |  |  | SAOUHSC_00257 | 275931 | + | 275734 | 197 | 37.08 | 2.51E-301 |
| 1352734 | gtaaatataagaac | 1352771 | + | 23.8 | GLAM2Scan_Hit_97 |  | SAOUHSC_01412 | Overlap | SAOUHSC_01412 | 1353310 | - | 1352725 | -585 | 18.16 | 5.27E-74 |
| 2032794 | ataaataaataattt | 2032757 | - | 23.8 | GLAM2Scan_Hit_98 |  | SAOUHSC_02166 | 5 bp 5' | SAOUHSC_02166 | 2032800 | + | None |  | NA | NA |
| 394065 | aatcatataaaaat | 394102 | + | 23.8 | GLAM2Scan_Hit_99 |  | SAOUHSC_00391 (superantigen-like protein) | 18 bp 5' | SAOUHSC_00391 | 394121 | + | 394069 | 52 | 5.11 | 1.61E-07 |
| 946374 | agcaatataataat | 946410 | + | 23.7 | GLAM2Scan_Hit_100 |  | SAOUHSC_00971 | Overlap | SAOUHSC_00971 | 946633 | - | 946792 | 159 | 46.89 | 0.00E+00 |
| 2520514 | ataaataaaacatc | 2520554 | + | 23.7 | GLAM2Scan_Hit_101 | Same location as 62 | SAOUHSC_02740 (MFS drug transporter) | 85 bp 5' | SAOUHSC_02740 | 2520428 | - | 2520496 | 68 | 16.54 | 9.79E-62 |
| 2294753 | aaaaatatattgatg | 2294716 | - | 23.7 | GLAM2Scan_Hit_102 |  | SAOUHSC_02471 | Overlap | SAOUHSC_02471 | 2295324 | - | 2295059 | -265 | 53.31 | 0.00E+00 |
| 1487923 | acaaattaattaata | 1487959 | + | 23.7 | GLAM2Scan_Hit_103 |  | SAOUHSC_01539 (terminase small subunit) | Overlap | SAOUHSC_01539 | 1487998 | - | 1488110 | 112 | 12.59 | 1.20E-36 |
| 169032 | aaacataatataag | 169070 | + | 23.6 | GLAM2Scan_Hit_104 | New sRNA | srn_0440 rsaK | 52 bp 5' | srn_0440 | 168979 | - | 169030 | 51 | 21.77 | 2.23E-105 |
| 342776 | agcaacattttaatt | 342740 | - | 23.6 | GLAM2Scan_Hit_105 |  | SAOUHSC_00329 (mttA/Hcf106 family protein-like protein) | 51 bp 5' | SAOUHSC_00329 | 342688 | - | 342733 | 45 | 21.29 | 6.75E-101 |
| 679937 | aaagataatttaata | 679975 | + | 23.6 | GLAM2Scan_Hit_106 |  | SAOUHSC_00695 | 25 bp 5' | SAOUHSC_00695 | 680001 | + | 679931 | 70 | 46.55 | 0.00E+00 |
| 2486738 | ataaattagtttaatt | 2486702 | - | 23.6 | GLAM2Scan_Hit_107 | Same location as 83 | SAOUHSC_02704 | 72 bp 5' | SAOUHSC_02704 | 2486631 | - | 2486704 | 73 | 28.12 | 3.09E-174 |
| 2741853 | aaagatttgaaaaa | 2741817 | - | 23.6 | GLAM2Scan_Hit_108 |  | SAOUHSC_02978 (phage infection protein) | Overlap | SAOUHSC_02978 | 2744058 | - | 2743336 | -722 | 12.44 | 7.94E-36 |
| 1846693 | acaattaaaagaat | 1846655 | - | 23.5 | GLAM2Scan_Hit_109 |  | SAOUHSC_01941 (serine protease SplB) / SAOUHSC_01942 | 126 bp 5' / On 3' | SAOUHSC_01941 | 1846529 | - | 1846639 | 110 | 27.20 | 2.87E-163 |
|  |  |  |  |  |  |  |  |  | SAOUHSC_01942 | N/A |  |  |  | NA | NA |
| 2801213 | aaaattaaattaac | 2801175 | - | 23.5 | GLAM2Scan_Hit_110 | Same location as 169 | SAOUHSC_03030 (sodium, sulfate symporter) | 88 bp 5' | SAOUHSC_03030 | 2801302 | + | 2801192 | 110 | 25.17 | 4.56E-140 |
| 50611 | gaaattaatttgaaa | 50647 | + | 23.5 | GLAM2Scan_Hit_111 |  | SAOUHSC_00049 | Overlap | SAOUHSC_00049 | 50359 | + | 50934 | -575 | 20.94 | 1.06E-97 |
| 1914776 | aaaaatatgtattta | 1914741 | - | 23.4 | GLAM2Scan_Hit_112 |  | SAOUHSC_02004 | 45 bp 5' | SAOUHSC_02004 | 1914695 | - | 1914808 | 113 | 42.16 | 0.00E+00 |
| 2330345 | aaaaatattttactc | 2330381 | + | 23.4 | GLAM2Scan_Hit_113 |  | SAOUHSC_02525 | 27 bp 5' | SAOUHSC_02525 | 233317 | - | None |  | NA | NA |
| 963387 | acaaatttgttgcat | 963350 | - | 23.4 | GLAM2Scan_Hit_114 | Same location as 58 | SAOUHSC_00991 / SAOUHSC_00992 (MarR family transcripti | 35 bp 5' / 79 bp 5' | SAOUHSC_00991 | 963314 | - | 963390 | 76 | 38.30 | 2.88E-321 |
|  |  |  |  |  |  |  |  |  | SAOUHSC_00992 | 963467 | + | 963390 | 77 | 38.30 | 2.88E-321 |
| 1844228 | gaaattaagaaatt | 1844191 | - | 23.4 | GLAM2Scan_Hit_115 |  | SAOUHSC_01938 (serine protease SplD) / SAOUHSC_01937 | On 3' / On 3' |  |  |  |  |  | NA | NA |
| 76230 | aaaaataatatgat | 76267 | + | 23.4 | GLAM2Scan_Hit_116 | sarS (Same location a SAOUHSC_00070 | | 77 bp 5' | SAOUHSC_00070 |  |  |  |  | NA | NA |
| 1461832 | aaaagtatgacaa | 1461797 | - | 23.3 | GLAM2Scan_Hit_117 | SAOUHSC_01512 | | Overlap | SAOUHSC_01512 | 1462280 | - | None |  | NA | NA |
| 1374205 | aaaaatattataac | 1374245 | + | 23.3 | GLAM2Scan_Hit_118 | SAOUHSC_01439 | | 36 bp 5' | SAOUHSC_01439 | 1374168 | - | 1374212 | 44 | 4.38 | 6.02E-06 |
| 2629149 | acaaatatataaatt | 2629113 | - | 23.3 | GLAM2Scan_Hit_119 | SAOUHSC_02857 | | 5 bp 5' | SAOUHSC_02857 | 2629107 | - | None |  | NA | NA |
| 1196703 | aagaataatagaa | 1196739 | + | 23.3 | GLAM2Scan_Hit_120 | SAOUHSC_01246 infB (translation initiation factor IF-2) | | Overlap | SAOUHSC_01246 | 1196412 | + | 1196706 | -294 | 20.08 | 5.22E-90 |
| 1249269 | aaaaatattaaaaa | 1249305 | + | 23.3 | GLAM2Scan_Hit_121 | SAOUHSC_01296 / SAOUHSC_01297 | | On 3' / 116 bp 5' | SAOUHSC_01296 | N/A |  |  |  | NA | NA |
|  |  |  |  |  |  |  | |  | SAOUHSC_01297 | 1249422 | + | 1249191 | 231 | 42.56 | 0.00E+00 |
| 1773695 | gaaaattttataattt | 1773732 | + | 23.3 | GLAM2Scan_Hit_122 | New sRNA, same locasrn_3470 | | 0 bp 5' | srn_3470 | 1773694 | - | 1773694 | 0 | NA | NA |
| 22092 | acagttaagttaca | 22128 | + | 23.3 | GLAM2Scan_Hit_123 | SAOUHSC_00018 (replicative DNA helicase) | | Overlap | SAOUHSC_00018 | 20766 | + | None |  | NA | NA |
| 1826529 | aaaaatatttttaaa | 1826565 | + | 23.3 | GLAM2Scan_Hit_124 | SAOUHSC_01917 / SAOUHSC_01918 | | 169 bp 5' / 101 bp 5' | SAOUHSC_01917 | 1826364 | - | 1826419 | 55 | 45.79 | 0.00E+00 |
|  |  |  |  |  |  |  | |  | SAOUHSC_01918 | 1826667 | + | 1826419 | 248 | 45.79 | 0.00E+00 |
| 2408310 | aaaaataaatt.cat | 2408276 | - | 23.2 | GLAM2Scan_Hit_125 | SAOUHSC_02619 | | 53 bp 5' | SAOUHSC_02619 | 2408222 | - | 2408273 | 51 | 32.60 | 1.80E-233 |
| 2298236 | aaaaatatttcaatt | 2298199 | - | 23.2 | GLAM2Scan_Hit_126 | SAOUHSC_02476 | | Overlap | SAOUHSC_02476 | 2298782 | - | 2297938 | -844 | 33.13 | 5.30E-241 |
| 2080901 | aaaaatatataatc | 2080945 | + | 23.2 | GLAM2Scan_Hit_127 | SAOUHSC_02246 | | Overlap | SAOUHSC_02246 | 2081634 | - | 2081710 | 76 | 43.18 | 0.00E+00 |
| 68140 | aaaaatataaaaat | 68098 | - | 23.2 | GLAM2Scan_Hit_128 | SAOUHSC_00061 (myosin-cross-reactive antigen) | | 70 bp 5' | SAOUHSC_00061 | 68027 | - | 68177 | 150 | NA | NA |
| 140744 | aaggatattaagat | 140708 | - | 23.2 | GLAM2Scan_Hit_129 | Same location as 187 SAOUHSC_00134 / SAOUHSC_00135 | | 82 bp to 5' / 59 bp to 5' | SAOUHSC_00134 | 140625 | - | 140683 | 58 | 23.18 | 3.35E-119 |
|  |  |  |  |  |  |  | |  | SAOUHSC_00135 | 140804 | + | 140683 | 121 | 23.18 | 3.35E-119 |
| 547306 | gaacatttaaaata | 547342 | + | 23.2 | GLAM2Scan_Hit_130 | Same location as 50, SAOUHSC_00543/ SAOUHSC_00544 | | On 3' | SAOUHSC_00543 | N/A |  |  |  | NA | NA |
| 49404 | ataaattaaacaag | 49442 | + | 23.1 | GLAM2Scan_Hit_131 | Same location as 72 SAOUHSC_00047 | | 47 bp 5' | SAOUHSC_00047 | 49490 | + | 49366 | 124 | 39.44 | 0.00E+00 |
| 357824 | ggaagtatataaaa | 357861 | + | 23.1 | GLAM2Scan_Hit_132 | SAOUHSC_00344 | | 24 bp 5' | SAOUHSC_00344 | 357886 | + | 357766 | 120 | 18.09 | 1.76E-73 |
| 963410 | aaaaat.tataaaa | 963444 | + | 23.1 | GLAM2Scan_Hit_133 | Same location as 58, SAOUHSC_00991 / SAOUHSC_00992 (MarR family transcripti | | 95 bp 5' / 22 bp 5' | SAOUHSC_00991 | 963314 | - | 963390 | 76 | 38.30 | 2.88E-321 |
|  |  |  |  |  |  |  | |  | SAOUHSC_00992 | 963467 | + | 963390 | 77 | 38.30 | 2.88E-321 |
| 2594967 | aaaagttaagcgg | 2594931 | - | 23.1 | GLAM2Scan_Hit_134 | SAOUHSC_02815 | | 151 bp 5' | SAOUHSC_02815 | 2594779 | - | 2594967 | 188 | 33.95 | 5.45E-253 |
| 1861615 | aaaaataaaatatt | 1861578 | - | 23 | GLAM2Scan_Hit_135 | Used for consensus, srn_3610 sprC | | On 5' | srn_3610 | 1861598 | - | 1861615 | 17 | 49.88 | 0.00E+00 |
| 1201793 | acaaatattaacatt | 1201829 | + | 23 | GLAM2Scan_Hit_136 | rpsO (30S ribosomal protein S15) | | 71 bp 3' | SAOUHSC_01250 |  |  |  |  | NA | NA |
| 1773724 | aaatataatgataa | 1773688 | - | 23 | GLAM2Scan_Hit_137 | Same location as 122 srn_3470 | | On 5' | srn_3470 | 1773694 | - | 1773694 | 0 | NA | NA |
| 1371421 | atacttattaaaata | 1371384 | - | 23 | GLAM2Scan_Hit_138 | thyA (thymidylate synthase) | | Overlap | SAOUHSC_01435 | 1371645 | - | 1371736 | 91 | 31.65 | 3.98E-220 |
| 1086722 | aaaaatcttttaaat | 1086759 | + | 23 | GLAM2Scan_Hit_139 | SAOUHSC_01133 | | Overlap | SAOUHSC_01133 | 1087094 | - | 1086786 | -308 | 23.35 | 7.64E-121 |
| 1338078 | ataataatttccaatt | 1338043 | - | 23 | GLAM2Scan_Hit_140 | SAOUHSC_01395 (aspartate semialdehyde dehydrogenase) | | 18 bp 5' | SAOUHSC_01395 | 1338097 | + | 1338053 | 44 | 25.97 | 5.10E-149 |
| 2383302 | acaattattttgtgat | 2383339 | + | 23 | GLAM2Scan_Hit_141 | SAOUHSC_02590 / SAOUHSC_02591 | | 154 bp 5' / 87 bp 5' | SAOUHSC_02590 | 2383147 | - | 2383274 | 127 | 31.50 | 5.08E-218 |
|  |  |  |  |  |  |  | |  | SAOUHSC_02591 | 2383427 | + | 2383274 | 153 | 31.50 | 5.08E-218 |
| 1567861 | aaaaataattaaac | 1567899 | + | 23 | GLAM2Scan_Hit_142 | SAOUHSC_01653 (superoxide dismutase) | | 122 bp 5' | SAOUHSC_01653 | 1567738 | - | 1567879 | 141 | 14.26 | 1.97E-46 |
| 886210 | gataatatttaattttt | 886249 | + | 22.9 | GLAM2Scan_Hit_143 | Same location as 184 SAOUHSC_00913 / SAOUHSC_00914 (2-isopropylmalate synt | | 36 bp 5' / 33 bp 5' | SAOUHSC_00913 | 886173 | - | 886231 | 58 | 50.43 | 0.00E+00 |
|  |  |  |  |  |  |  | |  | SAOUHSC_00914 | 886283 | + | 886231 | 52 | 50.43 | 0.00E+00 |
| 2152181 | aaaaatatgaacat | 2152144 | - | 22.9 | GLAM2Scan_Hit_144 | ddl (D-alanyl-alanine synthetase A) | | Overlap | SAOUHSC_02318 | 2152647 | - | 2152762 | 115 | 8.06 | 3.95E-16 |
| 1511605 | ttaaatatattactaa | 1511570 | - | 22.9 | GLAM2Scan_Hit_145 | SAOUHSC_01584 | | 140 bp 5' | SAOUHSC_01584 | 1511429 | - | 1511554 | 125 | 40.98 | 0.00E+00 |
| 592745 | acaaatattgaaaa | 592780 | + | 22.9 | GLAM2Scan_Hit_146 | SAOUHSC_00596 | | Overlap | SAOUHSC_00596 | 592358 | + | 592185 | 173 | 4.95 | 3.78E-07 |
| 2687748 | aaaaatataatgtg | 2687789 | + | 22.9 | GLAM2Scan_Hit_147 | SAOUHSC_02923 | | 202 bp 5' | SAOUHSC_02923 | 2687992 | + | 2687833 | 159 | 27.94 | 3.92E-172 |
| 597310 | ataaatatataaatt | 597346 | + | 22.8 | GLAM2Scan_Hit_148 | SAOUHSC_00605 | | 62 bp 5' | SAOUHSC_00605 | 597409 | + | 597311 | 98 | 19.21 | 1.67E-82 |
| 1410381 | aataatatattaaat | 1410345 | - | 22.8 | GLAM2Scan_Hit_149 | SAOUHSC_01452 (alanine dehydrogenase) | | 94 bp 5' | SAOUHSC_01452 | 1410250 | - | 1410443 | 193 | 52.15 | 0.00E+00 |
| 2454009 | aaaaatttaataac | 2454047 | + | 22.8 | GLAM2Scan_Hit_150 | SAOUHSC_02668 / SAOUHSC_02669 | | 135 bp 5' / 223 bp 5' | SAOUHSC_02668 | 2453873 | - | 2454090 | 217 | 38.02 | 1.14E-316 |
|  |  |  |  |  |  |  | |  | SAOUHSC_02669 | 2454270 | + | 2454090 | 180 | 38.02 | 1.14E-316 |
| 1612364 | aaaaataaaagaa | 1612328 | - | 22.8 | GLAM2Scan_Hit_151 | SAOUHSC_01705 | | 253 bp 5' | SAOUHSC_01705 | 1612074 | - | 1612530 | 456 | 43.29 | 0.00E+00 |
| 2424367 | aaatttatatgaaaa | 2424331 | - | 22.8 | GLAM2Scan_Hit_152 | SAOUHSC_02638 | | Overlap | SAOUHSC_02638 | 2424092 | + | 2424069 | 23 | 36.83 | 3.31E-297 |
| 716158 | aaggataaatcatt | 716194 | + | 22.8 | GLAM2Scan_Hit_153 | SAOUHSC_00731 (ABC transporter) | | 67 bp 5' | SAOUHSC_00731 | 716262 | + | 716194 | 68 | 14.99 | 4.27E-51 |
| 1884795 | acaattaaaacaca | 1884759 | - | 22.7 | GLAM2Scan_Hit_154 | SAOUHSC_01980 (DNA-binding response regulator) | | Overlap | SAOUHSC_01980 | 1885323 | - | None |  | NA | NA |
| 48556 | aaaaataaaatga | 48591 | + | 22.7 | GLAM2Scan_Hit_155 | SAOUHSC_00046 | | Overlap | SAOUHSC_00046 | 48313 | + | 48597 | -284 | 33.21 | 4.06E-242 |
| 163344 | gcaaataataaaat | 163379 | + | 22.7 | GLAM2Scan_Hit_156 | SAOUHSC_00151 (branched-chain amino acid transport syste | | Overlap | SAOUHSC_00151 | 163849 | - | 163904 | 55 | 29.42 | 1.46E-190 |
| 332838 | aaaaataaattgct | 332880 | + | 22.7 | GLAM2Scan_Hit_157 | SAOUHSC_00317 (glycerol-3-phosphate transporter) / SAOUH | | 133 bp 5' / 131 bp 5' | SAOUHSC_00317 | 332704 | - | 332828 | 124 | 12.27 | 6.24E-35 |

| 0.88975 | + | 7.72E-01 | 1.20761 | - | 6.66E-01 |
| --- | --- | --- | --- | --- | --- |
| 1.75562 | - | 1.29E-07 | 8.63256 | - | 1.87E-24 |
| 1.33268 | - | 5.69E-02 | 3.50813 | - | 3.74E-09 |
| 0.9791 | + | 1.00E+00 | 1.00685 | - | 9.76E-01 |
| 0.45703 | + | 2.98E-14 | 0.39176 | + | 1.21E-04 |
| 0.74508 | + | 2.11E-02 | 0.59783 | + | 3.81E-02 |
| 1.08752 | - | 7.75E-01 | 0.53626 | + | 8.04E-02 |
| 1.5648 | - | 4.18E-05 | 4.80626 | - | 4.97E-14 |
| 0.80676 | + | 1.11E-01 | 0.99483 | + | 9.21E-01 |
| 1.03281 | - | 7.82E-01 | 0.36069 | + | 1.45E-05 |
| 0.99595 | + | 1.00E+00 | 1.14619 | - | 6.68E-01 |
|  |  | |  |  | |
| 1.24437 | - | 1.60E-01 | 0.63828 | + | 3.88E-01 |
| 1.16376 | - | 2.38E-01 | 1.18176 | - | 6.02E-01 |
| 1.29895 | - | 3.38E-02 | 4.24235 | - | 2.03E-13 |
| 1.47036 | - | 2.31E-04 | 0.61923 | + | 5.39E-02 |
| 0.81049 | + | 2.58E-01 | 0.41501 | + | 2.78E-04 |
| 1.76079 | - | 3.83E-04 | 1.01532 | - | 1.00E+00 |
| 1.07398 | - | 6.84E-01 | 0.9233 | + | 8.48E-01 |
| 0.92257 | + | 5.91E-01 | 0.89695 | + | 7.11E-01 |
| 130.10088 | - | 4.46E-277 | 269.37109 | - | 4.52E-115 |
| 0.80167 | + | 8.99E-02 | 0.4806 | + | 1.85E-03 |
| 1.33136 | - | 6.14E-01 | 0.36115 | + | 1.28E-02 |
| 1.40997 | - | 1.57E-03 | 2.57996 | - | 3.07E-05 |
| 1.67979 | - | 1.17E-02 | 0.71784 | + | 1.93E-01 |
| 2.09776 | - | 7.04E-13 | 1.41272 | - | 1.72E-01 |
| 0.79824 | + | 5.96E-01 | 1.34523 | - | 4.28E-01 |
| 0.6471 | + | 1.26E-03 | 3.17929 | - | 2.42E-08 |
| 7.24342 | - | 6.77E-65 | 3.83755 | - | 1.32E-11 |
| 18.01329 | - | 6.41E-155 | 8.88514 | - | 1.18E-24 |
| 1.04229 | - | 8.28E-01 | 1.76912 | - | 9.88E-03 |
| 1.2582 | - | 5.33E-02 | 1.47581 | - | 1.44E-01 |
| 0.84053 | + | 5.69E-01 | 0.94623 | + | 9.64E-01 |
| 0.19759 | + | 9.56E-57 | 0.19202 | + | 6.83E-12 |
| 1.32053 | - | 1.94E-02 | 3.03244 | - | 2.75E-07 |
| 0.99678 | + | 1.00E+00 | 0.84476 | + | 5.81E-01 |
| 0.32747 | + | 6.06E-01 | 1.34821 | - | 9.99E-01 |
| 1.33943 | - | 8.84E-03 | 1.89445 | - | 4.95E-03 |
| 1.50837 | - | 3.93E-03 | 2.93759 | - | 8.88E-07 |
| 0.5966 | + | 1.95E-06 | 0.67686 | + | 1.17E-01 |
| 1.29895 | - | 3.38E-02 | 4.24235 | - | 2.03E-13 |
| 1.10653 | - | 4.45E-01 | 1.6279 | - | 3.30E-02 |
| 17.51075 | - | 1.22E-34 | 52.41194 | - | 5.50E-75 |
| 24.271 | - | 3.00E-50 | 62.27935 | - | 2.62E-77 |
| 1.37197 | - | 3.33E-03 | 0.84473 | + | 6.08E-01 |
| 2.09776 | - | 7.04E-13 | 1.41272 | - | 1.72E-01 |
| 1.17162 | - | 2.17E-01 | 0.69884 | + | 2.19E-01 |
| 0.60924 | + | 2.14E-06 | 0.94202 | + | 8.67E-01 |
| 2.45627 | - | 5.07E-19 | 2.32279 | - | 6.09E-05 |
| 17.29416 | - | 8.53E-136 | 28.67278 | - | 1.53E-54 |
|  |  | |  |  | |
| 0.96048 | + | 8.52E-01 | 0.88959 | + | 7.01E-01 |
| 0.88525 | + | 1.00E+00 | 0.97191 | + | 1.00E+00 |
| 0.9157 | + | 5.46E-01 | 1.37193 | - | 2.13E-01 |
| 0.88738 | + | 6.92E-01 | 8.45639 | - | 2.74E-20 |
| 0.95341 | + | 8.65E-01 | 0.88233 | + | 7.59E-01 |
| 2.18364 | - | 2.17E-02 | 1.68313 | - | 2.33E-01 |
| 1.29347 | - | 3.66E-01 | 2.80493 | - | 1.30E-02 |
| 0.85907 | + 2.65E-01 | | 0.90763 | + 7.49E-01 | |
| 0.80839 | + | 6.73E-02 | 1.12472 | - | 6.78E-01 |
| 6.3261 | - | 4.48E-28 | 5.3767 | - | 6.32E-11 |
| 0.96533 | + | 9.32E-01 | 0.30412 | + | 1.47E-06 |
| 2.3875 | - | 4.09E-14 | 1.31965 | - | 2.85E-01 |
| 0.70897 | + | 1.39E-02 | 1.65796 | - | 2.74E-02 |
| 2.70575 | - | 6.72E-07 | 1.20429 | - | 7.11E-01 |
| 11.29152 | - | 1.58E-116 | 21.4663 | - | 1.86E-46 |
| 0.3104 | + | 2.55E-19 | 0.22919 | + | 7.60E-10 |
| 0.37021 | + | 3.51E-19 | 0.72414 | + | 2.07E-01 |
| 0.92257 | + | 5.91E-01 | 0.89695 | + | 7.11E-01 |
| 1.33268 | - | 5.69E-02 | 3.50813 | - | 3.74E-09 |
| 1.14859 | - | 3.49E-01 | 1.41986 | - | 1.42E-01 |
| 2.45627 | - | 5.07E-19 | 2.32279 | - | 6.09E-05 |
| 17.29416 | - | 8.53E-136 | 28.67278 | - | 1.53E-54 |
| 1.61644 | - | 5.47E-06 | 2.5528 | - | 8.70E-05 |
| 7.09485 | - | 4.72E-64 | 8.90355 | - | 6.84E-28 |
| 1.05683 | - | 6.93E-01 | 0.7585 | + | 3.81E-01 |
| 0.85907 | + 2.65E-01 | | 0.90763 | + 7.49E-01 | |
| 0.97649 | + | 9.47E-01 | 0.82526 | + | 5.02E-01 |
| 0.68474 | + | 6.75E-04 | 0.89961 | + | 7.41E-01 |
| 1.55269 | - | 1.08E-04 | 3.88921 | - | 8.26E-11 |
| 1.10857 | - | 4.75E-01 | 1.99841 | - | 2.90E-03 |
| 1.20008 | - | 1.74E-01 | 0.6377 | + | 4.76E-02 |
| 0.87719 | + | 3.40E-01 | 0.81958 | + | 5.21E-01 |
| 65.05003 | - | 5.18E-208 | 42.8092 | - | 2.02E-71 |
| 2.63057 | - | 2.30E-04 | 2.15773 | - | 3.16E-02 |
| 1.02581 | - | 9.39E-01 | 2.04505 | - | 1.10E-03 |
| 3.07462 | - | 4.59E-11 | 2.32399 | - | 5.56E-05 |
| 0.84257 | + | 3.06E-01 | 0.66648 | + | 1.14E-01 |
| 1.28665 | - | 2.76E-02 | 12.33161 | - | 9.01E-33 |
| 1.28385 | - | 4.61E-02 | 2.17258 | - | 2.53E-04 |
| 16.71008 | - | 5.29E-147 | 38.30989 | - | 9.21E-64 |
| 1.0511 | - | 7.27E-01 | 3.06658 | - | 1.50E-08 |
| 1.77393 | - | 2.74E-08 | 2.09732 | - | 1.32E-03 |
| 0.94829 | + | 9.11E-01 | 0.88291 | + | 6.98E-01 |
| 1.40099 | - | 1.18E-02 | 2.90587 | - | 4.73E-07 |
| 1.50488 | - | 2.03E-04 | 0.78072 | + | 3.65E-01 |
| 1.05274 | - | 8.29E-01 | 1.2733 | - | 3.14E-01 |
| 1.08914 | - | 6.93E-01 | 0.99176 | + | 1.00E+00 |
| 1.74998 | - | 5.54E-08 | 2.46865 | - | 2.60E-05 |
| 1.28105 | - | 4.59E-02 | 2.80191 | - | 8.14E-07 |

|  | | | | | | | | SAOUHSC_00318 | 333011 | + | 332828 | 183 | 12.27 | 6.24E-35 |
| --- | --- | --- | --- | --- | --- | --- | --- | --- | --- | --- | --- | --- | --- | --- |
| 2479137 | taaaataaaaaaat | 2479175 | + | 22.7 | GLAM2Scan_Hit_158 | SAOUHSC_02695 / SAOUHSC_02696 (methicillin resistance d | 195 bp 5' / 30 bp 5' | SAOUHSC_02695 | 2478939 | - | 2479107 | 168 | 41.75 | 0.00E+00 |
|  |  |  |  |  |  |  |  | SAOUHSC_02696 | 2479206 | + | 2479107 | 99 | 41.75 | 0.00E+00 |
| 1245521 | aagaataatttatttt | 1245485 | - | 22.7 | GLAM2Scan_Hit_159 | SAOUHSC_01289 | 5 bp 5' | SAOUHSC_01289 | 1245527 | + | 1245523 | 4 | 46.22 | 0.00E+00 |
| 1562116 | aaaagtaaatgaa | 1562080 | - | 22.7 | GLAM2Scan_Hit_160 | SAOUHSC_01646 (glucokinase) | Overlap | SAOUHSC_01646 | 1562277 | - | 1561539 | -738 | 10.21 | 9.10E-25 |
| 377959 | gcagttattgaaaa | 377995 | + | 22.7 | GLAM2Scan_Hit_161 | Used for Consensus SAOUHSC_00371 / srn_0930 Teg76 | 142 bp ' / 29 bp 5' | SAOUHSC_00371 | 377816 | - | 377912 | 96 | 38.80 | 0.00E+00 |
|  |  |  |  |  |  |  |  | srn_0930 | 378025 | + | 377912 | 113 | 38.80 | 0.00E+00 |
| 314226 | caaaattaataaaa | 314191 | - | 22.7 | GLAM2Scan_Hit_162 | SAOUHSC_00299 / SAOUHSC_00300 (lipase) | 284 bp 5' / 99 bp 5' | SAOUHSC_00299 | 313908 | - | 314115 | 207 | NA | NA |
|  |  |  |  |  |  |  |  | SAOUHSC_00300 | 314326 | + | 314115 | 211 | NA | NA |
| 148672 | ataaatttattaaga | 148707 | + | 22.7 | GLAM2Scan_Hit_163 | New sRNA srn_0380 Teg140_sRNA23 | 33 bp 5' | srn_0380 | 148741 | + | 148645 | 96 | 34.79 | 1.91E-265 |
| 612145 | aaaattataaaaat | 612110 | - | 22.7 | GLAM2Scan_Hit_164 | Verified srn_1550 teg49 | Says overlap, but actually 26 bp 5 | srn_1550 | 612358 | - | 612508 | 150 | 37.82 | 2.95E-313 |
| 1461374 | aaagatgaataaa | 1461338 | - | 22.6 | GLAM2Scan_Hit_165 | SAOUHSC_01511 / SAOUHSC_01512 | 92 bp 5' / On 3' | SAOUHSC_01511 | 1461245 | - | 1461384 | 139 | 17.55 | 2.91E-69 |
|  |  |  |  |  |  |  |  | SAOUHSC_01512 | N/A |  |  |  | NA | NA |
| 1244994 | atatattatataaaa | 1244957 | - | 22.6 | GLAM2Scan_Hit_166 | SAOUHSC_01288 | 118 bp 3' | - |  |  |  |  | NA | NA |
| 50311 | acaaataatttaatc | 50274 | - | 22.6 | GLAM2Scan_Hit_167 | Same location as 94 SAOUHSC_00049 | 47 bp 5' | SAOUHSC_00049 | 50359 | + | 50268 | 91 | 29.44 | 7.88E-191 |
| 2744250 | aaaaatataataat | 2744213 | - | 22.6 | GLAM2Scan_Hit_168 | Same location as 17 SAOUHSC_02978 (phage infection protein) / SAOUHSC_0297 | 154 bp 5' / 18 bp 5' | SAOUHSC_02978 | 2744058 | - | 2744123 | 65 | 33.43 | 2.63E-245 |
|  |  |  |  |  |  |  |  | SAOUHSC_02979 | 2744269 | + | 2744123 | 146 | 33.43 | 2.63E-245 |
| 2801180 | ataattatattaatga | 2801226 | + | 22.6 | GLAM2Scan_Hit_169 | Same location as 110 SAOUHSC_03030 (sodium, sulfate symporter) | 75 bp 5' | SAOUHSC_03030 | 2801302 | + | 2801192 | 110 | 25.17 | 4.56E-140 |
| 2080700 | aaaatttttacaactt | 2080737 | + | 22.5 | GLAM2Scan_Hit_170 | SAOUHSC_02245 / SAOUHSC_02246 | 38 bp 5' / On 3' | SAOUHSC_02245 | 2080661 | - | 2080735 | 74 | 30.42 | 1.71E-203 |
|  |  |  |  |  |  |  |  | SAOUHSC_02246 | see notes |  |  |  | NA | NA |
| 2659696 | taaaatataaaacg | 2659655 | - | 22.5 | GLAM2Scan_Hit_171 | Same location as 55? SAOUHSC_02885 | 246 bp 5' | SAOUHSC_02885 | 2659408 | - | 2659660 | 252 | 39.94 | 0.00E+00 |
| 823845 | aaagttatttaaaag | 823882 | + | 22.5 | GLAM2Scan_Hit_172 | SAOUHSC_00853 | 44 bp 5' | SAOUHSC_00853 | 823800 | - | 824062 | 262 | 24.16 | 2.69E-129 |
| 110183 | aaagttattaaaatt | 110144 | - | 22.5 | GLAM2Scan_Hit_173 | SAOUHSC_00105 (phosphonate ABC transporter substrate-bi | 79 bp 5' / 109 bp 5' | SAOUHSC_00105 | 110064 | - | 110178 | 114 | 43.51 | 0.00E+00 |
|  |  |  |  |  |  |  |  | SAOUHSC_00106 | 110293 | + | 110178 | 115 | 43.51 | 0.00E+00 |
| 991527 | acaaatataataaa | 991565 | + | 22.5 | GLAM2Scan_Hit_174 | SAOUHSC_01019 | Overlap | SAOUHSC_01019 | 991627 | - | 991520 | -107 | 30.15 | 5.46E-200 |
| 2688587 | acaaataaaataat | 2688546 | - | 22.4 | GLAM2Scan_Hit_175 | SAOUHSC_02923 | Overlap | SAOUHSC_02923 | 2687992 | + | 2687833 | 159 | 27.94 | 3.92E-172 |
| 2774323 | ataaattaaatgca | 2774287 | - | 22.4 | GLAM2Scan_Hit_176 | SAOUHSC_03001 (ica operon transcriptional regulator IcaR) | 126 bp 3' | - |  |  |  |  | NA | NA |
| 344308 | ataaataactaaag | 344268 | - | 22.4 | GLAM2Scan_Hit_177 | SAOUHSC_00332 / SAOUHSC_00333 (ABC transporter ATP-b | Overlap / 47 bp 5' | SAOUHSC_00332 | 344356 | + | None |  | NA | NA |
|  |  |  |  |  |  |  |  | SAOUHSC_00333 | 343618 | + | None |  | NA | NA |
| 2708037 | acgaatctttaaac | 2708000 | - | 22.4 | GLAM2Scan_Hit_178 | Same location as 71 SAOUHSC_02942 (anaerobic ribonucleoside triphosphate redu 135 bp 5' | | SAOUHSC_02942 | 2707866 | - | 2707965 | 99 | 19.32 | 1.77E-83 |
| 2033901 | ataaatatagcaca | 2033940 | + | 22.3 | GLAM2Scan_Hit_179 | SAOUHSC_02169 (chemotaxis-inhibiting protein CHIPS) | 205 bp 5' | SAOUHSC_02169 | 2034054 | + | 2034054 | 0 | 5.87 | 2.24E-09 |
| 35114 | aaaaatgaagaca | 35150 | + | 22.3 | GLAM2Scan_Hit_180 | SAOUHSC_00030 | Overlap | SAOUHSC_00030 | 34712 | + | 35385 | -673 | NA | NA |
| 2731873 | aaaattatttaaata | 2731837 | - | 22.3 | GLAM2Scan_Hit_181 | SAOUHSC_A02811 / SAOUHSC_02970 | On 5' / On 3' | SAOUHSC_02970 | 2732305 | - | 2731640 | -665 | 15.69 | 8.77E-56 |
| 402131 | ataaatacatagat | 402170 | + | 22.3 | GLAM2Scan_Hit_182 | SAOUHSC_00398 (restriction modification system specificitysu | 5 bp 3' | - |  |  |  |  | NA | NA |
| 1964823 | aaaaatatatccac | 1964786 | - | 22.2 | GLAM2Scan_Hit_183 | SAOUHSC_02087 | Overlap | SAOUHSC_02087 | 1964729 | + | None |  | NA | NA |
| 886190 | aaaaagttaataat | 886226 | + | 22.2 | GLAM2Scan_Hit_184 | Same location as 143 SAOUHSC_00913 / SAOUHSC_00914 (2-isopropylmalate synt | 16 bp 5' / 56 bp 5' | SAOUHSC_00913 | 886173 | - | 886231 | 58 | 50.43 | 0.00E+00 |
|  |  |  |  |  |  |  |  | SAOUHSC_00914 | 886283 | + | 886231 | 52 | 50.43 | 0.00E+00 |
| 1045632 | aaaaatattttaaaa | 1045667 | + | 22.2 | GLAM2Scan_Hit_185 | SAOUHSC_01082 | On 5' | SAOUHSC_01082 | 1045629 | + | 1045581 | 48 | 39.09 | 0.00E+00 |
| 2568499 | ataaatatattactta | 2568534 | + | 22.2 | GLAM2Scan_Hit_186 | SAOUHSC_02796 / SAOUHSC_02797 | 47 bpt 5' / Overlap | SAOUHSC_02796 | 2568451 | - | 2568343 | -108 | 34.62 | 7.43E-263 |
|  |  |  |  |  |  |  |  | SAOUHSC_02797 | 2569625 | - | 2568343 | -1282 | 34.62 | 7.43E-263 |
| 140690 | aaatatattttacag | 140728 | + | 22.2 | GLAM2Scan_Hit_187 | Same location as 129 SAOUHSC_00134 / SAOUHSC_00135 | 64 bp 5' / 75 bp 5' | SAOUHSC_00134 | 140625 | - | 140683 | 58 | 23.18 | 3.35E-119 |
|  |  |  |  |  |  |  |  | SAOUHSC_00135 | 140804 | + | 140683 | 121 | 23.18 | 3.35E-119 |
| 864663 | aaaagtattttaaaa | 864698 | + | 22.2 | GLAM2Scan_Hit_188 | Same location as 52 SAOUHSC_00899 (argininosuccinate synthase) / SAOUHSC_0 153 bp 5' / 161 bp 5' | | SAOUHSC_00899 | 864509 | - | 864609 | 100 | 13.57 | 3.10E-42 |
|  |  |  |  |  |  |  |  | SAOUHSC_00900 | 864860 | + | 864609 | 251 | 13.57 | 3.10E-42 |
| 395182 | aatcatataaaaat | 395218 | + | 22.2 | GLAM2Scan_Hit_189 | SAOUHSC_00392 (superantigen-like protein 7) | 18 bp 5' | SAOUHSC_00392 | 395237 | + | 395159 | 78 | 15.75 | 3.42E-56 |
| 2281988 | gaagattatgcaag | 2281952 | - | 22.2 | GLAM2Scan_Hit_190 | SAOUHSC_02458 | Overlap | SAOUHSC_02458 | 2282528 | - | 2282077 | -451 | 42.53 | 0.00E+00 |
| 2154660 | gaaaataaattaaa | 2154624 | - | 22.2 | GLAM2Scan_Hit_191 | SAOUHSC_02320 / SAOUHSC_02321 | 55 bp 5' / 35 bp 3' | SAOUHSC_02320 | 2154568 | - | 2154715 | 147 | 5.28 | 6.47E-08 |
| 365302 | acgattaatttaaaa | 365340 | + | 22.2 | GLAM2Scan_Hit_192 | SAOUHSC_00355 / SAOUHSC_00356 | 58 bp 3' / 82 bp 5' | SAOUHSC_00356 | 365420 | + | 365328 | 92 | 29.10 | 1.80E-186 |
| 1887563 | aaaaacaaaaaa | 1887525 | - | 22.2 | GLAM2Scan_Hit_193 | SAOUHSC_01982 (ribosomal large subunit pseudouridine syn | 83 bp 3' | SAOUHSC_01982 | 1886620 | + | None |  | NA | NA |
| 2423000 | aagaacaatataat | 2423036 | + | 22.2 | GLAM2Scan_Hit_194 | SAOUHSC_02635 | Overlap | SAOUHSC_02635 | 2423202 | - | 2423298 | 96 | 32.18 | 1.53E-227 |
| 2202247 | caaaatatattgctg | 2202289 | + | 22.2 | GLAM2Scan_Hit_195 | deoD (purine nucleoside phosphorylase) | Overlap | SAOUHSC_02380 | 2202165 | + | 2202165 | 0 | 32.14 | 5.54E-227 |
| 924985 | aaatttaaattaaat | 924947 | - | 22.2 | GLAM2Scan_Hit_196 | SAOUHSC_00950 / SAOUHSC_00951 | 55 bp 3' / 99 bp bp 5' | SAOUHSC_00951 | 925085 | + | 924929 | 156 | 42.68 | 0.00E+00 |
| 2599106 | caaaataaattaaa | 2599069 | - | 22.1 | GLAM2Scan_Hit_197 | SAOUHSC_02821 (membrane spanning protein) | Overlap | SAOUHSC_02821 | 2598779 | + | 2599113 | -334 | 33.41 | 5.57E-245 |
| 2111493 | ttaaatttatcaaatt | 2111528 | + | 22.1 | GLAM2Scan_Hit_198 | Used as consensus, SAOUHSC_A02169 / srn_3950 teg16 | 4 bp 3' / On 5' | srn_3950 | 2111499 | + | 2111313 | 186 | NA | NA |
| 245100 | caaaatatgctaaa | 245136 | + | 22.1 | GLAM2Scan_Hit_199 | SAOUHSC_00223 (teichoic acid biosynthesis protein F) / ispD | 125 bp 3' / 113 bp 5' | SAOUHSC_00225 | 245250 | + | 245078 | 172 | 32.51 | 3.42E-232 |
| 682474 | ataaattaaaaatg | 682511 | + | 22.1 | GLAM2Scan_Hit_200 | SAOUHSC_00697 / SAOUHSC_00698 | 213 bp 3' / 31 bp 5' | SAOUHSC_00698 | 682543 | + | 682513 | 30 | 33.79 | 1.47E-250 |

| 0.81856 | + | 1.89E-01 | 0.64094 | + | 5.15E-02 |
| --- | --- | --- | --- | --- | --- |
| 0.68147 | + | 7.24E-03 | 1.13215 | - | 7.17E-01 |
| 38.91866 | - | 1.01E-198 | 20.9871 | - | 2.98E-51 |
| 23.43983 | - | 6.16E-62 | 13.10551 | - | 4.07E-33 |
| 0.87281 | + | 3.34E-01 | 0.62397 | + | 7.96E-02 |
| 0.87875 | + | 3.63E-01 | 0.46061 | + | 1.11E-03 |
| 1.02756 | - | 9.79E-01 | 3.76487 | - | 7.87E-11 |
| 1.51766 | - | 9.68E-02 | 0.61588 | + | 5.51E-02 |
| 5.16684 | - | 4.78E-59 | 0.89896 | + | 8.08E-01 |
| 0.41266 | + | 1.61E-05 | 0.54637 | + | 1.56E-02 |
| 0.27287 | + | 4.65E-36 | 0.03284 | + | 1.26E-33 |
| 1.19177 | - | 8.64E-01 | 0.81305 | + | 8.12E-01 |
| 0.88525 | + | 1.00E+00 | 0.97191 | + | 1.00E+00 |
| 1.07908 | - | 7.81E-01 | 2.79796 | - | 1.81E-04 |
| 2.09776 | - | 7.04E-13 | 1.41272 | - | 1.72E-01 |
| 1.10653 | - | 4.45E-01 | 1.6279 | - | 3.30E-02 |
| 1.31656 | - | 1.42E-02 | 3.15192 | - | 1.01E-07 |
| 1.37197 | - | 3.33E-03 | 0.84473 | + | 6.08E-01 |
| 2.12318 | - | 1.32E-05 | 0.92152 | + | 8.42E-01 |
| 2.70575 | - | 6.72E-07 | 1.20429 | - | 7.11E-01 |
| 0.7801 | + | 3.60E-02 | 0.68019 | + | 1.92E-01 |
| 1.01338 | - | 9.68E-01 | 1.80649 | - | 3.25E-02 |
| 41.82414 | - | 2.43E-193 | 19.03708 | - | 1.75E-49 |
| 11.41426 | - | 1.01E-69 | 10.60878 | - | 7.33E-32 |
| 1.0366 | - | 8.49E-01 | 1.24157 | - | 4.32E-01 |
| 1.28665 | - | 2.76E-02 | 12.33161 | - | 9.01E-33 |
|  |  |  |  |  |  |
| 0.8349 | + | 2.38E-01 | 0.83548 | + | 5.42E-01 |
| 0.86162 | + | 3.35E-01 | 0.79187 | + | 3.76E-01 |
| 1.75562 | - | 1.29E-07 | 8.63256 | - | 1.87E-24 |
| 16.62265 | - | 1.45E-98 | 4.2252 | - | 9.39E-10 |
| 0.87999 | + | 3.80E-01 | 0.60597 | + | 4.21E-02 |
| 7.90768 | - | 2.12E-43 | 4.80436 | - | 1.26E-12 |
|  |  | |  |  | |
| 0.85837 | + | 2.59E-01 | 0.55768 | + | 1.42E-02 |
| 65.05003 | - | 5.18E-208 | 42.8092 | - | 2.02E-71 |
| 2.63057 | - | 2.30E-04 | 2.15773 | - | 3.16E-02 |
| 1.98788 | - | 1.27E-04 | 1.5432 | - | 2.40E-01 |
| 0.90075 | + | 6.62E-01 | 1.56411 | - | 1.91E-01 |
| 1.45763 | - | 4.12E-03 | 2.08937 | - | 1.09E-03 |
| 0.3104 | + | 2.55E-19 | 0.22919 | + | 7.60E-10 |
| 0.37021 | + | 3.51E-19 | 0.72414 | + | 2.07E-01 |
| 3.25168 | - | 1.09E-32 | 1.43121 | - | 3.00E-01 |
| 0.70001 | + | 1.11E-03 | 0.52117 | + | 1.05E-02 |
| 0.88023 | + | 6.13E-01 | 1.33991 | - | 2.98E-01 |
| 1.54673 | - | 1.17E-03 | 2.03132 | - | 2.00E-03 |
| 0.59818 | + | 6.15E-07 | 0.4711 | + | 1.39E-03 |
| 1.62072 | - | 3.59E-06 | 1.8616 | - | 9.40E-03 |
| 1.05607 | - | 7.38E-01 | 0.88075 | + | 7.07E-01 |
| 1.53257 | - | 6.55E-05 | 1.10564 | - | 6.68E-01 |
| 0.80145 | + | 6.86E-02 | 0.63305 | + | 7.91E-02 |
| 1.21781 | - | 9.76E-02 | 0.476 | + | 2.77E-03 |
| 18.61809 | - | 3.92E-150 | 36.63207 | - | 4.17E-64 |
| 2.25465 | - | 2.45E-14 | 21.83322 | - | 4.41E-44 |
| 1.23034 | - | 8.37E-02 | 0.62525 | + | 5.53E-02 |
| 1.22628 | - | 1.08E-01 | 0.93839 | + | 8.65E-01 |
|  |  | | | | |

(B)

| **Group 1**  **Described in literature**  **14 targets** | **Group 2**  **ChIP-Seq**  **43 targets** | **Group 3**  **RNA-Seq**  **38 targets** | **Group 4**  **ChIP-Seq &**  **RNA-Seq**  **24 targets** | **Group 5**  **Literature and either RNA or ChIP-Seq**  **11 targets** | **Group 6**  **RNA-Seq & ChIP-Seq &**  **Literature**  **5 targets** | **Group 7**  **Newly identified targets**  **51 targets** |
| --- | --- | --- | --- | --- | --- | --- |
| SAOUHSC_00070 | SAOUHSC_00047 | SAOUHSC_00047 | SAOUHSC_00047 | SAOUHSC_00070 | SAOUHSC_00257 | SAOUHSC_00049 |
| SAOUHSC_00257 | SAOUHSC_00070 | SAOUHSC_00061 | SAOUHSC_00105 | SAOUHSC_00257 | SAOUHSC_00544 | SAOUHSC_00135 |
| SAOUHSC_00544 | SAOUHSC_00105 | SAOUHSC_00105 | SAOUHSC_00106 | SAOUHSC_00544 | SAOUHSC_00818 | SAOUHSC_00225 |
| SAOUHSC_00818 | SAOUHSC_00106 | SAOUHSC_00106 | SAOUHSC_00256 | SAOUHSC_00818 | SAOUHSC_00913 | SAOUHSC_00317 |
| SAOUHSC_00830 | SAOUHSC_00256 | SAOUHSC_00256 | SAOUHSC_00257 | SAOUHSC_00830 | SAOUHSC_00992 | SAOUHSC_00318 |
| SAOUHSC_00913 | SAOUHSC_00257 | SAOUHSC_00257 | SAOUHSC_00300 | SAOUHSC_00913 |  | SAOUHSC_00329 |
| SAOUHSC_00992 | SAOUHSC_00299 | SAOUHSC_00300 | SAOUHSC_00544 | SAOUHSC_00992 |  | SAOUHSC_00333 |
| SAOUHSC_01121 | SAOUHSC_00300 | SAOUHSC_00435 | SAOUHSC_00808 | SAOUHSC_01121 |  | SAOUHSC_00344 |
| SAOUHSC_01941 | SAOUHSC_00544 | SAOUHSC_00544 | SAOUHSC_00818 | SAOUHSC_01941 |  | SAOUHSC_00356 |
| SAOUHSC_02004 | SAOUHSC_00695 | SAOUHSC_00808 | SAOUHSC_00913 | SAOUHSC_02004 |  | SAOUHSC_00391 |
| SAOUHSC_02171 | SAOUHSC_00698 | SAOUHSC_00818 | SAOUHSC_00923 | SAOUHSC_02171 |  | SAOUHSC_00392 |
| SAOUHSC_02862 | SAOUHSC_00808 | SAOUHSC_00913 | SAOUHSC_00975 |  | | SAOUHSC_00434 |
| SAOUHSC_02978 | SAOUHSC_00817 | SAOUHSC_00923 | SAOUHSC_00992 |  |  | SAOUHSC_00543 |
| SAOUHSC_03001 | SAOUHSC_00818 | SAOUHSC_00975 | SAOUHSC_01121 |  |  | SAOUHSC_00605 |
|  | SAOUHSC_00830 | SAOUHSC_00989 | SAOUHSC_01289 |  |  | SAOUHSC_00659 |
|  | SAOUHSC_00913 | SAOUHSC_00992 | SAOUHSC_01452 |  |  | SAOUHSC_00731 |
|  | SAOUHSC_00914 | SAOUHSC_01289 | SAOUHSC_01584 |  |  | SAOUHSC_00845 |
|  | SAOUHSC_00922 | SAOUHSC_01395 | SAOUHSC_01917 |  |  | SAOUHSC_00853 |
|  | SAOUHSC_00923 | SAOUHSC_01452 | SAOUHSC_01918 |  |  | SAOUHSC_00899 |
|  | SAOUHSC_00928 | SAOUHSC_01584 | SAOUHSC_01923 |  |  | SAOUHSC_00900 |
|  | SAOUHSC_00951 | SAOUHSC_01917 | SAOUHSC_01941 |  |  | SAOUHSC_00912 |
|  | SAOUHSC_00975 | SAOUHSC_01918 | SAOUHSC_01944 |  |  | SAOUHSC_01037 |
|  | SAOUHSC_00991 | SAOUHSC_01923 | SAOUHSC_02667 |  |  | SAOUHSC_01128 |
|  | SAOUHSC_00992 | SAOUHSC_01941 | SAOUHSC_02696 |  |  | SAOUHSC_01246 |
|  | SAOUHSC_01082 | SAOUHSC_01944 |  | | | SAOUHSC_01264 |
|  | SAOUHSC_01289 | SAOUHSC_02167 |  |  |  | SAOUHSC_01265 |
|  | SAOUHSC_01297 | SAOUHSC_02169 |  |  |  | SAOUHSC_01385 |
|  | SAOUHSC_01301 | SAOUHSC_02171 |  |  |  | SAOUHSC_01439 |
|  | SAOUHSC_01452 | SAOUHSC_02667 |  |  |  | SAOUHSC_01511 |
|  | SAOUHSC_01584 | SAOUHSC_02668 |  |  |  | SAOUHSC_01512 |
|  | SAOUHSC_01705 | SAOUHSC_02696 |  |  |  | SAOUHSC_01539 |
|  | SAOUHSC_01917 | SAOUHSC_02704 |  |  |  | SAOUHSC_01653 |
|  | SAOUHSC_01918 | SAOUHSC_02740 |  |  |  | SAOUHSC_02245 |
|  | SAOUHSC_01923 | SAOUHSC_02855 |  |  |  | SAOUHSC_02320 |
|  | SAOUHSC_02004 | SAOUHSC_02857 |  |  |  | SAOUHSC_02464 |
|  | SAOUHSC_02549 | SAOUHSC_02923 |  |  |  | SAOUHSC_02525 |
|  | SAOUHSC_02550 | SAOUHSC_02942 |  |  |  | SAOUHSC_02590 |
|  | SAOUHSC_02668 | SAOUHSC_02979 |  |  |  | SAOUHSC_02591 |
|  | SAOUHSC_02669 |  | | | | SAOUHSC_02619 |
|  | SAOUHSC_02695 |  |  |  |  | SAOUHSC_02784 |
|  | SAOUHSC_02696 |  |  |  |  | SAOUHSC_02796 |
|  | SAOUHSC_02815 |  |  |  |  | SAOUHSC_02831 |
|  | SAOUHSC_02885 |  |  |  |  | SAOUHSC_02838 |
|  |  |  |  |  |  | SAOUHSC_02861 |
|  |  |  |  |  |  | SAOUHSC_03030 |
|  |  |  |  |  |  | SAOUHSC_A02811 |
|  |  |  |  |  |  | *srn_5010_teg33* |
|  |  |  |  |  |  | *srn_1530 sRNA133* |
|  |  |  |  |  |  | *srn_1270_teg44* |
|  |  |  |  |  |  | *srn_0440 rsaK* |
|  |  |  |  |  |  | *srn_3470* |
|  |  |  |  |  |  | *srn_0380_teg140_sRNA23* |

Table S6: (A) Glams2 scan analysis: 200 first *S. aureus* genome hits (GLAM2Scan results and their ties to possible regulatory targets for SarA (addition of RNA-Seq and ChIP-seq data when available)).The 15 sRNA promoter sequences used to generate the Glasm2 consensus are presented below

(B) Classification of SarA potential targets revealed by Glam2 Scan into 7 groups
